# Standardizing Proteomics Workflow for Liquid Chromatography-Mass Spectrometry: Technical and Statistical Considerations

**DOI:** 10.35248/0974-276x.19.12.496

**Published:** 2019-04-04

**Authors:** Sudhir Srivastava, Michael Merchant, Anil Rai, Shesh N. Rai

**Affiliations:** 1 Centre for Agricultural Bioinformatics, ICAR-Indian Agricultural Statistics Research Institute, New Delhi, India; 2 Department of Bioinformatics & Biostatistics, University of Louisville, Louisville, Kentucky, United States of America; 3 Department of Medicine, University of Louisville, Louisville, Kentucky, United States of America; 4 Department of Pharmacology & Toxicology, University of Louisville, Louisville, Kentucky, United States of America; 5 Biostatistics and Bioinformatics Facility, James Graham Brown Cancer Center, University of Louisville, Louisville, Kentucky, United States of America

**Keywords:** ANOVA, Imputation, Proteins, Tissue storage, Tissue extraction, Technical variability

## Abstract

**Introduction:**

The quantitative measurements based on liquid chromatography (LC) coupled with mass spectrometry (MS) often suffer from the problem of missing values and data heterogeneity from technical variability. We considered a proteomics data set generated from human kidney biopsy material to investigate the technical effects of sample preparation and the quantitative MS.

**Methods:**

We studied the effect of tissue storage methods (TSMs) and tissue extraction methods (TEMs) on data analysis. There are two TSMs: frozen (FR) and FFPE (formalin-fixed paraffin embedded); and three TEMs: MAX, TX followed by MAX and SDS followed by MAX. We assessed the impact of different strategies to analyze the data while considering heterogeneity and MVs. We have used analysis of variance (ANOVA) model to study the effects due to various sources of variability.

**Results and Conclusion:**

We found that the FFPE TSM is better than the FR TSM. We also found that the one-step TEM (MAX) is better than those of two-steps TEMs. Furthermore, we found the imputation method is a better approach than excluding the proteins with MVs or using unbalanced design.

## Introduction

Proteins are important biological macromolecules performing a wide variety of functions. The proteome can be defined as the entire set of proteins translated and/or modified within a living organism [1,2]. Proteomics more generally refers to large-scale LC-MS based discovery studies designed to address both quantitative and qualitative aspects of the proteome in question. Now proteomics has emerged as a powerful tool across various fields such as biomedicine mainly applied to diseases, agriculture and animal sciences [3–10]. The practical application of proteomics includes expression proteomics, structural proteomics, biomarker discovery, interaction proteomics, protein networks, etc. [11,12]. Here, we are dealing with proteomic expression data that are generated by using high throughput technologies usually involving MS [13–18]. LC-MS is used in proteomics as a method for identification and quantification of peptides and proteins in complex mixtures [19,20]. There are two basic proteomics approaches, namely bottom-up and top-down [10,21]. The most common proteomics approach is the bottom-up in which proteins in a sample are enzymatically digested into peptides and subjected to chromatographic separation, ionization and mass analysis. In the top-down approach, intact proteins are introduced into MS where they are subjected to fragmentation. Further, the quantification of peptides/proteins may be either label-free or labelled (metabolic, enzymatic, or chemical) to detect differences in protein abundances among different conditions [22–25]. In label-free quantification, MS ion intensity (peak area) and spectral counting of features are the major approaches. Conversely, top-down proteomics addresses the study of intact proteins and consequently is most often used to address purified or partially purified proteins [26]. Here, we are dealing with the bottom-up approach in which peak area values have been used in label-free quantification of proteins. Various approaches exist for proteomics data analysis in which the first step is to summarize the intensities of all features using a quantitative summary followed by some transformation such as log transformation to approximate it to normal distribution. However, each of these methods has several drawbacks which can be studied by examining the statistical properties of these methods [27–29]. When a data set contains an equal number of subjects in each group, and when features have no missing observations, the data set is called balanced. It is not always the condition; sometimes the data can be unbalanced, having an unequal number of subjects, or missing observations, or both. MVs in proteomics data can occur due to biological and/or technical issues. These are of three types: (i) missing completely at random (MCAR) in which MVs are independent of both unobserved and observed data; (ii) missing at random (MAR) if conditional on the observed data, the MVs are independent of the missing measurements; and (iii) missing not at random (MNAR) when data is neither MCAR nor MAR [30]. The data with missing observations can be analyzed either by excluding the features having missing observations, by using statistical methods that can handle unbalanced data, or by using imputation methods. If the features having missing observations are excluded, then there is loss of information from the experiment. Therefore, the use of methods that can handle MVs, such as imputation methods, are generally preferred. However, the use of imputation methods may lead to wrong interpretation and still these methods are questionable in statistical terms [31,32]. The data set usually consists of biological replicates only or both biological and technical replicates. Biological variability arises from genetic and environmental factors; it is intrinsic to all organisms. The technical approaches include sample collection and storage, sample preparation, extraction, LC separation and MS detection [20]. Sometimes, variations in the biological data or technical approaches to data collection lead to heterogeneity for the samples under study [33,34]. We performed analysis of laser capture microdissection (LCMD)-LCMS high-resolution proteomics dataset using multifactor ANOVA model. We studied the variability in the data based on different TSMs and TEMs. We estimated the contribution of various sources of variation to the overall variability. The study of data variability was done using various analysis methods and transformation and/or normalization techniques. In this paper, we investigated the technical effects of sample preparation and the quantitative MS resulting in heterogeneity for low abundant protein quantification. This will improve the biomarker discovery studies utilizing limited bioreposited tissue resources. We have done all the statistical analysis in R [35] and codes are available from the authors on request.

## Methods

### Proteomics experiment

Data for the methods used in the collection, extraction, and proteomic analysis have previously been published under Hobeika L et al. [36]. Individual data files for MS data (.RAW), peak lists (.mgf), and compressed search results (.mzIdentML) files can be downloaded from the MasslVE data repository (http://massive.ucsd.edu/; MasslVE ID: MSV000079914) and ProteomeXchange data repository [37] (http://www.proteomexchange.org/; ID: PXD004601). For consideration of variability of the feature detection and MVs abbreviated methods for these studies are provided below.

#### Tissue collection:

FR and FFPE tissue from the same human kidney unsuitable for transplant were cut into 10 μm sections on Polyethylene terephthalate membrane frame slides, stained with Mayer’s hematoxylin and glomerular tissue compartments isolated using a Leica LMD6500 Laser Microdissection System.

#### Protein extraction:

Experiments were conducted to compare a single tissue solubilization step using an acid labile surfactant to approaches for tissue decellularization. The single step method used the acid-labile surfactant Protease MAX surfactant with heating (MAX). Two tissue decellularization methods incorporated sequential decellularization with solubilization of the residual pellet with MAX. First tissue decellularization approach used 0.4% SDS + HALT protease/phosphatase inhibitor cocktail (Thermo Fisher) followed by solubilization of residual “ECM” pellet using MAX (SDS.MAX). Second tissue decellularization approach used sequential decellularization with 25mM NH4OH/ 0.5%TritonX-100 (TX) followed by solubilization of residual “ECM” pellet using MAX (TX.MAX). As described in Hobeika L et al. [36], the tryptic peptides were analyzed using a LC-MS Orbitrap ELITE approach with peptide assignments using a Mascot/Sequest search strategy. Scaffold4 was used to set false discovery rate (FDR) control. Finally, we obtained a label-free quantified data of identified proteins (Supplementary File 1). Please see more details about the experimental procedures in “Supplementary File 2”. We analyzed the data for comparing statistical methods with MVs in the presence of heterogeneity.

### Proteomics data analysis

The purpose of this study is to (1) compare variability between (a) tissue storage methods (TSMs) and (b) tissue extraction methods (TEMs); (2) compare various statistical approaches of analysis and normalization methods.

**Figure 1:**
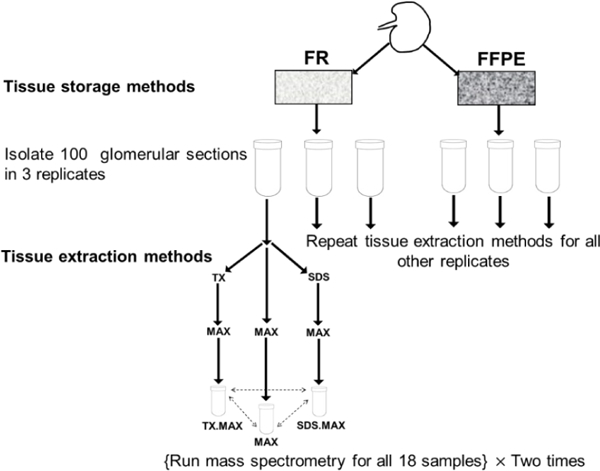
Flowchart of the experiment.

We have two TSMs (FR and FFPE) and three TEMs (MAX, TX.MAX, SDS.MAX) with three replicates and two MS runs leading to 36 samples (total number of samples = 2 × 3 × 3 × 2 = 36). A flow chart of the experiment is given below in Figure 1.

In the above flowchart, we have shown the basic steps of carrying out the experiment involving TSMs and TEMs. We have repeated the MS two times to get more reliable results for estimating experimental variability. We obtained the following six groups as given below in the Table 1. There are three replicates for each of the six groups thus leading to 18 samples. Then, we have repeated the MS two times for the 18 samples and we obtained six samples for each of the six groups.

#### Data preprocessing:

Initially, there were 728 proteins identified in both runs, 380 proteins identified in run 1 only and 342 proteins identified in run 2 only. There was a total of 1450 identified proteins out of which 1376 proteins were unique, and 37 proteins were redundant and duplicate entries were removed from the data. Furthermore, there were 111 proteins for which all the samples have NA values (MVs). Therefore, we are left with protein data with 1302 proteins that correspond to 1178 gene symbols (Supplementary File 1). The percentage of NA values within each sample (36 samples) ranges from 41.3%−78.3% with a median value of 49.5%. As we have a greater number of groups, therefore it is difficult to perform analysis with this data having MVs. If we discard the proteins having any MVs in any of the samples in a group, then there will be only 26 proteins available. Another way is to retain the proteins having at least one or two observations in each group. A summary of number of proteins available in each group is given below in Table 2. If we use the number of proteins having at least one observation in a group, then we can assess a greater number of proteins. However, we need at least two observations in each group to calculate CV for a protein in each group. Therefore, we used 372 proteins which have at least two observations in each of the six groups for further analysis.

#### Statistical approaches:

The analysis of proteomics data becomes more complex due to non-normality behavior of the data, and greater proportion of MVs within and across the samples. To get a better insight of proteomics data analysis while dealing with these problems, we have performed the analysis using three methods as given below:

#### A1. Method for data excluding missing values:

Proteins having complete observations for all the samples, i.e., no MVs, were used for comparison. Proteins having MVs were discarded from the analysis.

#### A2. Method for data including missing values:

The proteins with MVs across the samples were analyzed using unbalanced ANOVA method [38].

#### A3. Method for data using imputation:

The MVs were imputed after applying the normalization methods to the data [39] as given in next section. We have used the “impute. MAR” function of the R package “imputeLCMD” [40] for imputing the MVs. Three different types of imputation under the assumption of MAR or MCAR, namely, MLE [41], SVD [42] and KNN [43,44] are available in this package. We have used only the SVD method **(A3)** for imputation.

We applied three different data transformation and/or normalization methods:

#### N1. Logarithmic transformation:

The raw data is transformed by using logarithmic base 2.

#### N2. Quantile normalization:

It is done by using log base 2 transformation of raw data followed by “normalize.quantiles” method [45] available in R package “preprocessCore” [46].

#### N3. Variance stabilizing normalization:

It is done by applying “justvsn” function available in R package “vsn” [47] to the raw data.

Therefore, by using three methods of analysis (Al, A2 and A3) based on three transformation and/or normalization methods (N1, N2 and N3), we have 9 different combinations (statistical approaches): excluding MVs (A1.N1, A1.N2, A1.N3); including MVs (A2.N1, A2.N2, A2.N3); imputing MVs (A3.N1, A3.N2, A3.N3). We preprocessed the data using these methods to get 9 different datasets (preprocessed data) for 6 groups having 6 samples in each group. We calculated the coefficient of variation (CV) for each protein in the groups: TSM (FR *vs*. FFPE), TEM (MAX *vs*. TX.MAX *vs*. SDS. MAX) and TSM×TEM (FR_MAX, FR_TX.MAX, FR_SDS.MAX, FFPE_MAX, FFPE_TX.MAX, FFPE_SDS.MAX). It has two purposes: (i) Which TSM/ TEM/ TSM×TEM have the minimum CV based on different statistical approaches; (ii) Which statistical approach leads to the minimum CV. We have used ANOVA model as given below for studying the contribution of variability due of TSM, TEM and the interaction term TSM×TEM:

(1) $$y_{ijk}=\mu +\alpha_{i}+\beta_{j}+{\left(\alpha \beta \right)}_{ij}+\varepsilon_{ijk}$$

where, *y*_*ijk*_ is the transformed and/or normalized data for a protein, *α*_*i*_(*i*=1,2) is the *j*^*th*^ TSM effect,*β*_*j*_, (*j* = 1,2,3)is the *j*^*th*^ TEM effect and (*αβ*)_*ij*_ is the interaction effect, TSM×TEM. The term *ε*_*ijk*_ is the normally distributed error component and $\varepsilon_{ijk}\sim N\left(0,\sigma^{2}\right)$. The mapping of the above model to the experimental design allows us to estimate the contribution due to each source of variation for each protein.

## Results and Discussion

### Comparison of CV among various groups

We have 141, 372 and 372 proteins obtained by using the analysis methods Al, A2 and A3, respectively. The summary of CV using 9 different statistical approaches for comparisons among TSMs and TEMs is shown below in Table 3. The summary of CV using 9 different statistical approaches for comparisons among six groups of TSM×TEM is shown below in Table 4.

#### TSM:

We found that median value of CV is lowest in FFPE using all the statistical approaches. Furthermore, within FFPE, the normalization method N3 has the minimum value of median CV for each analysis method. Overall, the minimum median CV is for A1.N3 in FFPE.

#### TEM:

We have the minimum median value of CV in TX.MAX. We found A1.N2 has the minimum value of median CV.

#### TSM×TEM:

We have the minimum median value of CV in FR_ MAX followed by FFPE_SDS.MAX using all the approaches. We found A1.N3 has the minimum value of median CV in all the groups except for A1.N2 in FR_SDS.MAX. Overall, the minimum median CV is for A1.N3 in group FR_MAX.

Based on median CV, FFPE is a better choice than FR using all the statistical approaches. Similarly, among TSMs, TX.MAX has the least CV and can be a better choice. However, based on the maximum value of CV, MAX is a better choice for TEM. If we consider approaches (A2 & A3) having greater number of proteins and TEM within FFPE, we see that A3.N3 in FFPE_SDS.MAX is having the least median CV (1.63).

### Contribution of Sum of Squares (SS) due to each component

The percent contribution of SS due to each variable to the total SS was computed for each protein. A summary of contribution of each variable to the total variability is given below in Table 5. We found that the TSM has the least contribution to the total variability whereas interaction term has the maximum contribution (SS_tsm_ < SS_TEM_ < SS_TSM×TEM_). The imputation method leads to decrease in the SS contribution due to each variable. The proportion of proteins showing significant effects due to TSM, TEM and TSM×TEM using 9 different approaches are given below Table 6. The proportion of proteins showing significant effects due to TSM and TEM and their interaction vary with each statistical approach. The TSM has the least proportion of significant proteins as compared to those of TEM and TSM×TEM. This shows that TSM has the least influence. Furthermore, the imputation approach has the least proportion of significant proteins. This shows that imputation of MVs is a better approach for analysis as it leads to reduction in variability and increase in the number of proteins assessed for analysis.

### Analysis for imputed data using VSN

**Figure 2:**
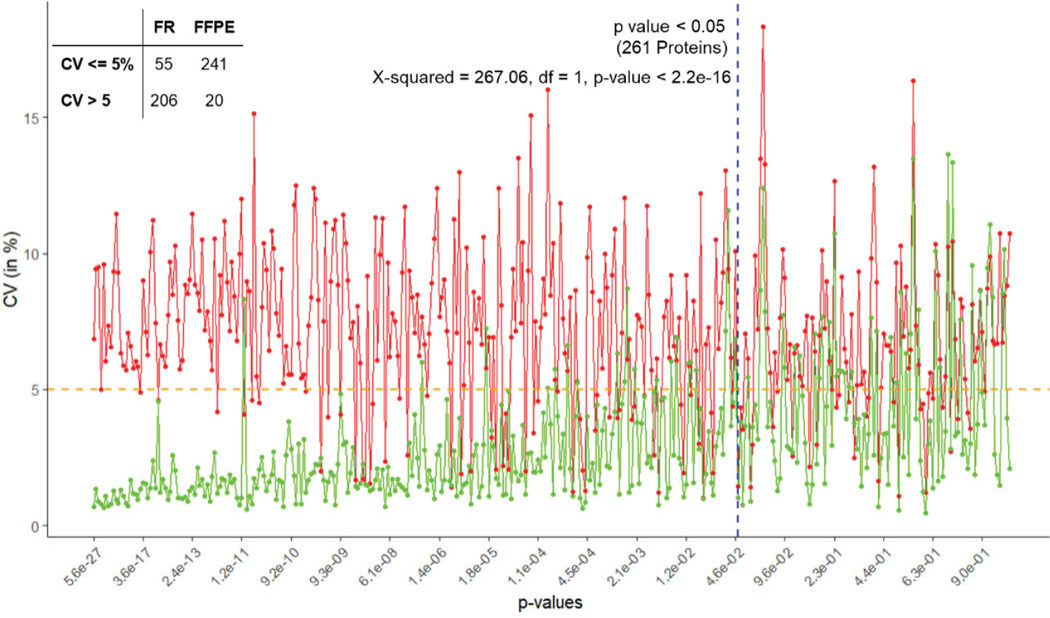
Plot of CV (in %) versus the proteins with increasing order of p-values for TSM (FR – red and FFPE – green).

**Figure 3:**
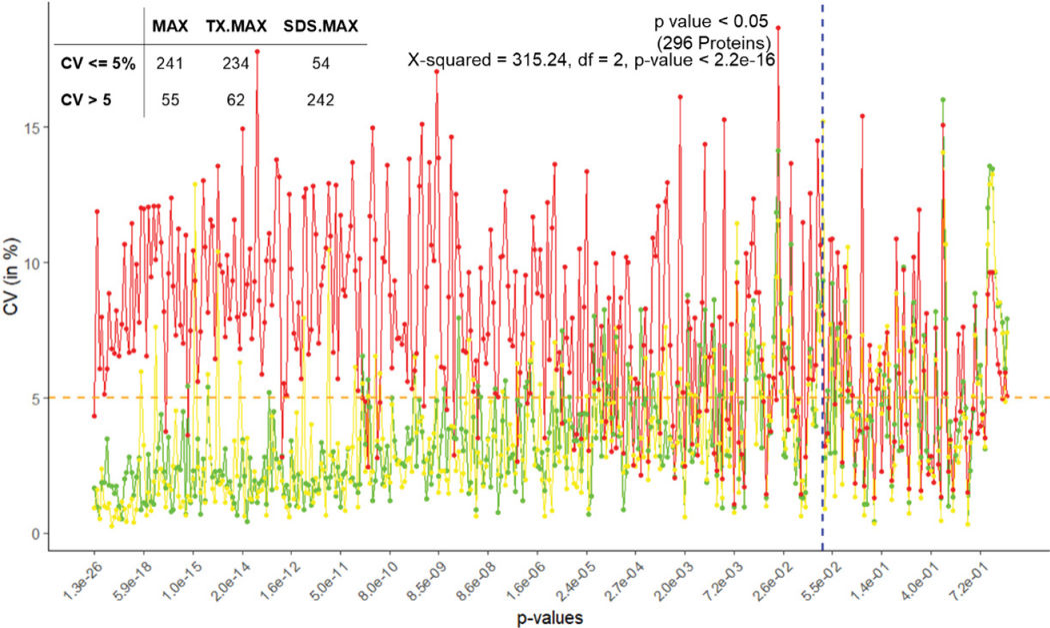
Plot of CV (in %) versus the proteins with increasing order of p-values for TSM (MAX – green, TX.MAX – yellow and SDS.MAX – red).

We used ANOVA to test the significance of proteins based on TSM and TEM. The plot of CV of the proteins in increasing order of p-values based on A3.N3 for TSM and TEM are respectively given below in Figures 2 and 3. There are respectively 261 and 296 proteins showing significant effects due to TSM and TEM. From Figure 2, we see that FR has more CV as compared to that of FFPE for most of the proteins. From Figure 3, we found SDS.MAX has more CV as compared to those of MAX and TX.MAX. We applied chi-square test for the proteins having significant effects due to TSM and TEM. We found that there is association between the TSM and the CV (p-value < 0.001). Similarly, in case of TSM, we found that there is association between the variables, TEM and CV (p-value < 0.001). We found that the FFPE is a better method than that of the FR for tissue storage. Further, we found that MAX, the single step approach is better than those of two-step approach for tissue extraction. The maximum contribution to the total variability is due to the interaction effect TSM×TEM and TEM. The TSMs and TEMs have significant effects on the protein expression. However, the effect due to TSM is the least. In the present article, we have used different analysis and normalization methods for the proteomics data. The number of proteins for testing can be increased by either by including the MVs (A2) or by using imputed data (A3). The imputation method (A3) has the least SS contribution than those of A1 (complete data) and A2 (unbalanced data). We found the least proportion of significant proteins when using the imputation method (A3). The normalization method N1, i.e., only logarithmic transformation is not suited for analyzing the proteomics data. The other normalization methods N2 and N3 having lesser CV can be a better approach.

## Conclusion

Our study discussed the technical issues with a focus on the statistical analysis. It will provide better insight to the researchers while designing and executing experiments. There may be small changes caused during sample handling and storage, different batches of buffer, electrospray, instrument components, calibration and tuning, etc. While designing any proteomics experiment, we must identify the technical steps with large variability. Therefore, it becomes necessary to understand the data heterogeneity due to biological variability and technical variability of the proteomics methods at each step. We have made the proteomics data available (Supporting file 1). The researchers involved in proteomics research area can use this data for further study. The data can further be used for planning new proteomics experiments. In the future, we will come up with a rigorous statistical approach using different proteomics dataset that could overcome the heterogeneity problem caused due to technical reasons in the proteomics data with MVs. Finally, we can recommend: (i) FFPE is the better choice than FR for tissue storage, (ii) one-step TEM is better than the two-step TEM, (iii) Imputation method (A3) is the best approach, (iv) N2 or N3 method of normalization should be the preferred choice.

## Supplementary Material

Suppl file 1 A label-free quantified data of identified proteins.

suppl file 2 More details about experimental procedures.

## Figures and Tables

**Table 1:** Table showing different groups understudy.

TSM → TEM ↓		FR	FFPE
Direct	MAX	1 (FR_MAX)	4 (FFPE_MAX)
Sequential Extraction	TX.MAX	2 (FR_TX.MAX)	5 (FFPE_TX.MAX)
	SDS.MAX	3 (FR_SDS.MAX)	6 (FFPE_SDS.MAX)

**Table 2:** Summary of number of proteins and missing values in different groups.

Groups	No. of proteins with no MVs	No. of proteins with MVs in all samples	No. of proteins with at least one observation	No. of proteins with at least two observations
FR_MAX	448	205	1097	995
FR_TX.MAX	357	324	978	881
FR_SDS.MAX	170	678	624	454
FFPE_MAX	373	295	1007	874
FFPE_TX.MAX	353	261	1041	890
FFPE_SDS.MAX	381	237	1065	920

**Table 3:** Summary of CV using 9 statistical approaches among TSM and TEM.

		TSM		TEM		
		FR	FFPE	MAX	TX.MAX	SDS.MAX
**MV Excluded**	**A1.N1**	6.92 (2.23, 12.77)	2.76 (2.00, 9.49)	3.25 (1.93, 9.64)	3.26 (2.05, 15.90)	7.40 (2.38, 15.24)
	**A1.N2**	6.29 (0.52, 12.50)	1.30 (0.55, 5.10)	1.94 (0.34, 9.02)	1.91 (0.32, 12.67)	6.74 (0.59, 14.99)
	**A1.N3**	6.25 (0.95, 12.51)	1.28 (0.48, 8.31)	2.03 (0.28, 9.33)	1.95 (0.26, 15.21)	6.81 (1.05, 15.01)
**MV Included**	**A2.N1**	7.08 (1.23, 12.77)	2.92 (0.83, 11)	3.50 (0.65, 12.21)	3.49 (0.73, 15.90)	7.53 (0.23, 16.95)
	**A2.N2**	6.62 (0.39, 12.51)	1.75 (0.52, 9.13)	2.71 (0.16, 12.16)	2.49 (0.32, 14.42)	7.17 (0.42, 16.48)
	**A2.N3**	6.68 (0.80, 12.49)	1.73 (0.47, 11.16)	2.71 (0.28, 11.61)	2.55 (0.20, 15.22)	7.21 (0.76, 15.54)
**MV Imputed**	**A3.N1**	7.72 (2.23, 17.47)	3.29 (1.70, 15.28)	4.03 (1.79, 15.49)	3.87 (1.72, 15.90)	8.03 (2.38, 18.01)
	**A3.N2**	7.10 (0.39, 15.96)	2.15 (0.52, 13.60)	3.10 (0.45, 14.64)	2.98 (0.38, 14.42)	7.35 (0.56, 19.25)
	**A3.N3**	7.07 (1.01, 18.34)	2.13 (0.47, 13.64)	3.10 (0.33, 16.02)	3.04 (0.28, 15.22)	7.35 (1.08, 18.68)

**Table 4:** Summary of CV using 9 statistical approaches among six groups of TSM×TEM.

		FR_	FR_	FR_	FFPE_	FFPE_	FFPE_
		MAX	TX.MAX	SDS.MAX	MAX	TX.MAX	SDS.MAX
**MV Excluded**	**A1.N1**	2.64 (1.34, 8.62)	2.71 (0.83, 9.95)	4.73 (2.25, 12.90)	3.00 (1.96, 7.14)	2.87 (2.08, 13.80)	2.34 (0.75, 8.34)
	**A1.N2**	0.87 (0.12, 6.26)	1.05 (0.18, 9.09)	2.32 (0.22, 10.55)	0.87 (0, 5.12)	0.96 (0, 7.28)	0.85 (0, 8.13)
	**A1.N3**	0.77 (0.17, 7.53)	1.01 (0.13, 9.87)	2.37 (0.32, 11.92)	0.84 (0.12, 6.14)	0.95 (0.18, 11.75)	0.83 (0.10, 8.10)
**MV Included**	**A2.N1**	2.64 (0.05, 11.71)	2.81 (0.14, 10.93)	4.49 (0.03, 19.81)	2.97 (0.09, 13.33)	3.01 (0.15, 13.8)	2.41 (0.17, 17.14)
	**A2.N2**	1.08 (0, 10.62)	1.47 (0, 9.33)	2.88 (0.07, 16.32)	1.28 (0, 10.50)	1.32 (0, 12.62)	1.14 (0, 13.32)
	**A2.N3**	1.09 (0.04, 9.67)	1.39 (0.04, 9.87)	2.44 (0.02, 17.52)	1.28 (0.01, 9.55)	1.41 (0.12, 12.45)	1.19 (0.07, 17.72)
**MV Imputed**	**A3.N1**	2.94 (0.95, 16.56)	3.26 (0.83, 15.27)	5.06 (2.25, 17.75)	3.40 (1.34, 16.87)	3.33 (0.62, 15.62)	2.86 (0.69, 16.21)
	**A3.N2**	1.59 (0.24, 17.06)	1.83 (0.06, 14.28)	2.77 (0.20, 19.86)	1.78 (0.02, 15.03)	1.70 (0.02, 14.08)	1.75 (0.03, 14.23)
	**A3.N3**	1.57 (0.14, 19.00)	1.82 (0.19, 15.69)	2.48 (0.32, 17.28)	1.74 (0.07, 14.88)	1.7 (0.21, 14.28)	1.63 (0.16, 15.38)

**Note:** The first figure is the median value and the figures inside the parenthesis are respectively, minimum and maximum value.

**Table 5:** Summary of the contribution of % SS due to TSM, TEM and TSM×TEM.

		SS_TSM_	SS_TEM_	SS_TSM×TEM_
**MV Excluded**	**A1.N1**	9.86 (0, 68.98)	20.9 (0.47, 36.32)	32.87 (0.29, 54.41)
	**A1.N2**	14.71 (0, 78.88)	27.49 (1.35, 48.44)	43.21 (0.92, 64.54)
	**A1.N3**	15.05 (0, 73.78)	26.7 (2.31, 44.92)	41.88 (0.59, 65.23)
**MV Included**	**A2.N1**	10.84 (0, 83.65)	20.97 (0.08, 49.47)	33.46 (0.29, 78.05)
	**A2.N2**	12.59 (0, 85)	25.56 (0.06, 54.68)	39.37 (0.08, 80.29)
	**A2.N3**	12.84 (0, 88.18)	25.72 (0.04, 53.37)	40.32 (0.06, 77.54)
**MV Imputed**	**A3.N1**	8.52 (0, 73.76)	18.83 (0, 40.46)	29.86 (0.09, 57.77)
	**A3.N2**	11.07 (0, 85.67)	23.53 (0.03, 50.93)	37.33 (0.05, 65.75)
	**A3.N3**	11.18 (0, 85.31)	23.32 (0, 49.68)	37.26 (0.14, 65.32)

**Table 6:** The summary of proportion of proteins showing effects due to the variables: TSM, TEM and TSM×TEM.

		N_TSM_	N_TEM_	N_TSM×TEM_
**MV Excluded**	**A1.N1**	0.65/ 0.62/ 0.33	0.77/ 0.76/ 0.5	0.77/ 0.77/ 0.65
	**A1.N2**	0.84/ 0.84/ 0.72	0.91/ 0.91/ 0.77	0.89/ 0.88/ 0.78
	**A1.N3**	0.82/ 0.82/ 0.71	0.87/ 0.87/ 0.72	0.87/ 0.85/ 0.77
**MV Included**	**A2.N1**	0.61/ 0.57/ 0.25	0.72/ 0.72/ 0.28	0.79/ 0.79/ 0.49
	**A2.N2**	0.75/ 0.73/ 0.48	0.83/ 0.82/ 0.58	0.87/ 0.87/ 0.68
	**A2.N3**	0.74/ 0.74/ 0.52	0.81/ 0.81/ 0.6	0.85/ 0.84/ 0.67
**MV Imputed**	**A3.N1**	0.58/ 0.53/ 0.24	0.69/ 0.67/ 0.35	0.78/ 0.77/ 0.52
	**A3.N2**	0.71/ 0.68/ 0.48	0.81/ 0.8/ 0.58	0.86/ 0.85/ 0.69
	**A3.N3**	0.7/ 0.69/ 0.49	0.8/ 0.78/ 0.58	0.84/ 0.83/ 0.67

**Note:** The result obtained using p-values corresponding to without adjustment, BH adjusted and Bonferroni adjusted are separated serially by slash “/” in the table.

## List of Abbreviations:

LC: Liquid Chromatography
MS: Mass Spectrometry
ANOVA: Analysis of Variance
MCAR: Missing Completely at Random
MAR: Missing at Random
MNAR: Missing not at Random
MVs: Missing Values
TSM: Tissue Storage Method
FFPE: Formalin-Fixed Paraffin Embedded
FR: Frozen
TEM: Tissue Extraction Method
MAX: Protease MAX
TX: Triton X-100
SDS: Sodium Dodecylsulfate
LCMD: Laser Capture Microdissection
ETD: Electron-Transfer Dissociation
CID: Collision-Induced Dissociation
cRAP: Common Repository of Adventitious Proteins
FDR: False Discovery Rate
SS: Sum of Squares
CV: Coefficient of Variation
