# Supplementary material for: Standardizing Proteomics Workflow for Liquid Chromatography-Mass Spectrometry: Technical and Statistical Considerations
Source: J Proteomics Bioinform. Author manuscript; Available in PMC 2020 Mar 6. (PMC7059694; doi:10.35248/0974-276x.19.12.496)
Supplement: Suppl file 1 — A label-free quantified data of identified proteins. [file NIHMS1032169-supplement-Suppl_file_1.pdf]

Accession.I	Identified.I	Gene.Symb	Molecular.	FR_MAX FR2.1	FR_MAX FR5.1	FR_MAX FR9.1	FR_MAX FR2.2	FR_MAX FR5.2
sp|P13647	Keratin, typ	KRT5	62 kDa	4.26E+09	4.39E+08	1.09E+09	2.13E+09	3.17E+08
sp|P35579	Myosin-9	MYH9	227 kDa	5.43E+09	8.59E+09	6.78E+09	2.56E+09	4.78E+09
sp|O43707	Alpha-actir	ACTN4	105 kDa	7.88E+09	1.04E+10	8.92E+09	3.32E+09	6.86E+09
sp|P98160	Basement r	HSPG2	469 kDa	2.64E+09	4.39E+09	2.87E+09	1.05E+09	2.21E+09
sp|P12111	Isoform 2 c	COL6A3	321 kDa	3.70E+09	5.60E+09	4.39E+09	1.81E+09	3.09E+09
sp|P35527	Keratin, typ	KRT9	62 kDa	1.67E+09	9.92E+08	3.04E+08	7.88E+08	4.61E+08
sp|P04264	Keratin, typ	KRT1	66 kDa	4.16E+09	1.15E+09	1.15E+09	1.72E+09	6.01E+08
sp|P08670	Vimentin	VIM	54 kDa	7.56E+09	1.24E+10	9.09E+09	3.48E+09	7.12E+09
sp|O15230	Laminin sul	LAMA5	400 kDa	2.11E+09	3.45E+09	2.64E+09	1.02E+09	1.93E+09
sp|Q9Y490	Talin-1	TLN1	270 kDa	1.63E+09	2.55E+09	1.93E+09	8.46E+08	1.44E+09
sp|P55268	Laminin sul	LAMB2	196 kDa	2.73E+09	4.08E+09	3.26E+09	1.25E+09	2.45E+09
sp|P13645	Keratin, typ	KRT10	59 kDa	4.21E+09	2.59E+08	1.30E+09	2.32E+09	2.24E+08
sp|O00468	Isoform 6 c	AGRN	215 kDa	2.26E+09	3.46E+09	2.77E+09	9.13E+08	1.90E+09
sp|P11047	Laminin sul	LAMC1	178 kDa	1.94E+09	2.98E+09	2.46E+09	7.27E+08	1.38E+09
sp|P35555	Fibrillin-1	FBN1	312 kDa	2.03E+09	3.24E+09	2.42E+09	7.54E+08	1.42E+09
sp|Q09666	Neuroblast	AHNAK	629 kDa	1.04E+09	1.81E+09	1.29E+09	4.85E+08	1.19E+09
sp|Q13813	Isoform 3 c	SPTAN1	282 kDa	6.19E+08	1.14E+09	8.01E+08	2.60E+08	6.27E+08
sp|P07437	Tubulin bet	TUBB	50 kDa	2.22E+09	2.83E+09	2.51E+09	1.10E+09	1.87E+09
sp|Q15149	Plectin	PLEC	532 kDa	3.68E+08	7.42E+08	5.44E+08	2.00E+08	4.20E+08
sp|P48681	Nestin	NES	177 kDa	7.84E+08	1.39E+09	1.10E+09	4.30E+08	9.12E+08
sp|P18206	Isoform 1 c	VCL	117 kDa	1.18E+09	1.82E+09	1.40E+09	5.20E+08	1.06E+09
sp|P14543	Nidogen-1	NID1	136 kDa	1.76E+09	2.93E+09	1.84E+09	7.24E+08	1.46E+09
sp|P02751	Isoform 14	FN1	249 kDa	7.09E+08	1.09E+09	8.16E+08	3.33E+08	6.05E+08
sp|P02675	Fibrinogen	FGB	56 kDa	2.14E+09	2.19E+09	1.32E+09	9.57E+08	1.10E+09
sp|P08572	Collagen al	COL4A2	168 kDa	1.79E+09	3.87E+09	2.70E+09	1.01E+09	1.59E+09
sp|P67936	Tropomyos	TPM4	29 kDa	1.16E+09	1.67E+09	1.38E+09	4.32E+08	8.58E+08
sp|Q01082	Spectrin be	SPTBN1	275 kDa	5.02E+08	8.11E+08	6.07E+08	2.00E+08	4.83E+08
sp|Q01955	Collagen al	COL4A3	162 kDa	1.96E+09	2.75E+09	2.04E+09	7.21E+08	1.39E+09
sp|P02545	Prelamin-A	LMNA	74 kDa	1.22E+09	1.98E+09	1.52E+09	4.98E+08	1.08E+09
sp|P53420	Collagen al	COL4A4	164 kDa	2.34E+09	3.23E+09	2.48E+09	5.61E+08	1.29E+09
sp|P02768	Serum albu	ALB	69 kDa	6.12E+08	1.02E+09	6.74E+08	2.68E+08	5.44E+08
sp|P02462	Collagen al	COL4A1	161 kDa	1.65E+09	2.71E+09	2.22E+09	7.11E+08	1.38E+09
sp|P07355	Isoform 2 c	ANXA2	40 kDa	1.32E+09	2.16E+09	1.55E+09	6.52E+08	1.19E+09
sp|P12110	Collagen al	COL6A2	109 kDa	5.04E+08	1.11E+09	8.88E+08	3.18E+08	6.17E+08
sp|P04406	Glyceraldel	GAPDH	36 kDa	9.49E+08	1.29E+09	9.19E+08	2.89E+08	8.45E+08
sp|P08473	Neprilysin	MME	86 kDa	4.61E+08	6.97E+08	5.82E+08	1.85E+08	3.71E+08
sp|P01892	HLA class I	HLA-A	41 kDa	5.15E+08	7.84E+08	5.16E+08	1.82E+08	4.40E+08
sp|P02679	Isoform Ga	FGG	49 kDa	1.38E+09	1.54E+09	9.63E+08	6.31E+08	7.96E+08
sp|Q8N3V7	Isoform 2 c	SYNPO	96 kDa	6.77E+08	9.86E+08	7.62E+08	3.44E+08	6.09E+08
sp|P07900	Isoform 2 c	HSP90AA1	98 kDa	8.14E+08	1.17E+09	8.60E+08	2.43E+08	5.26E+08
sp|Q13576	Ras GTPase	IQGAP2	181 kDa	2.28E+08	4.60E+08	3.05E+08	1.13E+08	2.55E+08
sp|P22314	Ubiquitin-li	UBA1	118 kDa	2.67E+08	4.13E+08	3.11E+08	1.67E+08	2.61E+08
sp|P06576	ATP syntha	ATP5B	57 kDa	4.78E+08	6.77E+08	4.31E+08	2.13E+08	4.22E+08
sp|P02461	Collagen al	COL3A1	139 kDa	2.17E+08	1.04E+09	4.58E+08	51400000	1.60E+08
sp|P08107	Heat shock	HSPA1A	70 kDa	7.31E+08	1.12E+09	8.34E+08	2.93E+08	6.08E+08

sp|P15924	Desmoplak	DSP	332 kDa	2751400	11100000	0	1579500	0
sp|P11021	78 kDa gluc	HSPA5	72 kDa	3.97E+08	6.06E+08	3.99E+08	2.09E+08	3.96E+08
sp|P54652	Heat shock	HSPA2	70 kDa	6.88E+08	1.03E+09	6.71E+08	2.54E+08	6.24E+08
sp|Q96JY6-	Isoform 5 c	PDLIM2	63 kDa	9.85E+08	1.47E+09	1.09E+09	4.27E+08	7.99E+08
sp|P07195	L-lactate de	LDHB	37 kDa	3.44E+08	5.20E+08	3.90E+08	2.34E+08	3.88E+08
sp|P55072	Transitiona	VCP	89 kDa	1.41E+08	2.12E+08	1.62E+08	75900000	1.24E+08
sp|P00558	Phosphogly	PGK1	45 kDa	1.98E+08	2.81E+08	1.46E+08	1.14E+08	2.17E+08
sp|ANXA5_	sp|ANXA5_	ANXA5	36 kDa	3.30E+08	4.95E+08	3.85E+08	1.57E+08	3.07E+08
sp|P05556	Integrin be	ITGB1	88 kDa	4.44E+08	6.03E+08	5.40E+08	1.66E+08	3.30E+08
sp|Q9GZM	Tubulointe	TINAGL1	52 kDa	2.48E+08	3.30E+08	2.91E+08	1.13E+08	1.75E+08
sp|P02452	Collagen al	COL1A1	139 kDa	40300000	3.08E+08	91200000	7580100	9.20E+07
sp|P26038	Moesin	MSN	68 kDa	4.11E+08	6.30E+08	4.18E+08	2.08E+08	3.66E+08
sp|P24844	Myosin reg	MYL9	20 kDa	2.92E+08	4.63E+08	2.45E+08	1.27E+08	2.00E+08
sp|P06733	Alpha-enol	ENO1	47 kDa	5.60E+07	54900000	73300000	38200000	53300000
sp|P62805	Histone H4	HIST1H4A	11 kDa	1.31E+09	2.19E+09	1.73E+09	7.92E+08	1.91E+09
sp|P21980	Protein-glu	TGM2	77 kDa	2.65E+08	3.47E+08	2.51E+08	1.17E+08	2.12E+08
sp|P08133	Annexin A6	ANXA6	76 kDa	1.28E+08	1.55E+08	1.55E+08	59100000	1.27E+08
sp|P30101	Protein disu	PDIA3	57 kDa	3.49E+08	5.62E+08	3.91E+08	1.47E+08	3.21E+08
sp|Q14204	Cytoplasmi	DYNC1H1	532 kDa	48800000	93300000	72100000	28800000	80300000
sp|P14625	Endoplasm	HSP90B1	92 kDa	3.26E+08	4.47E+08	3.43E+08	1.27E+08	2.61E+08
sp|P04792	Heat shock	HSPB1	23 kDa	3.06E+08	5.11E+08	4.22E+08	1.74E+08	3.11E+08
sp|P04083	Annexin A1	ANXA1	39 kDa	2.62E+08	3.90E+08	3.23E+08	1.17E+08	2.33E+08
sp|P25705	ATP syntha	ATP5A1	60 kDa	3.32E+08	4.65E+08	3.27E+08	1.57E+08	3.01E+08
sp|P62158	Calmodulin	CALM1	17 kDa	2.30E+08	2.68E+08	2.83E+08	94800000	1.31E+08
sp|Q96HC4	PDZ and LII	PDLIM5	64 kDa	2.48E+08	4.42E+08	3.29E+08	91200000	2.38E+08
sp|Q02413	Desmoglein	DSG1	114 kDa	16700000	0	0	3636200	0
sp|P0C0S8	Histone H2	HIST1H2AG	14 kDa	9.89E+08	1.34E+09	8.72E+08	5.48E+08	9.88E+08
sp|P05062	Fructose-bi	ALDOB	39 kDa	5.66E+08	4.41E+08	4.93E+08	2.17E+08	2.60E+08
sp|Q16555	Dihydropyr	DPYSL2	62 kDa	2.27E+08	3.72E+08	2.35E+08	1.09E+08	2.19E+08
sp|P37802-	Isoform 2 c	TAGLN2	24 kDa	1.93E+08	3.27E+08	2.56E+08	1.01E+08	2.04E+08
sp|Q86YZ3	Hornerin	HRNR	282 kDa	4127100	0	0	0	0
sp|P10809	60 kDa hea	HSPD1	61 kDa	1.72E+08	2.08E+08	1.80E+08	79100000	1.29E+08
sp|Q96A08	Histone H2	HIST1H2BA	14 kDa	3.16E+08	6.77E+08	5.42E+08	1.95E+08	1.93E+08
sp|P22626	Heterogene	HNRNPA2B	37 kDa	1.93E+08	3.26E+08	2.65E+08	95200000	1.61E+08
sp|P17655	Calpain-2 c	CAPN2	80 kDa	50500000	1.83E+08	1.47E+08	36200000	82100000
sp|P50895	Basal cell a	BCAM	67 kDa	1.84E+08	3.13E+08	2.30E+08	85700000	1.20E+08
sp|P01024	Compleme	C3	187 kDa	1.16E+08	2.43E+08	1.29E+08	36300000	6.40E+07
sp|P50440-	Isoform 2 c	GATM	45 kDa	3.20E+08	2.83E+08	3.48E+08	1.45E+08	1.44E+08
sp|P63104	14-3-3 prot	YWHAZ	28 kDa	2.57E+08	3.46E+08	4.46E+08	1.50E+08	2.98E+08
sp|P61106	Ras-related	RAB14	24 kDa	83500000	1.20E+08	1.07E+08	48900000	99400000
sp|Q07075	Glutamyl a	ENPEP	109 kDa	1.00E+08	1.46E+08	91700000	32700000	9.60E+07
sp|P13639	Elongation	EEF2	95 kDa	7.80E+07	97600000	73500000	41100000	7.80E+07
sp|P62258	14-3-3 prot	YWHAE	29 kDa	80400000	1.15E+08	1.95E+08	8.70E+07	1.89E+08
sp|Q96KP4	Cytosolic n	CNDP2	53 kDa	1.06E+08	1.58E+08	59900000	69800000	84900000
sp|P04004	Vitronectin	VTN	54 kDa	2.11E+08	3.50E+08	2.78E+08	92500000	1.77E+08
sp|P07339	Cathepsin I	CTSD	45 kDa	2.60E+07	40300000	32100000	17700000	28200000
tr|E7EVA0|	Microtubul	MAP4	245 kDa	39900000	1.17E+08	63400000	23100000	45100000

sp|Q01995	Transgelin	TAGLN	23 kDa	1.55E+08	2.61E+08	2.18E+08	6.90E+07	1.80E+08
sp|P00966	Argininosu	ASS1	47 kDa	1.63E+08	1.54E+08	1.54E+08	85500000	96900000
sp|P30041	Peroxiredo	PRDX6	25 kDa	1.84E+08	2.21E+08	1.63E+08	74900000	1.62E+08
sp|P07237	Protein dis	P4HB	57 kDa	8.60E+07	1.68E+08	1.25E+08	54300000	1.03E+08
sp|P27797	Calreticulin	CALR	48 kDa	3.10E+08	4.80E+08	3.13E+08	99100000	2.02E+08
sp|P61978	Isoform 2 c	HNRNPK	51 kDa	1.08E+08	1.64E+08	1.38E+08	57300000	1.19E+08
sp|P13489	Ribonuclea	RNH1	50 kDa	70200000	91300000	83200000	28200000	44800000
sp|P62937	Peptidyl-pr	PPIA	18 kDa	9.00E+07	1.01E+08	52200000	4.80E+07	81900000
sp|P46940	Ras GTPase	IQGAP1	189 kDa	82900000	1.07E+08	95700000	4.30E+07	69400000
sp|P08123	Collagen al	COL1A2	129 kDa	2.80E+07	5.06E+08	68400000	22600000	1.83E+08
sp|P62979	Ubiquitin-4	RPS27A	18 kDa	3.34E+08	5.20E+08	4.84E+08	1.72E+08	3.40E+08
sp|P0C0L4	Isoform 2 c	C4A	188 kDa	1.41E+08	1.61E+08	1.46E+08	57500000	99100000
sp|GSTP1_	sp|GSTP1_	GSTP1	23 kDa	1.29E+08	1.70E+08	71500000	63500000	90100000
sp|P24821	Isoform 4 c	TNC	231 kDa	55900000	73400000	34200000	24900000	3.50E+07
sp|P60174	Isoform 2 c	TPI1	27 kDa	1.20E+07	2.00E+07	6496500	10200000	6826600
sp|O00592	Isoform 2 c	PODXL	55 kDa	3.73E+08	5.82E+08	4.14E+08	1.14E+08	2.19E+08
sp|P06702	Protein S1(	S100A9	13 kDa	15300000	14100000	16700000	476620	5133200
sp|P02792	Ferritin ligh	FTL	20 kDa	67800000	1.07E+08	59200000	41400000	59500000
sp|Q15019	Isoform 2 c	2-Sep	45 kDa	63700000	1.26E+08	76400000	35100000	73100000
sp|P30086	Phosphatid	PEBP1	21 kDa	20600000	38400000	46100000	5554200	7728200
sp|Q5D862	Filaggrin-2	FLG2	248 kDa	8953300	0	0	4567000	0
sp|Q08554	Isoform 1B	DSC1	94 kDa	0	0	0	0	0
sp|Q63HR2	Isoform 4 c	TENC1	154 kDa	1.18E+08	1.54E+08	1.14E+08	29300000	81300000
sp|Q9NZA1	Isoform 1 c	CLIC5	28 kDa	1.64E+08	2.80E+08	1.81E+08	95500000	1.27E+08
sp|O00299	Chloride in	CLIC1	27 kDa	1.25E+08	1.95E+08	1.47E+08	8.90E+07	1.44E+08
sp|Q8WX9	Palladin	PALLD	151 kDa	77200000	1.21E+08	1.01E+08	26800000	63900000
sp|P21291	Cysteine ar	CSRP1	21 kDa	2.90E+08	4.03E+08	3.13E+08	1.11E+08	2.48E+08
sp|P07737	Profilin-1	PFN1	15 kDa	1.99E+08	4.26E+08	3.25E+08	1.27E+08	1.90E+08
sp|Q15365	Poly(rC)-bi	PCBP1	37 kDa	78700000	1.15E+08	94800000	26800000	63400000
sp|O15231	Isoform 3 c	ZNF185	74 kDa	66800000	1.06E+08	1.01E+08	21500000	40100000
sp|Q6UXI9	Isoform 6 c	NPNT	64 kDa	99400000	2.41E+08	1.72E+08	55400000	1.53E+08
sp|Q13418	Integrin-lin	ILK	51 kDa	58500000	1.16E+08	98600000	38400000	86100000
sp|O75083	WD repeat	WDR1	66 kDa	53400000	1.11E+08	59100000	32900000	74200000
sp|P56199	Integrin alp	ITGA1	131 kDa	40500000	67900000	25500000	24200000	56900000
sp|P61158	Actin-relat	ACTR3	47 kDa	81400000	2.21E+08	1.19E+08	35800000	85700000
sp|P23381	Tryptophar	WARS	53 kDa	64800000	1.14E+08	91600000	23200000	72100000
sp|P29401	Isoform 2 c	TKT	69 kDa	66800000	54200000	81600000	21700000	58900000
sp|Q15582	Transformi	TGFBI	75 kDa	44600000	76500000	49800000	24200000	51500000
sp|P27824	Isoform 2 c	CANX	72 kDa	1.15E+08	1.92E+08	1.53E+08	38100000	8.60E+07
sp|HBA_HU	sp|HBA_HU	HBA	15 kDa	1.65E+08	2.70E+08	1.83E+08	93800000	1.78E+08
sp|P02748	Compleme	C9	63 kDa	1.12E+08	1.81E+08	1.38E+08	25200000	46500000
sp|P02788	Lactotransf	LTF	78 kDa	0	0	0	0	0
sp|P14923	Junction pl	JUP	82 kDa	3920200	0	0	1339300	0
sp|P26006	Integrin alp	ITGA3	117 kDa	59600000	82700000	48600000	20100000	5.30E+07
sp|P38646	Stress-70 p	HSPA9	74 kDa	70800000	83800000	67300000	2.30E+07	27200000
sp|Q14764	Major vault	MVP	99 kDa	36700000	23900000	41500000	19300000	4.90E+07
tr|Q5JW85	Glutathion	GSTA3	20 kDa	85500000	1.02E+08	1.06E+08	65900000	47800000

sp|P05091	Aldehyde d	ALDH2	56 kDa	96600000	66700000	76700000	5.70E+07	59300000
sp|P04275	von Willebı	VWF	309 kDa	22300000	30800000	42500000	4325600	12900000
sp|P21399	Cytoplasmi	ACO1	98 kDa	26900000	23600000	23600000	14200000	9930200
sp|Q03154	Aminoacyla	ACY1	46 kDa	2.30E+07	24700000	3643400	15700000	13600000
sp|P02760	Protein AM	AMBP	39 kDa	68100000	84700000	1.02E+08	26300000	1.01E+08
sp|P40939	Trifunction	HADHA	83 kDa	17100000	62500000	32700000	10900000	39400000
sp|P19338	Nucleolin	NCL	77 kDa	63700000	1.24E+08	68400000	24600000	7.20E+07
sp|P13797	Plastin-3	PLS3	71 kDa	67200000	1.81E+08	1.17E+08	50800000	1.14E+08
sp|Q16822	Phosphoen	PCK2	71 kDa	1.17E+08	77200000	8.50E+07	57100000	43500000
sp|Q93088	Betaine--hc	BHMT	45 kDa	1.54E+08	1.36E+08	82400000	43300000	74900000
sp|P16402	Histone H1	HIST1H1D	22 kDa	1.21E+08	88200000	64900000	5.50E+07	70800000
sp|P14550	Alcohol deł	AKR1A1	37 kDa	1.32E+08	1.23E+08	84200000	74100000	54600000
sp|P01876	Ig alpha-1 c	IGHA1	38 kDa	24100000	11800000	2.40E+07	11900000	37600000
sp|Q06830	Peroxiredo	PRDX1	22 kDa	1.14E+08	1.19E+08	70900000	5.20E+07	67400000
sp|P26599	Polypyrimid	PTBP1	57 kDa	34100000	43800000	36400000	25100000	31100000
sp|Q16658	Fascin	FSCN1	55 kDa	3.80E+07	61600000	54500000	2.20E+07	5.10E+07
sp|P00367	Glutamate	GLUD1	61 kDa	1.30E+08	1.32E+08	1.59E+08	55900000	81900000
sp|P30038	Delta-1-pyr	ALDH4A1	62 kDa	1.85E+08	1.43E+08	1.68E+08	9.50E+07	3.60E+07
sp|Q16181	Isoform 2 c	7-Sep	51 kDa	67500000	94500000	60900000	29700000	8.70E+07
sp|P17927	Compleme	CR1	224 kDa	37100000	51200000	39500000	14100000	39800000
sp|P23246	Splicing fac	SFPQ	76 kDa	30500000	49700000	35900000	11900000	33100000
sp|P35241	Radixin	RDX	69 kDa	1.94E+08	3.07E+08	2.05E+08	98400000	1.72E+08
sp|P24752	Acetyl-CoA	ACAT1	45 kDa	86900000	75900000	95200000	43900000	47800000
sp|Q01518	Adenylyl cy	CAP1	52 kDa	54600000	1.02E+08	35900000	33300000	71600000
sp|Q02388	Collagen al	COL7A1	295 kDa	0	0	0	0	0
sp|Q8N1N4	Keratin, typ	KRT78	57 kDa	2120600	0	0	0	0
sp|P62879	Guanine nu	GNB2	37 kDa	18200000	44800000	38700000	7721100	26500000
sp|P42224	Signal trans	STAT1	87 kDa	30100000	80200000	51100000	11600000	3.20E+07
sp|O43795	Isoform 2 c	MYO1B	125 kDa	39400000	5.00E+07	4.00E+07	21600000	32900000
sp|Q08AH3	Acyl-coenzy	ACSM2A	64 kDa	66100000	49300000	48300000	46500000	22900000
sp|P18669	Phosphogly	PGAM1	29 kDa	37500000	22800000	0	13200000	12700000
sp|P13760	HLA class II	HLA-DRB1	30 kDa	65600000	1.30E+08	72200000	25800000	7.30E+07
sp|Q6PCB0	von Willebı	VWA1	47 kDa	3.40E+07	27800000	39900000	12500000	26400000
sp|Q99497	Protein deg	PARK7	20 kDa	69400000	1.35E+08	17600000	12900000	24900000
sp|P02511	Alpha-cryst	CRYAB	20 kDa	24200000	4.30E+07	3.40E+07	27400000	34700000
sp|Q5IJ48|	Protein cru	CRB2	134 kDa	14800000	34400000	22100000	10100000	13700000
sp|O15144	Actin-relate	ARPC2	34 kDa	29600000	3.90E+07	33600000	13400000	35600000
sp|P30084	Enoyl-CoA	ECHS1	31 kDa	5.40E+07	42900000	28100000	32300000	27700000
sp|Q93052	Lipoma-pre	LPP	66 kDa	72800000	84200000	84600000	8212100	54300000
tr|J3KNQ4	Alpha-parv	PARVA	47 kDa	51600000	89500000	49100000	29400000	26400000
sp|Q9NQC	Reticulon-4	RTN4	130 kDa	1.01E+08	1.62E+08	63200000	47500000	6.40E+07
sp|O43301	Heat shock	HSPA12A	75 kDa	18300000	35300000	48300000	11700000	23800000
sp|Q9NVA2	Isoform 2 c	11-Sep	51 kDa	64800000	1.05E+08	72600000	24300000	18700000
tr|Q5TEC6	Histone clu	HIST2H3PS	15 kDa	2.12E+08	2.35E+08	2.00E+08	1.10E+08	1.56E+08
sp|P05109	Protein S10	S100A8	11 kDa	25400000	21600000	26600000	8628100	9894600
sp|P61586	Transformi	RHOA	22 kDa	53700000	75700000	39300000	25300000	34900000
sp|P42765	3-ketoacyl-	ACAA2	42 kDa	74400000	59300000	71500000	28400000	37900000

sp|Q00577	Transcriptio	PURA	35 kDa	35100000	56500000	48800000	22400000	41100000
sp|Q92597	Protein ND	NDRG1	43 kDa	55800000	95800000	45800000	31400000	45300000
sp|Q15417	Calponin-3	CNN3	36 kDa	51600000	1.01E+08	70800000	20100000	37700000
sp|Q5T749	Keratinocyt	KPRP	64 kDa	8564400	0	0	1789200	0
sp|P28838	Isoform 2 c	LAP3	53 kDa	5206200	12300000	8581500	11500000	18900000
sp|P02649	Apolipopro	APOE	36 kDa	33300000	48100000	44700000	6989500	36300000
sp|P46939	Isoform 2 c	UTRN	395 kDa	3747500	10800000	4542900	3128800	6484000
sp|Q14195	Isoform LCI	DPYSL3	74 kDa	68600000	1.10E+08	87600000	34800000	94600000
sp|P61160	Actin-relate	ACTR2	45 kDa	4.50E+07	67700000	55400000	17700000	34500000
sp|P12956	X-ray repai	XRCC6	70 kDa	35400000	49300000	33800000	7747800	25600000
sp|Q9BSJ8	Isoform 2 c	ESYT1	124 kDa	19300000	21500000	28700000	9701800	16700000
sp|P60953	Cell divisior	CDC42	21 kDa	45800000	68200000	41500000	20900000	28300000
sp|P31944	Caspase-14	CASP14	28 kDa	0	0	0	0	0
sp|P02743	Serum amy	APCS	25 kDa	63700000	1.33E+08	68800000	29300000	77300000
sp|P40925	Isoform 3 c	MDH1	39 kDa	1.35E+08	1.45E+08	1.10E+08	7.10E+07	96600000
sp|P78371	T-complex	CCT2	57 kDa	10200000	1.60E+07	19200000	1.00E+07	18300000
sp|P55795	Heterogen	HNRNPH2	49 kDa	22400000	41300000	23500000	3085900	8659400
sp|Q96J84	Kin of IRRE	KIRREL	84 kDa	13400000	39100000	33200000	11100000	12100000
sp|Q15942	Zyxin	ZYX	61 kDa	1.04E+08	1.77E+08	95100000	28700000	28600000
sp|Q9H223	EH domain	EHD4	61 kDa	6.20E+07	1.05E+08	81700000	27100000	8.60E+07
sp|O14745	Na(+)/H(+)	SLC9A3R1	39 kDa	5.70E+07	88100000	64400000	21300000	38600000
sp|P61981	14-3-3 prot	YWHAG	28 kDa	1.04E+08	1.62E+08	1.97E+08	78400000	1.57E+08
sp|Q7Z794	Keratin, typ	KRT77	62 kDa	5.45E+08	1.78E+08	2.28E+08	2.73E+08	1.14E+08
sp|P09382	Galectin-1	LGALS1	15 kDa	8812700	0	0	3314300	0
tr|F8W6I7|	Heterogen	HNRNPA1	33 kDa	42300000	74600000	55900000	15700000	37800000
sp|O00151	PDZ and LII	PDLIM1	36 kDa	30200000	59600000	3.70E+07	15700000	26200000
sp|P01009	Alpha-1-an	SERPINA1	47 kDa	1.10E+07	12200000	19700000	1.00E+07	22900000
sp|P12277	Creatine ki	CKB	43 kDa	31800000	60400000	37300000	20800000	37700000
sp|Q07065	Cytoskelet	CKAP4	66 kDa	1.70E+07	25200000	11500000	2642100	12300000
sp|Q8NFW	Isoform 2 c	COL22A1	159 kDa	0	0	0	0	0
sp|Q9Y6C2	EMILIN-1	EMILIN1	107 kDa	22700000	28700000	19100000	6213700	17500000
sp|P27105	Erythrocyte	STOM	32 kDa	21600000	4.10E+07	16400000	17700000	39200000
sp|Q8WXI7	Mucin-16	MUC16	2353 kDa	0	0	0	0	0
sp|E9PAV3	Nascent po	NACA	205 kDa	0	7671800	3344000	0	0
sp|Q14847	LIM and SH	LASP1	30 kDa	45700000	58200000	49700000	22800000	32400000
sp|P10301	Ras-related	RRAS	23 kDa	25900000	87200000	68200000	9258100	42400000
sp|P52209	Isoform 2 c	PGD	52 kDa	4.10E+07	60700000	23600000	15500000	4.60E+07
sp|P61626	Lysozyme C	LYZ	17 kDa	13400000	5423600	0	9167300	6007000
sp|P06744	Glucose-6-p	GPI	63 kDa	27800000	30600000	2.60E+07	10700000	16400000
sp|Q01469	Fatty acid-b	FABP5	15 kDa	17700000	3464500	14600000	0	0
sp|P27348	14-3-3 prot	YWHAQ	28 kDa	44100000	72900000	1.31E+08	50300000	1.07E+08
sp|P51148	Isoform 2 c	RAB5C	27 kDa	33600000	64800000	36700000	16700000	30900000
sp|P07910	Isoform C1	HNRNPC	32 kDa	13600000	30600000	45700000	17600000	4.20E+07
sp|Q15404	Ras suppre	RSU1	32 kDa	48200000	77800000	52700000	24500000	41500000
sp|P25940	Collagen al	COL5A3	172 kDa	0	0	0	0	0
sp|Q9NP85	Podocin	NPHS2	42 kDa	13400000	7.00E+07	43200000	41800000	81500000
sp|Q9NYL9	Tropomodu	TMOD3	40 kDa	28600000	24300000	17600000	8632300	11200000

sp|Q13492	Isoform 2 c	PICALM	69 kDa	9224800	21300000	13500000	6282300	10900000
sp|P09525	Annexin A4	ANXA4	36 kDa	20600000	31500000	23400000	8416500	17800000
sp|P48735	Isocitrate d	IDH2	51 kDa	52800000	45500000	52400000	25900000	33900000
sp|O60500	Isoform 2 c	NPHS1	131 kDa	13700000	15400000	10300000	0	15900000
sp|P32119	Peroxiredo	PRDX2	22 kDa	50300000	48500000	35300000	18600000	22100000
sp|Q99832	T-complex	CCT7	59 kDa	5474400	869020	9637900	1474200	9482300
sp|Q3LXA3	Bifunctiona	DAK	59 kDa	34800000	20700000	28300000	2.10E+07	22700000
sp|P25311	Zinc-alpha-	AZGP1	34 kDa	0	0	0	0	0
sp|P05937	Calbindin	CALB1	30 kDa	79700000	36600000	5.60E+07	2.40E+07	29400000
sp|P0CG39	POTE ankyr	POTEJ	117 kDa	6.01E+09	7.51E+09	6.71E+09	2.92E+09	5.84E+09
sp|P61163	Alpha-cent	ACTR1A	43 kDa	30400000	42800000	21100000	8688000	24400000
sp|P17987	T-complex	TCP1	60 kDa	7180100	14900000	8681100	2096900	5494400
sp|P46821	Microtubul	MAP1B	271 kDa	9492500	22800000	19500000	6381600	1.80E+07
sp|P05362	Intercellula	ICAM1	58 kDa	8330200	7293800	9223400	15200000	10300000
sp|P35237	Serpin B6	SERPINB6	43 kDa	28100000	44900000	22800000	11700000	50500000
sp|O43294	Isoform 2 c	TGFB1I1	48 kDa	23200000	31300000	27700000	10700000	21600000
sp|P45880	Isoform 2 c	VDAC2	30 kDa	96700000	1.58E+08	1.27E+08	31200000	88400000
sp|P04843	Dolichyl-di	RPN1	69 kDa	20700000	34300000	2.80E+07	10700000	1.30E+07
sp|P31942	Isoform 3 c	HNRNPH3	32 kDa	17900000	20900000	18700000	7762700	7572900
sp|Q96QK1	Vacuolar pr	VPS35	92 kDa	14900000	24100000	16300000	7866300	13800000
sp|P26641	Isoform 2 c	EEF1G	56 kDa	25600000	41400000	21200000	4363000	24600000
sp|O00571	Isoform 2 c	DDX3X	71 kDa	5209800	14600000	6514900	4330500	13500000
sp|P63010	Isoform 2 c	AP2B1	106 kDa	6216600	5539100	9275800	5305100	11300000
sp|Q02952	A-kinase ar	AKAP12	191 kDa	22800000	2.80E+07	15100000	4480900	12200000
sp|P21281	V-type prot	ATP6V1B2	57 kDa	17200000	19100000	23200000	6424100	14400000
sp|P52943	Isoform 2 c	CRIP2	30 kDa	1.93E+08	2.48E+08	42100000	76800000	1.17E+08
sp|Q14697	Neutral alp	GANAB	107 kDa	22100000	28900000	17300000	8210700	20200000
sp|P55327	Isoform 3 c	TPD52	26 kDa	20500000	3.20E+07	19500000	10700000	11600000
sp|P13796	Plastin-2	LCP1	70 kDa	14600000	47200000	40800000	21600000	45200000
sp|P54727	UV excisior	RAD23B	43 kDa	19500000	33700000	21500000	7203800	2199200
sp|Q14247	Src substra	CTTN	62 kDa	33100000	54300000	25500000	14500000	31700000
sp|P52758	Ribonuclea	HRSP12	14 kDa	66600000	55500000	2.90E+07	30300000	34100000
sp|P49773	Histidine tr	HINT1	14 kDa	24500000	31300000	0	3105300	19200000
sp|Q9UHG	Prenylcyste	PCYOX1	57 kDa	1.10E+07	17200000	11500000	755130	3971800
sp|Q9Y696	Chloride in	CLIC4	29 kDa	25500000	33600000	50100000	11900000	18700000
sp|Q02878	60S ribosor	RPL6	33 kDa	23600000	39700000	28100000	4398700	6309100
sp|P05164	Isoform H1	MPO	74 kDa	0	1473900	0	0	0
sp|Q6NVY1	3-hydroxyis	HIBCH	43 kDa	28800000	34200000	19400000	20300000	2.50E+07
sp|P33176	Kinesin-1 h	KIF5B	110 kDa	20100000	35900000	34200000	9250900	14400000
sp|P49189	4-trimethyl	ALDH9A1	54 kDa	26400000	33400000	15600000	15700000	29100000
sp|Q15691	Microtubul	MAPRE1	30 kDa	32600000	49600000	25500000	9243500	5480000
sp|P49411	Elongation	TUFM	50 kDa	38500000	51900000	32400000	28100000	34100000
sp|P17174	Aspartate a	GOT1	46 kDa	2375400	8842900	2286000	6861000	6291900
sp|Q02252	Isoform 2 c	ALDH6A1	56 kDa	67700000	62400000	32600000	39800000	15700000
sp|Q9UBQ	Glyoxylate	GRHPR	36 kDa	30600000	49300000	25400000	16400000	25300000
sp|P10768	S-formylglu	ESD	31 kDa	11500000	37300000	5944100	8179300	11900000
sp|P40227	T-complex	CCT6A	58 kDa	17900000	28800000	21100000	9071500	21100000

sp|P18124	60S ribosor	RPL7	29 kDa	29500000	42700000	32700000	13800000	34400000
sp|P47755	F-actin-cap	CAPZA2	33 kDa	83800000	1.42E+08	1.07E+08	32500000	54800000
sp|P01903	HLA class II	HLA-DRA	29 kDa	29300000	2.80E+07	3.90E+07	9730600	12600000
sp|P13010	X-ray repai	XRCC5	83 kDa	18800000	52700000	38300000	8456100	4810500
sp|P30153	Serine/thre	PPP2R1A	65 kDa	25100000	39300000	32500000	13700000	29600000
sp|Q08188	Protein-glu	TGM3	77 kDa	0	0	0	0	0
sp|P40926	Malate deh	MDH2	36 kDa	16100000	16400000	8255200	15200000	1.70E+07
sp|Q8WWI	Isoform 2 c	LMO7	191 kDa	21300000	4.90E+07	19200000	5694100	16100000
sp|P21796	Voltage-de	VDAC1	31 kDa	28600000	7.90E+07	30600000	11700000	48500000
sp|P53708	Integrin alp	ITGA8	117 kDa	12200000	42200000	20700000	8103000	10100000
sp|P81605	Dermcidin	DCD	11 kDa	6313000	3498100	7906100	0	0
sp|P05106	Integrin be	ITGB3	87 kDa	21500000	22500000	13600000	1516800	9539000
sp|Q15517	Corneodesı	CDSN	52 kDa	0	0	0	0	0
sp|P14866	Heterogen	HNRNPL	64 kDa	21400000	40800000	43600000	8781900	20600000
sp|Q7KZF4	Staphyloco	SND1	102 kDa	13200000	15400000	9642200	5450500	7168800
sp|Q96I99	Succinyl-Cc	SUCLG2	47 kDa	45600000	22100000	29600000	19600000	18700000
sp|Q86UP2	Isoform 2 c	KTN1	150 kDa	1770800	471750	6028100	2848600	1073400
sp|P05388	60S acidic r	RPLP0	34 kDa	16200000	25600000	17800000	17200000	13400000
sp|P30740	Leukocyte	SERPINB1	43 kDa	21800000	31600000	12500000	2950200	17300000
sp|Q9NVM	Protein eva	EVA1B	18 kDa	13100000	10500000	12300000	6317000	3951700
sp|Q9Y6N5	Sulfide:quii	SQRDL	50 kDa	30500000	88100000	53100000	20800000	40600000
sp|P38606	Isoform 2 c	ATP6V1A	65 kDa	4326800	8043500	670180	5285800	7388100
sp|P60981	Destrin	DSTN	19 kDa	34100000	74800000	38800000	11800000	35500000
sp|P62424	60S ribosor	RPL7A	30 kDa	7419300	1.20E+07	2.10E+07	5303000	15700000
sp|Q08211	ATP-depen	DHX9	141 kDa	2203500	9530700	3894800	1802100	5009200
sp|P50991	T-complex	CCT4	58 kDa	11600000	40300000	14200000	1.10E+07	1.80E+07
sp|P04179	Isoform 4 c	SOD2	20 kDa	61800000	55600000	66700000	33400000	39800000
sp|P35232	Prohibitin	PHB	30 kDa	7931600	45600000	29700000	10500000	2.90E+07
sp|P61088	Ubiquitin-c	UBE2N	17 kDa	28900000	35100000	30400000	8153700	18600000
sp|P08311	Cathepsin (	CTSG	29 kDa	0	0	5054900	0	0
sp|P14174	Macrophag	MIF	12 kDa	2280800	2391100	5461600	3497000	4245000
sp|P29972	Isoform 2 c	AQP1	20 kDa	1.02E+08	1.90E+08	1.30E+08	4.70E+07	84100000
sp|Q15836	Vesicle-ass	VAMP3	11 kDa	36100000	54100000	39900000	10800000	23500000
sp|Q9UHD	Isoform 2 c	9-Sep	64 kDa	44600000	37600000	3.50E+07	11800000	18300000
sp|P35221	Catenin alp	CTNNA1	100 kDa	3444000	14200000	9712100	1916600	6080400
sp|P59998	Isoform 3 c	ARPC4	22 kDa	15900000	15900000	18300000	15400000	41900000
sp|P05089	Arginase-1	ARG1	35 kDa	0	0	0	0	0
sp|Q9Y4L1	Hypoxia up	HYOU1	111 kDa	547830	4597400	4350000	5567600	6568700
sp|P52907	F-actin-cap	CAPZA1	33 kDa	48900000	64800000	76100000	2.70E+07	51700000
sp|P35556	Fibrillin-2	FBN2	315 kDa	42400000	60800000	4.10E+07	23500000	34900000
sp|Q16827	Isoform 2 c	PTPRO	135 kDa	10600000	14600000	21500000	8316000	8166300
sp|P51149	Ras-related	RAB7A	23 kDa	7590000	11600000	10500000	5531400	11700000
sp|O76041	Isoform 2 c	NEBL	31 kDa	50600000	63700000	5.70E+07	14900000	24900000
sp|Q8IVN8	Somatome	SBSPON	30 kDa	6881000	4587000	0	2973800	0
sp|Q9Y4B5	Microtubul	MTCL1	210 kDa	77200000	0	0	0	0
sp|Q7Z6Z7	Isoform 2 c	HUWE1	480 kDa	1093100	3136100	1170000	1246600	1908300
sp|P00505	Aspartate a	GOT2	48 kDa	12400000	38600000	22800000	8475000	3.00E+07

sp|P18428	Lipopolysa	LBP	53 kDa	13100000	17300000	9986700	1007300	991490
sp|P19367	Isoform 2 c	HK1	102 kDa	12100000	6687300	10500000	4270300	8289200
sp|P50454	Serpin H1	SERPINH1	46 kDa	15600000	37500000	29500000	5027200	22100000
sp|Q08257	Isoform 3 c	CRYZ	32 kDa	20800000	27400000	23100000	16900000	21100000
sp|P40763	Isoform De	STAT3	88 kDa	9672100	14600000	12300000	4730900	6507200
sp|O15143	Actin-relat	ARPC1B	41 kDa	2.30E+07	14900000	26600000	8147700	24700000
sp|O15145	Actin-relat	ARPC3	21 kDa	21400000	30100000	26900000	9817200	20500000
sp|O95782	Isoform B c	AP2A1	105 kDa	5302800	9199800	5374900	3455800	11200000
sp|P20930	Filaggrin	FLG	435 kDa	0	0	0	0	0
sp|P00352	Retinal deh	ALDH1A1	55 kDa	9206300	16400000	7233400	19900000	15400000
sp|P00491	Purine nucl	PNP	32 kDa	1786500	5830900	5746500	5366600	3080500
sp|P05026	Isoform 2 c	ATP1B1	35 kDa	27100000	50700000	31700000	6486300	31300000
sp|O75947	Isoform 2 c	ATP5H	16 kDa	5658800	22800000	10200000	1526200	1631900
sp|Q9BR76	Coronin-1B	CORO1B	54 kDa	8014000	14900000	6390800	0	3366500
sp|P61604	10 kDa hea	HSPE1	11 kDa	29100000	18100000	15600000	11200000	17200000
sp|P50995	Isoform 2 c	ANXA11	51 kDa	11200000	9676400	4369100	4209700	0
sp|P50552	Vasodilator	VASP	40 kDa	5538000	8872600	4598800	0	7591000
sp|O14773	Isoform 2 c	TPP1	34 kDa	6754300	4846700	11200000	1812500	3517800
sp|P37837	Transaldola	TALDO1	38 kDa	16500000	25100000	24200000	8323100	26700000
sp|Q6UWP	Suprabasin	SBSN	61 kDa	0	0	0	0	0
sp|Q9NZT1	Calmodulin	CALML5	16 kDa	0	0	0	0	0
sp|Q99714	3-hydroxya	HSD17B10	27 kDa	9333600	13400000	7367900	6250300	1.10E+07
sp|P62140	Serine/thre	PPP1CB	37 kDa	1146400	2054700	3263100	465310	2930800
sp|P16284	Isoform De	PECAM1	80 kDa	6300700	12200000	4771200	2268500	5953400
sp|P20073	Isoform 2 c	ANXA7	50 kDa	9940500	0	14600000	0	2818600
sp|Q16836	Isoform 2 c	HADH	42 kDa	6514300	5239200	14800000	3525300	6108500
sp|P32455	Interferon-	GBP1	68 kDa	5150900	16600000	11900000	1661400	14200000
sp|P31151	Protein S1(	S100A7	11 kDa	0	0	0	0	0
sp|P25787	Proteasom	PSMA2	26 kDa	8455700	10600000	6804700	3613800	6578400
sp|Q14118	Dystroglyca	DAG1	97 kDa	1253700	10300000	11200000	2203000	5793400
sp|Q9HC84	Mucin-5B	MUC5B	596 kDa	0	0	0	0	0
sp|Q14203	Isoform 3 c	DCTN1	137 kDa	1616000	706840	1474100	1801400	0
sp|P48047	ATP syntha	ATP5O	23 kDa	8810700	17600000	1518200	781290	1308300
sp|Q99536	Synaptic ve	VAT1	42 kDa	0	0	5906900	0	888680
tr|K7ELL7|	Glucosidas	PRKCSH	60 kDa	0	0	0	0	0
sp|P34932	Heat shock	HSPA4	94 kDa	4216200	7672000	0	394550	1989900
sp|P04844	Isoform 2 c	RPN2	68 kDa	348840	12700000	9353300	3776100	8792200
sp|P0DJI8|	Serum amy	SAA1	14 kDa	29100000	39900000	43400000	8383300	18200000
sp|P31949	Protein S1(	S100A11	12 kDa	0	245640	269080	305250	328450
sp|Q04446	1,4-alpha-g	GBE1	80 kDa	9246300	12900000	9891500	3461600	5407100
sp|Q14974	Importin su	KPNB1	97 kDa	0	6795500	16500000	6410300	1.10E+07
sp|Q9BQI9	Isoform 2 c	NRIP2	30 kDa	0	2621200	5438800	522160	708100
sp|Q9HCM	Isoform 3 c	EPB41L5	82 kDa	973830	1435800	2615200	3733000	9325500
sp|P05387	60S acidic r	RPLP2	12 kDa	29200000	47100000	36600000	2.10E+07	38500000
sp|P60033	CD81 antig	CD81	26 kDa	39400000	58400000	44100000	10600000	23400000
sp|Q06323	Proteasom	PSME1	29 kDa	0	1017300	1013300	874330	1021300
sp|O60763	General ve	USO1	108 kDa	8920800	6457400	7913300	0	3988600

sp|P16152	Carbonyl re	CBR1	30 kDa	9459900	7696300	1970500	3149600	6534500
sp|P50990	T-complex	CCT8	60 kDa	13500000	9486700	14200000	4738200	15600000
sp|P62241	40S ribosor	RPS8	24 kDa	2.30E+07	25300000	10700000	9575000	21900000
sp|Q96TA1	Isoform 2 c	FAM129B	83 kDa	6536100	12200000	9070900	1627300	5157800
sp|Q9BW3	Tubulin pol	TPPP3	19 kDa	2.20E+07	40300000	14600000	9759800	26200000
sp|Q96JE9	Microtubul	MAP6	87 kDa	0	0	0	0	0
sp|Q13011	Delta(3,5)-I	ECH1	36 kDa	1.70E+07	17800000	16300000	8036000	8613200
sp|O75874	Isocitrate d	IDH1	47 kDa	9723700	1847400	3001300	4639600	1.00E+07
sp|Q8IWA!	Isoform 2 c	SLC44A2	81 kDa	1.10E+07	18300000	13600000	3125300	11700000
sp|Q9UH9!	Isoform 3 c	SUN2	80 kDa	1601500	1776400	1603200	1236800	1294400
sp|P05386	60S acidic r	RPLP1	12 kDa	2.20E+07	30300000	22500000	1.80E+07	12200000
sp|P11279	Lysosome-a	LAMP1	45 kDa	0	0	0	1570100	1394200
sp|P00403	Cytochrom	MT-CO2	26 kDa	8283700	26700000	5370600	3498700	9198600
sp|P08246	Neutrophil	ELANE	29 kDa	0	0	0	0	0
sp|P17858	Isoform 2 c	PFKL	90 kDa	0	0	0	0	0
sp|P21695	Isoform 2 c	GPD1	35 kDa	4.80E+07	17100000	14500000	34300000	2.50E+07
sp|P61313	60S ribosor	RPL15	24 kDa	0	2036100	987760	6849500	4946400
sp|Q9C0C2	182 kDa tai	TNKS1BP1	182 kDa	4403600	16600000	1.20E+07	3150800	8871200
sp|Q96IU4	Alpha/beta	ABHD14B	22 kDa	16700000	21700000	16600000	3791200	9402400
sp|Q9UGM	Isoform 8 c	DMBT1	181 kDa	0	0	0	0	0
sp|P10599	Thioredoxii	TXN	12 kDa	0	0	0	0	0
sp|P35558	Phosphoen	PCK1	69 kDa	19600000	7085600	7408300	12200000	11600000
sp|Q06418	Tyrosine-pr	TYRO3	97 kDa	557600	9386300	0	0	500290
sp|P43490	Nicotinami	NAMPT	56 kDa	18700000	24800000	1.40E+07	5210900	1844800
sp|O75396	Vesicle-traf	SEC22B	25 kDa	4035200	5875500	5332700	2335800	4408700
sp|P05455	Lupus La pr	SSB	47 kDa	17300000	6374800	11300000	4849300	1.20E+07
sp|P58166	Inhibin bet	INHBE	39 kDa	0	22700000	7506800	4756800	15100000
sp|P63241	Isoform 2 c	EIF5A	20 kDa	5483800	6750100	5216300	2727500	2433000
tr|G3V325	Protein ATI	ATP5J2-PT(	84 kDa	7256800	17900000	8178100	3906000	5660500
sp|Q15907	Ras-related	RAB11B	24 kDa	1.00E+07	12200000	10900000	9529200	8938700
sp|Q5SSJ5	Heterochrc	HP1BP3	61 kDa	5086400	24200000	1.10E+07	2747300	10200000
sp|Q63ZY3	Isoform 2 c	KANK2	92 kDa	2507700	3306700	836400	458720	1703400
sp|P63208	S-phase kir	SKP1	19 kDa	5118800	12900000	8034500	3889300	4365400
sp|P00325	Alcohol del	ADH1B	40 kDa	23300000	33600000	19800000	11800000	16800000
sp|CYB5_H	sp|CYB5_H	CYB5	15 kDa	26900000	43200000	40900000	11400000	27800000
sp|O94832	Unconvent	MYO1D	116 kDa	2647900	8855500	1.10E+07	2521700	6034600
sp|O94919	Endonuclea	ENDOD1	55 kDa	7163600	16300000	6621300	3257300	5874900
sp|P27144	Adenylate l	AK4	25 kDa	45300000	3.60E+07	42100000	18200000	27700000
sp|P46108	Isoform Crl	CRK	23 kDa	7599600	1286900	6318400	661190	676430
sp|P13667	Protein disı	PDIA4	73 kDa	7395200	3330600	1075100	0	11600000
sp|Q14019	Coactosin-l	COTL1	16 kDa	21500000	8736200	5594400	8643100	5667300
sp|O75367	Isoform 1 c	H2AFY	39 kDa	19500000	3.00E+07	2.10E+07	13500000	28800000
sp|P62701	40S ribosor	RPS4X	30 kDa	7821000	18600000	16400000	7482700	19300000
sp|O75131	Copine-3	CPNE3	60 kDa	3108100	2705200	3590000	777380	3412900
sp|P06703	Protein S1(	S100A6	10 kDa	0	0	3622000	0	0
sp|P07954	Isoform Cyt	FH	50 kDa	17200000	778350	4699500	10400000	6667900
sp|P09972	Fructose-bi	ALDOC	39 kDa	12500000	11900000	8653600	4485900	7602700

sp|Q14315	Isoform 2 c	FLNC	287 kDa	21800000	37900000	6321400	9466900	2.20E+07
sp|P17252	Protein kin	PRKCA	77 kDa	0	7746800	4950000	0	8553200
sp|Q9UBG(	C-type mar	MRC2	167 kDa	0	2223700	1906800	0	1114200
sp|Q8WUP	Isoform 3 c	FBLIM1	31 kDa	18700000	3.10E+07	25300000	6372800	1.30E+07
sp|Q13409	Isoform 2B	DYNC1I2	71 kDa	0	9153200	9360100	4538000	5812900
sp|O75915	PRA1 famil	ARL6IP5	22 kDa	6514600	23900000	14900000	6429400	1.00E+07
sp|P09429	High mobil	HMGB1	25 kDa	16700000	52100000	12500000	8906100	1.40E+07
sp|P0CW2.	40S riboson	RPS17L	16 kDa	3917800	17400000	9709600	2635700	3900100
sp|P20160	Azurocidin	AZU1	27 kDa	0	0	0	0	0
sp|Q92882	Osteoclast-	OSTF1	24 kDa	1291500	2180200	10300000	919960	1383800
sp|Q14011	Cold-induci	CIRBP	19 kDa	6266100	5373800	8395800	1756400	2065300
sp|P42166	Lamina-ass	TMPO	75 kDa	5395400	9561200	6280700	1809200	1790500
sp|Q16543	Hsp90 co-c	CDC37	44 kDa	5254600	1062700	6951400	5468100	4427700
sp|P12273	Prolactin-ir	PIP	17 kDa	0	0	0	0	0
sp|P15090	Fatty acid-l	FABP4	15 kDa	18900000	16900000	20500000	11300000	15800000
sp|P15880	40S riboson	RPS2	31 kDa	10200000	14700000	1467600	8580900	9294200
sp|P23526	Adenosylh	AHCY	48 kDa	9330700	9652200	0	3523900	8891600
sp|P62714	Serine/thre	PPP2CB	36 kDa	8978400	0	4940200	1335400	3987200
sp|Q07960	Rho GTPas	ARHGAP1	50 kDa	12600000	14500000	11800000	4181300	3559400
sp|Q13835	Isoform 1 c	PKP1	80 kDa	0	0	0	0	0
sp|Q15121	Isoform 2 c	PEA15	17 kDa	1.50E+07	27500000	15600000	6570800	0
sp|Q16762	Thiosulfate	TST	33 kDa	7085800	39700000	7135900	2720800	5710500
sp|Q9H2G.	Isoform 2 c	SLK	139 kDa	11800000	13600000	17300000	7441900	7655500
sp|O95336	6-phospho	PGLS	28 kDa	2763000	24900000	17300000	12100000	26300000
sp|P30837	Aldehyde d	ALDH1B1	57 kDa	4804100	2541400	1660700	4139500	3214700
sp|P31146	Coronin-1A	CORO1A	51 kDa	3215000	0	3741300	0	2899200
sp|P31323	cAMP-depe	PRKAR2B	46 kDa	14900000	21700000	26600000	3608400	12400000
sp|P84103	Isoform 2 c	SRSF3	14 kDa	6040500	10800000	43700000	18100000	33900000
sp|P07384	Calpain-1 c	CAPN1	82 kDa	2088500	6057400	4680600	875500	6870300
sp|P26373	60S riboson	RPL13	24 kDa	9299000	13800000	8978800	3299700	0
sp|P11216	Glycogen p	PYGB	97 kDa	799150	11100000	8915700	2593100	7508900
sp|P46782	40S riboson	RPS5	23 kDa	3964500	5688200	3280000	946160	2377300
sp|P61247	40S riboson	RPS3A	30 kDa	0	7114700	0	3019400	5920800
sp|P80404	4-aminobu	ABAT	56 kDa	6772900	13800000	2887500	3567800	2979500
sp|Q03518	Antigen pe	TAP1	87 kDa	2314400	7171000	5671800	916670	0
sp|Q9NZN!	Isoform 2 c	ARHGEF12	171 kDa	4822600	35500000	320180	0	2.10E+07
sp|Q04917	14-3-3 prot	YWHAH	28 kDa	16300000	19800000	1.27E+08	40400000	87900000
sp|Q9Y3Z3	Isoform 2 c	SAMHD1	69 kDa	0	0	918000	1561400	479430
sp|P30711	Glutathion	GSTT1	27 kDa	0	15200000	5912200	2106300	0
sp|P30048	Isoform 2 c	PRDX3	26 kDa	6777200	9291200	8326700	6065100	7386000
sp|Q13867	Bleomycin	BLMH	53 kDa	0	0	0	1322800	0
sp|P62269	40S riboson	RPS18	18 kDa	6304100	10400000	8267700	0	6402000
sp|P61769	Beta-2-mic	B2M	14 kDa	0	6410900	5733600	4349300	4132200
sp|P49368	T-complex	CCT3	61 kDa	0	1248900	0	748420	6549600
sp|O43488	Aflatoxin B	AKR7A2	40 kDa	3222200	4560400	0	8347900	1.20E+07
sp|P09496	Isoform No	CLTA	24 kDa	12100000	20300000	23700000	2807200	6458500
sp|O94973	Isoform 2 c	AP2A2	104 kDa	1523100	1964000	1367200	591680	1479000

sp|O60664	Isoform 4 c	PLIN3	46 kDa	0	13100000	761480	1873100	7783000
sp|P05090	Apolipopro	APOD	21 kDa	0	0	0	0	0
sp|P30050	60S ribosor	RPL12	18 kDa	5036100	0	4737900	2476500	0
sp|P43686	Isoform 2 c	PSMC4	44 kDa	2830800	1964300	3671900	977740	720840
sp|Q12965	Unconvent	MYO1E	127 kDa	4207500	11600000	6453400	0	7411200
sp|Q9NRV!	Heme-bind	HEBP1	21 kDa	9477400	13700000	12700000	3410400	7904000
sp|Q9Y3I0	tRNA-splici	RTCB	55 kDa	6966600	9152000	8612700	0	5392500
sp|Q14117	Dihydropyr	DPYS	57 kDa	2618400	3912100	2183300	681020	570610
sp|P13164	Interferon-	IFITM1	14 kDa	0	0	0	0	4155600
sp|Q9Y2D!	Isoform 3 c	AKAP2	104 kDa	6466900	12100000	12700000	5373100	11800000
sp|P39023	60S ribosor	RPL3	46 kDa	5831100	5653000	0	4067100	4792600
sp|P09936	Ubiquitin c	UCHL1	25 kDa	2.40E+07	34300000	28500000	4911500	1.10E+07
sp|P11766	Alcohol del	ADH5	40 kDa	6082500	26200000	23700000	1.00E+07	10400000
sp|Q14574	Desmocolli	DSC3	100 kDa	0	0	0	0	0
sp|Q96CM	Isoform 2 c	ACSF2	71 kDa	19600000	7029800	15200000	7922100	8768500
sp|Q9Y2S2	Isoform 2 c	CRYL1	33 kDa	9978000	8103500	9085500	4980800	9173300
sp|P56537	Eukaryotic	EIF6	27 kDa	2319100	2259700	1837000	0	0
sp|P63167	Dynein ligh	DYNLL1	10 kDa	8404100	0	0	2675400	4573300
sp|P22352	Glutathion	GPX3	26 kDa	17200000	36300000	21500000	5409400	10900000
sp|P07858	Cathepsin I	CTSB	38 kDa	0	0	0	0	0
sp|P01833	Polymeric i	PIGR	83 kDa	0	0	0	0	0
sp|P61019	Isoform 2 c	RAB2A	21 kDa	13700000	14900000	1.40E+07	7201100	23700000
sp|O43491	Band 4.1-lil	EPB41L2	113 kDa	8120100	1390200	19600000	5586900	5695700
sp|P60900	Isoform 2 c	PSMA6	25 kDa	8718000	4237900	7361800	4086000	2255500
sp|O75347	Tubulin-spe	TBCA	13 kDa	0	0	0	0	0
sp|Q9UPZ6	Thrombosp	THSD7A	185 kDa	0	0	0	1271200	0
sp|P35080	Isoform IIb	PFN2	15 kDa	0	0	0	8336900	16100000
sp|Q13148	TAR DNA-b	TARDBP	45 kDa	0	0	0	958560	2406200
sp|P01859	Ig gamma-2	IGHG2	36 kDa	48200000	74100000	56900000	2.40E+07	45600000
sp|P62328	Thymosin k	TMSB4X	5 kDa	0	0	0	0	0
sp|Q96QA!	Gasdermin	GSDMA	49 kDa	0	0	0	0	0
sp|Q9UQ0	Isoform 2 c	CORO2B	54 kDa	0	0	0	1360300	0
sp|P36578	60S ribosor	RPL4	48 kDa	15100000	18700000	1.80E+07	1482400	5530700
sp|P62917	60S ribosor	RPL8	28 kDa	9002700	13200000	8779200	4071700	8479600
sp|Q96G03	Phosphoglu	PGM2	68 kDa	6208500	7404600	3396900	0	2836500
sp|P00390	Isoform Cy	GSR	52 kDa	11100000	16300000	20500000	9251200	7919900
sp|P13861	Isoform 2 c	PRKAR2A	43 kDa	8747900	425030	9004700	1738800	9343800
sp|Q96DG6	Carboxyme	CMBL	28 kDa	12300000	4127100	6800200	3474100	3633500
sp|Q96P63	Isoform 2 c	SERPINB12	48 kDa	0	0	0	0	0
sp|Q9HDC!	Isoform 2 c	APMAP	32 kDa	9289800	12400000	15200000	3549000	3942000
sp|P31689	DnaJ homo	DNAJA1	45 kDa	0	4535000	4755900	2063100	2708100
sp|P46777	60S ribosor	RPL5	34 kDa	0	0	0	0	0
tr|J3KQL8|	Apolipopro	APOL2	49 kDa	472460	6676600	0	0	0
sp|P52597	Heterogen	HNRNPF	46 kDa	13500000	2.70E+07	14700000	7648000	17700000
sp|P98172	Ephrin-B1	EFNB1	38 kDa	4502200	8697900	5683700	1395400	5242300
sp|P98088	Mucin-5AC	MUC5AC	586 kDa	0	0	0	0	0
sp|P12955	Isoform 2 c	PEPD	50 kDa	2881000	3636700	0	1759500	2639100

sp|Q53GQ(	Very-long-c	HSD17B12	34 kDa	8989000	15200000	7728400	1803300	5977400
sp|P28066	Proteasom	PSMA5	26 kDa	2501700	3151800	0	1509700	0
sp|P35268	60S ribosor	RPL22	15 kDa	8863500	10400000	10600000	4321200	7903500
sp|P45974	Isoform Sh	USP5	93 kDa	1433300	2460600	846800	419150	4692400
sp|P36957	Dihydrolipo	DLST	49 kDa	5206300	5842000	7429700	2155400	5896800
sp|O95865	N(G),N(G)-(	DDAH2	30 kDa	3456900	6880400	8904400	5174800	7521700
sp|Q07955	Isoform AS	SRSF1	32 kDa	17700000	26600000	16900000	7666500	9693500
sp|Q9ULA(	Aspartyl an	DNPEP	52 kDa	0	0	9300300	0	0
sp|FABPH_	sp|FABPH_	FABPH	15 kDa	6621800	6849200	7472900	0	3971100
sp|O43242	26S protea	PSMD3	61 kDa	1214800	1158100	1062700	744510	1101500
sp|P00740	Coagulatio	F9	52 kDa	18900000	33200000	19400000	3312000	5846400
sp|P24158	Myeloblast	PRTN3	28 kDa	0	0	0	0	0
sp|P46778	60S ribosor	RPL21	19 kDa	0	3680000	4543300	1174700	997480
sp|P51884	Lumican	LUM	38 kDa	0	1879200	165830	3889100	5220600
sp|O00461	Golgi integ	GOLIM4	82 kDa	0	2343800	0	0	2655400
sp|P48444	Coatomer s	ARCN1	57 kDa	683860	3300000	3772200	1395400	4411400
sp|Q96CW	Isoform 2 c	AP2M1	49 kDa	5547600	8550300	10700000	0	7503900
sp|Q9UEY8	Isoform 1 c	ADD3	76 kDa	9149300	24500000	12100000	1724600	1453900
sp|Q96C23	Aldose 1-ep	GALM	38 kDa	1885700	903060	0	0	0
sp|P50570	Isoform 4 c	DNM2	98 kDa	2478900	4142800	2138700	2866600	5992400
sp|Q01130	Isoform 2 c	SRSF2	24 kDa	2865700	6147700	3258800	0	3222600
sp|P01040	Cystatin-A	CSTA	11 kDa	0	0	0	0	0
sp|P62829	60S ribosor	RPL23	15 kDa	0	3333400	0	1864500	0
sp|Q8NI99	Angiopoiet	ANGPTL6	52 kDa	0	0	0	0	0
sp|Q9HCY8	Protein S10	S100A14	12 kDa	0	0	0	0	0
tr|F6TLX2|	Glyoxalase	GLOD4	55 kDa	8581500	26800000	8432400	4695900	11400000
sp|Q9BRA2	Thioredoxi	TXNDC17	14 kDa	12300000	15500000	5810800	0	5165200
sp|P28289	Tropomodu	TMOD1	41 kDa	0	3573300	3586300	1070300	0
sp|Q9HD89	Resistin	RETN	11 kDa	0	0	0	0	0
sp|O00487	26S protea	PSMD14	35 kDa	2753200	3414200	1329900	1460800	2040800
sp|P36542	ATP syntha	ATP5C1	33 kDa	5499900	8997000	8594800	2472700	8563600
sp|Q7L576	Cytoplasmi	CYFIP1	145 kDa	1700700	3756100	0	901640	1232300
sp|P11177	Isoform 2 c	PDHB	37 kDa	6791700	9704400	8326100	2905900	4506400
sp|Q9BQI0	Allograft in	AIF1L	17 kDa	0	0	0	0	0
sp|Q8N3R9	Isoform 2 c	MPP5	73 kDa	2425400	4472500	8440500	2015500	8635000
sp|P51572	Isoform 2 c	BCAP31	35 kDa	13600000	17500000	9962600	3428000	0
sp|P78417	Glutathion	GSTO1	28 kDa	5311200	5908700	0	2743900	5315000
sp|P36269	Isoform 2 c	GGT5	59 kDa	2409700	1043800	2659700	1615200	4166600
sp|O15400	Isoform 2 c	STX7	27 kDa	4873200	6448700	10200000	4384700	3660100
sp|P12724	Eosinophil	RNASE3	18 kDa	0	0	0	0	0
sp|P61970	Nuclear tra	NUTF2	14 kDa	0	4346500	2235100	2373400	2752700
sp|Q00325	Isoform B c	SLC25A3	40 kDa	40100000	56600000	38300000	18300000	35900000
sp|P62263	40S ribosor	RPS14	16 kDa	0	4426800	2796300	0	0
tr|F5H2F4|	C-1-tetrahy	MTHFD1	111 kDa	5830000	7450600	4596600	1851200	2577400
sp|P07148	Fatty acid-l	FABP1	14 kDa	0	0	0	0	0
sp|O00232	26S protea	PSMD12	53 kDa	2154600	3265800	1657300	1309900	1952300
tr|M0QYS1	60S ribosor	RPL13A	24 kDa	0	0	0	815840	0

sp|P53621	Isoform 2 c	COPA	139 kDa	0	4414900	2303100	1497400	4458500
tr|H0Y300|	Haptoglobi	HP	49 kDa	0	0	0	0	0
sp|Q9UI42	Isoform 2 c	CPA4	44 kDa	0	0	0	0	0
sp|O75891	Isoform 3 c	ALDH1L1	100 kDa	1377300	1656700	2894400	1546600	984660
sp|P02790	Hemopexir	HPX	52 kDa	6338300	5987600	5465700	1973200	0
sp|P62888	60S ribosor	RPL30	13 kDa	3062900	0	0	0	0
sp|P00441	Superoxide	SOD1	16 kDa	0	0	0	0	0
sp|Q8NBS9	Thioredoxi	TXNDC5	48 kDa	3163500	2237000	4371000	0	2631800
sp|P32969	60S ribosor	RPL9	22 kDa	3044300	4754000	4429400	4222200	7699200
sp|P22695	Cytochrom	UQCRC2	48 kDa	4920800	13600000	6765100	0	7865500
sp|P62081	40S ribosor	RPS7	22 kDa	0	0	5743900	2088100	3018200
sp|P49593	Protein phc	PPM1F	50 kDa	7529800	11200000	0	3043800	6355600
sp|Q13185	Chromobo›	CBX3	21 kDa	385390	5182200	357530	0	1985300
sp|O15511	Actin-relate	ARPC5	16 kDa	10900000	13200000	0	0	0
sp|Q02818	Nucleobinc	NUCB1	54 kDa	0	0	0	0	0
sp|P09619	Platelet-de	PDGFRB	124 kDa	0	0	9177700	2730400	7801900
sp|P31930	Cytochrom	UQCRC1	53 kDa	0	0	0	763460	3603900
sp|P55000	Secreted Ly	SLURP1	11 kDa	0	0	0	0	0
sp|Q9UNZ2	Isoform 3 c	NSFL1C	41 kDa	2227400	0	0	0	2969400
sp|Q9Y2A7	Isoform 2 c	NCKAP1	130 kDa	666250	0	523830	1325200	0
sp|P49591	Serine--tRN	SARS	59 kDa	0	1180500	979310	759140	1620700
sp|P49755	Transmeml	TMED10	25 kDa	4373000	10100000	8080900	3046000	7557800
sp|P35270	Sepiapterir	SPR	28 kDa	466700	399290	0	0	0
sp|P39656	Isoform 2 c	DDOST	47 kDa	109340	3361700	570420	1466900	3082400
sp|Q96AM	Mas-relate	MRGPRF	38 kDa	0	0	0	0	0
sp|Q9UJC5	SH3 domai	SH3BGRL2	12 kDa	0	0	0	0	2465300
sp|O75531	Barrier-to-a	BANF1	10 kDa	8961500	0	0	0	6095400
sp|P31937	3-hydroxyis	HIBADH	35 kDa	4442000	0	0	0	0
sp|P17980	26S protea	PSMC3	49 kDa	1850100	2931900	0	1649300	3765800
sp|P62753	40S ribosor	RPS6	29 kDa	4987000	6232000	0	0	0
sp|P62899	60S ribosor	RPL31	14 kDa	0	13500000	0	0	9771600
sp|P22735	Protein-glu	TGM1	90 kDa	0	0	0	0	0
sp|P62191	26S protea	PSMC1	49 kDa	0	3430000	0	0	2988300
sp|Q9Y5K6	CD2-associ	CD2AP	71 kDa	409460	3765400	3303900	4677700	5173300
sp|P04080	Cystatin-B	CSTB	11 kDa	0	0	0	0	0
sp|O75390	Citrate synt	CS	52 kDa	8584100	1.50E+07	9220800	10400000	14100000
sp|P53396	ATP-citrate	ACLY	121 kDa	2687000	3635900	389680	265800	1803400
sp|O95183	Vesicle-ass	VAMP5	13 kDa	4634500	0	4794400	2011700	4686000
sp|P07814	Bifunctiona	EPRS	171 kDa	0	1300200	681490	0	1228400
sp|P05230	Fibroblast g	FGF1	17 kDa	971340	1565700	1168300	616400	824860
sp|P51858	Hepatoma-	HDGF	27 kDa	10400000	16300000	9047500	0	8688700
sp|P52566	Rho GDP-d	ARHGDIB	23 kDa	0	0	0	0	0
sp|Q6IB77	Glycine N-a	GLYAT	34 kDa	0	0	7573800	0	0
sp|P12429	Annexin A3	ANXA3	36 kDa	0	6725500	0	3577700	1339000
sp|P34896	Isoform 2 c	SHMT1	49 kDa	6733300	0	11800000	9098900	8450500
sp|Q5T750	Skin-specifi	XP32	26 kDa	0	0	0	0	0
sp|Q9C075	Keratin, typ	KRT23	48 kDa	0	0	0	0	0

sp|O14494	Isoform 2 c	PPAP2A	32 kDa	8944900	21100000	9196800	8156300	18500000
sp|Q99733	Isoform 2 c	NAP1L4	44 kDa	0	672170	0	255840	453820
sp|O14980	Exportin-1	XPO1	123 kDa	1092600	1428200	1728200	1027800	0
sp|O75936	Gamma-bu	BBOX1	45 kDa	3302500	0	0	0	2505200
sp|P23284	Peptidyl-pr	PPIB	24 kDa	0	0	0	0	0
sp|P47929	Galectin-7	LGALS7	15 kDa	0	0	0	0	0
sp|P62330	ADP-ribosy	ARF6	20 kDa	7469900	37100000	4240600	1922500	8591300
sp|Q9Y678	Coatomer s	COPG1	98 kDa	0	0	1120700	0	2787500
sp|P53597	Succinyl-Cc	SUCLG1	36 kDa	14200000	17600000	7339100	1810300	0
sp|Q92804	Isoform Sh	TAF15	62 kDa	7343700	8076500	8668100	1980600	3961000
sp|P31939	Isoform 2 c	ATIC	65 kDa	0	11700000	503080	0	3369700
sp|Q14108	Lysosome r	SCARB2	54 kDa	0	0	0	0	0
sp|Q9NZ08	Isoform 2 c	ERAP1	108 kDa	1541500	2194700	1385900	870180	1315900
sp|O75954	Tetraspanir	TSPAN9	27 kDa	6070300	9793400	5785200	2986900	4734900
sp|P29279	Connective	CTGF	38 kDa	0	0	0	0	2501300
sp|Q0VF96	Cingulin-lik	CGNL1	149 kDa	2344000	3906300	10400000	6140200	0
sp|Q9BX68	Histidine tr	HINT2	17 kDa	3246300	3582600	2769400	2177100	2678500
sp|Q9NR31	GTP-bindin	SAR1A	22 kDa	2265000	2821500	2264100	832760	1097100
sp|P20700	Lamin-B1	LMNB1	66 kDa	2473000	15100000	3601700	1856500	9770800
sp|P02647	Apolipopro	APOA1	31 kDa	0	0	0	0	0
sp|Q8N335	Glycerol-3-	GPD1L	38 kDa	8403000	0	856250	4107300	8910800
sp|Q9NZN4	EH domain	EHD2	61 kDa	3470700	3295000	3299900	1419600	3547400
sp|Q9P2R7	Isoform 2 c	SUCLA2	48 kDa	8062200	0	4923300	0	0
sp|P53004	Biliverdin r	BLVRA	33 kDa	29600000	50100000	38700000	12800000	20800000
sp|P21266	Glutathion	GSTM3	27 kDa	0	4161100	2912100	0	0
sp|Q14894	Ketimine re	CRYM	34 kDa	0	0	2059200	403800	1643700
sp|Q6NUJ1	Proactivato	PSAPL1	57 kDa	0	0	0	0	0
sp|P28065	Isoform LM	PSMB9	22 kDa	0	1474400	0	0	0
sp|P30043	Flavin redu	BLVRB	22 kDa	0	0	0	0	0
sp|P61353	60S ribosor	RPL27	16 kDa	0	0	0	2371800	9155200
sp|Q9Y224	UPF0568 p	C14orf166	28 kDa	0	4098600	0	0	0
sp|Q8NC56	LEM domai	LEMD2	57 kDa	0	10900000	4524100	0	8753500
sp|O60884	DnaJ homo	DNAJA2	46 kDa	0	0	0	601830	854940
sp|P25325	Isoform 2 c	MPST	35 kDa	891430	3289600	1599300	2544900	3797300
sp|Q9UBR2	Cathepsin Z	CTSZ	34 kDa	0	0	0	0	0
sp|P46783	40S ribosor	RPS10	19 kDa	1085100	0	0	792710	783810
sp|P03973	Antileukop	SLPI	14 kDa	0	0	0	0	0
sp|P11215	Isoform 2 c	ITGAM	127 kDa	0	0	0	0	0
sp|P80188	Neutrophil	LCN2	23 kDa	0	0	0	0	0
sp|Q6YN16	Hydroxyste	HSDL2	45 kDa	3524900	3314400	2261000	0	0
sp|Q96CX2	BTB/POZ d	KCTD12	36 kDa	0	0	0	2558500	4897400
sp|Q9Y617	Phosphose	PSAT1	40 kDa	6514300	10900000	0	1143800	0
sp|P84098	60S ribosor	RPL19	23 kDa	0	0	0	0	0
sp|Q8NFJ5	Retinoic ac	GPRC5A	40 kDa	0	1.80E+07	7727300	6734500	10700000
sp|Q9UL25	Ras-related	RAB21	24 kDa	0	6003700	0	0	0
sp|Q15274	Nicotinate-	QPRT	31 kDa	0	8403200	5866200	3322400	0
sp|P31948	Isoform 2 c	STIP1	68 kDa	6568700	8727000	6656700	3295200	7774900

sp|O00264	Membrane	PGRMC1	22 kDa	0	0	0	0	0
sp|P61960	Ubiquitin-f	UFM1	9 kDa	1425200	2582300	1449800	1115900	3584700
sp|O95154	Aflatoxin B	AKR7A3	37 kDa	5806700	0	0	2761500	0
sp|O15173	Isoform 2 c	PGRMC2	26 kDa	4319500	0	4486500	5971000	10400000
sp|P43034	Platelet-act	PAFAH1B1	47 kDa	0	0	0	0	0
sp|P62913	Isoform 2 c	RPL11	20 kDa	12500000	15200000	7278300	3633400	11500000
sp|P10253	Lysosomal	GAA	105 kDa	0	0	0	0	0
sp|Q96NY7	Isoform A c	CLIC6	71 kDa	18900000	22700000	12200000	13800000	18900000
sp|P31025	Lipocalin-1	LCN1	19 kDa	0	0	0	0	0
sp|O75223	Gamma-glu	GGCT	21 kDa	0	0	0	0	0
sp|P35754	Glutaredox	GLRX	12 kDa	0	0	0	0	0
sp|O15247	Chloride in	CLIC2	28 kDa	0	4654000	3000100	0	0
sp|P09497	Isoform No	CLTB	23 kDa	0	0	0	0	0
sp|P16401	Histone H1	HIST1H1B	23 kDa	0	0	0	0	0
sp|P20618	Proteasom	PSMB1	26 kDa	0	0	0	0	0
sp|Q9P1F3	Costars fan	ABRACL	9 kDa	0	0	0	0	0
sp|P62318	Isoform 2 c	SNRPD3	13 kDa	0	0	0	0	0
sp|Q9Y6I3-	Isoform 2 c	EPN1	69 kDa	0	0	0	0	0
sp|O75436	Vacuolar pr	VPS26A	38 kDa	2937900	0	4329900	0	4294600
sp|P01861	Ig gamma-4	IGHG4	36 kDa	52900000	67500000	58600000	88500000	1.59E+08
sp|P14780	Matrix met	MMP9	78 kDa	0	0	0	0	0
sp|Q8WVV	Isoform 1 c	POF1B	69 kDa	0	0	0	0	0
sp|P01008	Antithromb	SERPINC1	53 kDa	0	3604900	2852100	996300	3878800
sp|O43175	D-3-phosph	PHGDH	57 kDa	4339800	100070	3931100	0	0
sp|P11233	Ras-related	RALA	24 kDa	4527300	8553200	910190	0	3225900
sp|P09417	Dihydropte	QDPR	26 kDa	0	0	0	0	0
sp|Q9UIJ7|	GTP:AMP p	AK3	26 kDa	2129500	0	8296300	1098000	1018400
sp|P49588	Isoform 2 c	AARS	109 kDa	0	0	0	0	1797900
sp|P05107	Integrin be	ITGB2	85 kDa	0	0	0	0	0
sp|P24534	Elongation	EEF1B2	25 kDa	0	0	7545800	0	10100000
sp|Q07021	Compleme	C1QBP	31 kDa	0	0	0	0	0
sp|Q96MM	Heat shock	HSPA12B	76 kDa	0	0	7189200	1391700	10800000
sp|P09543	Isoform CN	CNP	45 kDa	2637500	2958600	4367100	1679000	2440300
sp|P13473	Isoform LA	LAMP2	45 kDa	0	0	0	0	0
sp|Q8TDL5	BPI fold-co	BPIFB1	52 kDa	0	0	0	0	0
sp|Q9NP55	Isoform 2 c	BPIFA1	25 kDa	0	0	0	0	0
sp|Q13155	Aminoacyl	AIMP2	35 kDa	0	8063400	0	0	0
sp|O14561	Acyl carrier	NDUFAB1	17 kDa	0	0	0	0	0
sp|P50148	Guanine nu	GNAQ	42 kDa	0	6746100	214660	0	3577400
sp|O75489	NADH dehy	NDUFS3	30 kDa	0	0	0	0	6081400
sp|P30040	Endoplasm	ERP29	29 kDa	3722400	0	0	0	2035400
sp|Q92747	Actin-relate	ARPC1A	42 kDa	5151300	0	0	1744000	6722900
sp|Q9Y277	Isoform 2 c	VDAC3	31 kDa	10700000	19300000	10800000	2099400	14800000
sp|P51571	Translocon	SSR4	19 kDa	4326100	7370600	11400000	4572500	4839200
sp|P25815	Protein S10	S100P	10 kDa	0	0	0	0	0
sp|Q6ZVX7	F-box only	NCCRP1	31 kDa	0	0	0	0	0
sp|Q96HE7	ERO1-like p	ERO1L	54 kDa	4106500	0	3117700	332440	562920

sp|Q9NQW	Isoform 3 c	XPNPEP1	75 kDa	0	3473000	0	0	702820
sp|P01591	Immunoglc	IGJ	18 kDa	0	0	0	0	0
sp|P31947	Isoform 2 c	SFN	24 kDa	0	0	89100000	35800000	7.60E+07
sp|P51452	Dual specif	DUSP3	20 kDa	0	2878900	0	0	3527900
sp|Q16378	Proline-rich	PRR4	15 kDa	0	0	0	0	0
sp|Q9H444	Charged m	CHMP4B	25 kDa	0	4608400	0	0	0
sp|Q9Y287	Integral me	ITM2B	30 kDa	0	0	0	0	0
sp|Q15293	Reticulocal	RCN1	39 kDa	0	0	0	0	0
sp|Q6DN03	Putative his	HIST2H2BC	21 kDa	28100000	5.60E+07	25500000	2.40E+07	0
sp|Q8NCW	NAD(P)H-h	APOA1BP	32 kDa	3366000	0	7231100	0	6025400
sp|P59827	Isoform 2 c	BPIFB4	61 kDa	0	0	0	0	0
sp|O75342	Arachidona	ALOX12B	80 kDa	0	0	0	0	0
sp|P13073	Cytochrom	COX4I1	20 kDa	0	8271800	4859900	0	4184600
sp|P35222	Catenin be	CTNNB1	85 kDa	0	0	0	0	0
sp|P49913	Cathelicidir	CAMP	19 kDa	0	0	0	0	0
sp|Q5VU13	V-set and ir	VSIG8	44 kDa	0	0	0	0	0
sp|P62277	40S ribosor	RPS13	17 kDa	0	0	0	0	0
sp|Q96DA0	Zymogen g	ZG16B	23 kDa	0	0	0	0	0
sp|Q9H853	Putative tu	TUBA4B	28 kDa	72900000	90600000	71700000	47800000	77400000
sp|P20061	Transcobal	TCN1	48 kDa	0	0	0	0	0
sp|Q02543	60S ribosor	RPL18A	21 kDa	0	0	0	0	0
sp|Q14651	Plastin-1	PLS1	70 kDa	37800000	53700000	47200000	12900000	3.10E+07
sp|P25788	Isoform 2 c	PSMA3	28 kDa	0	0	0	0	0
sp|P01620	Ig kappa ch	KV302	12 kDa	0	0	0	0	0
sp|P21333	Isoform 2 c	FLNA	280 kDa	1.85E+09	2.76E+09	2.15E+09	NA	NA
sp|P02533	Keratin, typ	KRT14	52 kDa	1.05E+09	9.00E+07	1.98E+08	NA	NA
sp|P63261	Actin, cyto	ACTG1	42 kDa	1.97E+10	2.65E+10	2.25E+10	NA	NA
sp|P02671	Fibrinogen	FGA	95 kDa	1.11E+09	9.42E+08	5.24E+08	NA	NA
sp|P06396	Isoform 2 c	GSN	81 kDa	6.87E+08	1.12E+09	7.82E+08	NA	NA
sp|Q6PEY2	Tubulin alp	TUBA3E	50 kDa	1.99E+09	2.95E+09	2.21E+09	NA	NA
sp|P53675	Isoform 2 c	CLTCL1	180 kDa	2.27E+08	3.54E+08	2.87E+08	NA	NA
sp|P29400	Isoform 2 c	COL4A5	162 kDa	9.17E+08	5.81E+08	6.14E+08	NA	NA
tr|G3V1L9|	Tight juncti	TJP1	197 kDa	2.09E+08	4.44E+08	2.79E+08	NA	NA
sp|P14618	Isoform M1	PKM	58 kDa	4.42E+08	5.81E+08	4.25E+08	NA	NA
sp|Q8WZ4	Isoform 6 c	TTN	632 kDa	0	0	0	NA	NA
sp|Q9NZN3	EH domain	EHD3	61 kDa	1.65E+08	4.11E+08	2.27E+08	NA	NA
sp|P39060	Collagen al	COL18A1	178 kDa	1.64E+08	1.65E+08	1.67E+08	NA	NA
sp|Q5VTE0	Putative elc	EEF1A1P5	50 kDa	4.17E+08	5.37E+08	4.97E+08	NA	NA
sp|O00159	Unconvent	MYO1C	122 kDa	87900000	1.76E+08	1.29E+08	NA	NA
sp|Q96AC1	Fermitin fa	FERMT2	78 kDa	96600000	1.75E+08	1.63E+08	NA	NA
sp|P35580	Isoform 2 c	MYH10	231 kDa	2.87E+08	4.26E+08	4.33E+08	NA	NA
sp|Q05682	Caldesmon	CALD1	93 kDa	1.77E+08	2.33E+08	1.79E+08	NA	NA
sp|P68871	Hemoglobi	HBB	16 kDa	3.14E+08	4.72E+08	3.37E+08	NA	NA
sp|P50395	Rab GDP di	GDI2	51 kDa	91600000	1.52E+08	87600000	NA	NA
sp|P05023	Isoform 3 c	ATP1A1	110 kDa	74700000	1.61E+08	94900000	NA	NA
sp|P04075	Isoform 2 c	ALDOA	45 kDa	1.92E+08	2.52E+08	2.25E+08	NA	NA
sp|P10909	Isoform 5 c	CLU	54 kDa	1.46E+08	2.76E+08	1.42E+08	NA	NA

sp|Q27J81	Inverted fo	INF2	136 kDa	16300000	45800000	32600000	NA	NA
sp|P31946	Isoform Sh	YWHAB	28 kDa	1.75E+08	2.59E+08	3.05E+08	NA	NA
sp|Q9Y281	Isoform 3 c	CFL2	17 kDa	2.62E+08	3.64E+08	2.41E+08	NA	NA
sp|O75369	Isoform 8 c	FLNB	282 kDa	65700000	1.27E+08	55600000	NA	NA
sp|P02458	Collagen al	COL2A1	142 kDa	0	0	0	NA	NA
sp|A6NIZ1	Ras-related	RP1BL	21 kDa	61900000	59700000	45400000	NA	NA
sp|P06756	Integrin alp	ITGAV	116 kDa	38500000	66700000	33900000	NA	NA
sp|Q14240	Eukaryotic	EIF4A2	46 kDa	40400000	74100000	93200000	NA	NA
sp|P23142	Fibulin-1	FBLN1	77 kDa	43400000	46400000	54700000	NA	NA
sp|Q15599	Isoform 2 c	SLC9A3R2	36 kDa	77300000	52600000	42800000	NA	NA
tr|C9JMN2	Collagen al	COL11A1	62 kDa	0	0	0	NA	NA
sp|P61204	ADP-ribosy	ARF3	21 kDa	26100000	31800000	16600000	NA	NA
tr|M0QZM	Heterogen	HNRNPM	40 kDa	19300000	6576500	15600000	NA	NA
sp|CATA_H	sp|CATA_H	CATA	60 kDa	35200000	43500000	20400000	NA	NA
sp|P05997	Collagen al	COL5A2	145 kDa	0	0	0	NA	NA
sp|CAH2_H	sp|CAH2_H	CAH2	29 kDa	1.83E+08	1.97E+08	1.99E+08	NA	NA
sp|Q15084	Isoform 4 c	PDIA6	49 kDa	34100000	72500000	40700000	NA	NA
sp|Q68CZ2	Isoform 2 c	TNS3	129 kDa	63400000	68400000	47500000	NA	NA
tr|J9JID7|J	Lamin B2, i	LMNB2	70 kDa	11200000	42100000	46200000	NA	NA
tr|A6NCT7	Collagen al	COL16A1	97 kDa	0	0	0	NA	NA
sp|P04899	Isoform sG	GNAI2	42 kDa	40300000	54200000	41900000	NA	NA
sp|P47756	Isoform 2 c	CAPZB	31 kDa	74200000	1.21E+08	8.70E+07	NA	NA
sp|P51991	Heterogen	HNRNPA3	40 kDa	53200000	97800000	73200000	NA	NA
sp|Q9UJU6	Drebrin-like	DBNL	48 kDa	28400000	28300000	26800000	NA	NA
tr|A0A087	Ig gamma-1	IGHG1	51 kDa	1.71E+08	2.18E+08	1.84E+08	NA	NA
sp|P00387	Isoform 2 c	CYB5R3	32 kDa	4.10E+07	92100000	6.30E+07	NA	NA
tr|G3V192	Ferritin	FTH1	18 kDa	0	5571100	0	NA	NA
sp|P06748	Isoform 2 c	NPM1	29 kDa	36500000	70800000	9838000	NA	NA
sp|O60506	Isoform 3 c	SYNCRIP	63 kDa	21900000	26300000	19800000	NA	NA
sp|Q15746	Isoform 2 c	MYLK	203 kDa	23900000	41700000	33400000	NA	NA
sp|Q86VP6	Cullin-asso	CAND1	136 kDa	4536500	3907200	3729900	NA	NA
sp|Q00839	Heterogen	HNRNPU	91 kDa	5.70E+07	97100000	62600000	NA	NA
sp|Q9UPA5	Protein bas	BSN	416 kDa	0	0	0	NA	NA
sp|Q9H2D6	TRIO and F	TRIOBP	261 kDa	0	0	0	NA	NA
sp|P48634	Isoform 3 c	PRRC2A	229 kDa	0	0	0	NA	NA
sp|P05141	ADP/ATP tr	SLC25A5	33 kDa	33700000	85100000	45700000	NA	NA
tr|H7BY57	Neurofasci	NFASC	117 kDa	13500000	28400000	28800000	NA	NA
sp|Q8IVF2	Isoform 3 c	AHNAK2	606 kDa	0	0	0	NA	NA
tr|H0Y5N9	Collagen al	COL12A1	80 kDa	0	0	0	NA	NA
sp|Q86UL8	Membrane	MAGI2	159 kDa	14400000	33200000	16900000	NA	NA
sp|Q8NEZ4	Isoform 3 c	KMT2C	548 kDa	0	0	0	NA	NA
sp|Q13561	Dynactin su	DCTN2	44 kDa	7214600	19300000	18400000	NA	NA
sp|P30044	Peroxiredo	PRDX5	22 kDa	30200000	47600000	24600000	NA	NA
sp|P19013	Keratin, typ	KRT4	57 kDa	4.60E+08	67600000	1.09E+08	NA	NA
sp|Q9UQ3	Serine/argi	SRRM2	300 kDa	0	0	0	NA	NA
tr|A0A087	Immunoglo	IGLL5	25 kDa	4.80E+07	48800000	62100000	NA	NA
tr|E9PBC6|	Transformi	TACC2	303 kDa	0	0	0	NA	NA

sp|Q12955	Ankyrin-3	ANK3	480 kDa	0	0	0	NA	NA
sp|P20929	Isoform 2 c	NEB	987 kDa	0	0	0	NA	NA
sp|O00429	Isoform 2 c	DNM1L	79 kDa	7900800	8258300	3877200	NA	NA
sp|P22392	Isoform 3 c	NME2	30 kDa	37900000	53700000	41100000	NA	NA
sp|Q8WUN	Programme	PDCD6IP	96 kDa	16100000	24400000	18300000	NA	NA
sp|O14686	Histone-lys	KMT2D	593 kDa	0	0	0	NA	NA
sp|P59665	Neutrophil	DEFA1	10 kDa	42600000	98300000	45800000	NA	NA
sp|P49748	Isoform 3 c	ACADVL	73 kDa	23900000	36900000	26500000	NA	NA
sp|P17813	Endoglin	ENG	71 kDa	8390300	2.40E+07	1.50E+07	NA	NA
sp|Q9NR1	PDZ and LII	PDLIM7	50 kDa	9904900	20900000	2.20E+07	NA	NA
tr|E9PCA1|	T-complex	CCT5	57 kDa	7263600	7889700	9907800	NA	NA
sp|P13804	Electron tr	ETFA	35 kDa	22600000	21300000	18200000	NA	NA
sp|Q17RW	Collagen al	COL24A1	175 kDa	0	0	0	NA	NA
sp|O75955	Isoform 2 c	FLOT1	42 kDa	4281200	11300000	7749700	NA	NA
sp|Q13642	Isoform 1 c	FHL1	32 kDa	27400000	44900000	37500000	NA	NA
sp|Q9P2P6	StAR-relate	STARD9	516 kDa	0	0	0	NA	NA
sp|Q7Z4I7	Isoform 2 c	LIMS2	42 kDa	6518700	6831800	12600000	NA	NA
sp|P20810	Calpastatin	CAST	77 kDa	1.20E+07	21400000	14600000	NA	NA
tr|A0A0A0	Protein-L-is	PCMT1	30 kDa	5133100	24600000	15300000	NA	NA
sp|P31040	Succinate c	SDHA	73 kDa	1878600	10800000	6217900	NA	NA
sp|P63244	Guanine nu	GNB2L1	35 kDa	0	35400000	29600000	NA	NA
sp|P07602	Isoform Sa	PSAP	58 kDa	831390	294800	216710	NA	NA
sp|Q14055	Collagen al	COL9A2	65 kDa	66600000	0	0	NA	NA
sp|P28799	Isoform 2 c	GRN	47 kDa	0	0	11800000	NA	NA
sp|Q7Z5J4	Retinoic ac	RAI1	203 kDa	10900000	0	0	NA	NA
sp|Q01844	Isoform 3 c	EWSR1	68 kDa	11100000	0	0	NA	NA
sp|Q9P2E9	Ribosome-l	RRBP1	152 kDa	7739200	20400000	9262700	NA	NA
tr|Q5STU3	Spliceosom	DDX39B	49 kDa	15300000	38600000	19200000	NA	NA
sp|Q6KB66	Keratin, typ	KRT80	51 kDa	0	0	34800000	NA	NA
sp|Q03001	Dystonin	DST	861 kDa	0	0	0	NA	NA
sp|Q14103	Isoform 3 c	HNRNPD	33 kDa	6439000	9868200	6964800	NA	NA
tr|H0Y586|	Proteasom	PSMA7	21 kDa	1.00E+07	1.00E+07	5671300	NA	NA
sp|P29692	Isoform 2 c	EEF1D	71 kDa	4167400	7585000	6264400	NA	NA
sp|Q07020	Isoform 2 c	RPL18	18 kDa	0	8848000	21900000	NA	NA
sp|Q6ZVM	Isoform 5 c	TOM1L2	53 kDa	5523400	575660	482740	NA	NA
sp|Q13263	Transcripti	TRIM28	89 kDa	8383500	16500000	21500000	NA	NA
sp|O94850	Dendrin	DDN	76 kDa	0	0	13800000	NA	NA
sp|Q5T4S7	Isoform 2 c	UBR4	576 kDa	729820	897060	276480	NA	NA
sp|Q86UX2	Isoform 2 c	ITIH5	77 kDa	4212600	8193200	4154300	NA	NA
sp|P30085	UMP-CMP	CMPK1	22 kDa	8064300	12700000	10800000	NA	NA
sp|Q96L91	Isoform 3 c	EP400	336 kDa	0	0	0	NA	NA
sp|Q9NU2	Midasin	MDN1	633 kDa	0	0	0	NA	NA
sp|Q2LD37	Isoform 4 c	KIAA1109	555 kDa	0	0	0	NA	NA
sp|P11940	Isoform 2 c	PABPC1	61 kDa	0	8370800	7995400	NA	NA
sp|P51659	Isoform 2 c	HSD17B4	83 kDa	14400000	3.00E+07	25100000	NA	NA
sp|P78527	Isoform 2 c	PRKDC	466 kDa	0	1583200	0	NA	NA
sp|O00401	Neural Wis	WASL	55 kDa	817000	1882800	0	NA	NA

sp|Q5QGS(	Protein KIA	KIAA2022	168 kDa	0	0	0	NA	NA
sp|P09960	Isoform 2 c	LTA4H	60 kDa	17800000	13500000	15700000	NA	NA
sp|P20711	Isoform 2 c	DDC	37 kDa	8370200	8172400	7780000	NA	NA
tr|Q5STX8	Allograft in	AIF1	18 kDa	3960100	0	3150300	NA	NA
sp|P49023	Paxillin	PXN	65 kDa	11100000	18600000	1.40E+07	NA	NA
sp|Q16851	Isoform 2 c	UGP2	56 kDa	12800000	7347700	5044500	NA	NA
tr|B1ALD9	Periostin	POSTN	90 kDa	0	0	1165300	NA	NA
sp|P0DME(	Protein SET	SETSIP	35 kDa	4447300	5393600	0	NA	NA
sp|P55084	Trifunction	HADHB	51 kDa	0	18500000	7922300	NA	NA
sp|Q96L96	Alpha-prot	ALPK3	201 kDa	0	0	0	NA	NA
tr|Q5W0X3	Peptidyl-pr	FKBP1A	11 kDa	0	0	9956800	NA	NA
sp|Q16787	Laminin sul	LAMA3	367 kDa	19800000	26300000	23300000	NA	NA
sp|O14617	Isoform 5 c	AP3D1	137 kDa	1849400	6356700	1936500	NA	NA
sp|Q9NZM	Isoform 2 c	MYOF	230 kDa	4772600	16600000	4397000	NA	NA
sp|Q15233	Non-POU d	NONO	54 kDa	5121300	8395300	7628300	NA	NA
sp|Q86TX2	Acyl-coenz	ACOT1	46 kDa	14200000	11400000	12100000	NA	NA
sp|P09622	Isoform 2 c	DLD	44 kDa	5139100	18600000	0	NA	NA
sp|Q9NQX	Isoform 2 c	GPHN	83 kDa	3879800	0	0	NA	NA
sp|P16615	Sarcoplasm	ATP2A2	115 kDa	5842300	1691500	2937000	NA	NA
tr|E7EVG6	Peroxisome	PPRC1	149 kDa	0	0	0	NA	NA
sp|P22531	Small prolir	SPRR2E	8 kDa	0	0	0	NA	NA
sp|P19971	Thymidine	TYMP	50 kDa	1119200	6937900	1356700	NA	NA
sp|Q9P0K7	Isoform 4 c	RAI14	107 kDa	1665800	6166600	5143400	NA	NA
sp|P35749	Isoform 2 c	MYH11	228 kDa	3.76E+08	5.79E+08	5.00E+08	NA	NA
sp|Q9H0W	Isoform 2 c	C11orf54	33 kDa	0	0	0	NA	NA
tr|H0Y9Y3|	Synaptopo	SYNPO2	125 kDa	0	14400000	0	NA	NA
sp|Q9BXM	Periaxin	PRX	155 kDa	0	0	0	NA	NA
tr|H3BPE7	RNA-bindir	FUS	53 kDa	12700000	19300000	10800000	NA	NA
sp|P19838	Isoform 2 c	NFKB1	105 kDa	0	0	0	NA	NA
sp|Q16610	Isoform 2 c	ECM1	46 kDa	0	0	0	NA	NA
sp|O43399	Isoform 4 c	TPD52L2	21 kDa	3865400	5019300	3375100	NA	NA
sp|Q96IZ0	PRKC apop	PAWR	37 kDa	5169800	2838800	0	NA	NA
sp|P25786	Isoform Lo	PSMA1	30 kDa	0	0	0	NA	NA
sp|Q9BQG	Isoform 2 c	MYBBP1A	149 kDa	0	0	0	NA	NA
sp|Q9H8L6	Multimerin	MMRN2	104 kDa	5986500	0	3481500	NA	NA
sp|Q8NDA	Hemicentir	HMCN2	543 kDa	0	0	0	NA	NA
sp|Q92841	Probable A	DDX17	80 kDa	13600000	14300000	11700000	NA	NA
sp|P02787	Serotransfe	TF	77 kDa	0	0	5225300	NA	NA
sp|P40121	Isoform 2 c	CAPG	37 kDa	6985900	9043100	6805800	NA	NA
sp|P62847	40S ribosor	RPS24	15 kDa	1800600	2045000	0	NA	NA
tr|J3KTF8|	Rho GDP-d	ARHGDIA	22 kDa	11300000	18500000	0	NA	NA
tr|H0YMM	Papilin (Fra	PAPLN	33 kDa	489610	467720	359120	NA	NA
tr|A0A0A0	Protein ND	NDRG2	40 kDa	0	9032500	0	NA	NA
sp|Q13510	Acid ceram	ASAH1	45 kDa	0	0	0	NA	NA
sp|Q12906	Isoform 2 c	ILF3	76 kDa	3555000	5836500	6001700	NA	NA
sp|Q9C098	Serine/thre	DCLK3	74 kDa	0	0	0	NA	NA
sp|P34913	Bifunctiona	EPHX2	63 kDa	5195300	0	5369500	NA	NA

sp|Q5VT97	Rho GTPas	SYDE2	133 kDa	0	0	0	NA	NA
tr|C9J406|	MIC compl	IMMT	73 kDa	4367400	10100000	4398100	NA	NA
sp|P55209	Isoform 2 c	NAP1L1	43 kDa	5063000	5356800	4902200	NA	NA
sp|P38117	Electron tr	ETFB	28 kDa	21700000	24200000	21600000	NA	NA
sp|P29508	Serpin B3	SERPINB3	45 kDa	0	0	0	NA	NA
sp|P10155	Isoform 4 c	TROVE2	60 kDa	2948300	6519500	5414100	NA	NA
sp|P06737	Isoform 2 c	PYGL	93 kDa	488390	9355600	7558700	NA	NA
sp|Q9BUT1	Isoform 2 c	BDH2	19 kDa	1080600	765130	627410	NA	NA
tr|K7EQ16	UV excisior	RAD23A	17 kDa	466030	294860	0	NA	NA
sp|P13688	Isoform 10	CEACAM1	51 kDa	4196600	7983400	0	NA	NA
sp|Q12967	Isoform 2 c	RALGDS	105 kDa	0	0	0	NA	NA
sp|Q14152	Eukaryotic	EIF3A	167 kDa	0	1865000	0	NA	NA
sp|O60749	Sorting nex	SNX2	58 kDa	4903700	7543600	5957400	NA	NA
sp|Q15847	Adipogene	ADIRF	8 kDa	0	0	0	NA	NA
sp|Q6UB9	Ankyrin rep	ANKRD11	298 kDa	0	0	0	NA	NA
sp|Q13228	Selenium-b	SELENBP1	52 kDa	0	0	0	NA	NA
sp|Q7Z7H	Isoform 2 c	TMED4	24 kDa	0	0	0	NA	NA
sp|Q6XQN	Isoform 2 c	NAPRT	60 kDa	2767600	932760	768290	NA	NA
sp|Q9ULV	Isoform 2 c	CORO1C	54 kDa	3784100	6251600	4918900	NA	NA
sp|P62195	26S protea	PSMC5	46 kDa	0	0	0	NA	NA
sp|Q8TCD	Isoform 2 c	NT5C	13 kDa	0	875220	721950	NA	NA
sp|Q5H9U	Probable A	DDX60L	198 kDa	0	0	0	NA	NA
sp|A7E2Y1	Myosin-7B	MYH7B	221 kDa	13800000	0	2.00E+07	NA	NA
sp|Q16775	Hydroxyac	HAGH	34 kDa	0	0	0	NA	NA
sp|O43852	Isoform 3 c	CALU	38 kDa	0	0	8883000	NA	NA
sp|P14868	Isoform 2 c	DARS	46 kDa	7765700	8031500	6817100	NA	NA
sp|Q9BPX5	Actin-relat	ARPC5L	17 kDa	0	916890	0	NA	NA
sp|Q9H4A	Aminopept	RNPEP	73 kDa	0	0	4459600	NA	NA
sp|P18084	Integrin be	ITGB5	88 kDa	0	5914000	0	NA	NA
sp|P21964	Catechol O	COMT	30 kDa	1005300	0	2388000	NA	NA
sp|Q5U651	Ras-interac	RASIP1	103 kDa	0	0	0	NA	NA
sp|O75340	Isoform 2 c	PDCD6	22 kDa	2683000	0	2803900	NA	NA
sp|P21810	Biglycan	BGN	42 kDa	2365700	2.00E+07	3533300	NA	NA
sp|P51608	Isoform B c	MECP2	53 kDa	0	0	0	NA	NA
sp|Q96QR	Transcripti	PURB	33 kDa	0	4275600	2228700	NA	NA
tr|H0Y8X4	2'-deoxynu	DNPH1	26 kDa	6058000	0	0	NA	NA
sp|Q5T0Z8	Uncharact	C6orf132	124 kDa	0	0	0	NA	NA
sp|Q12802	A-kinase a	AKAP13	308 kDa	0	3003200	0	NA	NA
sp|P08603	Compleme	CFH	139 kDa	0	0	0	NA	NA
sp|Q92734	Isoform 2 c	TFG	43 kDa	0	0	0	NA	NA
sp|Q86WA	Valacyclovi	BPHL	33 kDa	4848600	3383700	3335200	NA	NA
sp|Q14980	Isoform Nu	NUMA1	201 kDa	603300	603550	437050	NA	NA
sp|P46776	60S riboso	RPL27A	17 kDa	7428800	11800000	8733900	NA	NA
sp|Q96AP7	Endothelial	ESAM	41 kDa	6424800	8395900	0	NA	NA
sp|P04196	Histidine-ri	HRG	60 kDa	1283300	1496000	1611400	NA	NA
sp|Q2M3C	Isoform 2 c	C15orf27	54 kDa	373750	459190	0	NA	NA
sp|P28482	Mitogen-a	MAPK1	41 kDa	4488900	9105900	9968300	NA	NA

sp|O95571	Persulfide (	ETHE1	28 kDa	3430600	5952600	0	NA	NA
sp|P35998	Isoform 2 c	PSMC2	33 kDa	5782400	0	17700000	NA	NA
sp|P35606	Isoform 2 c	COPB2	99 kDa	0	3278700	5782600	NA	NA
sp|A7MCY(	TANK-bindi	TBKBP1	68 kDa	0	0	0	NA	NA
tr|F2Z2V0|	Copine-1 (F	CPNE1	59 kDa	417660	0	0	NA	NA
sp|P29992	Guanine nu	GNA11	42 kDa	6137300	8522100	6266100	NA	NA
sp|P46937	Isoform 9 c	YAP1	55 kDa	0	0	0	NA	NA
sp|O94762	ATP-depen	RECQL5	109 kDa	0	0	0	NA	NA
sp|P54578	Ubiquitin c	USP14	56 kDa	0	920770	1691100	NA	NA
sp|Q9BTV4	Transmeml	TMEM43	45 kDa	0	3092700	5837100	NA	NA
sp|Q9HCM	Fibrosin-1-l	FBRSL1	111 kDa	0	0	0	NA	NA
sp|Q04837	Single-strar	SSBP1	17 kDa	0	0	0	NA	NA
sp|P43307	Isoform 2 c	SSR1	29 kDa	2129400	6392900	1945200	NA	NA
sp|Q9UKX3	Myosin-13	MYH13	224 kDa	13800000	0	2.00E+07	NA	NA
sp|Q9UQ8(	Isoform 2 c	PA2G4	38 kDa	3995500	6482600	5565700	NA	NA
sp|P27635	60S ribosor	RPL10	25 kDa	0	7854800	1768000	NA	NA
sp|Q15642	Cdc42-inte	TRIP10	68 kDa	450310	0	0	NA	NA
sp|P60903	Protein S1(	S100A10	11 kDa	0	0	0	NA	NA
sp|P62995	Transforme	TRA2B	34 kDa	0	0	0	NA	NA
sp|Q10589	Isoform 2 c	BST2	18 kDa	5730400	12100000	11200000	NA	NA
sp|O95886	Disks large-	DLGAP3	106 kDa	7066200	0	0	NA	NA
sp|P54819	Isoform 6 c	AK2	22 kDa	0	0	0	NA	NA
sp|Q15436	Protein trai	SEC23A	86 kDa	0	0	0	NA	NA
sp|Q15654	Thyroid rec	TRIP6	50 kDa	0	0	8433700	NA	NA
sp|Q14257	Isoform 2 c	RCN2	39 kDa	0	2949000	2210300	NA	NA
sp|Q15075	Early endos	EEA1	162 kDa	0	0	0	NA	NA
sp|Q92896	Isoform 2 c	GLG1	137 kDa	0	0	0	NA	NA
sp|P48637	Glutathion	GSS	52 kDa	0	0	0	NA	NA
sp|P01112	GTPase HR	HRAS	21 kDa	0	0	0	NA	NA
tr|A0A087	Sorting nex	SNX12	20 kDa	0	0	8125200	NA	NA
sp|Q92833	Protein Jun	JARID2	139 kDa	0	0	0	NA	NA
tr|E9PM76	Sulfotransf	SULT1A2	24 kDa	8229900	11100000	11300000	NA	NA
sp|P80723	Brain acid s	BASP1	23 kDa	0	0	0	NA	NA
sp|Q99729	Isoform 2 c	HNRNPAB	36 kDa	0	0	3992700	NA	NA
sp|P01023	Alpha-2-ma	A2M	163 kDa	1375400	2168200	0	NA	NA
sp|P62826	GTP-bindin	RAN	24 kDa	1244300	0	0	NA	NA
sp|Q15185	Isoform 3 c	PTGES3	15 kDa	0	0	0	NA	NA
sp|P11498	Pyruvate ca	PC	130 kDa	0	692790	724660	NA	NA
sp|Q16643	Isoform 2 c	DBN1	72 kDa	0	0	0	NA	NA
tr|F8VVD5	2-methoxy-	COQ5	10 kDa	0	0	0	NA	NA
sp|P08237	Isoform 2 c	PFKM	82 kDa	0	1729800	677550	NA	NA
sp|Q96AJ9	Isoform 1 c	VTI1A	23 kDa	2080400	0	5218900	NA	NA
sp|P61457	Pterin-4-alp	PCBD1	12 kDa	0	0	0	NA	NA
sp|Q9BS26	Endoplasm	ERP44	47 kDa	0	3789100	2628000	NA	NA
sp|Q14258	E3 ubiquitir	TRIM25	71 kDa	0	0	0	NA	NA
sp|P16219	Short-chair	ACADS	44 kDa	0	1136200	0	NA	NA
sp|P36871	Phosphoglu	PGM1	61 kDa	1553400	6442100	0	NA	NA

tr|H0YIZ1|	Epididymal	NPC2	19 kDa	0	0	0	NA	NA
sp|Q08722	Isoform OA	CD47	32 kDa	9587100	0	11300000	NA	NA
sp|P26196	Probable A	DDX6	54 kDa	0	4680100	0	NA	NA
sp|Q5T7P3	Late cornifi	LCE1B	12 kDa	0	0	0	NA	NA
sp|A4D1E1	Zinc finger	ZNF804B	153 kDa	0	0	0	NA	NA
sp|P62857	40S ribosor	RPS28	8 kDa	0	0	0	NA	NA
sp|A4D0S4	Isoform 3 c	LAMB4	189 kDa	0	0	0	NA	NA
sp|Q9P219	Protein Da	CCDC88C	228 kDa	4015600	0	0	NA	NA
sp|Q9NPH	Inositol-3-p	ISYNA1	61 kDa	1095600	6601300	928960	NA	NA
sp|P0DJI9|	Serum amy	SAA2	14 kDa	7210200	8577900	6338700	NA	NA
sp|P18859	Isoform 2 c	ATP5J	13 kDa	0	0	0	NA	NA
sp|Q8N16	Isoform 2 c	CCAR2	103 kDa	0	0	0	NA	NA
sp|P10398	Serine/thre	ARAF	68 kDa	0	0	0	NA	NA
sp|P17844	Probable A	DDX5	69 kDa	0	7890100	1304600	NA	NA
sp|P36507	Dual specif	MAP2K2	44 kDa	0	519470	0	NA	NA
sp|P11413	Isoform 3 c	G6PD	62 kDa	2555200	1699700	0	NA	NA
sp|Q9Y2Q	Isoform 2 c	GSTK1	32 kDa	0	0	0	NA	NA
sp|Q92973	Isoform 2 c	TNPO1	101 kDa	2859400	0	3748500	NA	NA
sp|Q9BUP	Isoform 2 c	EFHD1	16 kDa	1097200	1635400	976500	NA	NA
sp|P35916	Isoform 2 c	FLT4	146 kDa	0	0	3246300	NA	NA
sp|Q9H211	DNA replica	CDT1	60 kDa	0	0	0	NA	NA
sp|P83731	60S ribosor	RPL24	18 kDa	0	0	0	NA	NA
sp|P11217	Glycogen p	PYGM	97 kDa	0	8651500	7013700	NA	NA
sp|P62266	40S ribosor	RPS23	16 kDa	0	0	0	NA	NA
sp|Q93009	Isoform 3 c	USP7	126 kDa	6224700	0	0	NA	NA
sp|Q5TFQ	Signal-regu	SIRPB1	43 kDa	0	0	0	NA	NA
sp|P19961	Alpha-amyl	AMY2B	58 kDa	0	0	0	NA	NA
sp|O76041	Nebulette	NEBL	116 kDa	35500000	4.60E+07	35400000	NA	NA
sp|P30046	D-dopachr	DDT	13 kDa	0	0	0	NA	NA
sp|O14633	Late cornifi	LCE2B	11 kDa	0	0	0	NA	NA
sp|Q16795	NADH deh	NDUFA9	43 kDa	0	913570	0	NA	NA
sp|P32754	Isoform 2 c	HPD	40 kDa	0	14900000	0	NA	NA
sp|Q9Y5Z4	Isoform 2 c	HEBP2	21 kDa	0	0	0	NA	NA
sp|O95433	Isoform 2 c	AHSA1	32 kDa	0	0	0	NA	NA
sp|Q13596	Isoform 1A	SNX1	52 kDa	1833400	6491500	6464300	NA	NA
sp|Q14677	Isoform 3 c	CLINT1	70 kDa	0	0	1966700	NA	NA
sp|Q92820	Gamma-gl	GGH	36 kDa	0	0	0	NA	NA
sp|O94925	Isoform 3 c	GLS	65 kDa	0	0	0	NA	NA
sp|Q13162	Peroxiredo	PRDX4	31 kDa	3158800	7057700	0	NA	NA
sp|Q16629	Isoform 2 c	SRSF7	16 kDa	0	18300000	5154500	NA	NA
sp|P62244	40S ribosor	RPS15A	15 kDa	0	3883200	2910400	NA	NA
sp|Q13526	Peptidyl-pr	PIN1	18 kDa	0	0	0	NA	NA
sp|Q9BTT0	Acidic leuci	ANP32E	31 kDa	0	0	0	NA	NA
sp|Q9NVJ2	ADP-ribosy	ARL8B	22 kDa	0	0	0	NA	NA
sp|Q13103	Secreted pl	SPP2	24 kDa	0	0	0	NA	NA
sp|O94760	N(G),N(G)-	DDAH1	31 kDa	0	0	0	NA	NA
sp|P14406	Cytochrom	COX7A2	9 kDa	0	0	0	NA	NA

sp|P25789	Proteasom	PSMA4	29 kDa	0	0	0	NA	NA	
sp|Q15828	Cystatin-M	CST6	17 kDa	0	0	0	NA	NA	
sp|Q13643	Four and a	FHL3	31 kDa	1452500	0	0	NA	NA	
sp|O14594	Neurocan c	NCAN	143 kDa	0	0	0	NA	NA	
sp|P41218	Myeloid ce	MNDA	46 kDa	0	0	0	NA	NA	
sp|O00231	Isoform 2 c	PSMD11	48 kDa	0	0	0	NA	NA	
sp|P36543	Isoform 2 c	ATP6V1E1	24 kDa	0	0	0	NA	NA	
sp|Q13057	Isoform 2 c	COASY	65 kDa	2196100	4915600	1340800	NA	NA	
tr|E7EPV7|	Alpha-synu	SNCA	12 kDa	0	0	0	NA	NA	
sp|Q8WXE	Isoform 2 c	CASKIN2	118 kDa	0	0	0	NA	NA	
sp|O75348	V-type prot	ATP6V1G1	14 kDa	0	4065600	0	NA	NA	
tr|H0YHD8	Cysteine-ri	CRIP2	11 kDa	6.60E+07	82800000	5466800	NA	NA	
sp|P30511	Isoform 3 c	HLA-F	50 kDa	2.10E+07	0	0	NA	NA	
sp|Q9UBC5	Unconvent	MYO1A	118 kDa	0	0	0	NA	NA	
sp|P12883	Myosin-7	MYH7	223 kDa	0	0	0	NA	NA	
sp|Q86YQ8	Copine-8	CPNE8	63 kDa	2097100	2551300	2249500	NA	NA	
sp|Q9NRX4	14 kDa pho	PHPT1	14 kDa	0	0	0	NA	NA	
sp|Q6IAA8	Ragulator c	LAMTOR1	18 kDa	0	0	0	NA	NA	
sp|O75594	Peptidogly	PGLYRP1	22 kDa	0	0	0	NA	NA	
sp|P47985	Cytochrom	UQCRFS1	30 kDa	0	7213800	330210	NA	NA	
sp|Q9P0L0	Isoform 2 c	VAPA	33 kDa	0	0	0	NA	NA	
sp|O14979	Isoform 2 c	HNRNPDL	34 kDa	4191900	8105700	4472400	NA	NA	
sp|P19652	Alpha-1-aci	ORM2	24 kDa	0	0	0	NA	NA	
sp|Q13347	Eukaryotic	EIF3I	37 kDa	0	3557600	0	NA	NA	
sp|P33241	Isoform 3 c	LSP1	50 kDa	0	0	0	NA	NA	
sp|P30049	ATP syntha	ATP5D	17 kDa	0	0	0	NA	NA	
sp|Q9BT88	Synaptotag	SYT11	48 kDa	0	0	0	NA	NA	
tr|A0A087	Ig mu chair	IGHM	64 kDa	0	0	0	NA	NA	
sp|P30039	Isoform 2 c	PBLD	31 kDa	0	0	0	NA	NA	
sp|P27482	Calmodulin	CALML3	17 kDa	0	0	0	NA	NA	
sp|Q9Y2B0	Protein car	CNPY2	21 kDa	0	0	0	NA	NA	
sp|P04280	Basic saliva	PRB1	39 kDa	0	0	0	NA	NA	
sp|O75964	ATP syntha	ATP5L	11 kDa	0	0	0	NA	NA	
sp|Q14576	ELAV-like p	ELAVL3	40 kDa	0	0	0	NA	NA	
sp|Q14508	WAP four-c	WFDC2	13 kDa	0	0	0	NA	NA	
sp|Q15758	Neutral am	SLC1A5	57 kDa	0	0	0	NA	NA	
sp|P08590	Myosin ligh	MYL3	22 kDa	36800000	59200000	55700000	NA	NA	
tr|H3BUN4	Nucleolar p	NOL3	22 kDa	204370	145900	0	NA	NA	
sp|Q8IWE2	Isoform 2 c	FAM114A1	40 kDa	0	0	0	NA	NA	
sp|P51153	Ras-related	RAB13	23 kDa	10300000	0	11500000	NA	NA	
tr|Q60FE5|	Filamin A	FLNA	278 kDa	NA	NA	NA		8.34E+08	1.80E+09
sp|P60709	Actin, cyto	ACTB	42 kDa	NA	NA	NA		9.02E+09	1.64E+10
sp|Q71U36	Tubulin alp	TUBA1A	50 kDa	NA	NA	NA		1.08E+09	1.71E+09
sp|P02671	Isoform 2 c	FGA	70 kDa	NA	NA	NA		4.22E+08	5.26E+08
sp|Q00610	Isoform 2 c	CLTC	188 kDa	NA	NA	NA		1.24E+08	2.37E+08
tr|Q5T0I0|	Gelsolin (Fr	GSN	29 kDa	NA	NA	NA		3.56E+08	5.96E+08
sp|P14618	Pyruvate ki	PKM	58 kDa	NA	NA	NA		2.28E+08	3.70E+08

tr|H0Y998	Collagen al	COL4A5	12 kDa	NA	NA	NA	1.58E+08	8.73E+08
sp|Q8WZ4	Isoform 12	TTN	3994 kDa	NA	NA	NA	69900	0
sp|Q07157	Tight juncti	TJP1	195 kDa	NA	NA	NA	59200000	2.06E+08
tr|A0A024I	EH domain	EHD1	62 kDa	NA	NA	NA	86600000	1.97E+08
tr|H7BXV5	Collagen al	COL18A1	71 kDa	NA	NA	NA	94700000	1.51E+08
sp|Q96AC1	Isoform 3 c	FERMT2	79 kDa	NA	NA	NA	47500000	1.05E+08
sp|P68104	Isoform 2 c	EEF1A1	48 kDa	NA	NA	NA	2.55E+08	4.44E+08
sp|O00159	Isoform 3 c	MYO1C	120 kDa	NA	NA	NA	5.90E+07	1.50E+08
sp|HBB_HU	sp|HBB_HU	HBB	16 kDa	NA	NA	NA	1.53E+08	2.82E+08
sp|P04075	Fructose-bi	ALDOA	39 kDa	NA	NA	NA	90900000	1.42E+08
sp|P35580	Isoform 3 c	MYH10	231 kDa	NA	NA	NA	1.38E+08	2.65E+08
sp|Q05682	Isoform 3 c	CALD1	64 kDa	NA	NA	NA	73200000	1.96E+08
sp|Q13733	Sodium/po	ATP1A4	114 kDa	NA	NA	NA	35300000	1.17E+08
sp|P08238	Heat shock	HSP90AB1	83 kDa	NA	NA	NA	2.39E+08	5.07E+08
sp|O75369	Isoform 2 c	FLNB	276 kDa	NA	NA	NA	2.90E+07	69500000
sp|P10909	Isoform 2 c	CLU	58 kDa	NA	NA	NA	81300000	1.25E+08
sp|P06756	Isoform 3 c	ITGAV	111 kDa	NA	NA	NA	19900000	27800000
sp|P23528	Cofilin-1	CFL1	19 kDa	NA	NA	NA	1.20E+08	2.31E+08
sp|P52272	Heterogene	HNRNPM	78 kDa	NA	NA	NA	5129200	17200000
sp|P31946	14-3-3 prot	YWHAB	28 kDa	NA	NA	NA	74400000	1.35E+08
sp|P00918	Carbonic ar	CA2	29 kDa	NA	NA	NA	81900000	1.22E+08
sp|P02794	Ferritin hea	FTH1	21 kDa	NA	NA	NA	3518800	7320400
sp|P84077	ADP-ribosy	ARF1	21 kDa	NA	NA	NA	9067500	13800000
sp|P23142	Isoform C c	FBLN1	74 kDa	NA	NA	NA	12700000	25300000
tr|F5H491|	Ras-related	RAP1B	10 kDa	NA	NA	NA	28500000	32400000
sp|Q27J81	Isoform 2 c	INF2	135 kDa	NA	NA	NA	7443300	11300000
sp|Q8IZC6	Collagen al	COL27A1	187 kDa	NA	NA	NA	0	0
sp|Q15599	Na(+)/H(+)	SLC9A3R2	37 kDa	NA	NA	NA	20500000	37700000
sp|P00387	Isoform 3 c	CYB5R3	38 kDa	NA	NA	NA	3.40E+07	72900000
sp|Q15084	Isoform 5 c	PDIA6	53 kDa	NA	NA	NA	8156000	3.50E+07
sp|P50395	Isoform 2 c	GDI2	46 kDa	NA	NA	NA	4.60E+07	67500000
sp|P04040	Catalase	CAT	60 kDa	NA	NA	NA	8366200	23700000
sp|Q9UJU6	Isoform 2 c	DBNL	48 kDa	NA	NA	NA	10200000	30400000
sp|Q68CZ2	Tensin-3	TNS3	155 kDa	NA	NA	NA	17800000	41700000
sp|Q00839	Isoform Sh	HNRNPU	89 kDa	NA	NA	NA	28900000	57400000
tr|B1AK88	Capping pr	CAPZB	34 kDa	NA	NA	NA	27300000	39700000
sp|Q86VP6	Isoform 2 c	CAND1	118 kDa	NA	NA	NA	3982700	13600000
sp|O94856	Isoform 12	NFASC	120 kDa	NA	NA	NA	7880400	14400000
sp|P30044	Isoform Cy	PRDX5	17 kDa	NA	NA	NA	28500000	2.80E+07
sp|Q03252	Lamin-B2	LMNB2	68 kDa	NA	NA	NA	7135100	13700000
sp|Q15746	Isoform De	MYLK	211 kDa	NA	NA	NA	2614300	1.40E+07
sp|Q07092	Isoform 2 c	COL16A1	158 kDa	NA	NA	NA	0	0
sp|P51991	Isoform 2 c	HNRNPA3	37 kDa	NA	NA	NA	21800000	60600000
sp|P12235	ADP/ATP tr	SLC25A4	33 kDa	NA	NA	NA	31400000	47400000
sp|Q13561	Isoform 2 c	DCTN2	45 kDa	NA	NA	NA	1132200	19800000
sp|P06748	Nucleopho	NPM1	33 kDa	NA	NA	NA	0	14300000
sp|P04899	Isoform 3 c	GNAI2	36 kDa	NA	NA	NA	9461900	14100000

sp|Q8WUM	Isoform 2 c	PDCD6IP	97 kDa	NA	NA	NA	11400000	1.80E+07
sp|Q03164	Isoform 3 c	KMT2A	432 kDa	NA	NA	NA	0	0
sp|Q8NFD5	Isoform 2 c	ARID1B	238 kDa	NA	NA	NA	0	0
tr|D6W5N(	Membrane	MAGI2	139 kDa	NA	NA	NA	8144300	23200000
sp|Q8IVF2	Protein AH	AHNAK2	617 kDa	NA	NA	NA	0	0
sp|Q86VF2	Isoform 5 c	IGFN1	384 kDa	NA	NA	NA	0	0
sp|P0CG05	Ig lambda-2	IGLC2	11 kDa	NA	NA	NA	0	49900000
sp|P63000	Ras-related	RAC1	21 kDa	NA	NA	NA	8335200	28500000
sp|Q8IZP2	Putative pr	ST13P4	27 kDa	NA	NA	NA	14600000	30600000
sp|P49748	Isoform 2 c	ACADVL	68 kDa	NA	NA	NA	11700000	26100000
sp|P31150	Rab GDP di	GDI1	51 kDa	NA	NA	NA	2.60E+07	46100000
sp|P51659	Peroxisoma	HSD17B4	80 kDa	NA	NA	NA	5230900	19400000
sp|O75955	Flotillin-1	FLOT1	47 kDa	NA	NA	NA	1915100	4985200
sp|P17813	Isoform Sh	ENG	68 kDa	NA	NA	NA	2404600	4818200
tr|F8VUA6	60S ribosor	RPL18	15 kDa	NA	NA	NA	5499600	22900000
sp|O95428	Isoform 6 c	PAPLN	135 kDa	NA	NA	NA	0	2760200
sp|Q13838	Isoform 2 c	DDX39B	51 kDa	NA	NA	NA	10800000	24300000
sp|Q9NR12	Isoform 2 c	PDLIM7	47 kDa	NA	NA	NA	5471600	6448400
sp|Q15063	Isoform 2 c	POSTN	87 kDa	NA	NA	NA	791360	1258100
sp|P63092	Isoform Gn	GNAS	44 kDa	NA	NA	NA	8553200	37800000
sp|P11940	Polyadenyl	PABPC1	71 kDa	NA	NA	NA	0	9566800
sp|P22061	Isoform 2 c	PCMT1	25 kDa	NA	NA	NA	7866000	27500000
sp|Q9NZM	Isoform 6 c	MYOF	233 kDa	NA	NA	NA	1423900	9550100
sp|O00429	Isoform 5 c	DNM1L	79 kDa	NA	NA	NA	2006400	2175100
sp|P13804	Isoform 2 c	ETFA	30 kDa	NA	NA	NA	11100000	6288300
tr|D6RGG3	Collagen al	COL12A1	333 kDa	NA	NA	NA	0	3.55E+08
sp|P60842	Eukaryotic	EIF4A1	46 kDa	NA	NA	NA	33700000	60300000
sp|Q86UX2	Inter-alpha	ITIH5	105 kDa	NA	NA	NA	2649800	5199500
sp|P16615	Isoform 2 c	ATP2A2	110 kDa	NA	NA	NA	1619200	2154000
sp|P59666	Neutrophil	DEFA3	10 kDa	NA	NA	NA	17400000	0
tr|E7EUC7|	UTP--gluco	UGP2	58 kDa	NA	NA	NA	5257200	6138200
sp|O15417	Trinucleoti	TNRC18	315 kDa	NA	NA	NA	0	0
sp|Q8TEW	Isoform 2 c	PARD3B	126 kDa	NA	NA	NA	2776400	12300000
sp|P20711	Aromatic-L	DDC	54 kDa	NA	NA	NA	8044600	8015100
sp|Q13642	Isoform 5 c	FHL1	34 kDa	NA	NA	NA	10900000	24800000
sp|Q9P2E9	Isoform 1 c	RRBP1	152 kDa	NA	NA	NA	4560000	4287600
tr|J3QQX2	Rho GDP-d	ARHGDIA	26 kDa	NA	NA	NA	0	0
sp|P09960	Leukotrien	LTA4H	69 kDa	NA	NA	NA	7151000	9949500
sp|P48643	Isoform 2 c	CCT5	50 kDa	NA	NA	NA	3881200	5611100
sp|P26640	Valine--tRN	VARS	140 kDa	NA	NA	NA	0	0
tr|E9PEX6|	Dihydrolipo	DLD	52 kDa	NA	NA	NA	2167400	13200000
sp|Q8TAA3	Isoform 2 c	PSMA8	24 kDa	NA	NA	NA	0	4166500
tr|J3KPD9|	Nucleoside	NME2	22 kDa	NA	NA	NA	5248400	20200000
sp|P28799	Granulins	GRN	64 kDa	NA	NA	NA	5320700	5947100
sp|Q14103	Isoform 2 c	HNRNPD	36 kDa	NA	NA	NA	3372500	38500000
sp|P49419	Isoform 4 c	ALDH7A1	54 kDa	NA	NA	NA	2571200	5386600
sp|O60506	Heterogene	SYNCRIP	70 kDa	NA	NA	NA	8255200	12100000

sp|Q92945	Far upstrea	KHSRP	73 kDa	NA	NA	NA	0	0
sp|Q6ZVM	TOM1-like	TOM1L2	56 kDa	NA	NA	NA	515670	0
sp|Q01105	Isoform 3 c	SET	31 kDa	NA	NA	NA	4597200	7229600
sp|Q9P0K7	Isoform 2 c	RAI14	110 kDa	NA	NA	NA	2785700	4551000
sp|P54819	Isoform 2 c	AK2	26 kDa	NA	NA	NA	794200	884850
sp|O43390	Isoform 2 c	HNRNPR	71 kDa	NA	NA	NA	6730900	13100000
tr|D6R9L0|	Guanine nu	GNB2L1	33 kDa	NA	NA	NA	1995700	0
tr|C9JZR2|	Catenin del	CTNND1	105 kDa	NA	NA	NA	0	0
sp|P07602	Prosaposin	PSAP	58 kDa	NA	NA	NA	198540	413440
sp|Q6KC79	Nipped-B-li	NIPBL	316 kDa	NA	NA	NA	0	0
sp|P35611	Isoform 3 c	ADD1	84 kDa	NA	NA	NA	4064500	11100000
sp|Q9ULV4	Isoform 3 c	CORO1C	59 kDa	NA	NA	NA	2269900	2047900
sp|P28331	Isoform 2 c	NDUFS1	81 kDa	NA	NA	NA	1424600	0
sp|P07305	Histone H1	H1F0	21 kDa	NA	NA	NA	3364200	6101400
sp|P55008	Allograft in	AIF1	17 kDa	NA	NA	NA	2084100	2728700
sp|P62942	Peptidyl-pr	FKBP1A	12 kDa	NA	NA	NA	0	0
sp|Q12906	Isoform 3 c	ILF3	83 kDa	NA	NA	NA	1868800	3905600
sp|O43399	Isoform 5 c	TPD52L2	24 kDa	NA	NA	NA	2118700	4233300
sp|Q14980	Isoform 2 c	NUMA1	237 kDa	NA	NA	NA	5581200	344410
sp|Q6KB66	Isoform 2 c	KRT80	47 kDa	NA	NA	NA	0	34700000
sp|P62873	Guanine nu	GNB1	37 kDa	NA	NA	NA	5483700	17600000
sp|P55290	Isoform 4 c	CDH13	83 kDa	NA	NA	NA	0	4034900
sp|Q9BZF9	Isoform 2 c	UACA	162 kDa	NA	NA	NA	0	1262100
sp|P38159	RNA-bindir	RBMX	42 kDa	NA	NA	NA	5363000	10200000
sp|Q13228	Isoform 4 c	SELENBP1	57 kDa	NA	NA	NA	0	0
sp|P55084	Isoform 2 c	HADHB	49 kDa	NA	NA	NA	4610900	2296000
sp|O60504	Vinexin	SORBS3	75 kDa	NA	NA	NA	0	0
sp|O95996	Isoform 3 c	APC2	244 kDa	NA	NA	NA	0	0
sp|P20591	Interferon-	MX1	76 kDa	NA	NA	NA	7942700	10900000
sp|Q13200	26S protea	PSMD2	100 kDa	NA	NA	NA	0	0
sp|Q9UMS	Isoform 4 c	SYNPO2	133 kDa	NA	NA	NA	4018400	7530900
sp|Q9Y6W	Wiskott-Alc	WASF2	54 kDa	NA	NA	NA	541940	888680
sp|Q9H0W	Ester hydro	C11orf54	35 kDa	NA	NA	NA	0	0
sp|P50914	60S riboson	RPL14	23 kDa	NA	NA	NA	3082500	7664600
sp|P34913	Isoform 2 c	EPHX2	57 kDa	NA	NA	NA	529730	0
sp|P35637	Isoform Sho	FUS	53 kDa	NA	NA	NA	4597600	6840000
sp|P38117	Isoform 2 c	ETFB	37 kDa	NA	NA	NA	9117400	18400000
sp|P09467	Fructose-1,	FBP1	37 kDa	NA	NA	NA	1647400	1091700
tr|B0QZ18	Copine-1	CPNE1	60 kDa	NA	NA	NA	0	0
sp|O15020	Spectrin be	SPTBN2	271 kDa	NA	NA	NA	2273400	2634700
sp|P02747	Compleme	C1QC	26 kDa	NA	NA	NA	0	0
sp|P62854	40S riboson	RPS26	13 kDa	NA	NA	NA	2227500	10400000
sp|P54725	Isoform 3 c	RAD23A	40 kDa	NA	NA	NA	0	0
sp|Q9BUT1	3-hydroxyb	BDH2	27 kDa	NA	NA	NA	821680	599290
sp|P53999	Activated R	SUB1	14 kDa	NA	NA	NA	0	0
sp|O75368	SH3 domai	SH3BGRL	13 kDa	NA	NA	NA	0	0
sp|P17612	cAMP-depe	PRKACA	41 kDa	NA	NA	NA	876290	3904900

sp|P68036	Isoform 3 c	UBE2L3	24 kDa	NA	NA	NA	1280100	0
sp|O75340	Programme	PDCD6	22 kDa	NA	NA	NA	1277900	3220800
sp|P35998	26S protea:	PSMC2	49 kDa	NA	NA	NA	2160000	734120
sp|Q96AB3	Isochorism	ISOC2	22 kDa	NA	NA	NA	3497700	2152500
sp|Q4ZHG4	Fibronectin	FNDC1	206 kDa	NA	NA	NA	0	0
sp|P13688	Carcinoeml	CEACAM1	58 kDa	NA	NA	NA	0	6151100
sp|Q16610	Isoform 4 c	ECM1	64 kDa	NA	NA	NA	0	0
sp|P00747	Plasminoge	PLG	91 kDa	NA	NA	NA	3045800	0
sp|Q14240	Isoform 2 c	EIF4A2	46 kDa	NA	NA	NA	30100000	53900000
sp|P62314	Small nucle	SNRPD1	13 kDa	NA	NA	NA	0	0
sp|P62995	Isoform 3 c	TRA2B	22 kDa	NA	NA	NA	1328800	3629900
sp|Q9NYC9	Dynein hea	DNAH9	512 kDa	NA	NA	NA	0	0
sp|P46937	Isoform 3 c	YAP1	49 kDa	NA	NA	NA	0	0
sp|Q13510	Isoform 2 c	ASAH1	47 kDa	NA	NA	NA	0	0
sp|P40121	Macrophag	CAPG	38 kDa	NA	NA	NA	1966200	3308400
sp|Q92841	Isoform 4 c	DDX17	73 kDa	NA	NA	NA	2939800	15300000
sp|Q9P2J5	Isoform 2 c	LARS	128 kDa	NA	NA	NA	1156900	920970
sp|P14868	Aspartate--	DARS	57 kDa	NA	NA	NA	3174800	0
sp|P62847	Isoform 2 c	RPS24	15 kDa	NA	NA	NA	1245200	0
sp|P07203	Glutathion	GPX1	22 kDa	NA	NA	NA	0	0
sp|O14787	Isoform 2 c	TNPO2	100 kDa	NA	NA	NA	0	3341000
sp|Q9NZB2	Isoform D c	FAM120A	117 kDa	NA	NA	NA	0	0
sp|Q15056	Isoform Sh	EIF4H	25 kDa	NA	NA	NA	6448900	0
sp|P48059	Isoform 2 c	LIMS1	38 kDa	NA	NA	NA	1718100	7642500
tr|M0R210	40S ribosor	RPS16	14 kDa	NA	NA	NA	0	0
sp|Q3L8U1	Chromodoi	CHD9	326 kDa	NA	NA	NA	0	0
sp|Q9ULD9	Zinc finger	ZNF608	162 kDa	NA	NA	NA	0	0
sp|Q9BUP(	EF-hand dc	EFHD1	27 kDa	NA	NA	NA	582910	523270
sp|O00560	Isoform 2 c	SDCBP	32 kDa	NA	NA	NA	333540	646190
tr|D6W648	Calmodulin	CAMSAP3	136 kDa	NA	NA	NA	0	0
sp|O95837	Guanine nu	GNA14	42 kDa	NA	NA	NA	2525000	0
sp|Q9Y2Q3	Glutathion	GSTK1	25 kDa	NA	NA	NA	0	0
sp|P49753	Acyl-coenz	ACOT2	53 kDa	NA	NA	NA	3204300	2778900
sp|P10155	Isoform 3 c	TROVE2	58 kDa	NA	NA	NA	2481200	6200000
tr|G3V126	ATPase, H+	ATP6V1H	52 kDa	NA	NA	NA	0	4015400
tr|D6RIA3|	Protein LO(	LOC285556	190 kDa	NA	NA	NA	0	0
sp|P08493	Isoform 2 c	MGP	15 kDa	NA	NA	NA	0	10600000
sp|P58107	Epiplakin	EPPK1	556 kDa	NA	NA	NA	2242600	2913100
sp|Q765P7	MTSS1-like	MTSS1L	80 kDa	NA	NA	NA	0	0
sp|Q16666	Gamma-int	IFI16	88 kDa	NA	NA	NA	0	1669800
sp|Q32MZ4	Leucine-ric	LRRFIP1	89 kDa	NA	NA	NA	0	0
sp|Q16643	Isoform 3 c	DBN1	76 kDa	NA	NA	NA	1516100	0
sp|Q9BSE5	Agmatinas	AGMAT	38 kDa	NA	NA	NA	7490100	8025800
sp|P42126	Enoyl-CoA	ECI1	33 kDa	NA	NA	NA	1397400	0
sp|P50213	Isoform 2 c	IDH3A	31 kDa	NA	NA	NA	0	0
sp|Q86UX7	Isoform 2 c	FERMT3	75 kDa	NA	NA	NA	1445900	1619600
tr|A8MUS3	60S ribosor	RPL23A	22 kDa	NA	NA	NA	0	0

sp|Q14152	Isoform 2 c	EIF3A	163 kDa	NA	NA	NA	237830	0
sp|P54098	DNA polym	POLG	140 kDa	NA	NA	NA	4925300	0
sp|A6NDB9	Paralemmi	PALM3	72 kDa	NA	NA	NA	0	0
tr|H0YL54|	LIM/home	LHX9	38 kDa	NA	NA	NA	0	0
sp|O43813	LanC-like p	LANCL1	45 kDa	NA	NA	NA	1290800	2648700
sp|Q16629	Serine/argi	SRSF7	27 kDa	NA	NA	NA	0	14200000
sp|Q71UI9	Histone H2	H2AFV	14 kDa	NA	NA	NA	1.72E+08	4.32E+08
sp|O15014	Zinc finger	ZNF609	151 kDa	NA	NA	NA	0	0
sp|P54920	Alpha-solul	NAPA	33 kDa	NA	NA	NA	3472100	4786400
sp|Q13263	Isoform 2 c	TRIM28	79 kDa	NA	NA	NA	1762200	8318200
sp|Q15569	Dual specif	TESK1	68 kDa	NA	NA	NA	0	28100000
sp|Q9BYX7	Putative be	POTEKP	42 kDa	NA	NA	NA	2.04E+09	3.42E+09
sp|P49720	Proteasom	PSMB3	23 kDa	NA	NA	NA	0	0
sp|P53618	Coatomer s	COPB1	107 kDa	NA	NA	NA	1070300	1432600
sp|O43852	Isoform 2 c	CALU	37 kDa	NA	NA	NA	0	0
sp|Q6XQN	Isoform 3 c	NAPRT	56 kDa	NA	NA	NA	1816700	1718700
sp|P22307	Isoform SC	SCP2	15 kDa	NA	NA	NA	894460	0
sp|TRFE_H	sp|TRFE_H	TRFE	77 kDa	NA	NA	NA	0	0
sp|P28070	Proteasom	PSMB4	29 kDa	NA	NA	NA	0	0
sp|Q16775	Isoform 2 c	HAGH	29 kDa	NA	NA	NA	0	0
sp|Q8TCD5	5'(3')-deox	NT5C	23 kDa	NA	NA	NA	0	0
sp|Q9BXR6	Compleme	CFHR5	64 kDa	NA	NA	NA	0	2574100
sp|O60749	Isoform 2 c	SNX2	46 kDa	NA	NA	NA	0	0
sp|P20339	Isoform 2 c	RAB5A	22 kDa	NA	NA	NA	1441500	0
sp|Q99460	Isoform 2 c	PSMD1	102 kDa	NA	NA	NA	534950	0
tr|J3KS54|	Protein flig	FLII	78 kDa	NA	NA	NA	0	0
sp|P29508	Isoform 2 c	SERPINB3	39 kDa	NA	NA	NA	0	0
sp|P48637	Isoform 2 c	GSS	40 kDa	NA	NA	NA	771790	2709500
sp|Q9NPH	Isoform 2 c	ISYNA1	47 kDa	NA	NA	NA	784290	1242200
sp|P61077	Isoform 2 c	UBE2D3	17 kDa	NA	NA	NA	0	994590
sp|O75064	DENN dom	DENND4B	164 kDa	NA	NA	NA	0	0
sp|P54578	Isoform 3 c	USP14	55 kDa	NA	NA	NA	2157600	4577100
sp|Q6NZI2	Isoform 2 c	PTRF	33 kDa	NA	NA	NA	1911300	3543200
sp|Q8N1G	Leucine-ric	LRRC47	63 kDa	NA	NA	NA	1956300	0
sp|Q96AJ9	Vesicle trar	VTI1A	25 kDa	NA	NA	NA	0	0
sp|O43583	Density-reg	DENR	22 kDa	NA	NA	NA	0	0
sp|O00203	Isoform 2 c	AP3B1	116 kDa	NA	NA	NA	1513600	0
tr|H0Y9K1|	Protein trai	SEC31A	26 kDa	NA	NA	NA	0	328380
sp|O95954	Isoform C c	FTCD	61 kDa	NA	NA	NA	1469100	1067600
sp|Q86W5	Poly(ADP-ri	PARG	111 kDa	NA	NA	NA	0	0
sp|P37840	Isoform 2-4	SNCA	11 kDa	NA	NA	NA	0	3030400
sp|P61020	Ras-related	RAB5B	24 kDa	NA	NA	NA	1554700	3114300
sp|Q9UKG	DCC-intera	APPL1	80 kDa	NA	NA	NA	0	3463500
tr|C9JQS9|	Propionyl-C	PCCB	61 kDa	NA	NA	NA	0	0
sp|Q2M1P	Kinesin-like	KIF7	151 kDa	NA	NA	NA	0	0
sp|O14979	Heterogen	HNRNPDL	46 kDa	NA	NA	NA	2611000	8427000
sp|Q5HY64	Putative pr	FAM47C	115 kDa	NA	NA	NA	0	0

sp|O14974	Isoform 4 c	PPP1R12A	109 kDa	NA	NA	NA	0	0
sp|Q99729	Heterogen	HNRNPAB	36 kDa	NA	NA	NA	0	0
sp|P45954	Short/bran	ACADSB	47 kDa	NA	NA	NA	2160400	0
sp|P11310	Isoform 2 c	ACADM	47 kDa	NA	NA	NA	3512800	2397300
sp|Q13596	Isoform 3 c	SNX1	63 kDa	NA	NA	NA	1897000	5335900
sp|Q92734	Isoform 4 c	TFG	31 kDa	NA	NA	NA	0	0
sp|P21912	Succinate c	SDHB	32 kDa	NA	NA	NA	0	1227900
sp|Q6ZMP	Thrombosp	THSD4	112 kDa	NA	NA	NA	0	0
sp|P04440	HLA class II	HLA-DPB1	29 kDa	NA	NA	NA	2708600	6564600
sp|Q58FF6	Putative he	HSP90AB4F	58 kDa	NA	NA	NA	0	39600000
sp|Q9P275	Isoform 2 c	USP36	123 kDa	NA	NA	NA	0	0
tr|H3BUX2	Cytochrom	CYB5B	16 kDa	NA	NA	NA	8303000	12600000
sp|P50453	Serpin B9	SERPINB9	42 kDa	NA	NA	NA	0	0
sp|P21926	CD9 antige	CD9	25 kDa	NA	NA	NA	0	0
sp|P35914	Isoform 2 c	HMGCL	27 kDa	NA	NA	NA	2344200	437560
sp|Q14344	Guanine nu	GNA13	44 kDa	NA	NA	NA	6302400	19600000
sp|P11413	Isoform Lo	G6PD	64 kDa	NA	NA	NA	1016500	0
sp|P41743	Protein kin	PRKCI	68 kDa	NA	NA	NA	0	0
sp|O60701	Isoform 2 c	UGDH	48 kDa	NA	NA	NA	0	0
sp|O94760	Isoform 2 c	DDAH1	20 kDa	NA	NA	NA	0	0
sp|P43307	Translocon	SSR1	32 kDa	NA	NA	NA	0	0
sp|Q96EP5	Isoform 2 c	DAZAP1	41 kDa	NA	NA	NA	0	4473900
sp|Q9UI17	Dimethylgl	DMGDH	97 kDa	NA	NA	NA	0	0
sp|A1A5D9	Isoform 2 c	CCDC64B	34 kDa	NA	NA	NA	0	0
sp|P32456	Interferon-	GBP2	67 kDa	NA	NA	NA	0	5893700
sp|K1H8_H	sp|K1H8_H	K1H8	50 kDa	NA	NA	NA	0	0
sp|P35612	Isoform 9 c	ADD2	64 kDa	NA	NA	NA	0	0
sp|P63220	40S ribosor	RPS21	9 kDa	NA	NA	NA	0	0
tr|A0A087	Endophilin-	SH3GLB1	44 kDa	NA	NA	NA	534840	1286400
tr|D6RGV5	Cytochrom	COX7A2	12 kDa	NA	NA	NA	0	0
sp|P55145	Mesencepł	MANF	21 kDa	NA	NA	NA	5751500	0
sp|Q14194	Dihydropyr	CRMP1	62 kDa	NA	NA	NA	10300000	35200000
sp|O14558	Heat shock	HSPB6	17 kDa	NA	NA	NA	0	0
sp|Q92764	Keratin, typ	KRT35	50 kDa	NA	NA	NA	0	0
sp|P54136	Isoform Mc	RARS	67 kDa	NA	NA	NA	0	0
sp|Q12931	Heat shock	TRAP1	80 kDa	NA	NA	NA	50900000	96500000
sp|P09488	Isoform 2 c	GSTM1	21 kDa	NA	NA	NA	3978200	9015700
sp|Q9NQR	Omega-am	NIT2	31 kDa	NA	NA	NA	0	0
sp|Q16698	Isoform 2 c	DECR1	35 kDa	NA	NA	NA	0	0
sp|Q8IYF3-	Isoform 3 c	TEX11	107 kDa	NA	NA	NA	0	0
sp|P05814	Beta-casein	CSN2	25 kDa	NA	NA	NA	0	0
tr|A0A087	Ig gamma-3	IGHG3	57 kDa	NA	NA	NA	19600000	3.90E+07
sp|P42677	40S ribosor	RPS27	9 kDa	NA	NA	NA	0	0
sp|Q13283	Ras GTPase	G3BP1	52 kDa	NA	NA	NA	0	5029400
sp|P20592	Interferon-	MX2	82 kDa	NA	NA	NA	3936600	4678400
sp|P38919	Eukaryotic	EIF4A3	47 kDa	NA	NA	NA	12800000	26700000
sp|P17844	Isoform 2 c	DDX5	61 kDa	NA	NA	NA	2939800	10400000

sp|P18621	Isoform 2 c	RPL17	17 kDa	NA	NA	NA	1283900	5131000
sp|P04279	Isoform 2 c	SEMG1	45 kDa	NA	NA	NA	0	0
sp|P80723	Isoform 2 c	BASP1	18 kDa	NA	NA	NA	0	0
sp|P13798	Acylamino-	APEH	81 kDa	NA	NA	NA	0	0
sp|P22528	Cornifin-B	SPRR1B	10 kDa	NA	NA	NA	0	0
sp|Q9BVK6	Transmem	TMED9	27 kDa	NA	NA	NA	0	0
sp|Q9Y5Y2	Cytosolic F	NUBP2	29 kDa	NA	NA	NA	0	0
sp|NQO2_	sp|NQO2_	NQO2	26 kDa	NA	NA	NA	0	0
sp|P26583	High mobil	HMGB2	24 kDa	NA	NA	NA	1627600	8150800
sp|P17213	Bactericida	BPI	54 kDa	NA	NA	NA	0	0
sp|P32320	Cytidine de	CDA	16 kDa	NA	NA	NA	0	0
tr|C9JNS9|	V-type prot	ATP6V1B1	27 kDa	NA	NA	NA	2373100	3041300
sp|P30613	Isoform L-t	PKLR	58 kDa	NA	NA	NA	12100000	16400000
sp|Q6E0U4	Isoform 3 c	DMKN	37 kDa	NA	NA	NA	0	0
sp|Q9Y5G5	Protocadhe	PCDHGA8	101 kDa	NA	NA	NA	0	0
sp|Q02218	2-oxogluta	OGDH	116 kDa	NA	NA	NA	2943200	3074900
sp|P23396	40S ribosor	RPS3	27 kDa	NA	NA	NA	2263400	3571300
sp|P08865	40S ribosor	RPSA	33 kDa	31900000	42800000	31100000	NA	NA
sp|P39687	Acidic leuci	ANP32A	29 kDa	NA	NA	NA	0	0
sp|Q99798	Aconitate h	ACO2	85 kDa	NA	NA	NA	77200000	75300000
sp|P07741	Adenine ph	APRT	20 kDa	1727200	936850	1309800	NA	NA
sp|P00568	Adenylate l	AK1	22 kDa	NA	NA	NA	538810	661860
sp|Q10588	ADP-ribosy	BST1	36 kDa	0	0	0	NA	NA
sp|P42330	Aldo-keto r	AKR1C3	37 kDa	37200000	36300000	26600000	NA	NA
sp|P15121	Aldose redu	AKR1B1	36 kDa	NA	NA	NA	3293000	7219400
sp|Q92499	ATP-depen	DDX1	82 kDa	NA	NA	NA	1021400	2830100
tr|A0A0C4	Calpain sm	CAPNS1	34 kDa	82300000	1.34E+08	22800000	NA	NA
sp|Q99439	Calponin-2	CNN2	34 kDa	1.20E+07	14500000	11800000	NA	NA
sp|P13987	CD59 glyco	CD59	14 kDa	0	5694000	19800000	NA	NA
sp|P20908	Collagen al	COL5A1	184 kDa	NA	NA	NA	0	7364400
sp|P12109	Collagen al	COL6A1	109 kDa	1.24E+09	1.90E+09	1.44E+09	NA	NA
sp|Q16527	Cysteine ar	CSRP2	21 kDa	NA	NA	NA	6788100	15300000
sp|Q9HC35	Echinoderm	EML4	109 kDa	1370800	0	0	NA	NA
sp|P15311	Ezrin	EZR	69 kDa	2.86E+08	3.98E+08	3.32E+08	NA	NA
sp|Q14192	Four and a	FHL2	32 kDa	NA	NA	NA	1.40E+07	2.90E+07
sp|Q08380	Galectin-3-	LGALS3BP	65 kDa	4363100	18900000	5393800	9880900	14900000
tr|H0YBD7	Heterogen	HNRNPH1	21 kDa	NA	NA	NA	7648000	16200000
tr|A0A087	Ig kappa ch	IGKC	26 kDa	1.54E+08	2.54E+08	1.74E+08	NA	NA
sp|Q12905	Interleukin	ILF2	43 kDa	10200000	1.30E+07	4264000	NA	NA
sp|P24043	Laminin sul	LAMA2	344 kDa	0	2466200	0	NA	NA
sp|P07942	Laminin sul	LAMB1	198 kDa	NA	NA	NA	0	1202500
sp|P43243	Matrin-3	MATR3	95 kDa	3173700	8516000	6805000	NA	NA
tr|H3BQK9	Microtubul	MACF1	861 kDa	27400000	14800000	49200000	NA	NA
sp|P60660	Myosin ligh	MYL6	17 kDa	4.36E+08	7.81E+08	5.49E+08	NA	NA
sp|Q14112	Nidogen-2	NID2	151 kDa	NA	NA	NA	8509600	29600000
sp|Q9NTK5	Obg-like AT	OLA1	45 kDa	NA	NA	NA	3148400	4174700
sp|Q99623	Prohibitin-2	PHB2	33 kDa	NA	NA	NA	1134000	0

sp|Q9UL46	Proteasom	PSME2	27 kDa	3849700	2013200	1687500	NA	NA
sp|P55786	Puromycin-	NPEPPS	103 kDa	0	0	3265500	NA	NA
sp|Q9HBL0	Tensin-1	TNS1	186 kDa	1.00E+08	1.19E+08	1.15E+08	NA	NA
sp|P13693	Translation	TPT1	20 kDa	3500600	15300000	11800000	NA	NA
sp|Q96JG9	Zinc finger	ZNF469	410 kDa	NA	NA	NA	2382200	28500000

FR_MAX FR9.2	FR_TX.MAX FR3.1.M	FR_TX.MAX FR6.1.M	FR_TX.MAX FR8.1.M	FR_TX.MAX FR3.2.M	FR_TX.MAX FR6.2.M	FR_TX.MAX FR8.2.M	FR_SDS.M/ FR1.1.M
5.45E+08	1.30E+09	5.80E+09	1.44E+09	3.96E+08	3.32E+09	1.11E+09	6.37E+09
2.50E+09	5.52E+09	5.94E+09	7.50E+09	1.97E+09	2.91E+09	4.65E+09	1.10E+09
3.85E+09	5.94E+09	5.49E+09	9.43E+09	2.08E+09	2.84E+09	6.07E+09	7.46E+08
1.16E+09	2.63E+09	2.51E+09	3.29E+09	9.42E+08	1.22E+09	1.93E+09	3.54E+09
1.74E+09	3.69E+09	3.57E+09	4.72E+09	1.34E+09	1.91E+09	3.03E+09	5.85E+09
1.19E+08	1.69E+09	8.08E+09	1.77E+09	5.55E+08	3.81E+09	1.07E+09	5.09E+09
4.31E+08	2.72E+09	1.02E+10	2.67E+09	7.29E+08	5.23E+09	1.67E+09	8.37E+09
3.73E+09	2.05E+09	1.79E+09	3.42E+09	7.32E+08	1.04E+09	2.13E+09	2.37E+08
1.08E+09	2.32E+09	2.35E+09	3.03E+09	8.56E+08	1.24E+09	1.93E+09	4.79E+09
8.05E+08	1.84E+09	1.74E+09	2.39E+09	7.50E+08	9.86E+08	1.66E+09	5.58E+08
1.37E+09	3.10E+09	2.99E+09	3.79E+09	1.06E+09	1.62E+09	2.48E+09	6.10E+09
6.60E+08	9.62E+08	5.58E+09	1.58E+09	4.00E+08	4.07E+09	1.17E+09	5.59E+09
1.03E+09	1.80E+09	1.56E+09	2.11E+09	5.91E+08	7.84E+08	1.27E+09	1.66E+09
8.10E+08	1.95E+09	1.91E+09	2.37E+09	5.95E+08	8.44E+08	1.15E+09	4.51E+09
8.90E+08	1.55E+09	1.50E+09	1.98E+09	4.46E+08	6.57E+08	8.50E+08	5.63E+09
5.48E+08	3.66E+08	3.94E+08	6.08E+08	1.40E+08	1.99E+08	3.89E+08	1.23E+08
2.95E+08	4.32E+08	3.90E+08	6.27E+08	1.56E+08	2.06E+08	3.95E+08	69200000
9.76E+08	1.92E+09	1.81E+09	2.97E+09	8.43E+08	9.75E+08	1.97E+09	3.35E+08
1.89E+08	6.07E+08	5.62E+08	7.41E+08	2.44E+08	3.51E+08	5.22E+08	11500000
5.36E+08	3.38E+08	3.26E+08	5.40E+08	1.21E+08	1.50E+08	3.41E+08	1038600
5.75E+08	7.48E+08	8.36E+08	1.35E+09	3.05E+08	4.52E+08	7.86E+08	1.17E+08
7.70E+08	1.64E+09	1.67E+09	1.89E+09	4.99E+08	7.79E+08	1.17E+09	1.65E+09
3.54E+08	6.47E+08	6.16E+08	8.33E+08	2.53E+08	3.03E+08	4.71E+08	1.53E+09
5.11E+08	8.43E+08	1.94E+09	1.64E+09	2.89E+08	9.88E+08	8.56E+08	1.17E+09
9.96E+08	2.85E+09	2.84E+09	2.81E+09	8.60E+08	1.19E+09	1.79E+09	6.86E+09
5.09E+08	11100000	1.20E+07	28800000	1799700	4204000	16100000	33900000
2.32E+08	5.02E+08	4.88E+08	6.94E+08	1.86E+08	2.40E+08	4.16E+08	33400000
1.07E+09	1.84E+09	1.96E+09	2.78E+09	8.90E+08	1.44E+09	2.02E+09	3.72E+09
6.35E+08	7.60E+08	7.64E+08	1.05E+09	2.50E+08	3.81E+08	6.81E+08	38700000
8.95E+08	3.23E+09	2.24E+09	2.92E+09	8.89E+08	9.96E+08	1.77E+09	5.62E+09
2.70E+08	3.68E+08	4.44E+08	5.81E+08	1.33E+08	2.37E+08	2.98E+08	9.87E+08
6.92E+08	2.27E+09	1.88E+09	1.94E+09	5.56E+08	1.04E+09	1.18E+09	3.62E+09
6.37E+08	1.22E+09	1.39E+09	1.90E+09	3.99E+08	6.89E+08	1.09E+09	1.96E+08
3.33E+08	4.80E+08	5.51E+08	7.51E+08	2.45E+08	2.98E+08	4.97E+08	1.33E+09
4.23E+08	7.38E+08	8.08E+08	1.13E+09	2.81E+08	4.22E+08	6.72E+08	1.31E+08
2.11E+08	2.29E+08	2.22E+08	3.44E+08	98200000	74500000	2.52E+08	1425000
1.95E+08	1.40E+08	1.89E+08	3.16E+08	5.20E+07	58500000	1.62E+08	16700000
3.75E+08	6.72E+08	1.42E+09	1.19E+09	2.19E+08	6.83E+08	6.50E+08	8.93E+08
3.57E+08	4.76E+08	4.28E+08	6.05E+08	2.00E+08	2.34E+08	3.95E+08	13100000
2.62E+08	7.30E+08	7.79E+08	1.10E+09	1.73E+08	2.81E+08	4.67E+08	1.03E+08
1.11E+08	3.41E+08	2.67E+08	4.64E+08	1.34E+08	1.43E+08	2.44E+08	14100000
1.32E+08	3.03E+08	2.74E+08	3.54E+08	1.18E+08	1.84E+08	2.56E+08	87800000
2.12E+08	4.66E+08	5.01E+08	6.41E+08	1.90E+08	2.86E+08	4.03E+08	3257300
1.08E+08	3.32E+08	4.26E+08	4.03E+08	36500000	66300000	63100000	1.09E+09
3.56E+08	6.16E+08	5.21E+08	7.82E+08	1.89E+08	2.87E+08	5.19E+08	91300000

0	0	48400000	0	0	13900000	0	32400000
1.96E+08	1.27E+08	1.15E+08	2.16E+08	53400000	8.30E+07	1.60E+08	17500000
3.45E+08	4.98E+08	4.60E+08	6.90E+08	1.69E+08	2.68E+08	4.08E+08	82200000
3.78E+08	5.57E+08	4.60E+08	9.69E+08	1.90E+08	2.56E+08	6.05E+08	69700000
1.97E+08	4.25E+08	4.73E+08	5.84E+08	1.40E+08	2.53E+08	4.08E+08	1.67E+08
62700000	1.28E+08	1.37E+08	2.02E+08	43400000	65800000	1.33E+08	13200000
81600000	2.03E+08	2.49E+08	3.22E+08	1.07E+08	1.48E+08	2.12E+08	67500000
1.44E+08	1.67E+08	1.99E+08	3.16E+08	73800000	1.19E+08	2.29E+08	9233400
1.71E+08	45300000	41200000	93300000	13600000	14700000	45600000	34900000
91800000	1.11E+08	1.37E+08	1.52E+08	3.70E+07	61600000	62100000	3.20E+07
2181000	84500000	1.61E+08	3.50E+07	15600000	5.00E+07	56700000	2.81E+08
1.94E+08	3.21E+08	3.31E+08	4.73E+08	1.21E+08	2.01E+08	3.72E+08	37400000
89900000	20300000	22800000	67400000	7005200	9851800	42200000	9846100
40500000	14800000	32500000	45300000	7350200	14600000	16300000	5.00E+07
7.41E+08	9.72E+08	1.12E+09	1.10E+09	3.56E+08	7.44E+08	1.01E+09	74800000
1.32E+08	1.67E+08	1.55E+08	2.43E+08	75200000	1.08E+08	2.17E+08	77100000
54900000	1.51E+08	1.49E+08	2.53E+08	42300000	72100000	1.36E+08	8530800
1.63E+08	1.43E+08	1.58E+08	2.37E+08	60400000	80800000	2.10E+08	25700000
33400000	1.30E+08	96100000	1.65E+08	56200000	6.10E+07	86900000	4821800
1.30E+08	2.03E+08	1.85E+08	3.11E+08	67600000	1.07E+08	2.30E+08	7245700
1.75E+08	26400000	18600000	51700000	11900000	13700000	20300000	38600000
1.19E+08	2.03E+08	2.12E+08	2.60E+08	7.00E+07	84600000	1.70E+08	30800000
1.58E+08	3.39E+08	3.51E+08	4.35E+08	1.33E+08	1.92E+08	2.91E+08	17800000
1.27E+08	0	0	0	0	0	0	0
1.13E+08	1.48E+08	1.35E+08	2.47E+08	53700000	34600000	1.04E+08	5374800
0	4318600	51800000	0	0	29900000	0	6.50E+07
4.66E+08	6.23E+08	8.83E+08	7.37E+08	2.68E+08	5.22E+08	4.98E+08	59300000
1.88E+08	3.66E+08	3.41E+08	4.33E+08	1.37E+08	1.97E+08	2.74E+08	3.76E+08
96200000	1.69E+08	2.36E+08	3.40E+08	67600000	1.14E+08	1.86E+08	26700000
1.11E+08	57200000	42700000	1.05E+08	20100000	34700000	51300000	20300000
0	0	0	0	804680	1738400	0	3878900
6.00E+07	1.02E+08	1.06E+08	1.81E+08	21300000	72200000	1.22E+08	49900000
1.54E+08	2.14E+08	5.46E+08	4.42E+08	8.90E+07	1.89E+08	1.94E+08	60500000
98100000	1.09E+08	91200000	1.49E+08	18500000	4.70E+07	95700000	0
42100000	99200000	1.20E+08	1.66E+08	37200000	63100000	1.09E+08	22600000
1.02E+08	24100000	2.00E+07	68800000	7498500	11800000	36400000	4468900
56300000	1.83E+08	1.43E+08	2.08E+08	41300000	78600000	86800000	2.63E+08
1.25E+08	2.53E+08	3.07E+08	4.07E+08	1.12E+08	1.46E+08	2.24E+08	1.11E+08
1.65E+08	2.10E+08	1.48E+08	2.68E+08	53900000	79100000	1.87E+08	33600000
49400000	50200000	33300000	64900000	23800000	30700000	59200000	1246500
37800000	2.70E+07	19600000	39200000	10500000	10800000	28800000	1166000
44300000	51700000	1.02E+08	1.37E+08	36800000	6.80E+07	1.03E+08	7526200
88500000	71800000	32900000	73500000	21300000	36400000	1.05E+08	0
37900000	84500000	1.10E+08	1.12E+08	23500000	55700000	85600000	22200000
96400000	84900000	91300000	1.34E+08	4.00E+07	59700000	89900000	1.67E+08
16100000	0	0	1346800	0	1143200	1475300	0
3.20E+07	33800000	3.20E+07	36600000	6438600	3182700	12600000	6563400

76100000	72400000	7.50E+07	1.04E+08	19600000	31800000	50500000	23800000
66800000	1.58E+08	1.80E+08	2.39E+08	47700000	88900000	1.72E+08	45800000
62200000	66700000	1.31E+08	87200000	26200000	35700000	93200000	16900000
50100000	34600000	29800000	6.70E+07	11300000	22800000	51100000	7313900
1.06E+08	25200000	24100000	1.13E+08	11500000	21200000	54600000	0
60200000	90200000	82900000	1.40E+08	40100000	48300000	85600000	7126000
31800000	1.14E+08	1.22E+08	1.30E+08	33300000	5.30E+07	77700000	15700000
20800000	16500000	57800000	68100000	6097800	31200000	3.90E+07	5662900
41600000	1.05E+08	97200000	1.05E+08	3.60E+07	51500000	75900000	3265100
38400000	93400000	5.60E+07	3.90E+07	11200000	28300000	33200000	4.37E+08
1.32E+08	2.99E+08	2.86E+08	2.85E+08	88800000	1.70E+08	1.88E+08	1.06E+08
48800000	1.37E+08	91200000	1.76E+08	61400000	64700000	92700000	2.56E+08
39700000	7.30E+07	1.03E+08	1.59E+08	35100000	6.30E+07	69800000	10900000
19600000	48500000	43400000	72200000	20300000	39800000	33800000	4.50E+07
2603100	6193700	24500000	10100000	2146200	14300000	3797200	0
1.31E+08	67300000	53100000	88200000	14900000	18500000	45200000	17900000
15200000	0	6184600	0	0	2790600	0	4970700
23700000	7984900	0	14600000	5331400	7273700	11400000	6416000
33700000	54700000	50200000	1.03E+08	25700000	33400000	58300000	3059200
16900000	8491500	2.80E+07	31200000	964870	12700000	16100000	0
0	0	21700000	0	0	7467500	0	11900000
0	0	19500000	0	0	6947600	0	29800000
42300000	1.23E+08	84300000	72600000	37500000	40600000	56600000	17600000
83400000	38800000	4.90E+07	93600000	10300000	23200000	45300000	3846900
60700000	1.08E+08	1.17E+08	1.47E+08	41600000	59400000	95800000	19400000
27400000	22600000	1.10E+07	32100000	4089300	7587800	15600000	0
1.25E+08	1.40E+08	1.20E+08	2.24E+08	40200000	42200000	99400000	10500000
1.73E+08	1.41E+08	56700000	2.16E+08	50300000	56200000	1.55E+08	25600000
32200000	89400000	61800000	82800000	29700000	44200000	61200000	4742400
19900000	33300000	30600000	57500000	8909800	7832300	23700000	0
80200000	1.26E+08	1.56E+08	1.94E+08	56100000	94400000	97700000	5.13E+08
49500000	74100000	60100000	98400000	37700000	42500000	71500000	11900000
40700000	9.90E+07	86200000	1.01E+08	35800000	47200000	87500000	8797100
21100000	6036800	3231100	41600000	4426400	8062300	35300000	2877100
5.60E+07	66200000	1.09E+08	1.73E+08	36400000	39500000	60900000	6495400
24600000	83900000	75900000	1.31E+08	22100000	37400000	70900000	759440
20900000	6.50E+07	61100000	98500000	1.50E+07	40500000	74100000	0
25600000	19400000	4940300	21100000	18600000	20600000	36400000	22300000
47800000	28600000	34400000	64600000	6382200	10700000	28400000	0
87800000	28800000	51100000	74200000	8815900	27700000	51100000	2.50E+07
25200000	1.12E+08	94800000	1.20E+08	22300000	31600000	21600000	64300000
0	0	0	0	0	0	0	1433600
0	0	26600000	0	0	44800000	0	27100000
1.90E+07	0	8747700	7281400	0	0	3103300	0
19200000	24200000	24400000	33600000	6947300	9439200	13300000	0
17700000	67200000	5.00E+07	55200000	22700000	30500000	60700000	0
5.40E+07	9781600	51700000	57700000	8444000	24600000	10400000	0

34800000	87100000	85900000	1.29E+08	29100000	54300000	62600000	65100000
9667500	16600000	19300000	14900000	13300000	15800000	18500000	83400000
6641500	32300000	36700000	40900000	12600000	11500000	20900000	14700000
736750	22700000	41900000	26900000	8078900	21900000	14900000	2823300
37400000	59900000	96300000	90700000	13500000	36300000	57900000	1.96E+08
24200000	59700000	56500000	5.60E+07	29600000	34700000	48600000	0
27900000	14400000	9858300	1.50E+07	5466300	5939700	16300000	352390
40800000	98800000	1.03E+08	1.56E+08	30700000	57800000	1.00E+08	15400000
34700000	85500000	88100000	1.35E+08	34500000	54800000	85400000	58600000
31300000	1.03E+08	1.37E+08	1.64E+08	31800000	63600000	93800000	42300000
31900000	4.10E+07	6.00E+07	26600000	3177200	7.18E+08	28900000	4677000
36500000	1.05E+08	1.68E+08	1.73E+08	44200000	82700000	1.27E+08	73800000
10200000	13200000	6765500	19800000	6435200	3201100	14600000	28800000
29200000	1.50E+07	22800000	21800000	3805400	5571700	11700000	6098200
18700000	32300000	38700000	47200000	22400000	30200000	36900000	0
1.60E+07	62700000	43900000	78100000	2.10E+07	20800000	36300000	6833400
50200000	1.09E+08	81100000	1.11E+08	34200000	63100000	88200000	43900000
49800000	98500000	1.22E+08	1.85E+08	32300000	78600000	94900000	54700000
3.80E+07	62900000	39100000	91100000	23300000	31700000	69100000	0
12500000	0	0	0	590060	628370	4176400	0
11800000	15400000	2.80E+07	17200000	7615700	11800000	28900000	2.30E+08
80100000	1.46E+08	1.33E+08	1.90E+08	61700000	86800000	1.46E+08	21200000
33900000	60300000	6.50E+07	95300000	43400000	4.20E+07	68800000	3.20E+07
40600000	59600000	21700000	84800000	11400000	45900000	61300000	0
0	0	0	0	0	0	0	0
0	0	9587900	0	0	3519200	0	4007000
8289000	38100000	26900000	58300000	13900000	14900000	41900000	4408600
19600000	52400000	39500000	65300000	15700000	25700000	42800000	0
20800000	59400000	41200000	8.00E+07	22400000	31800000	3.70E+07	2997900
19800000	64700000	95600000	55100000	23800000	4.50E+07	4.20E+07	2.60E+07
1896600	1.50E+07	17200000	6663500	2748800	10400000	4485700	0
24900000	4201300	5065000	19700000	0	0	9090200	0
1.80E+07	31400000	26300000	5.10E+07	22800000	19800000	25400000	47400000
15500000	9580800	12900000	28600000	1608800	4944300	20700000	10100000
27100000	3622000	3566500	5891600	960000	0	6452100	0
8400400	0	0	5219600	0	0	0	0
14900000	38900000	25200000	50300000	15100000	17200000	15600000	0
1.50E+07	29200000	29300000	46500000	13600000	16600000	27600000	17100000
32400000	46200000	34900000	86200000	10100000	6927300	22900000	0
18500000	3.50E+07	31100000	47100000	13400000	12600000	24900000	773440
44400000	22600000	18200000	52800000	22300000	9891500	25300000	0
1.40E+07	38200000	30900000	39800000	12900000	12100000	26400000	0
19300000	53800000	52700000	70900000	17700000	16500000	20800000	0
1.25E+08	2.52E+08	8.90E+07	77400000	42800000	1.16E+08	85900000	12600000
6696200	0	2776100	0	0	4284700	0	521180
13200000	8538100	27200000	26300000	3832600	5684500	15800000	0
19900000	32700000	46800000	86300000	23600000	33800000	49800000	11700000

17800000	24600000	16600000	27800000	8027000	16900000	20700000	0
27400000	23600000	13700000	27900000	1.20E+07	15600000	32400000	0
30200000	24300000	1886300	31200000	9113600	5003300	1.40E+07	0
0	0	1.19E+08	0	0	45200000	0	72800000
4402400	15100000	1.40E+07	7009400	6505500	15900000	9443500	0
22900000	8483500	11500000	11900000	3139800	3554900	5421400	4907700
3835500	14100000	10500000	13700000	3236100	5970800	14300000	0
27900000	73700000	62200000	93200000	28700000	4.80E+07	64400000	2088900
17100000	52100000	43300000	8.00E+07	14400000	23100000	44700000	0
9039300	43100000	44700000	39600000	10500000	15200000	27700000	0
6983300	29400000	24500000	28100000	7978200	19800000	26900000	0
15600000	11600000	37900000	37400000	6111600	7900800	16800000	0
0	0	0	0	0	0	0	0
40900000	53200000	47700000	58800000	39300000	1.90E+07	4.50E+07	1.11E+08
57500000	54800000	92700000	1.61E+08	4.10E+07	60800000	1.12E+08	16200000
8902600	10900000	12600000	17600000	5478200	9567800	20500000	1759600
1100100	11200000	8365900	26200000	1170000	1118400	5269200	0
10500000	0	0	3145800	0	0	0	0
28700000	84900000	53100000	64400000	9269100	25500000	21700000	0
27600000	79700000	66400000	95900000	21900000	38700000	51500000	0
18100000	0	3158100	0	0	0	4235100	0
82500000	1.05E+08	82600000	1.06E+08	7400700	42200000	67900000	0
1.15E+08	3.31E+08	1.26E+09	3.34E+08	1.08E+08	8.14E+08	2.86E+08	1.41E+09
0	0	0	0	0	0	0	0
21300000	10100000	10800000	28400000	7110800	7242500	25300000	0
15400000	5321200	0	16400000	3311900	2335300	5649200	0
12100000	0	0	2226100	1482700	2874300	0	9237400
14800000	5558000	1639300	18500000	2061100	11300000	9234500	0
4505500	0	0	10400000	0	788960	1688500	0
0	0	1570700	0	0	0	0	74200000
12700000	16900000	22800000	24400000	6469700	7925800	9370200	1.01E+08
20800000	1.90E+07	16600000	43600000	10300000	18900000	28600000	0
0	0	0	0	0	0	0	0
0	0	0	0	904610	0	0	0
1.50E+07	42200000	14500000	20800000	4798700	1.70E+07	2.80E+07	2794600
2.10E+07	27100000	12800000	16700000	13900000	11900000	28900000	2150100
16100000	15700000	28500000	54300000	14600000	2.10E+07	33400000	6833800
1631300	0	4320800	4144200	1117000	2833600	4273700	6889500
11900000	25900000	34400000	30500000	6364900	20200000	22400000	6172000
0	0	0	0	0	0	0	0
52700000	60900000	22400000	35500000	1.00E+07	14100000	66300000	191480
14800000	20200000	3516900	19500000	8283800	10400000	10100000	0
24400000	14200000	15700000	42100000	11900000	18200000	16400000	0
25700000	40300000	36400000	50300000	26200000	28700000	43300000	0
0	0	0	0	0	0	0	0
38100000	22600000	1.90E+07	85300000	8803100	26900000	3.00E+07	0
5524300	0	0	474870	0	0	0	0

7314500	86900000	5485000	14300000	6777400	6557800	12600000	0
9803200	1.20E+07	13600000	22800000	2837000	6438900	11700000	0
13500000	4.50E+07	46400000	61600000	8767200	16500000	22100000	14300000
0	0	0	0	4669200	0	1424900	0
1.10E+07	16500000	16600000	17600000	2814200	5571700	9094600	0
1436300	10600000	9384000	16400000	5002200	9497000	11200000	608880
6914100	17300000	28100000	35700000	18600000	13200000	32600000	11100000
0	0	0	0	0	0	0	0
20300000	32200000	45200000	47500000	5138200	14100000	28500000	14900000
3.46E+09	4.78E+09	4.88E+09	7.50E+09	2.37E+09	2.69E+09	4.28E+09	1.10E+09
20300000	27600000	40100000	47100000	14900000	12600000	28200000	0
1974500	11600000	2226000	22900000	6871300	6882900	11400000	0
6031700	3731400	5074600	2626700	3386400	4958300	9565700	0
12800000	0	0	0	0	0	2548300	0
13700000	38200000	3.40E+07	52900000	11200000	18500000	22100000	0
13900000	18300000	17500000	23800000	10800000	9436200	16100000	2029300
47700000	6.50E+07	91100000	97200000	32900000	37800000	72100000	0
9856000	22500000	23400000	29800000	7952400	11800000	12900000	0
7451300	8165600	6257700	11300000	1885700	2415800	8511900	0
8611700	12700000	19600000	27300000	7534700	13700000	11300000	0
1.10E+07	29500000	18800000	1.60E+07	2351800	7513600	11200000	0
2560900	6091500	6446400	8855400	2225000	4011400	6055400	424240
6242000	12700000	9143800	16800000	8371400	11400000	12500000	0
5875300	0	0	0	1068600	2271000	3032000	0
6358000	25100000	15500000	26500000	4859100	8114200	11600000	391010
49100000	80800000	1.08E+08	1.43E+08	34600000	42400000	53100000	52600000
8107100	21400000	22500000	31800000	9399600	9941800	20500000	0
8203300	0	0	0	0	0	0	0
16100000	16100000	3794800	14600000	7221800	21300000	36200000	0
7069800	0	0	0	0	0	0	0
1.20E+07	0	0	12100000	0	0	6030000	0
21900000	2.70E+07	21100000	34900000	8446600	15200000	2.10E+07	1784500
9076500	0	0	0	0	0	0	0
2476100	2104000	2670400	0	1268100	1970000	1555000	0
19800000	28600000	31100000	44100000	883950	1507600	2994600	0
7147500	0	8214100	12100000	0	0	647410	0
0	0	0	0	0	0	0	0
8561500	41500000	27500000	38600000	12100000	1.70E+07	36100000	5778800
9502900	30800000	18300000	52800000	4313200	5237800	1.20E+07	0
11300000	24600000	27500000	49800000	15500000	23500000	40400000	10100000
9180200	4610700	4886400	19200000	0	0	0	0
20700000	32800000	27300000	48600000	18500000	25800000	50800000	3572600
2082400	6335700	11700000	10900000	5205000	11700000	12200000	1473300
19400000	29500000	27700000	7.50E+07	23500000	28600000	24500000	8011500
11600000	31100000	47600000	59600000	8656400	19100000	28800000	12900000
1871200	13600000	15100000	24300000	6502700	9883600	1.30E+07	0
9297700	12900000	12600000	15800000	9894000	10400000	24800000	3442900

14800000	5975700	2408800	8540500	0	0	0	0
38600000	35800000	49800000	76100000	15700000	15200000	29600000	5250100
12300000	0	0	0	0	0	0	0
2892000	18100000	14800000	46500000	9790700	12600000	13200000	0
16100000	21700000	32300000	44600000	13100000	1.90E+07	28400000	0
0	0	0	0	0	0	0	362890
6843200	11200000	20200000	20300000	4846700	15300000	17500000	0
8333600	14700000	3265700	22200000	7814400	4549800	11400000	0
12700000	1.10E+07	9371500	21800000	1510600	1617600	12200000	0
2899700	0	3386300	5054000	0	0	4600200	0
0	0	12100000	11300000	0	1867800	2732100	16400000
1021300	0	0	0	0	0	0	0
0	0	0	0	0	1451200	0	0
10800000	20900000	23400000	28900000	4385100	1342300	1.40E+07	0
4339600	9587200	6962800	9054600	4626400	12700000	17100000	0
8312500	19100000	40200000	35100000	8737800	20100000	20400000	1389300
0	0	0	600960	660020	0	0	0
8870500	2.00E+07	8165900	10600000	9714600	10700000	12500000	0
0	14100000	13300000	28900000	3937400	4321700	6077200	0
10500000	0	0	0	0	0	0	0
15200000	67900000	50700000	67900000	18200000	22700000	33900000	0
1175500	15300000	9482200	1.70E+07	5279200	11400000	6613800	0
14100000	23800000	14100000	26100000	7962800	14300000	22700000	6287000
5048200	0	0	0	0	0	0	0
881180	5410200	2973600	2663500	735970	4398900	9439700	0
11900000	14600000	13100000	21100000	9670300	11300000	16900000	0
27200000	9762400	18600000	12500000	4922400	13100000	18600000	0
13900000	21200000	17700000	24300000	11500000	15200000	22300000	0
8563400	248860	3108500	16100000	0	366390	9370000	0
819870	0	0	0	0	0	0	0
3115100	0	0	0	717180	665660	1159900	0
39100000	54800000	59800000	1.44E+08	12600000	45600000	8.10E+07	0
14600000	0	0	0	0	0	0	0
15900000	36100000	21100000	12200000	4855100	6740700	16400000	0
3648700	6462600	2040000	4334900	1197200	2299900	2336200	0
13200000	2.40E+07	20200000	21500000	10900000	16700000	30800000	0
0	0	0	0	0	0	0	0
3204200	2063900	0	5963500	2952400	3103200	7053100	0
27100000	15300000	15200000	24500000	6059600	8747700	15300000	5250100
24100000	42700000	37500000	47900000	0	20500000	28200000	1.06E+08
1.10E+07	4374000	2498200	6008100	1514300	1842700	3671900	0
4720600	3215100	0	8322700	1131900	0	5951100	0
18700000	36900000	19600000	36100000	6266800	8485400	12300000	0
2520200	0	0	0	0	0	0	0
0	6269600	0	0	0	0	0	0
405950	1129600	1632000	0	599660	0	0	0
12800000	3.00E+07	4.30E+07	41500000	17400000	16100000	20900000	3613300

3264200	12300000	1.30E+07	8388600	4285600	6309800	6692900	131250
3987000	3827800	10200000	10500000	2019200	5598400	9630800	0
10300000	14600000	26700000	2610000	4384700	4633200	13300000	0
13800000	9418600	27400000	14100000	10700000	15400000	27900000	8514800
4439500	14800000	12900000	17600000	4188800	4380500	10700000	0
10500000	15200000	10500000	30600000	3200400	11500000	34300000	0
10100000	17700000	1.80E+07	21700000	6506200	8526000	19800000	1013700
3875900	13300000	16800000	19400000	9344300	12700000	15800000	0
0	0	0	0	0	0	0	0
0	16300000	14400000	27800000	9107200	18600000	27200000	5410900
2506900	14600000	13100000	15200000	3718400	8173700	7884700	0
14300000	2665200	0	6878200	4231800	955740	12100000	0
499650	0	0	0	0	0	0	0
0	10300000	9705000	13600000	2101100	0	5511800	0
9636600	0	0	0	0	0	0	0
1904600	2877400	6329100	5330600	0	0	0	1262100
3477000	0	0	3828400	0	2542500	0	0
3070100	0	0	0	0	0	0	0
11800000	11800000	10700000	26100000	5589300	8082000	25800000	0
0	0	0	0	0	0	0	0
0	0	0	0	0	0	0	3279600
5475800	13100000	8206600	16900000	8263500	10200000	22400000	1889100
698380	3313700	4353900	6158300	1571200	760730	1.20E+07	0
3571400	0	0	0	0	0	0	0
1367100	10900000	9290300	14100000	1710900	5350500	8926800	0
1658000	9294300	5006700	23500000	1379600	899380	4580200	0
0	13700000	9324100	12300000	1896100	1831000	10300000	0
0	0	0	0	0	0	0	0
1668900	12400000	6294200	27200000	5900200	4708500	6879600	0
4126600	0	0	0	0	0	0	0
0	0	0	0	0	0	0	0
563050	0	1145600	5340000	2757500	2348500	4850200	0
2898100	7743100	7811900	7944100	3050000	0	0	0
0	5751400	0	6751300	0	0	1047000	0
3490300	0	0	0	0	0	0	0
2108900	5981100	6476200	8348100	1741200	2373800	2844300	0
3620700	776520	0	0	3907200	7101200	7224600	0
16300000	0	0	0	0	0	0	0
0	0	0	0	0	306070	0	0
2411500	8516300	9920800	13400000	3233000	2949600	5800200	1576900
8665600	1253100	1733500	9409800	8437800	10500000	20700000	0
502890	658300	0	483880	467450	0	2237200	715710
6989400	9159800	0	2976000	2536000	3463200	1049200	0
29400000	0	0	0	0	0	0	0
12400000	0	0	0	0	0	0	0
436160	0	0	0	0	0	684480	0
1863600	5602500	8245800	9224900	4408400	1963000	5121700	0

2066500	11700000	35400000	8488900	5833300	20800000	8503100	0
3248100	18800000	12300000	16400000	8164400	3750400	16500000	0
9883100	10900000	11100000	11600000	4174600	7325000	11800000	0
1359500	8797400	10700000	10300000	3226700	2319200	8039600	0
5433500	2928200	0	4492200	0	0	0	0
0	0	13500000	0	0	0	0	0
8030100	19600000	8933000	12800000	4962000	5431700	10700000	2740100
2759600	3894800	5027600	6905000	3715200	4008900	5506400	0
4799700	0	0	5046400	0	0	0	0
0	10300000	3235000	4940000	1243500	1916600	2384600	0
16200000	2099600	0	0	0	0	0	0
943550	0	0	0	0	0	0	0
3323800	4108600	0	12800000	3450900	5396100	6910300	0
0	0	0	0	0	0	0	0
3916100	3740300	3645600	7675800	3582200	6743100	4687200	0
3.20E+07	47400000	60400000	1.60E+07	8337500	9283600	3.20E+07	20900000
3296300	0	0	0	0	0	0	0
2936700	0	0	4492800	1561300	1842700	5081600	0
5595100	9488100	13500000	16800000	4615600	4938600	7341400	0
0	0	0	0	0	0	0	0
0	0	0	0	0	0	0	0
10800000	7171200	13800000	15600000	5522000	7959700	23700000	30900000
0	585460	1179800	1421700	0	1519000	2003600	0
4708600	12200000	19500000	22400000	4390300	7334100	11700000	0
2971100	4021200	0	0	1784400	2613600	4585800	0
3554400	3375500	4056000	4283300	2776600	2377700	3421400	0
1794400	7228100	7196300	15900000	8833100	14300000	9233200	24500000
2119200	1758800	2713400	4362800	961130	1412800	4114100	754270
3208500	9854800	8104300	1.10E+07	3911300	5030100	6835100	0
5899600	0	2541200	3918800	0	0	1379200	0
2977900	2789800	3641000	0	863200	0	0	0
1201600	2813200	1484900	2046500	1228400	1435400	2388900	0
3911500	0	0	0	0	0	0	0
8618500	26600000	22900000	42100000	12700000	16800000	14300000	0
11100000	0	0	0	0	0	0	0
4418400	15300000	6387200	19100000	4299000	5379500	6462300	0
4700900	5953000	8588500	3664900	2654800	4824600	6349500	0
1.70E+07	27200000	43600000	65300000	5497700	20100000	37200000	0
543000	0	209250	0	0	0	334400	0
3997700	0	0	1511200	0	0	1895700	0
5523800	10500000	10900000	23200000	3601700	4429800	9175800	0
13500000	6958200	7241900	0	4859600	9561700	7828700	0
3335100	4602200	2706200	0	3723100	6032300	5308300	0
579860	2552900	2051500	4417200	885920	1177300	3290900	0
0	0	0	0	0	0	0	0
5869400	16400000	12800000	1.90E+07	4918100	10100000	12800000	0
7494500	0	1.10E+07	6005600	4251000	6614600	7923400	0

3888200	16500000	3807900	26600000	2227000	4258400	24300000	0
5198500	2731900	4679800	5857800	0	3305200	4683300	0
578000	1067100	0	0	513390	0	1029300	0
6204600	20300000	17400000	20600000	4015200	3585900	9549300	4691300
0	7342100	0	0	0	1550100	1652000	0
6272100	7631900	8629300	13700000	3360100	4583600	8319100	0
5989900	3185400	2438200	4538300	0	0	0	0
1719800	3674800	0	5029300	0	0	0	0
0	0	0	0	0	0	0	0
781030	538260	459130	980210	261580	0	862790	0
2011500	0	1154900	3301600	488740	0	1183900	0
371280	0	0	4464000	0	0	0	0
3422300	3761100	0	6518500	0	0	0	834560
0	0	0	0	0	0	3467600	0
6028300	4526600	5797600	17100000	2674800	2299400	13800000	0
3841100	6566200	6059900	9845100	1881100	2783600	6773000	0
1256900	9225500	16300000	4263400	1213300	8809300	5816200	0
1747200	4094400	4422800	6126600	3217300	1768300	0	0
3753300	15100000	12200000	10600000	5274900	0	0	0
0	0	0	0	0	0	0	0
7380500	0	0	0	0	0	0	0
2424700	2.00E+07	15500000	7524900	7394900	8529500	14900000	0
4435800	9485900	2936600	5128300	1242800	2584800	2729100	0
11700000	22100000	14800000	19700000	3076300	8955900	2202400	4771100
1153900	2041300	5089400	6615400	1318100	5837300	5160700	0
2172300	2619200	0	0	0	1814800	4440100	0
4633200	4053200	5094400	15300000	2294200	2374300	6036300	0
17500000	13900000	19700000	28300000	1635800	2283000	0	0
2076700	2573600	488140	4155200	2129800	3405500	0	0
3852200	4128800	3114300	5790500	1340500	0	0	0
3753600	12300000	9845300	18900000	3395400	7989400	9468200	0
820570	1042700	0	2107100	1214100	671150	1127900	0
0	0	0	2734900	0	1535700	2406800	0
798900	8766600	4300900	17600000	4203100	6498300	6929500	6874500
0	7875000	0	0	3055300	1795600	6435900	0
0	16500000	4038500	0	3861400	4013200	2936400	0
35300000	4.50E+07	0	0	7145900	9763300	59600000	0
0	3780100	1173200	2907000	423550	3705700	4251500	0
0	2816200	4868400	1.40E+07	2041400	0	0	0
3724100	0	0	0	0	0	0	2543200
0	0	0	0	0	0	0	0
0	3672700	5168100	5897600	3141600	2262100	0	0
3141900	0	0	0	0	0	0	0
620650	1601900	5339000	7162900	3136900	4326100	9075700	0
2032000	2666600	0	4457600	5046000	8757300	3380800	5107400
2728900	0	0	0	0	0	0	0
0	2666600	1782600	10200000	4651500	6178800	11900000	0

2055000	0	0	0	0	0	0	0
0	0	0	0	0	0	0	0
2309000	3610200	0	0	751510	1756500	0	0
1063800	2626600	406300	1152500	388010	775830	2700700	0
1758000	14900000	14200000	3.20E+07	4172500	0	1669800	0
3631100	4632900	4359400	4825000	0	0	2625600	0
2045900	8409700	6915100	12400000	2989000	902680	3943700	0
1400500	1683500	6905800	2770700	408440	0	958000	10700000
0	0	0	0	0	0	0	0
8251800	0	0	0	996620	949080	11400000	0
6234900	3740500	3933800	0	1682700	0	0	0
1787500	0	0	4966600	0	0	3988300	0
7252900	22300000	5904400	7142700	9328700	9584900	18300000	0
0	0	0	0	0	0	0	0
5288400	7073500	16400000	19300000	6328900	9112400	10400000	9881700
0	5231700	9907700	7980600	1165500	1838000	5707800	887960
0	409880	0	0	0	0	0	0
0	0	0	0	0	0	0	0
7674900	4366600	4878200	14500000	0	0	3739700	0
0	0	0	0	0	0	0	0
0	0	0	0	0	0	0	0
12100000	6062900	0	8438700	3343700	0	5484800	0
8207600	8487900	12300000	0	0	3155100	10400000	0
3017600	6146900	4765900	1.00E+07	2507900	0	6178700	0
0	0	0	0	0	0	0	0
2935600	0	0	0	0	0	0	0
9898000	0	0	0	0	7347400	12600000	0
1859700	353720	386020	0	549780	419330	771390	0
22600000	37800000	27300000	51500000	25900000	22500000	39100000	1.07E+08
0	0	0	0	0	0	0	0
0	0	0	0	0	0	0	0
0	0	2150200	0	1217500	730810	2424500	0
1369200	7830100	7524500	11300000	0	0	0	0
4044900	0	0	0	0	0	4455000	0
0	4624700	7254300	5849100	0	2608100	4263900	0
3270700	8096200	8903200	15200000	841220	4720300	12200000	0
1809200	342020	238980	609940	0	350120	911100	0
1806900	8238500	7935000	17500000	3228100	3780200	5976800	248680
0	0	0	0	0	0	0	0
1553100	15900000	9865200	5342600	2760400	3337300	1973400	0
2284800	2878300	5083300	4635200	1139600	2039100	3553500	0
0	0	0	0	0	0	0	0
0	0	3465000	1848400	630300	0	1329200	0
7358900	10500000	5812800	22900000	2677500	4570700	14200000	0
0	0	0	4028600	0	0	0	0
0	0	0	0	0	0	0	0
0	2690500	3067600	0	1037800	2933900	3121800	0

2232000	1.00E+07	9969700	8060300	1255300	2817900	11700000	0
0	0	0	0	0	0	0	0
3709700	0	0	0	0	0	0	0
631810	5118000	3281600	6154800	0	1826600	977350	0
2434200	0	0	0	0	1778500	3098600	0
6716900	1246700	0	10200000	1461200	0	1797400	0
5660900	6612900	6812300	13600000	0	0	4578800	0
0	0	0	556180	0	0	0	0
1423300	0	0	0	0	0	0	0
665570	1477300	0	2210200	2151200	878050	1638600	0
3555000	0	5212100	0	0	1776100	11100000	0
0	0	0	0	0	0	0	0
1260500	0	0	0	0	0	0	0
2530600	0	0	0	0	0	0	2364900
1033400	8779000	0	0	0	0	930760	0
0	0	792450	0	3502100	0	2133600	0
2624200	6534500	4796300	8016400	6966400	6457800	9889400	0
3973900	9333100	10700000	13800000	0	5198700	6903000	0
0	0	0	0	0	0	0	0
3061400	4949300	0	4718800	2568900	2642600	5348100	0
0	0	0	0	0	0	0	0
0	0	0	0	0	0	0	0
0	0	0	0	0	0	0	0
0	0	0	5626000	6233000	0	6092500	12500000
0	0	0	0	0	0	0	0
5392300	5328600	0	1.30E+07	1581900	0	6300000	0
4762800	0	4012700	0	0	0	2234600	0
620470	0	0	0	0	0	2169500	0
0	0	0	0	0	0	0	0
0	183710	759910	0	1658100	2173000	2920400	0
4219000	5528900	1536300	8015800	3878400	0	6725600	0
674470	6160300	876200	0	0	2820400	2528900	0
2278800	5798600	4359900	7425600	0	2237100	0	0
0	0	0	0	0	0	0	0
3827700	0	0	0	0	0	8329500	0
3942700	9203700	2241800	14100000	1880000	3332400	5654600	0
0	15600000	3877700	19700000	5157300	2906000	5403500	0
1195600	0	0	1008500	424050	512330	0	0
4137200	0	0	0	0	0	0	0
0	0	0	0	0	0	0	0
1097500	0	0	2900200	0	0	0	0
19200000	41100000	41700000	41700000	14400000	24900000	29800000	0
0	0	0	0	0	0	0	0
1742800	2912500	4782200	6618000	2320400	1901900	3105300	0
0	0	0	0	0	0	0	0
1196400	2188400	0	4061500	1169800	1658100	2324400	0
1060900	0	0	0	0	0	0	0

0	9092700	5676600	6549700	1788200	3919000	2933100	2574400
0	0	0	0	0	0	0	0
0	0	0	0	0	0	0	0
1292800	1993800	434840	2954600	869150	0	1593500	3280200
1946000	2962900	2781700	6354000	0	0	2857200	4197500
1693200	0	0	2480300	0	0	0	0
0	0	0	0	0	0	0	0
1945400	0	0	0	0	0	0	0
4404200	2040600	0	0	1104500	440640	557260	0
2547500	4819500	0	5520000	0	1996800	6180700	0
2386600	3785200	1209800	0	0	1282300	2646500	0
2899800	0	0	0	1163600	2767700	6769300	0
0	0	0	0	0	0	0	0
0	0	0	0	0	0	0	0
0	11500000	0	0	0	0	0	0
2739800	0	0	4230000	1656700	4066800	12300000	0
2070700	4028100	4641500	4817200	2433000	1186400	0	0
0	0	0	0	0	0	0	0
1310100	0	0	0	0	0	0	0
643970	3577200	3277700	2892500	2381200	2692900	3784300	0
363930	0	0	0	0	0	0	0
2819200	1330000	0	2303800	0	478330	1510000	0
0	0	0	366970	0	0	472570	0
1675800	456640	531370	531560	745630	1112800	1955500	0
0	2326700	0	2893300	0	0	0	0
821300	0	0	0	0	0	0	0
0	0	0	0	0	0	0	0
0	0	0	370700	0	0	278350	0
987300	2333500	0	2768800	967860	568410	1630200	0
0	0	0	4546200	0	0	0	0
0	0	0	0	0	0	0	0
0	0	0	0	0	0	0	0
0	0	0	0	741790	0	4557000	0
3253100	0	0	0	0	0	0	0
0	0	0	0	0	0	0	0
3485400	14800000	8019300	10100000	6636700	8708600	8551400	0
217970	0	0	0	0	1819000	2077300	0
1602600	0	0	0	0	0	0	0
620910	3573400	925110	755600	1027600	884940	2780900	0
372230	0	0	0	0	0	0	0
3305900	0	0	0	0	0	0	0
0	0	0	0	0	0	0	0
0	7476300	0	0	0	0	0	0
2853300	0	1198800	0	0	0	1637900	0
6872100	8078000	12500000	10700000	0	9974300	6941500	2627600
0	0	5109500	0	0	2928100	0	0
0	0	0	0	0	0	0	0

8006600	0	0	6421600	1308200	2784400	3559300	0
227540	0	0	0	0	0	0	0
0	1947100	552290	2052400	1192100	1573200	604650	0
0	5654200	3550500	0	0	0	5968300	0
0	0	0	0	0	0	0	0
0	0	0	0	0	0	0	0
7081100	4202400	6203600	4168700	2718900	0	2567900	0
981340	3500800	2148800	1760900	1802400	1499800	5056200	0
0	941270	0	0	897900	0	0	0
1288900	4279300	6448300	3088000	0	0	0	0
524220	549830	2784000	3894100	2064500	2584400	5975600	0
0	0	0	0	0	0	0	0
626000	3523600	0	2268600	858080	898590	2434800	0
2147900	0	0	0	0	0	0	1496500
0	0	0	0	0	0	0	0
3774000	3430700	0	4879800	0	0	0	0
1141900	0	0	0	0	0	0	0
561870	907410	948420	0	557100	550580	2129100	0
1645100	0	0	0	0	0	1536000	0
0	0	0	0	0	0	0	0
0	9778900	0	10400000	2581900	4984000	8080900	0
2059400	1201800	877200	4246400	2214400	3422300	4241600	0
1738200	3027600	0	8530200	0	0	0	0
16300000	32800000	37500000	40900000	10300000	14600000	2.40E+07	0
0	3443000	3279100	1813400	0	2075300	4382200	0
0	1942000	2753600	5935500	1695400	1497800	3237200	0
0	0	0	0	0	0	0	0
0	0	0	0	0	0	0	0
0	0	0	1338400	0	0	0	0
2492700	0	0	0	0	0	0	0
0	0	0	0	0	0	0	0
2995700	3944900	0	4517600	669760	1357900	4484300	0
0	1127100	0	0	271480	574530	909500	0
716390	647240	0	0	0	1172400	1285900	0
0	0	0	0	0	0	0	0
0	0	0	0	0	0	922930	0
0	0	0	0	0	0	0	0
0	0	0	0	0	0	0	0
0	0	0	0	0	0	0	0
0	946960	0	0	0	640100	1101600	0
0	0	751860	0	0	1417300	2795800	0
0	6343100	0	0	2637300	3285000	3789900	0
0	0	0	0	0	0	0	0
1790200	0	0	0	0	0	5124700	0
0	0	0	4233900	0	0	0	0
0	3290600	0	0	1785000	0	4638500	0
3260500	0	0	0	0	0	2796800	0

0	0	0	0	0	0	0	0
0	0	0	0	0	0	0	0
815050	4701500	8630800	10500000	0	3850000	5500300	0
2939200	0	0	0	0	0	0	0
0	0	0	5681600	522880	659360	0	0
3526000	0	0	0	1627400	2436500	3343200	0
0	0	0	0	0	0	0	0
11500000	9952600	6518100	14800000	0	0	0	0
0	0	0	0	0	0	0	0
0	0	0	0	0	0	0	0
0	0	0	0	0	0	0	0
0	0	0	0	0	0	0	0
0	0	0	0	0	0	0	0
0	0	0	0	0	0	0	0
0	0	0	0	0	0	0	0
0	0	0	0	0	0	0	0
0	0	0	0	0	0	0	0
969270	0	0	0	0	0	0	0
0	4646500	0	5160700	1465500	3704000	3614200	0
72500000	32700000	22200000	48100000	6.80E+07	81600000	1.60E+08	73500000
0	0	0	0	0	0	4064000	0
0	0	0	0	0	0	0	0
2428100	0	0	0	0	0	0	1264500
0	2963700	0	4074100	1091900	252750	2603300	0
0	0	0	0	0	0	1472100	0
0	0	0	0	0	0	0	0
835030	1970500	0	2864100	1934800	1241000	7399600	0
0	848900	4480400	5729900	0	322100	511310	0
0	0	0	0	0	0	0	0
6198300	0	0	0	0	0	0	0
0	0	0	0	0	0	0	0
4215700	9915100	5694900	671990	3590200	5006700	9343900	0
0	4950600	6954500	8352500	2573200	2695800	4852500	0
0	0	0	0	0	0	0	0
0	0	0	0	0	0	0	0
0	0	0	0	0	0	0	0
0	6513900	7121600	8801000	273660	3460200	567710	0
0	0	0	0	0	0	0	0
0	2064900	1586500	2623000	1504600	0	600740	0
0	0	0	0	0	0	0	0
919060	0	0	0	0	0	0	0
3570100	0	0	8797300	4183700	5205100	9392900	0
1843100	2492400	4865700	5246900	2221600	1617600	4566500	0
3451200	3041900	0	0	0	0	2859600	0
0	0	0	0	0	0	0	0
0	0	0	0	0	0	0	0
2311100	0	743730	0	0	2225800	2521600	0

0	0	0	0	0	0	0	0
0	0	0	0	0	0	0	0
29300000	4.50E+07	0	0	0	0	51700000	0
4486900	0	0	0	247760	0	2621800	0
0	0	0	0	0	0	0	0
0	0	0	0	0	0	0	0
0	0	0	0	0	0	0	0
0	0	0	0	0	0	0	0
2.30E+07	41800000	28700000	11300000	8547200	18300000	10400000	0
0	0	0	0	779630	1670600	1438400	0
0	0	0	0	0	0	0	0
0	0	0	0	0	0	0	1364600
0	0	0	0	0	0	0	0
0	0	0	0	0	37600000	293240	0
0	0	0	0	0	0	0	0
0	0	0	0	0	0	0	0
0	0	0	0	0	0	0	0
0	0	0	0	0	0	0	0
44200000	99900000	8.20E+07	1.34E+08	50300000	63100000	1.04E+08	8683300
0	0	0	0	0	0	0	0
0	0	0	0	0	0	0	0
5870600	48100000	36700000	46500000	3803700	4896600	21600000	0
0	0	0	0	0	0	0	0
0	0	0	0	0	0	0	0
NA	1.60E+09	1.41E+09	1.87E+09	NA	NA	NA	4.42E+08
NA	94800000	1.13E+09	1.84E+08	NA	NA	NA	1.26E+09
NA	1.46E+10	1.20E+10	2.35E+10	NA	NA	NA	4.17E+09
NA	4.32E+08	1.01E+09	7.90E+08	NA	NA	NA	5.59E+08
NA	4.31E+08	4.85E+08	7.73E+08	NA	NA	NA	46800000
NA	1.92E+09	2.00E+09	2.84E+09	NA	NA	NA	4.26E+08
NA	4.35E+08	4.97E+08	6.10E+08	NA	NA	NA	72100000
NA	1.05E+09	1.13E+09	1.27E+09	NA	NA	NA	2.74E+09
NA	1.61E+08	1.48E+08	1.95E+08	NA	NA	NA	0
NA	3.83E+08	4.58E+08	5.73E+08	NA	NA	NA	69700000
NA	0	2.60E+07	31100000	NA	NA	NA	2032100
NA	2.45E+08	2.16E+08	3.45E+08	NA	NA	NA	3811500
NA	1.62E+08	1.60E+08	1.54E+08	NA	NA	NA	1.37E+08
NA	4.37E+08	4.31E+08	5.69E+08	NA	NA	NA	1.13E+08
NA	1.34E+08	1.28E+08	1.67E+08	NA	NA	NA	25400000
NA	1.74E+08	1.44E+08	1.43E+08	NA	NA	NA	24200000
NA	3.96E+08	4.41E+08	5.22E+08	NA	NA	NA	48300000
NA	37500000	43400000	3.30E+07	NA	NA	NA	31800000
NA	57200000	96700000	1.29E+08	NA	NA	NA	50500000
NA	1.17E+08	85700000	1.36E+08	NA	NA	NA	14100000
NA	1.00E+08	68900000	95800000	NA	NA	NA	0
NA	1.97E+08	1.83E+08	2.43E+08	NA	NA	NA	52300000
NA	1.20E+07	4243800	19600000	NA	NA	NA	7512600

NA	52100000	2.20E+07	64700000	NA	NA	NA	0
NA	1.27E+08	83600000	1.28E+08	NA	NA	NA	2876200
NA	92400000	1.44E+08	2.36E+08	NA	NA	NA	36500000
NA	59600000	30100000	1.40E+08	NA	NA	NA	5010100
NA	0	0	0	NA	NA	NA	0
NA	33600000	31500000	38100000	NA	NA	NA	745420
NA	3240200	3905300	7236600	NA	NA	NA	0
NA	59300000	5.10E+07	68100000	NA	NA	NA	3837400
NA	26800000	17800000	38800000	NA	NA	NA	66400000
NA	0	0	17100000	NA	NA	NA	0
NA	66200000	22100000	0	NA	NA	NA	4032100
NA	11400000	26500000	29300000	NA	NA	NA	0
NA	28800000	16400000	10600000	NA	NA	NA	0
NA	27500000	4.40E+07	38700000	NA	NA	NA	0
NA	0	0	0	NA	NA	NA	21100000
NA	9.30E+07	1.02E+08	1.60E+08	NA	NA	NA	27200000
NA	17500000	30900000	35200000	NA	NA	NA	0
NA	72700000	53300000	77100000	NA	NA	NA	10800000
NA	0	0	0	NA	NA	NA	0
NA	0	0	0	NA	NA	NA	0
NA	26700000	17200000	47100000	NA	NA	NA	0
NA	49300000	6600900	68200000	NA	NA	NA	0
NA	27700000	37300000	50500000	NA	NA	NA	0
NA	0	1475500	0	NA	NA	NA	0
NA	98700000	1.77E+08	1.86E+08	NA	NA	NA	1.80E+08
NA	68700000	66300000	1.05E+08	NA	NA	NA	0
NA	0	2273700	3474800	NA	NA	NA	0
NA	0	2196400	0	NA	NA	NA	0
NA	18500000	15200000	26600000	NA	NA	NA	684570
NA	32900000	26200000	24500000	NA	NA	NA	0
NA	11500000	6301400	11800000	NA	NA	NA	2131400
NA	50900000	5.40E+07	72100000	NA	NA	NA	0
NA	0	427740	15400000	NA	NA	NA	0
NA	0	0	0	NA	NA	NA	0
NA	0	1083100	0	NA	NA	NA	0
NA	68700000	60700000	59300000	NA	NA	NA	0
NA	555180	0	0	NA	NA	NA	0
NA	0	5445400	0	NA	NA	NA	0
NA	0	0	0	NA	NA	NA	1.37E+09
NA	12700000	10700000	15900000	NA	NA	NA	0
NA	0	0	0	NA	NA	NA	0
NA	0	0	0	NA	NA	NA	0
NA	4262600	2704000	2012800	NA	NA	NA	301000
NA	1.18E+08	5.79E+08	2.01E+08	NA	NA	NA	4.46E+08
NA	0	0	0	NA	NA	NA	0
NA	9286800	30200000	16700000	NA	NA	NA	21400000
NA	0	0	0	NA	NA	NA	8011200

NA	0	0	0	NA	NA	NA	0
NA	0	0	0	NA	NA	NA	0
NA	6534700	7447200	16100000	NA	NA	NA	0
NA	3264800	9371300	21300000	NA	NA	NA	0
NA	14100000	1.80E+07	22700000	NA	NA	NA	6526300
NA	0	36600000	36300000	NA	NA	NA	0
NA	0	0	0	NA	NA	NA	0
NA	8753000	8987500	25700000	NA	NA	NA	1410900
NA	0	0	10400000	NA	NA	NA	0
NA	14800000	23500000	27300000	NA	NA	NA	0
NA	10700000	0	10600000	NA	NA	NA	0
NA	23400000	23800000	26300000	NA	NA	NA	7392800
NA	0	0	0	NA	NA	NA	674850
NA	4426800	4424400	1060900	NA	NA	NA	0
NA	17600000	21300000	25500000	NA	NA	NA	0
NA	0	0	0	NA	NA	NA	0
NA	12400000	7130400	1.20E+07	NA	NA	NA	0
NA	0	0	0	NA	NA	NA	0
NA	2841700	4036500	11100000	NA	NA	NA	0
NA	2772900	2291000	1.60E+07	NA	NA	NA	0
NA	6037200	16800000	5707800	NA	NA	NA	0
NA	0	0	0	NA	NA	NA	0
NA	0	0	0	NA	NA	NA	1.29E+08
NA	0	0	0	NA	NA	NA	0
NA	0	0	0	NA	NA	NA	0
NA	0	0	4982700	NA	NA	NA	0
NA	0	0	1410100	NA	NA	NA	0
NA	16500000	4995200	27700000	NA	NA	NA	0
NA	0	0	1.38E+08	NA	NA	NA	4.46E+08
NA	0	0	0	NA	NA	NA	0
NA	2432700	3933900	5241700	NA	NA	NA	0
NA	0	10500000	6167300	NA	NA	NA	0
NA	0	0	0	NA	NA	NA	0
NA	0	0	0	NA	NA	NA	0
NA	471200	378490	920900	NA	NA	NA	0
NA	9237200	13600000	23600000	NA	NA	NA	0
NA	0	5566500	0	NA	NA	NA	0
NA	0	848500	1264100	NA	NA	NA	0
NA	6986800	4672100	4038100	NA	NA	NA	1545200
NA	4982200	915270	9140900	NA	NA	NA	0
NA	0	0	157520	NA	NA	NA	3591400
NA	0	0	0	NA	NA	NA	0
NA	0	0	0	NA	NA	NA	0
NA	4156300	0	5008400	NA	NA	NA	0
NA	23800000	19600000	10300000	NA	NA	NA	0
NA	378170	596120	628240	NA	NA	NA	0
NA	1287500	0	821460	NA	NA	NA	0

NA	93100000	0	1.25E+08	NA	NA	NA	0
NA	9836700	9173100	22400000	NA	NA	NA	1618000
NA	10900000	12400000	19500000	NA	NA	NA	6029900
NA	0	0	0	NA	NA	NA	864030
NA	6564700	7180200	10700000	NA	NA	NA	0
NA	0	10300000	12500000	NA	NA	NA	0
NA	0	0	3963100	NA	NA	NA	8613700
NA	0	0	0	NA	NA	NA	0
NA	14900000	13100000	6313700	NA	NA	NA	0
NA	0	0	0	NA	NA	NA	5603900
NA	0	0	0	NA	NA	NA	0
NA	22800000	22100000	27400000	NA	NA	NA	41400000
NA	2257100	2167400	3644600	NA	NA	NA	0
NA	9065700	3454100	5554300	NA	NA	NA	0
NA	5836500	52700000	6618700	NA	NA	NA	0
NA	9344100	11700000	13700000	NA	NA	NA	1406700
NA	7547800	6170700	12500000	NA	NA	NA	0
NA	0	0	685710	NA	NA	NA	0
NA	1685300	9131800	2604800	NA	NA	NA	0
NA	0	0	0	NA	NA	NA	0
NA	0	0	0	NA	NA	NA	0
NA	4983500	1182800	6345500	NA	NA	NA	0
NA	5605500	1203500	7578400	NA	NA	NA	241450
NA	4.50E+08	4.50E+08	5.24E+08	NA	NA	NA	0
NA	0	0	0	NA	NA	NA	0
NA	0	0	4473700	NA	NA	NA	0
NA	0	0	0	NA	NA	NA	0
NA	4279300	4206100	0	NA	NA	NA	0
NA	0	8600100	0	NA	NA	NA	0
NA	0	0	0	NA	NA	NA	0
NA	0	0	0	NA	NA	NA	0
NA	0	0	0	NA	NA	NA	0
NA	0	7032200	7116500	NA	NA	NA	0
NA	0	0	0	NA	NA	NA	4294300
NA	0	0	0	NA	NA	NA	475860
NA	0	0	0	NA	NA	NA	0
NA	4137400	0	6816700	NA	NA	NA	0
NA	0	3177300	0	NA	NA	NA	5245000
NA	1728400	0	3021900	NA	NA	NA	0
NA	0	0	0	NA	NA	NA	0
NA	0	0	5224400	NA	NA	NA	0
NA	626960	663620	752280	NA	NA	NA	503770
NA	2630200	0	0	NA	NA	NA	0
NA	0	0	0	NA	NA	NA	0
NA	4273100	4083300	4798800	NA	NA	NA	0
NA	0	0	0	NA	NA	NA	0
NA	7754900	8581500	146950	NA	NA	NA	336770

NA	392480	0	0	NA	NA	NA	0
NA	4548900	2138100	10600000	NA	NA	NA	0
NA	0	2104700	0	NA	NA	NA	0
NA	5552000	11400000	15200000	NA	NA	NA	2734400
NA	0	0	0	NA	NA	NA	0
NA	6279200	4478200	6257700	NA	NA	NA	0
NA	7852900	5480100	9174000	NA	NA	NA	0
NA	694900	3567700	3975200	NA	NA	NA	143460
NA	0	0	0	NA	NA	NA	0
NA	0	0	0	NA	NA	NA	0
NA	0	0	0	NA	NA	NA	0
NA	2618300	783090	12200000	NA	NA	NA	0
NA	6857800	0	8186400	NA	NA	NA	0
NA	0	0	0	NA	NA	NA	0
NA	0	0	0	NA	NA	NA	0
NA	0	0	0	NA	NA	NA	0
NA	0	0	0	NA	NA	NA	0
NA	3068000	6984000	8890100	NA	NA	NA	0
NA	4686800	5057700	9905000	NA	NA	NA	0
NA	1862700	7104600	2729300	NA	NA	NA	0
NA	708340	0	1314300	NA	NA	NA	0
NA	0	0	0	NA	NA	NA	19100000
NA	0	9599400	0	NA	NA	NA	0
NA	0	0	0	NA	NA	NA	0
NA	0	0	0	NA	NA	NA	0
NA	0	12900000	8483300	NA	NA	NA	0
NA	0	0	0	NA	NA	NA	0
NA	5673300	0	1821100	NA	NA	NA	0
NA	0	0	0	NA	NA	NA	0
NA	3286600	0	5298000	NA	NA	NA	0
NA	471840	0	2902500	NA	NA	NA	0
NA	0	0	0	NA	NA	NA	0
NA	1732200	2026700	1811700	NA	NA	NA	0
NA	0	0	0	NA	NA	NA	0
NA	0	0	0	NA	NA	NA	0
NA	0	0	0	NA	NA	NA	0
NA	0	0	0	NA	NA	NA	6640900
NA	0	0	0	NA	NA	NA	0
NA	0	0	0	NA	NA	NA	6751800
NA	0	0	0	NA	NA	NA	0
NA	3713700	3679400	6057700	NA	NA	NA	0
NA	1290700	1103700	1437700	NA	NA	NA	0
NA	3886700	4692200	0	NA	NA	NA	0
NA	0	0	0	NA	NA	NA	0
NA	0	0	0	NA	NA	NA	1405700
NA	8763300	371230	596210	NA	NA	NA	0
NA	5327100	4913700	10700000	NA	NA	NA	0

NA	0	6426400	0	NA	NA	NA	0
NA	0	0	4670300	NA	NA	NA	0
NA	3502400	3739400	5077200	NA	NA	NA	0
NA	0	0	0	NA	NA	NA	0
NA	700180	728860	1371200	NA	NA	NA	0
NA	7545500	6391900	0	NA	NA	NA	0
NA	0	0	0	NA	NA	NA	0
NA	0	0	0	NA	NA	NA	0
NA	4931100	5177900	3919800	NA	NA	NA	0
NA	0	4369600	1.10E+07	NA	NA	NA	0
NA	0	0	0	NA	NA	NA	0
NA	0	0	0	NA	NA	NA	0
NA	0	0	0	NA	NA	NA	0
NA	11500000	0	1.60E+07	NA	NA	NA	0
NA	0	0	4466400	NA	NA	NA	0
NA	0	0	0	NA	NA	NA	0
NA	0	403970	1059900	NA	NA	NA	0
NA	0	0	0	NA	NA	NA	0
NA	0	0	0	NA	NA	NA	0
NA	0	0	0	NA	NA	NA	0
NA	0	0	0	NA	NA	NA	0
NA	468150	0	1205200	NA	NA	NA	0
NA	2027300	0	3006000	NA	NA	NA	27800000
NA	5291300	0	0	NA	NA	NA	0
NA	0	0	0	NA	NA	NA	0
NA	0	0	0	NA	NA	NA	0
NA	0	0	0	NA	NA	NA	0
NA	1098500	1539400	1952600	NA	NA	NA	0
NA	0	0	0	NA	NA	NA	0
NA	0	0	0	NA	NA	NA	0
NA	0	0	0	NA	NA	NA	38600000
NA	1715000	4283700	9188700	NA	NA	NA	0
NA	0	0	0	NA	NA	NA	0
NA	0	0	0	NA	NA	NA	0
NA	682680	1667500	0	NA	NA	NA	0
NA	0	3094600	0	NA	NA	NA	0
NA	0	0	0	NA	NA	NA	0
NA	2469900	0	0	NA	NA	NA	0
NA	0	0	0	NA	NA	NA	0
NA	0	0	0	NA	NA	NA	0
NA	924280	1174900	1993200	NA	NA	NA	0
NA	0	0	0	NA	NA	NA	0
NA	0	0	0	NA	NA	NA	0
NA	0	0	0	NA	NA	NA	0
NA	430380	331370	641740	NA	NA	NA	0
NA	1022700	0	1632000	NA	NA	NA	0
NA	0	3821900	0	NA	NA	NA	0

NA	0	0	0	NA	NA	NA	0
NA	0	0	0	NA	NA	NA	0
NA	8857200	0	3375000	NA	NA	NA	0
NA	0	224130	0	NA	NA	NA	0
NA	0	0	0	NA	NA	NA	0
NA	0	0	0	NA	NA	NA	0
NA	0	96900000	0	NA	NA	NA	13900000
NA	0	0	0	NA	NA	NA	0
NA	1300800	13700000	1951300	NA	NA	NA	0
NA	0	0	0	NA	NA	NA	0
NA	0	0	0	NA	NA	NA	0
NA	2194500	1944700	0	NA	NA	NA	0
NA	0	0	0	NA	NA	NA	0
NA	0	1873600	0	NA	NA	NA	0
NA	775830	3886200	0	NA	NA	NA	0
NA	0	0	0	NA	NA	NA	0
NA	0	0	0	NA	NA	NA	0
NA	3837000	808530	1316700	NA	NA	NA	0
NA	0	0	0	NA	NA	NA	0
NA	0	0	4230000	NA	NA	NA	16200000
NA	0	0	0	NA	NA	NA	0
NA	0	0	0	NA	NA	NA	0
NA	7286200	5480100	8118800	NA	NA	NA	0
NA	0	0	0	NA	NA	NA	0
NA	0	0	0	NA	NA	NA	0
NA	0	0	0	NA	NA	NA	0
NA	0	0	0	NA	NA	NA	0
NA	1.90E+07	9999900	23500000	NA	NA	NA	0
NA	0	0	0	NA	NA	NA	0
NA	0	0	0	NA	NA	NA	0
NA	0	0	0	NA	NA	NA	0
NA	0	0	0	NA	NA	NA	0
NA	0	0	0	NA	NA	NA	0
NA	0	0	0	NA	NA	NA	0
NA	0	0	0	NA	NA	NA	0
NA	0	0	0	NA	NA	NA	0
NA	0	0	0	NA	NA	NA	0
NA	0	0	0	NA	NA	NA	0
NA	0	0	0	NA	NA	NA	0
NA	5979500	10100000	11500000	NA	NA	NA	0
NA	0	0	0	NA	NA	NA	0
NA	0	0	0	NA	NA	NA	0
NA	0	0	0	NA	NA	NA	0
NA	0	0	0	NA	NA	NA	0
NA	0	0	0	NA	NA	NA	0
NA	0	0	0	NA	NA	NA	0
NA	0	0	0	NA	NA	NA	0

NA	0	0	0	NA	NA	NA	0
NA	0	0	0	NA	NA	NA	0
NA	0	0	0	NA	NA	NA	0
NA	0	13100000	0	NA	NA	NA	0
NA	0	0	0	NA	NA	NA	0
NA	0	0	0	NA	NA	NA	0
NA	0	0	0	NA	NA	NA	0
NA	0	0	2580100	NA	NA	NA	0
NA	0	0	0	NA	NA	NA	0
NA	0	0	0	NA	NA	NA	0
NA	0	0	0	NA	NA	NA	0
NA	23700000	34800000	41800000	NA	NA	NA	0
NA	3210500	0	6597600	NA	NA	NA	0
NA	0	0	0	NA	NA	NA	0
NA	0	9599400	0	NA	NA	NA	0
NA	1592100	2051500	2346900	NA	NA	NA	0
NA	0	0	0	NA	NA	NA	0
NA	0	0	0	NA	NA	NA	0
NA	0	0	0	NA	NA	NA	0
NA	467970	354400	0	NA	NA	NA	0
NA	0	0	0	NA	NA	NA	0
NA	2432700	3111500	4251300	NA	NA	NA	0
NA	0	0	0	NA	NA	NA	0
NA	0	0	0	NA	NA	NA	0
NA	0	0	0	NA	NA	NA	0
NA	0	0	0	NA	NA	NA	0
NA	0	0	0	NA	NA	NA	0
NA	0	0	0	NA	NA	NA	0
NA	0	0	0	NA	NA	NA	0
NA	0	0	0	NA	NA	NA	0
NA	0	0	0	NA	NA	NA	0
NA	0	0	0	NA	NA	NA	0
NA	0	0	0	NA	NA	NA	0
NA	0	0	0	NA	NA	NA	0
NA	0	0	0	NA	NA	NA	0
NA	0	0	0	NA	NA	NA	0
NA	0	0	0	NA	NA	NA	0
NA	0	0	0	NA	NA	NA	0
NA	0	0	0	NA	NA	NA	0
NA	8835600	0	0	NA	NA	NA	0
9.84E+08	NA	NA	NA	6.26E+08	7.73E+08	1.43E+09	NA
9.73E+09	NA	NA	NA	6.36E+09	8.21E+09	1.46E+10	NA
9.13E+08	NA	NA	NA	7.34E+08	1.06E+09	1.85E+09	NA
2.23E+08	NA	NA	NA	1.42E+08	4.87E+08	3.84E+08	NA
1.27E+08	NA	NA	NA	1.67E+08	2.34E+08	3.82E+08	NA
2.95E+08	NA	NA	NA	1.99E+08	2.55E+08	4.99E+08	NA
1.80E+08	NA	NA	NA	1.63E+08	2.41E+08	3.78E+08	NA

3.92E+08	NA	NA	NA	4.93E+08	3.25E+08	4.10E+08	NA
0	NA	NA	NA	3724800	0	1.20E+07	NA
1.04E+08	NA	NA	NA	55700000	47700000	1.10E+08	NA
9.10E+07	NA	NA	NA	8.70E+07	1.09E+08	2.26E+08	NA
8.10E+07	NA	NA	NA	7.20E+07	1.00E+08	1.45E+08	NA
47500000	NA	NA	NA	75400000	83800000	1.23E+08	NA
2.09E+08	NA	NA	NA	1.62E+08	2.67E+08	4.14E+08	NA
66100000	NA	NA	NA	61700000	89200000	1.42E+08	NA
1.42E+08	NA	NA	NA	19600000	46200000	90800000	NA
1.01E+08	NA	NA	NA	6.10E+07	78900000	1.35E+08	NA
1.52E+08	NA	NA	NA	1.39E+08	2.30E+08	3.22E+08	NA
74600000	NA	NA	NA	7456400	18900000	29700000	NA
53800000	NA	NA	NA	35700000	32100000	71400000	NA
2.60E+08	NA	NA	NA	1.68E+08	2.81E+08	4.74E+08	NA
31600000	NA	NA	NA	17700000	18100000	61400000	NA
90200000	NA	NA	NA	9449400	10700000	37200000	NA
27600000	NA	NA	NA	634840	906260	3646800	NA
1.04E+08	NA	NA	NA	25400000	73100000	1.53E+08	NA
3043000	NA	NA	NA	7010000	4653000	14200000	NA
89500000	NA	NA	NA	9761300	24100000	79100000	NA
74300000	NA	NA	NA	32400000	54900000	90200000	NA
3253200	NA	NA	NA	0	997120	3920300	NA
6188200	NA	NA	NA	4296600	1.10E+07	9149800	NA
12300000	NA	NA	NA	10100000	13100000	27900000	NA
26900000	NA	NA	NA	5355500	14900000	2.80E+07	NA
10300000	NA	NA	NA	14700000	14100000	29200000	NA
0	NA	NA	NA	0	0	0	NA
0	NA	NA	NA	2085000	2904900	11100000	NA
27200000	NA	NA	NA	27800000	45800000	60800000	NA
12700000	NA	NA	NA	8959100	11100000	10700000	NA
43500000	NA	NA	NA	3.60E+07	47800000	1.04E+08	NA
698560	NA	NA	NA	4704800	18200000	24900000	NA
12200000	NA	NA	NA	0	865850	2214500	NA
18800000	NA	NA	NA	2.30E+07	24600000	3.10E+07	NA
2.90E+07	NA	NA	NA	26100000	33100000	42600000	NA
30800000	NA	NA	NA	8312000	1.70E+07	31400000	NA
2719200	NA	NA	NA	8598100	6364200	12200000	NA
6731700	NA	NA	NA	0	0	0	NA
19800000	NA	NA	NA	1726000	6044600	9541100	NA
9615000	NA	NA	NA	0	0	0	NA
11600000	NA	NA	NA	6161900	10600000	9407300	NA
0	NA	NA	NA	0	563160	386870	NA
32200000	NA	NA	NA	8198500	13200000	26600000	NA
19600000	NA	NA	NA	21800000	29300000	47600000	NA
8367000	NA	NA	NA	0	0	0	NA
6008400	NA	NA	NA	2450200	0	3639800	NA
4743500	NA	NA	NA	12100000	5168600	24100000	NA

9756400	NA	NA	NA	10100000	12200000	14900000	NA
0	NA	NA	NA	0	0	0	NA
0	NA	NA	NA	0	0	0	NA
8341100	NA	NA	NA	3914600	10900000	16300000	NA
0	NA	NA	NA	0	0	0	NA
0	NA	NA	NA	0	0	0	NA
3.00E+07	NA	NA	NA	579940	1036700	1317800	NA
5476300	NA	NA	NA	8832800	9431500	14200000	NA
14400000	NA	NA	NA	2279500	3157000	14200000	NA
6636300	NA	NA	NA	5950800	10800000	27100000	NA
23600000	NA	NA	NA	25800000	3.50E+07	63800000	NA
14900000	NA	NA	NA	5123800	12800000	13300000	NA
3105700	NA	NA	NA	1595300	4350600	4553200	NA
5269400	NA	NA	NA	1452900	0	4210700	NA
11700000	NA	NA	NA	0	0	3979500	NA
0	NA	NA	NA	423080	620210	2160800	NA
6368400	NA	NA	NA	5001400	8502300	23500000	NA
4192700	NA	NA	NA	7528500	0	21700000	NA
578900	NA	NA	NA	2831600	3678100	813090	NA
14900000	NA	NA	NA	13800000	13300000	22700000	NA
3030400	NA	NA	NA	0	2916200	6245000	NA
4773000	NA	NA	NA	2731700	4824900	12100000	NA
1871800	NA	NA	NA	2149600	3668100	7307600	NA
2175100	NA	NA	NA	2148600	2643200	5418800	NA
8953200	NA	NA	NA	11200000	10400000	22800000	NA
0	NA	NA	NA	0	7855800	0	NA
25900000	NA	NA	NA	2.70E+07	35700000	59200000	NA
908840	NA	NA	NA	1910800	2040800	6476400	NA
1005700	NA	NA	NA	2578400	2709500	5140700	NA
16700000	NA	NA	NA	10200000	0	0	NA
1720900	NA	NA	NA	6242000	7508600	11700000	NA
0	NA	NA	NA	0	0	0	NA
6454300	NA	NA	NA	3340900	7073100	8954500	NA
3535800	NA	NA	NA	6946500	13400000	16800000	NA
13300000	NA	NA	NA	4488900	5309400	1.70E+07	NA
4757300	NA	NA	NA	0	0	1783100	NA
0	NA	NA	NA	0	0	0	NA
3292500	NA	NA	NA	4694100	4626900	12200000	NA
2722900	NA	NA	NA	3428900	3489300	4156800	NA
0	NA	NA	NA	0	903060	2673900	NA
0	NA	NA	NA	0	11400000	6753300	NA
0	NA	NA	NA	1065700	0	4174600	NA
6705400	NA	NA	NA	0	0	4369500	NA
0	NA	NA	NA	0	0	0	NA
17900000	NA	NA	NA	1542900	1431700	5439500	NA
3123000	NA	NA	NA	0	2573600	6659200	NA
7068300	NA	NA	NA	6535000	7622900	11400000	NA

2404100	NA	NA	NA	0	0	0	NA
391870	NA	NA	NA	0	330470	3440200	NA
2446200	NA	NA	NA	0	0	0	NA
4308800	NA	NA	NA	1980600	2378500	3958800	NA
0	NA	NA	NA	0	2336600	845350	NA
9544300	NA	NA	NA	6427700	7873600	11900000	NA
9319700	NA	NA	NA	4833100	4872000	0	NA
0	NA	NA	NA	3708900	1764800	601250	NA
0	NA	NA	NA	0	0	0	NA
0	NA	NA	NA	0	0	0	NA
5214600	NA	NA	NA	1541200	3652500	9208900	NA
1902600	NA	NA	NA	4403700	1476900	9594100	NA
476250	NA	NA	NA	1102900	3839400	0	NA
2375000	NA	NA	NA	0	2711300	1989900	NA
0	NA	NA	NA	0	0	0	NA
0	NA	NA	NA	0	0	0	NA
1883200	NA	NA	NA	1192700	3813900	3948000	NA
1774900	NA	NA	NA	0	0	0	NA
0	NA	NA	NA	648770	2402200	860830	NA
0	NA	NA	NA	0	0	0	NA
8861100	NA	NA	NA	8632700	10400000	27200000	NA
2121200	NA	NA	NA	0	0	0	NA
893760	NA	NA	NA	559620	669350	1991700	NA
8507200	NA	NA	NA	0	0	5775900	NA
0	NA	NA	NA	0	0	0	NA
9959300	NA	NA	NA	2024100	2288600	13100000	NA
0	NA	NA	NA	0	0	0	NA
0	NA	NA	NA	7371300	0	0	NA
7061000	NA	NA	NA	9487500	6782600	11800000	NA
0	NA	NA	NA	387640	928810	1233200	NA
4951000	NA	NA	NA	0	0	0	NA
340600	NA	NA	NA	303890	407270	796330	NA
0	NA	NA	NA	0	0	0	NA
4068200	NA	NA	NA	0	2056700	4337800	NA
2022700	NA	NA	NA	923520	3915700	5110700	NA
3469500	NA	NA	NA	0	0	0	NA
8116800	NA	NA	NA	3545600	6879800	4463900	NA
1314000	NA	NA	NA	291780	1193300	1664200	NA
0	NA	NA	NA	325720	760170	936730	NA
1288500	NA	NA	NA	1679800	2240500	5820000	NA
0	NA	NA	NA	0	0	0	NA
1905300	NA	NA	NA	0	1512200	2513500	NA
0	NA	NA	NA	0	0	0	NA
0	NA	NA	NA	458010	0	1018000	NA
0	NA	NA	NA	0	0	0	NA
0	NA	NA	NA	0	0	0	NA
269110	NA	NA	NA	1904100	2660700	2588700	NA

683840	NA	NA	NA	710250	0	0	NA
897210	NA	NA	NA	0	0	0	NA
1451500	NA	NA	NA	0	3041300	3302100	NA
2025000	NA	NA	NA	2671300	3931600	5380100	NA
0	NA	NA	NA	0	0	0	NA
0	NA	NA	NA	0	0	0	NA
0	NA	NA	NA	0	0	0	NA
0	NA	NA	NA	0	0	0	NA
1.70E+07	NA	NA	NA	23100000	35500000	58300000	NA
0	NA	NA	NA	0	691830	819070	NA
1616000	NA	NA	NA	0	0	0	NA
0	NA	NA	NA	0	0	0	NA
0	NA	NA	NA	0	0	0	NA
0	NA	NA	NA	0	0	0	NA
0	NA	NA	NA	0	0	0	NA
4877600	NA	NA	NA	4822500	2971300	3326800	NA
1347300	NA	NA	NA	416060	1172600	1531700	NA
1815600	NA	NA	NA	0	3275500	3128600	NA
1021200	NA	NA	NA	0	0	0	NA
724610	NA	NA	NA	0	0	1.50E+07	NA
0	NA	NA	NA	230980	2661400	2849300	NA
0	NA	NA	NA	0	0	0	NA
0	NA	NA	NA	0	0	0	NA
1977500	NA	NA	NA	1519300	3104200	7816300	NA
1351800	NA	NA	NA	0	0	2447400	NA
0	NA	NA	NA	0	0	0	NA
0	NA	NA	NA	0	3060100	0	NA
347630	NA	NA	NA	0	0	0	NA
274680	NA	NA	NA	425160	0	289090	NA
0	NA	NA	NA	0	0	0	NA
0	NA	NA	NA	0	3609500	611630	NA
0	NA	NA	NA	0	0	0	NA
3048300	NA	NA	NA	2404300	3557100	5497800	NA
2494700	NA	NA	NA	2517600	3433200	5419600	NA
2018200	NA	NA	NA	1292300	2019000	5146100	NA
0	NA	NA	NA	0	0	0	NA
0	NA	NA	NA	1288500	0	0	NA
1450200	NA	NA	NA	2789600	3076100	4632600	NA
0	NA	NA	NA	0	0	0	NA
0	NA	NA	NA	0	329820	348460	NA
0	NA	NA	NA	0	0	0	NA
0	NA	NA	NA	0	0	0	NA
4289800	NA	NA	NA	0	5878600	7573800	NA
1018600	NA	NA	NA	596950	1055000	1651800	NA
1540100	NA	NA	NA	822000	1918000	0	NA
1097200	NA	NA	NA	2427000	1333200	2048200	NA
0	NA	NA	NA	0	0	0	NA

951680	NA	NA	NA	945340	609350	805050	NA
0	NA	NA	NA	0	0	0	NA
0	NA	NA	NA	0	0	0	NA
0	NA	NA	NA	0	0	0	NA
1681700	NA	NA	NA	0	2202200	0	NA
1533500	NA	NA	NA	0	0	0	NA
1.67E+08	NA	NA	NA	92400000	1.70E+08	1.82E+08	NA
0	NA	NA	NA	0	0	0	NA
3109800	NA	NA	NA	0	4080500	3425900	NA
5440500	NA	NA	NA	1912100	4793500	6925400	NA
0	NA	NA	NA	0	0	0	NA
2.22E+09	NA	NA	NA	1.38E+09	1.77E+09	2.76E+09	NA
0	NA	NA	NA	0	0	0	NA
1433100	NA	NA	NA	0	2161600	751640	NA
0	NA	NA	NA	0	0	0	NA
1202200	NA	NA	NA	2179200	2758400	3493500	NA
0	NA	NA	NA	0	0	0	NA
0	NA	NA	NA	0	0	0	NA
0	NA	NA	NA	0	0	0	NA
0	NA	NA	NA	0	0	0	NA
0	NA	NA	NA	0	0	0	NA
0	NA	NA	NA	0	0	0	NA
1941900	NA	NA	NA	1297600	0	0	NA
980610	NA	NA	NA	1651400	0	3070400	NA
0	NA	NA	NA	529340	955250	1162200	NA
0	NA	NA	NA	0	772060	1591400	NA
0	NA	NA	NA	0	0	0	NA
0	NA	NA	NA	515260	830770	1349100	NA
538300	NA	NA	NA	2203900	809880	1489500	NA
650960	NA	NA	NA	1334000	1825400	0	NA
0	NA	NA	NA	0	0	0	NA
0	NA	NA	NA	0	2608700	7576400	NA
2179500	NA	NA	NA	0	0	0	NA
1944900	NA	NA	NA	1610100	1540600	0	NA
0	NA	NA	NA	0	0	0	NA
0	NA	NA	NA	0	0	0	NA
0	NA	NA	NA	0	0	2069200	NA
0	NA	NA	NA	0	259510	610730	NA
0	NA	NA	NA	560990	876750	2014100	NA
0	NA	NA	NA	0	2673400	2995800	NA
0	NA	NA	NA	0	0	0	NA
1195800	NA	NA	NA	1016100	948060	1396400	NA
0	NA	NA	NA	0	0	0	NA
0	NA	NA	NA	1004300	801030	0	NA
0	NA	NA	NA	0	0	0	NA
1890900	NA	NA	NA	1542900	1431700	4132900	NA
0	NA	NA	NA	0	0	0	NA

1213700	NA	NA	NA	0	0	0	NA
0	NA	NA	NA	0	0	0	NA
2018700	NA	NA	NA	1091800	2100500	2944800	NA
0	NA	NA	NA	2013900	0	3321400	NA
3373100	NA	NA	NA	0	1198500	0	NA
0	NA	NA	NA	0	0	0	NA
0	NA	NA	NA	1136800	1541700	1522000	NA
0	NA	NA	NA	0	0	0	NA
0	NA	NA	NA	1033800	0	0	NA
0	NA	NA	NA	18500000	0	41900000	NA
0	NA	NA	NA	0	0	0	NA
6584600	NA	NA	NA	0	0	0	NA
0	NA	NA	NA	1686200	563190	607400	NA
0	NA	NA	NA	0	0	0	NA
0	NA	NA	NA	449470	0	1120100	NA
8018500	NA	NA	NA	5846000	6992400	16100000	NA
0	NA	NA	NA	0	0	999560	NA
0	NA	NA	NA	0	2485200	0	NA
0	NA	NA	NA	0	0	0	NA
0	NA	NA	NA	0	1932800	0	NA
0	NA	NA	NA	0	0	0	NA
896960	NA	NA	NA	0	0	1316300	NA
0	NA	NA	NA	0	0	0	NA
0	NA	NA	NA	0	0	0	NA
0	NA	NA	NA	750830	953790	5664600	NA
0	NA	NA	NA	0	0	0	NA
0	NA	NA	NA	0	0	0	NA
0	NA	NA	NA	0	0	0	NA
0	NA	NA	NA	0	0	0	NA
0	NA	NA	NA	0	0	0	NA
0	NA	NA	NA	0	7163500	0	NA
11500000	NA	NA	NA	1.20E+07	18900000	25500000	NA
0	NA	NA	NA	0	0	0	NA
0	NA	NA	NA	0	0	0	NA
0	NA	NA	NA	0	0	0	NA
0	NA	NA	NA	34800000	55700000	94700000	NA
4520100	NA	NA	NA	0	3445600	4941900	NA
0	NA	NA	NA	0	0	0	NA
0	NA	NA	NA	0	0	0	NA
0	NA	NA	NA	0	83200000	0	NA
0	NA	NA	NA	0	0	0	NA
19600000	NA	NA	NA	22800000	20800000	32200000	NA
0	NA	NA	NA	0	0	0	NA
0	NA	NA	NA	0	0	0	NA
3200200	NA	NA	NA	4078800	4988600	6642100	NA
3764900	NA	NA	NA	9276000	16200000	23100000	NA
3044300	NA	NA	NA	3202100	2971300	3326800	NA

4226600	NA	NA	NA	0	0	3196900	NA
0	NA	NA	NA	0	0	0	NA
0	NA	NA	NA	0	0	0	NA
0	NA	NA	NA	986860	1615400	0	NA
0	NA	NA	NA	0	0	0	NA
0	NA	NA	NA	0	0	0	NA
0	NA	NA	NA	1386800	1284800	1479200	NA
0	NA	NA	NA	0	0	0	NA
0	NA	NA	NA	0	0	0	NA
0	NA	NA	NA	0	0	0	NA
0	NA	NA	NA	0	0	0	NA
2422400	NA	NA	NA	2821500	3619900	5464700	NA
8903400	NA	NA	NA	6408600	11700000	15900000	NA
0	NA	NA	NA	0	0	0	NA
0	NA	NA	NA	0	0	0	NA
1713600	NA	NA	NA	2753200	4170700	5963100	NA
2362700	NA	NA	NA	1167700	3805600	3774600	NA
NA	9302200	15200000	27300000	NA	NA	NA	0
0	NA	NA	NA	0	0	0	NA
61800000	NA	NA	NA	44800000	5.10E+07	1.00E+08	NA
NA	1069300	1275500	0	NA	NA	NA	0
883980	NA	NA	NA	537480	716280	1527400	NA
NA	0	0	0	NA	NA	NA	0
NA	35500000	11800000	37900000	NA	NA	NA	6962100
2725800	NA	NA	NA	3211700	4249400	7553400	NA
0	NA	NA	NA	542310	1022900	3050200	NA
NA	3298700	24100000	37400000	NA	NA	NA	3826800
NA	4105300	3902100	7216900	NA	NA	NA	1051900
NA	0	0	0	NA	NA	NA	0
0	NA	NA	NA	0	0	0	NA
NA	9.44E+08	9.04E+08	1.37E+09	NA	NA	NA	2.56E+09
4717300	NA	NA	NA	0	4346000	9454200	NA
NA	0	0	0	NA	NA	NA	0
NA	3.24E+08	2.47E+08	3.88E+08	NA	NA	NA	27400000
21800000	NA	NA	NA	9076100	9936800	25100000	NA
4648700	0	0	0	0	0	0	0
6616700	NA	NA	NA	2125700	4570700	12400000	NA
NA	1.16E+08	1.12E+08	1.35E+08	NA	NA	NA	2.73E+08
NA	8351700	9924900	11300000	NA	NA	NA	0
NA	4050000	0	0	NA	NA	NA	429440
1259800	NA	NA	NA	718190	1212100	633900	NA
NA	3607200	224690	4129000	NA	NA	NA	0
NA	484780	10700000	28400000	NA	NA	NA	0
NA	93400000	82200000	1.40E+08	NA	NA	NA	47500000
13600000	NA	NA	NA	2858700	5592100	6516400	NA
2722600	NA	NA	NA	2036800	1449500	4620600	NA
0	NA	NA	NA	7585800	6301300	1599700	NA

NA	3249300	3347300	9126700	NA	NA	NA	0
NA	5543300	0	870810	NA	NA	NA	1322500
NA	73800000	8.70E+07	91700000	NA	NA	NA	66300000
NA	0	2395600	0	NA	NA	NA	0
0	NA	NA	NA	1080200	0	12800000	NA

FR_SDS.MA	FR_SDS.MA	FR_SDS.MA	FR_SDS.MA	FR_SDS.MA	FFPE_MAX	FFPE_MAX	FFPE_MAX	FFPE_MAX
**FR4.1.M**	**FR7.1.M**	**FR1.2.M**	**FR4.2.M**	**FR7.2.M**	**F1.1**	**F4.1**	**F8.1**	**F1.2**
9.73E+09	7.12E+09	1.79E+09	2.67E+09	3.07E+09	1.12E+09	1.15E+09	2.84E+09	2.94E+08
8.07E+08	1.30E+09	3.10E+08	2.05E+08	5.40E+08	4.22E+09	3.06E+09	3.41E+09	1.03E+09
6.72E+08	1.34E+09	1.72E+08	1.47E+08	4.52E+08	8.07E+09	5.66E+09	7.17E+09	1.67E+09
2.07E+09	1.72E+09	7.38E+08	4.82E+08	6.52E+08	2.77E+09	1.98E+09	2.45E+09	6.47E+08
3.56E+09	2.88E+09	1.62E+09	1.05E+09	1.31E+09	3.64E+09	2.19E+09	2.52E+09	8.10E+08
8.51E+09	3.13E+09	1.23E+09	2.25E+09	1.39E+09	1.06E+09	7.37E+08	1.91E+09	2.94E+08
1.29E+10	8.96E+09	2.35E+09	3.19E+09	3.24E+09	2.35E+09	1.42E+09	3.80E+09	5.08E+08
2.60E+08	9.99E+08	6.10E+07	57600000	4.46E+08	1.04E+10	7.18E+09	8.94E+09	2.54E+09
3.46E+09	2.28E+09	1.22E+09	7.99E+08	9.32E+08	3.22E+09	2.12E+09	2.57E+09	7.87E+08
3.90E+08	4.36E+08	1.85E+08	1.13E+08	2.35E+08	1.15E+09	9.54E+08	1.11E+09	3.37E+08
4.47E+09	3.44E+09	1.69E+09	1.14E+09	1.34E+09	3.65E+09	2.67E+09	2.89E+09	9.96E+08
7.51E+09	8.61E+09	1.99E+09	2.64E+09	4.32E+09	1.41E+09	7.64E+08	2.85E+09	3.75E+08
1.11E+09	9.23E+08	3.99E+08	2.76E+08	3.91E+08	3.11E+09	2.18E+09	2.46E+09	6.74E+08
3.12E+09	2.17E+09	8.11E+08	7.01E+08	7.41E+08	2.38E+09	1.55E+09	1.80E+09	5.38E+08
3.79E+09	2.57E+09	1.21E+09	7.73E+08	9.24E+08	1.50E+09	1.45E+09	1.32E+09	3.04E+08
1.16E+08	1.03E+08	30100000	27600000	49500000	1.92E+08	1.56E+08	1.62E+08	79700000
28400000	5.50E+07	19800000	6981500	27200000	4.69E+08	3.21E+08	4.25E+08	1.05E+08
2.55E+08	4.69E+08	1.06E+08	6.80E+07	2.59E+08	2.76E+09	1.84E+09	2.32E+09	6.22E+08
22100000	41100000	4238200	1210400	17600000	5.87E+08	3.48E+08	4.50E+08	1.42E+08
1.00E+07	47700000	942830	4691000	18400000	1.04E+09	6.48E+08	8.07E+08	2.08E+08
1.28E+08	1.46E+08	39500000	33200000	74700000	1.20E+09	7.72E+08	9.91E+08	2.66E+08
1.17E+09	8.85E+08	4.25E+08	2.72E+08	3.53E+08	2.41E+09	1.67E+09	1.90E+09	5.28E+08
9.31E+08	7.25E+08	3.80E+08	2.83E+08	3.43E+08	7.00E+08	4.21E+08	5.66E+08	1.82E+08
1.16E+09	1.13E+09	2.93E+08	2.69E+08	5.12E+08	2.27E+09	1.77E+09	1.77E+09	4.98E+08
4.36E+09	3.24E+09	1.25E+09	8.59E+08	9.65E+08	1.19E+09	1.38E+09	1.02E+09	3.51E+08
3.30E+07	1.46E+08	1.30E+07	6161600	51700000	1.19E+09	7.71E+08	8.91E+08	2.35E+08
14600000	20500000	10900000	5602000	21600000	4.10E+08	2.64E+08	3.51E+08	92800000
2.43E+09	2.88E+09	9.66E+08	9.97E+08	1.12E+09	1.94E+09	1.79E+09	1.61E+09	6.63E+08
27400000	1.20E+08	6506500	6317500	54500000	1.42E+09	9.35E+08	1.11E+09	3.35E+08
4.40E+09	3.44E+09	1.45E+09	8.51E+08	9.58E+08	1.33E+09	1.34E+09	1.35E+09	2.37E+08
5.92E+08	5.06E+08	2.02E+08	1.54E+08	2.13E+08	3.22E+08	3.06E+08	3.00E+08	61700000
3.04E+09	2.41E+09	8.39E+08	8.66E+08	1.02E+09	2.01E+09	9.05E+08	1.26E+09	4.05E+08
1.65E+08	2.87E+08	50400000	34900000	1.37E+08	1.03E+09	7.35E+08	8.33E+08	2.17E+08
9.24E+08	5.90E+08	3.82E+08	2.27E+08	2.97E+08	5.11E+08	4.35E+08	6.02E+08	1.67E+08
96300000	1.39E+08	27600000	21500000	77400000	9.44E+08	6.81E+08	7.87E+08	2.32E+08
4116200	28700000	1855500	3752700	16700000	4.59E+08	3.32E+08	3.30E+08	9.90E+07
13400000	37700000	2455800	2370800	15500000	3.75E+08	2.58E+08	3.13E+08	79700000
8.18E+08	6.88E+08	2.22E+08	1.92E+08	2.21E+08	1.39E+09	1.14E+09	1.07E+09	3.11E+08
32200000	63600000	1760100	7851600	27200000	4.20E+08	3.73E+08	3.50E+08	1.16E+08
96400000	1.26E+08	22600000	20700000	42300000	3.64E+08	2.90E+08	3.92E+08	60800000
14100000	31800000	4510900	4788400	9652300	1.70E+08	84200000	1.19E+08	50900000
68800000	77800000	29900000	25400000	41700000	1.47E+08	97500000	1.11E+08	42500000
6286100	41400000	1888200	1839200	26600000	5.71E+08	3.56E+08	4.03E+08	97700000
5.75E+08	8.81E+08	2.17E+08	1.56E+08	65800000	45700000	42700000	27200000	2620800
64100000	1.18E+08	15900000	19900000	51800000	8.19E+08	4.62E+08	5.95E+08	1.83E+08

40400000	26500000	5493600	10200000	12100000	0	2482800	2254800	0
18900000	41400000	637690	5497100	20200000	4.24E+08	1.95E+08	2.54E+08	99400000
61200000	86800000	16700000	1.50E+07	35800000	6.38E+08	3.83E+08	4.63E+08	1.43E+08
1.02E+08	1.71E+08	21400000	25200000	8.00E+07	1.13E+09	7.02E+08	9.41E+08	2.54E+08
6.60E+07	1.10E+08	44200000	20500000	45700000	1.76E+08	1.52E+08	1.52E+08	44200000
1.20E+07	19300000	3257800	2263000	8162700	1.13E+08	74300000	90100000	32600000
24500000	9.40E+07	29300000	5211800	35900000	3.20E+08	2.02E+08	2.29E+08	61100000
1.10E+07	53400000	3775300	4306200	18700000	1.54E+08	1.35E+08	1.47E+08	36800000
17300000	38900000	5806500	3915700	1.80E+07	3.09E+08	2.16E+08	2.01E+08	53200000
21300000	47700000	6137400	4495700	16200000	3.55E+08	1.92E+08	3.20E+08	69800000
1.78E+08	1.98E+08	1.22E+08	38700000	94100000	1.03E+08	0	0	1909500
26700000	5.30E+07	8212700	2944800	27600000	3.26E+08	1.82E+08	2.15E+08	56100000
3521600	16900000	1516700	1944000	16800000	4.77E+08	3.23E+08	3.70E+08	1.16E+08
4529400	4228800	12500000	1043300	4190400	3.67E+08	2.79E+08	2.25E+08	85500000
1.07E+08	2.50E+08	24400000	26800000	98300000	1.81E+09	1.16E+09	1.15E+09	4.81E+08
1.27E+08	76800000	56900000	19200000	31300000	2.14E+08	1.43E+08	1.75E+08	53200000
5287300	18200000	3064300	1257700	8973300	97500000	57100000	72200000	25200000
22800000	45300000	7974700	5465300	15100000	1.52E+08	1.15E+08	1.29E+08	36200000
588290	11100000	1757300	985500	3212400	62400000	5.60E+07	46100000	14200000
0	18200000	1610300	1358900	11500000	1.10E+08	92700000	1.38E+08	26700000
29900000	69700000	10600000	6439400	30800000	2.29E+08	2.28E+08	2.53E+08	82300000
24900000	30100000	4236100	5118700	11500000	1.50E+08	92100000	1.16E+08	36700000
8606100	37300000	2353600	3554400	17300000	3.61E+08	2.19E+08	2.82E+08	73100000
2150600	3166500	0	0	0	3.57E+08	2.50E+08	3.29E+08	1.01E+08
3107900	7901000	1012500	985510	3700300	1.71E+08	1.05E+08	1.47E+08	35900000
92200000	98100000	6201900	20200000	49600000	0	2790700	29600000	0
68100000	1.48E+08	20500000	19900000	75300000	1.83E+09	1.38E+09	1.49E+09	4.81E+08
1.15E+08	1.17E+08	85200000	24300000	60200000	2.04E+08	2.13E+08	1.63E+08	58200000
26500000	47300000	7657400	7147400	22100000	3.38E+08	2.39E+08	2.68E+08	67900000
33300000	49500000	5051900	6994200	22100000	2.93E+08	1.69E+08	2.01E+08	57200000
28300000	0	590450	0	0	0	0	0	0
267160	12500000	6163800	564600	5976000	48600000	3.30E+07	56900000	10500000
62800000	1.05E+08	25800000	41600000	52600000	6.32E+08	3.84E+08	5.86E+08	1.91E+08
0	6664400	0	0	3271900	2.97E+08	2.46E+08	3.30E+08	73200000
4299700	13100000	1415800	5180700	4917200	59900000	49400000	47400000	1.70E+07
5433600	5378000	1566000	1597800	5093800	2.82E+08	1.54E+08	2.77E+08	67200000
1.54E+08	98400000	73300000	54300000	60500000	88500000	69700000	89200000	34800000
54600000	70700000	36100000	11700000	74800000	22900000	5580400	21100000	4931100
27100000	14400000	4120100	1037800	19400000	2.14E+08	1.69E+08	1.68E+08	52500000
1476500	3304600	0	1299500	5261500	54900000	4.50E+07	36500000	15700000
0	7423200	474130	360830	1108000	43600000	44900000	54700000	1.30E+07
3456800	7099700	2236000	6222400	9537300	42700000	35800000	38800000	14700000
15100000	9727100	1575900	0	5698600	1.79E+08	1.56E+08	1.76E+08	4.90E+07
2589200	21900000	2447200	0	5157700	53700000	47100000	58500000	16300000
1.27E+08	82100000	40100000	30400000	57300000	2.91E+08	1.61E+08	1.80E+08	65900000
0	2873300	285830	262260	1829600	36300000	34600000	20600000	8366700
9526200	16200000	2676000	1619000	1900600	28800000	11400000	18900000	3038100

10500000	1.80E+07	598530	1720900	8499700	5.90E+07	71900000	86500000	17200000
17900000	3.20E+07	12300000	3860800	11600000	19900000	26300000	24600000	8940800
17400000	39400000	1130100	3888200	18100000	1.23E+08	9.00E+07	1.13E+08	30100000
7557900	6732300	1294600	1015100	4299700	3.30E+07	24100000	30200000	9495400
0	22200000	819790	264430	9069500	34800000	21800000	1.30E+07	5566800
8541100	8147200	759540	2373100	8578900	1.37E+08	71800000	1.05E+08	39700000
11800000	14500000	3017600	3045000	3167400	51200000	30700000	34800000	10500000
5178700	24100000	2990300	2959300	7986200	4.32E+08	2.95E+08	4.90E+08	98600000
1249000	7591200	1565300	2148600	8019900	35400000	3.00E+07	36800000	8552200
77400000	1.50E+08	75800000	38900000	49600000	8110600	0	0	0
92200000	84900000	28400000	26800000	49900000	5.23E+08	4.21E+08	3.37E+08	1.62E+08
1.62E+08	1.29E+08	59200000	46200000	53600000	1.18E+08	39200000	76400000	2.50E+07
5112700	44300000	9109300	1063900	19600000	2.19E+08	1.55E+08	1.94E+08	6.00E+07
1.50E+07	6562900	11800000	2968600	13300000	60500000	36700000	29900000	17400000
0	0	0	0	0	2.25E+08	1.64E+08	1.71E+08	5.40E+07
12800000	55800000	4297700	3631700	15100000	3.24E+08	2.34E+08	2.74E+08	6.40E+07
3527000	2843500	613510	559470	0	1.12E+08	9.70E+07	74900000	31200000
0	10500000	2084200	1575600	5504500	1.93E+08	1.37E+08	1.52E+08	5.40E+07
2589800	11600000	992180	373340	9820300	1.25E+08	52200000	80100000	26800000
0	8205600	0	0	4024900	2.14E+08	1.64E+08	2.05E+08	4.30E+07
19400000	1.90E+07	4012200	4073900	7597400	4181900	0	4410800	0
35500000	43200000	4601400	5742600	18600000	0	0	1764500	0
1.20E+07	20200000	4685800	4208100	5155400	43100000	47400000	47400000	12100000
6240000	9578000	1085400	991730	3414300	80500000	1.02E+08	1.17E+08	40600000
10800000	21700000	4370000	2877400	12300000	43400000	49600000	47200000	1.90E+07
0	0	0	559990	2429000	27500000	32300000	4.00E+07	9114100
12800000	23300000	1542800	3790100	4677800	2.01E+08	1.59E+08	2.00E+08	58300000
15700000	42800000	12300000	9513600	14400000	2.27E+08	1.34E+08	1.73E+08	41800000
6186500	4831600	3140200	1764900	4457700	85400000	65200000	61100000	23700000
0	3472400	0	682130	0	4.60E+07	45300000	49600000	10300000
3.42E+08	2.17E+08	1.27E+08	77700000	9.80E+07	1.28E+08	1.01E+08	1.24E+08	3.70E+07
11800000	14800000	1110100	2575900	7456500	1.35E+08	1.19E+08	1.21E+08	28400000
10500000	9534500	4711000	1831500	4662700	16900000	16700000	27500000	7562900
0	6178600	195880	459490	2131200	46500000	32800000	37900000	15300000
211080	2389500	1357600	1547200	3106700	30700000	51700000	86500000	9398100
1462300	4565200	0	0	2402800	12500000	14600000	1.40E+07	6782800
0	5790400	375610	0	4035300	39300000	36400000	47500000	13500000
17600000	16400000	22700000	8186300	14600000	51200000	32700000	43500000	16600000
1146500	0	0	0	2325100	43900000	21700000	45300000	5432800
18100000	37700000	6973700	4226100	18200000	1.88E+08	2.48E+08	2.43E+08	65600000
32500000	44600000	14600000	7830700	14500000	74500000	87200000	1.06E+08	9141600
0	0	0	0	0	37600000	21400000	27600000	6459900
19900000	24900000	5092300	5565800	11800000	0	0	0	0
0	0	0	0	0	1.15E+08	1.01E+08	1.03E+08	24800000
0	2502700	0	0	588420	4.00E+07	25500000	33800000	5174700
0	1456300	0	0	988890	85400000	17200000	43700000	6696900
0	10700000	1258400	0	7129600	0	0	9082000	2535300

11700000	19200000	12700000	4433900	12200000	2456400	5092600	8860000	0
21400000	1.70E+07	17400000	7234800	12700000	19100000	15700000	16400000	6227200
2323000	6306100	5062700	3740200	3190300	16100000	1.10E+07	11700000	3486800
615060	5567000	1870700	398340	4074300	44800000	50200000	20400000	9580800
1.02E+08	1.25E+08	4.80E+07	34100000	31800000	2.30E+08	44800000	1.13E+08	43100000
0	600630	0	0	1103000	14500000	13900000	18700000	5052900
579150	3563800	0	368110	3074800	24200000	18200000	13400000	4112600
10300000	7416100	2335500	2152700	6581300	74400000	55700000	4.60E+07	1.40E+07
16800000	14900000	18300000	6116800	1.20E+07	0	0	0	0
19700000	32600000	8504800	2144600	11400000	34300000	38500000	2295200	8580500
30500000	20800000	3771000	5167000	5408100	3.26E+08	2.03E+08	3.15E+08	78900000
2.10E+07	46900000	17100000	4723900	11700000	17600000	50200000	3.10E+07	1.40E+07
28100000	19400000	10300000	7588800	4161900	41600000	18600000	15300000	3687800
1.20E+07	1.40E+07	6518000	2018100	4593700	1.36E+08	82800000	1.90E+08	31800000
0	204010	220950	0	1648000	39700000	26600000	33300000	11400000
3381500	13300000	2963500	944030	5045500	38800000	30700000	32500000	13700000
17100000	23300000	6980300	3431400	7418000	4.80E+07	24500000	29300000	7072300
8037900	22200000	12200000	2236800	9389200	0	0	4373200	0
0	10400000	250650	531210	2708700	62700000	42700000	60400000	15300000
0	0	0	0	452200	52800000	39900000	39500000	6822400
364850	0	0	642790	1467500	3.50E+07	23400000	31900000	9176400
13800000	24200000	6146500	1630600	1.20E+07	1.19E+08	89600000	1.13E+08	30900000
9245200	8815300	13800000	3204400	7807600	0	0	4557400	0
0	3216200	0	0	2134100	4483300	14800000	9669200	4410800
0	2116700	859990	0	0	0	0	0	0
14400000	14300000	1251100	2323100	5171200	0	0	0	0
0	7667900	1016600	0	1650900	58800000	50200000	60200000	10900000
1498900	1907100	525920	398560	885530	20100000	8477900	8509600	5679700
2940700	10300000	1931700	1446000	4545600	35400000	17300000	28800000	6898100
1598700	11500000	8489600	1323300	472920	0	0	0	0
0	11100000	353730	0	2088300	83800000	25900000	37100000	12700000
0	3722200	0	0	1844700	61300000	61200000	36900000	25100000
20500000	20800000	7448600	6807300	7176300	41400000	11100000	3.50E+07	10700000
6242400	18700000	2496900	0	1737000	31800000	52400000	42500000	9196200
0	3059200	904050	0	3863200	43300000	29400000	31700000	10800000
0	0	0	0	0	53100000	23500000	34100000	7024500
0	4140800	0	0	1137200	2.40E+07	16700000	22900000	6362600
398140	7855300	5273700	1233700	4269300	15300000	8160200	13200000	4511500
0	5904600	0	0	1287200	13600000	15600000	8271800	5936100
794110	1978200	0	249600	1026500	25700000	17900000	20700000	6460400
0	3162100	0	0	2323200	48700000	48200000	57200000	9369600
0	1020900	0	0	398660	32600000	2.30E+07	13400000	6279200
0	5170000	0	0	2676900	13700000	16800000	21200000	4380700
9738900	30200000	0	3055900	0	4.24E+08	2.88E+08	3.87E+08	1.10E+08
0	0	0	0	0	35200000	28900000	32600000	7338700
0	2806700	0	0	0	21400000	24100000	23500000	2857800
5592400	9453500	4828700	1324700	3711100	8077900	0	4348700	0

0	3546000	0	0	2233000	30600000	17400000	33300000	10900000
0	8691400	0	0	2858800	48800000	52600000	63300000	16200000
0	0	0	0	2931200	53100000	38200000	44100000	11300000
47800000	1.20E+08	7695100	15100000	32900000	0	0	3831400	0
0	0	0	0	754060	49100000	27800000	62100000	9772500
3356900	3013600	2413300	806540	1814300	15200000	11600000	17800000	6732500
0	933350	225380	0	2591200	4987800	0	6047600	1184700
0	6515000	815960	856610	3555700	1.36E+08	74700000	92200000	24900000
0	1950600	0	0	1432200	42800000	32300000	35900000	1.50E+07
0	165830	0	0	0	17600000	10700000	14600000	2796500
0	0	0	0	404960	18900000	7461300	1.20E+07	4074800
667160	15400000	0	0	0	30700000	19400000	31900000	9259700
0	0	0	0	0	0	0	0	0
1.01E+08	73900000	42700000	29800000	27700000	1.50E+08	68300000	1.05E+08	34900000
2713400	2.40E+07	10400000	5872400	1.60E+07	19100000	4710300	11800000	5289100
811530	3700900	778490	340570	1729400	26400000	19200000	15700000	2878800
0	837720	0	0	0	41700000	22900000	22700000	6817500
0	0	0	0	0	20800000	21700000	2.00E+07	6361200
2394200	0	0	1677000	0	43600000	44800000	29800000	11800000
0	3488500	0	0	2351700	1.36E+08	80300000	1.15E+08	31700000
0	2177900	0	0	842170	97500000	1.21E+08	98300000	35800000
12600000	21800000	462490	0	7779800	1.33E+08	1.13E+08	1.13E+08	34700000
2.64E+09	1.16E+09	3.62E+08	6.76E+08	4.45E+08	2.00E+08	2.25E+08	4.75E+08	75200000
0	0	0	0	0	98900000	91100000	99900000	7943700
0	2109900	691350	0	1613500	55600000	5.50E+07	70100000	11800000
0	0	0	0	2347500	42200000	13500000	27500000	8678100
4647100	3602900	3183300	1570800	3360300	11900000	0	3173000	0
0	4496800	0	0	1614700	18700000	10200000	12300000	10400000
0	0	0	0	0	18700000	16900000	33400000	8372800
0	10400000	0	0	0	3981800	0	0	0
77900000	56600000	21500000	14900000	1.70E+07	3.70E+07	9364600	13800000	3766900
0	1575800	0	0	436500	38400000	21600000	32300000	14200000
0	0	0	0	0	0	0	0	0
0	0	0	0	0	7094800	0	0	0
2720000	10700000	3740400	683440	2625800	22500000	12700000	6370100	2043500
1975200	1606500	1616500	461890	980150	63800000	32200000	45100000	11300000
3843300	7625400	2251700	1273500	3581000	36800000	25100000	29800000	7142800
5241500	0	2588100	1441400	1064500	29900000	23700000	22800000	7611900
1812500	268660	1188900	635490	2649400	1049100	2370100	980220	1769100
37100000	0	0	0	0	19600000	10800000	1.60E+07	2103000
18700000	8557500	2660100	1573600	952970	98700000	80300000	72100000	23800000
0	0	0	0	0	30200000	19400000	23900000	2457100
0	0	0	0	0	87500000	4.90E+07	52600000	20600000
0	8615300	0	1252500	3684000	18900000	13100000	15800000	1109600
0	0	0	0	0	4356000	0	0	0
0	2480900	0	0	5496000	62700000	46500000	54300000	20700000
0	0	0	0	0	30500000	8257600	23600000	8266800

0	0	354210	0	421790	6236000	1984300	4558100	939790
0	0	0	0	501270	30300000	12700000	20500000	8084200
3916000	8797800	796880	0	527210	0	0	0	0
0	54300000	0	0	0	10100000	7430600	11400000	2158200
1.20E+07	14200000	4481800	2484200	4806500	11800000	7840600	1.10E+08	2285300
0	823760	0	0	520320	13200000	9955100	1.20E+07	3675700
2780500	2001200	2056600	0	859150	0	1493700	0	0
0	0	0	0	0	0	0	0	0
4489300	11500000	5964400	871590	4804800	0	6218400	3670100	0
1.01E+09	9.84E+08	3.39E+08	1.74E+08	6.93E+08	6.86E+09	4.97E+09	5.84E+09	8.72E+08
0	0	0	0	0	47100000	3.90E+07	46700000	11500000
0	0	0	0	0	10300000	2136800	5444400	2907000
0	0	0	0	0	43700000	3230000	7075700	1010900
0	0	0	0	0	13300000	9566500	28900000	4345700
0	0	0	0	0	10200000	12600000	8864000	5304300
1992700	3069100	938790	375070	3105000	24900000	12100000	2.50E+07	5078300
0	0	0	0	1171000	0	6195200	7333900	0
0	1000900	0	0	745400	9732800	5507200	12300000	2413500
0	633700	0	0	0	2.10E+07	12200000	16500000	7069900
0	0	327310	0	603620	10500000	11500000	9275200	4214000
0	0	0	0	328070	8282900	1212000	6795800	1781200
1858600	337610	190520	0	240420	6591500	585420	5860900	2689600
0	0	0	0	0	1.80E+07	11800000	1.30E+07	3595300
0	0	0	0	0	0	1305200	1868300	4533200
0	0	0	0	659040	2804700	3970600	5716200	4098100
60300000	35500000	12400000	12400000	16600000	76900000	99900000	1.17E+08	27100000
0	2202500	0	0	0	11400000	15200000	19200000	3916400
0	2429200	0	0	954620	2.20E+07	9730100	1.50E+07	0
0	253800	0	0	193470	18300000	14500000	3744300	4459900
0	0	0	0	0	6041400	5356500	2526600	1241700
0	0	0	0	0	0	0	6088700	0
0	8086900	3884400	338830	5122100	5387800	21100000	5927600	0
0	0	0	0	0	38900000	13600000	24600000	2309700
0	701060	0	0	483320	12700000	6827700	10400000	5943600
0	1923000	0	0	496020	9730700	8168300	8375100	1819500
0	0	0	0	0	12100000	1669400	11600000	2918400
0	0	0	0	0	18500000	8152000	12200000	4409100
1402200	2392600	2223900	386400	2120500	0	0	0	0
0	0	0	0	0	0	0	447990	0
6099900	5227700	3596600	1624500	5853600	12400000	8634200	8947100	2925700
0	0	0	0	0	8650800	10800000	10600000	2896300
1537400	3385500	2343700	805250	1806400	6644900	4090300	5410100	4203500
0	0	0	7598900	0	12200000	13100000	13500000	2242500
0	8234300	1481500	0	1720000	0	0	0	0
1524700	6881100	2530700	352280	3679000	7109100	2589000	11400000	1729500
0	2529400	0	0	0	27500000	20800000	12700000	4486500
2298100	2627000	554680	434620	2726000	10600000	7430100	10300000	2768900

0	0	0	0	838650	2.20E+07	28100000	39500000	7197200
3071300	13100000	1413500	1072100	6695700	80400000	41700000	54400000	17800000
0	0	0	0	1016500	39200000	19400000	38400000	11900000
0	0	0	0	0	548140	0	228390	1516900
0	6356200	0	1391200	3787200	8532000	15200000	11900000	1405900
586780	0	0	0	1222800	0	0	0	0
0	1555000	0	0	797730	1.40E+07	10100000	1.40E+07	4118900
0	0	0	0	0	2891700	1827200	4103100	849000
0	0	0	0	0	20700000	12900000	8315700	3046400
0	0	0	0	0	17600000	7540600	9205800	4345300
77500000	0	1607300	19200000	1256100	12900000	5551400	12900000	0
0	0	0	0	0	11700000	1.30E+07	17800000	3391000
8347300	9149400	0	1610700	5492400	0	0	0	0
0	1008400	0	0	0	3534400	8328300	14800000	0
0	0	0	3233600	802610	2362500	3492400	6973700	2915400
0	0	0	0	2042200	0	0	0	0
0	0	0	0	0	750170	328800	737690	0
0	0	0	0	0	14600000	9111400	7630400	5567100
0	816030	0	0	0	13700000	13600000	11300000	735370
0	0	0	0	0	13200000	17900000	7794400	6310900
0	0	0	0	0	3192800	1.00E+07	3680000	1269800
0	0	0	0	0	39400000	1.50E+07	1.90E+07	8457100
5489100	8133800	2770700	2162100	3661100	42900000	25500000	33700000	8190800
0	0	0	0	0	14200000	12900000	12200000	1827800
0	0	0	0	0	9546100	1195700	8497200	669300
0	1307300	0	0	763600	12500000	0	8079400	3216000
0	7366300	3917200	2038700	5952000	4946200	3346700	9268000	3017500
0	0	0	0	0	24500000	8225600	4765000	7553900
0	0	0	0	0	16300000	16700000	10500000	1120600
0	0	0	0	0	14100000	17200000	23700000	4499400
0	0	0	0	0	53100000	28200000	35300000	9431700
0	12600000	0	0	0	36600000	12200000	27500000	3134200
0	4925400	0	0	1871300	7999600	12200000	11900000	4401800
0	0	0	0	1678100	9671100	3861500	1451300	185640
0	0	0	0	0	3363200	3198500	0	1464100
0	0	0	0	1669100	49200000	23500000	32500000	13700000
0	0	0	0	1896700	0	0	0	0
0	0	0	0	0	7591600	1422200	3779200	1673900
3071300	6337200	1413500	1072100	2574900	51800000	29300000	37200000	10500000
86500000	46700000	29100000	47400000	22300000	41700000	24300000	31200000	35700000
0	0	0	0	0	10100000	3005800	2836800	1132900
0	0	0	0	0	14300000	10900000	12900000	4121000
0	6460100	0	0	0	1.22E+08	93200000	1.01E+08	24200000
0	0	0	0	0	20900000	2.80E+07	35300000	14800000
6590000	5405600	2594100	0	2233000	0	0	0	0
0	0	0	0	0	0	773220	0	0
0	1933600	874400	0	820690	17200000	8573700	4684700	1605100

0	1957500	0	0	1402700	1718100	9738100	5585000	0
0	1133900	0	0	442540	5787200	3510400	11600000	1685700
0	3028400	0	0	588840	1.10E+07	8433400	12400000	2622000
0	3270000	1806200	0	3645800	4658400	0	0	0
0	0	0	0	0	6274800	4891700	7203300	1313400
0	0	0	0	0	13800000	4799200	10700000	2582200
847080	3757600	0	0	3718800	30900000	2.10E+07	23400000	500640
0	1031800	0	0	420610	20500000	2117500	3422700	1097300
0	0	0	0	0	0	0	0	0
0	0	0	609060	1873500	2876800	2055300	2023100	686460
0	0	0	0	0	0	8564100	0	0
0	0	0	0	0	11500000	0	849330	0
0	0	0	0	0	0	6083300	9591200	0
0	1069300	0	0	659830	31400000	5684100	13500000	467100
0	0	0	0	0	4264400	2820800	1.10E+07	0
14700000	0	0	244900	0	28800000	16300000	13200000	8017400
0	0	0	0	0	14800000	12500000	19100000	3759700
0	0	0	0	0	13400000	8553800	8172000	3266700
0	3064400	1156700	629410	1811200	12700000	7670500	8297300	1522600
0	0	0	0	0	0	0	0	0
17900000	0	0	4032400	940620	0	0	0	0
0	2004200	0	455250	1627200	10400000	2729900	1.30E+07	2753700
0	0	0	0	0	4740800	1615100	10900000	887850
0	0	0	0	0	2992800	3861200	0	1160800
0	0	0	578850	0	48700000	32400000	41300000	10500000
0	0	0	0	0	0	0	770420	0
0	0	0	0	0	1088600	797320	0	438660
0	0	0	0	0	0	0	0	0
0	0	0	0	488710	3102100	2179100	3225800	650070
0	0	0	0	0	0	0	0	0
0	0	0	0	0	0	0	0	0
0	0	0	0	0	4156500	1040600	3541400	664820
0	0	0	0	0	9050200	13600000	8234200	2860200
0	0	0	0	0	8789300	0	1.10E+07	3643000
0	0	0	0	0	0	2676500	5784500	0
0	0	159180	0	0	5041100	2796700	0	2081400
0	0	0	0	0	17900000	1752000	9943300	2187900
0	4586300	0	0	1285300	37800000	29100000	18200000	5161500
0	412490	0	0	0	4283400	4609300	8192100	1313000
0	1483800	0	0	967800	6588400	1734700	5488600	0
0	0	0	0	902710	12100000	5630300	1.10E+07	816050
0	214530	0	0	0	12300000	606570	10100000	2756100
0	0	0	0	0	5155000	0	0	0
0	0	0	0	0	0	0	0	0
0	3257800	0	0	0	5829500	3316800	4935600	1048800
0	0	0	0	268860	8152700	765990	3897300	606820
0	0	0	0	0	0	0	0	565640

0	1608100	0	0	0	13200000	1.20E+07	13600000	2522700
0	0	0	0	745340	4318800	5308900	7333400	2263100
0	0	0	0	702370	16500000	11600000	8752800	3208500
0	0	0	0	4448000	3683900	2569200	3110800	850910
0	0	0	0	0	30600000	15300000	29800000	7227800
0	0	0	0	0	10600000	2225700	17300000	3307000
0	2659900	693740	0	1856700	6924700	4166000	8279800	1797800
0	575570	188640	0	0	0	7274200	6007200	980350
0	0	0	0	0	0	0	0	0
0	0	0	0	0	1571300	2043100	5231400	0
0	0	0	0	1098900	0	0	0	0
0	0	0	0	0	7256100	16200000	12300000	2028900
0	0	0	0	0	1.40E+07	4523200	14600000	4647500
0	0	0	0	0	0	3033900	3130300	1131700
0	0	0	0	0	0	0	0	1764700
9632200	11400000	5142100	2866300	868320	0	0	0	993820
0	0	0	0	0	12400000	10700000	25900000	7043000
0	0	0	0	0	0	0	0	1459200
0	3376400	0	0	2207900	8026900	18800000	5151500	3626400
0	0	0	0	0	0	0	0	0
0	0	0	370360	644110	15600000	13300000	12500000	2599200
17900000	6442900	9214500	3763400	5700900	0	0	0	0
0	0	0	0	0	4011700	444070	1802100	1984100
0	0	0	0	0	0	0	0	0
0	0	0	0	0	9484100	3744100	2882700	2611600
0	0	0	0	631870	13300000	956470	3540700	1335300
26100000	10200000	5119100	2819500	5937100	2417600	2437800	11300000	0
714860	1415900	0	0	612510	3637200	837440	3913400	954130
0	1306800	0	0	341720	2693900	1358700	1565200	613190
0	1416700	0	0	467450	0	8967100	4818000	2965700
0	0	0	0	0	10800000	17600000	5621000	1064200
0	0	0	0	0	0	543810	621810	264780
0	0	0	0	0	0	2120800	0	0
0	0	0	546410	424160	0	0	0	0
0	0	0	0	0	5403800	9025800	9678500	0
0	0	0	0	0	40100000	0	0	0
0	0	0	0	0	1850300	0	1795100	0
0	4635300	0	0	0	8271100	0	0	0
0	0	0	0	0	3668100	0	0	0
0	0	0	0	0	1375200	0	0	0
0	997150	0	0	0	0	316600	0	0
0	0	0	0	0	6573900	0	8540600	3644900
0	0	0	0	0	12900000	5030900	5312800	0
0	0	0	0	392250	11200000	6368400	9981500	1369200
0	0	0	0	0	0	0	0	0
0	0	0	0	0	4195900	1688100	2895400	1114300
0	0	0	0	0	0	1911200	1809100	0

0	0	1130500	0	1293400	8858300	16400000	2481800	775710
0	0	0	0	0	1243300	1092400	920370	299080
0	0	0	0	0	1650400	977110	1342000	281150
0	2172000	2585600	0	0	0	2701900	5351400	2662200
0	0	0	0	0	5608000	0	1713500	0
0	0	0	0	402950	8911700	2552600	7303400	1743400
4634000	0	0	1168300	0	15700000	4237900	13700000	6307400
0	0	0	0	0	0	6965700	7946900	1982400
0	0	0	0	0	0	0	0	0
0	489470	0	0	265460	1200100	680350	891580	254730
593200	1350900	0	0	309240	7980200	4238900	6409200	1394800
0	0	0	0	0	1975400	4103100	6424600	0
0	0	0	0	0	0	0	0	0
0	0	0	0	0	0	0	0	0
0	1407800	0	0	1321600	0	0	17900000	4186900
0	0	0	0	0	8829600	8172700	4835100	0
0	0	0	0	0	6460000	2098800	6957000	701920
0	0	0	0	0	19700000	8920800	9369900	1146700
0	2000900	0	0	0	0	0	0	0
6233100	1455000	0	0	0	0	0	0	0
0	0	0	0	0	1.70E+07	7572200	0	4373000
0	0	0	0	0	0	0	0	0
0	0	0	0	0	0	0	2033400	0
0	0	1223300	1146100	0	0	0	0	2638500
0	0	1157500	242050	551190	0	0	0	0
0	0	0	0	0	5432500	3366200	5111600	1506200
0	0	0	0	0	5355800	6082600	4488800	4499300
0	0	0	0	0	15300000	7234800	48800000	13400000
0	0	0	0	0	0	2053000	0	0
0	0	0	0	0	14600000	10700000	13800000	2752700
0	219060	0	0	0	349350	306320	416900	0
0	0	0	0	0	9066200	5590300	7529000	228420
0	0	0	0	0	19800000	9033700	3491700	2713000
0	558880	653270	0	550570	0	0	0	0
0	0	0	0	0	0	6611300	3277200	0
0	0	0	0	0	0	0	13500000	0
12600000	3682700	0	0	0	72600000	63700000	54600000	18700000
0	0	0	0	0	0	0	0	0
0	0	0	0	0	0	0	0	0
0	2061100	0	596540	0	34100000	22500000	39100000	9384200
0	0	0	0	1829700	0	0	0	0
0	0	0	0	0	23600000	0	4738000	1482000
0	790840	0	0	2030400	27900000	16900000	28600000	8547600
0	0	0	0	0	7750500	5022100	5171800	1112300
0	0	2444200	775390	2640600	11500000	2567200	2369700	3633900
0	0	0	0	0	3995900	1680800	3626300	1811700
0	0	0	0	0	973930	1874500	5763900	1145400

0	0	0	0	0	0	0	0	0
0	0	0	0	0	0	0	0	0
0	0	0	0	0	18400000	8317000	10700000	1223300
0	0	0	0	0	296580	0	261950	0
0	931850	9291700	0	0	4058900	2087700	8097200	927240
0	2550800	0	0	0	0	4947700	3998800	1152600
0	0	0	0	0	1209700	813360	8480700	1705000
0	0	0	0	308270	0	1754600	1710800	0
0	0	0	0	0	0	12200000	0	0
0	0	0	0	0	1982000	0	1261400	458190
0	0	0	0	0	3095000	2457400	5356500	1389600
0	5611700	0	0	0	0	0	0	0
0	0	0	0	0	0	1188100	0	0
0	0	0	0	0	0	0	0	0
0	1940600	3907700	0	321540	0	0	0	0
0	1047900	0	373430	455390	0	0	0	0
1133100	1365400	0	0	0	3563000	2608200	3224800	0
0	0	0	0	0	7714600	4995500	10800000	4752000
0	0	0	0	0	14400000	0	0	0
0	0	0	0	0	3998900	1776300	0	2012100
0	0	0	0	0	0	0	0	0
0	0	0	0	0	0	1315800	0	1533400
0	0	0	0	0	0	0	3806700	0
0	0	0	0	973680	11500000	7683200	2153100	1747700
0	0	0	0	0	1.50E+07	1.30E+07	12100000	1993500
0	0	0	0	0	2713700	0	0	0
0	0	0	0	0	24800000	1336500	15900000	7911100
0	0	0	0	0	0	0	2264300	1375800
79200000	43700000	37200000	23800000	29600000	25500000	18700000	12600000	4113400
0	0	0	0	0	12800000	8160000	20500000	6198800
0	0	0	0	0	0	0	0	0
0	0	0	0	0	6945800	3484000	4327700	0
0	0	0	0	0	3448000	12500000	15300000	1060700
0	0	0	0	0	15900000	11900000	13800000	2708800
0	1319500	0	0	0	0	0	0	2932500
0	0	0	0	1258300	2661700	0	0	976270
0	0	0	0	0	401040	2550700	377540	2873400
0	0	0	0	0	0	0	0	0
0	0	0	0	0	0	0	0	0
0	0	0	0	0	2114500	9497100	0	192440
0	0	0	0	0	476530	2244600	3839300	549160
0	0	0	0	0	17700000	3461400	8339600	2885000
0	0	0	0	0	10200000	0	0	0
0	837720	211470	0	1210900	23900000	15200000	6724400	6077000
0	0	0	0	0	4708300	3458400	0	0
0	0	0	0	0	0	0	0	0
0	0	0	0	0	0	2796200	2362700	1073000

0	0	0	0	0	0	0	0	0
0	0	0	0	863580	0	0	0	0
0	0	0	0	0	10400000	0	4679800	5798800
0	0	0	0	0	0	0	0	0
0	0	0	0	0	15600000	7138900	13600000	3779400
0	982670	242370	178360	1219200	2619300	2179700	2258900	891850
0	0	0	0	0	6593400	14200000	18900000	6238800
0	0	0	0	0	2894900	6979000	6978000	0
0	0	0	0	0	0	0	0	0
0	0	0	0	0	0	0	0	0
0	1521900	0	0	0	0	0	0	0
0	0	0	0	0	0	0	0	0
0	0	0	0	0	12200000	6668400	8710400	2616200
0	0	552060	371490	0	0	0	0	0
10700000	0	0	0	0	0	0	0	0
0	0	0	0	0	5285200	3348300	4282200	526700
0	0	0	0	0	0	0	0	2160600
4781400	0	0	0	0	0	0	0	0
0	0	0	0	0	1.10E+07	11500000	0	1708400
0	0	0	0	0	0	0	0	0
0	0	0	0	0	19900000	13100000	15300000	4074200
0	0	0	0	0	0	0	0	0
0	0	0	0	0	10800000	7218100	7480300	3533700
29700000	2950500	11400000	4571800	2664000	0	0	1137700	0
0	1367200	0	0	1322100	0	0	0	0
0	0	944160	0	0	0	0	0	0
0	0	0	0	0	304400	0	0	0
0	0	4324300	0	0	0	0	0	391780
0	0	0	0	0	11600000	3110700	10500000	2707100
0	0	0	0	322020	0	0	0	0
0	0	0	0	0	2321000	1347900	1638600	431160
0	0	0	0	0	7627800	0	4262100	0
0	0	0	0	0	3957000	0	0	0
0	0	0	0	0	9949600	0	4912300	0
0	0	0	0	0	0	0	0	0
0	0	0	0	0	0	0	0	0
0	0	845510	0	0	3143000	0	0	0
0	0	0	0	0	597020	299790	424480	0
0	0	0	0	0	7487900	3354300	8001100	2292300
0	0	0	0	0	4844600	3487800	2722200	980830
0	0	0	0	0	0	0	0	0
0	0	0	0	0	8181300	7518300	7412900	2856600
0	0	0	0	0	7042700	0	2241700	0
0	0	0	0	0	0	2242000	0	0
0	0	0	0	0	11100000	1179200	9458900	3503200
0	0	0	0	290220	2228200	689450	1464400	358150
0	0	0	0	0	0	0	0	0

0	0	0	0	0	0	0	0	0
0	0	0	0	0	0	0	0	0
0	0	0	0	0	0	0	0	0
557320	904990	1408700	0	0	1187700	1528900	0	0
0	0	683400	932790	2491300	4184100	0	0	764590
0	0	0	0	0	6806900	3366800	4346500	0
0	0	0	0	0	6727300	7882800	23500000	3599900
0	0	0	0	0	1778500	0	0	0
0	0	0	0	0	7568800	372050	271770	1118100
0	0	0	0	0	7088200	0	3329900	736400
0	0	0	0	376060	2655800	4727800	2219100	642290
0	0	0	0	0	0	0	0	0
0	0	0	0	0	0	7391800	0	2614700
0	0	0	0	0	20900000	0	0	0
0	0	0	0	0	0	0	0	1225700
0	0	0	0	0	5968000	8506500	3815100	508500
0	0	0	0	0	0	2229500	0	0
0	5980000	0	0	0	0	0	0	0
0	0	0	0	0	0	0	0	0
0	0	0	0	0	0	492730	0	0
0	0	0	0	0	0	0	0	0
0	0	0	0	0	0	0	0	1591400
0	0	0	0	0	1884900	701130	879640	0
0	0	0	0	0	4503700	0	3368900	889900
0	0	0	0	0	17900000	13500000	18700000	5931200
0	0	0	0	0	3861000	16300000	15800000	0
0	0	0	0	0	7720600	5526900	3445100	1230000
0	0	0	0	0	0	0	0	0
0	0	0	0	323720	0	0	0	0
0	0	0	0	0	0	0	5110900	0
0	0	0	0	0	10600000	8164500	0	3565900
0	0	0	0	912900	0	0	0	0
0	0	0	0	0	0	0	0	0
0	0	0	0	0	0	0	0	0
0	0	0	0	0	6358700	4066800	3774700	0
0	0	0	0	0	8728500	5397000	8119800	0
0	0	0	0	0	6148400	3757200	4350000	949670
0	0	0	0	0	9898800	0	0	2081400
0	0	0	0	0	0	0	0	0
0	180690	0	0	0	2627600	2664000	2487200	2109400
0	0	0	0	0	7278700	0	6257200	0
0	0	0	0	0	0	3966000	2099000	0
0	0	0	0	0	0	0	0	0
0	0	0	0	0	0	0	0	0
0	1650700	1428600	0	0	0	2685300	0	0
3261800	6599400	1400700	886430	1039300	0	0	0	0
0	0	0	0	0	0	0	0	0

0	0	0	0	0	0	0	0	0
0	0	0	0	0	0	0	0	0
0	825140	0	0	0	0	0	0	0
0	0	0	0	0	0	0	0	0
0	0	0	0	0	9189800	18300000	7774600	0
0	0	0	0	0	0	0	0	0
0	0	0	0	866690	0	5415200	10600000	0
301240	540650	0	0	390100	1200500	1761400	0	1583700
0	0	0	0	0	0	0	0	0
0	0	0	0	0	0	0	0	4776000
0	0	0	0	0	0	0	0	0
0	0	0	0	0	0	0	0	0
0	0	0	0	0	0	0	0	0
0	1566600	0	0	0	0	0	0	0
0	0	0	0	0	8571100	4506100	0	1542000
0	0	0	0	0	0	0	0	0
0	0	0	0	736490	0	0	0	0
0	0	0	0	0	3710500	2564500	0	0
0	0	0	0	0	0	0	0	1589700
0	0	0	0	0	0	0	0	0
0	0	0	0	0	5779400	0	0	2681200
0	0	0	0	0	0	0	0	929230
0	0	0	0	0	0	0	0	0
0	0	0	0	0	0	1372500	0	0
0	0	0	0	0	0	0	0	0
0	0	0	0	0	0	0	0	0
0	0	0	0	0	0	0	0	0
0	0	0	0	0	4479600	2753700	3478100	2147200
0	0	0	0	0	0	0	0	0
0	0	0	0	0	0	0	0	0
0	0	0	0	0	0	0	0	0
0	0	0	0	0	0	0	0	0
0	0	4483000	0	0	1627500	0	979920	222500
0	0	0	0	0	0	0	0	131640
0	0	0	0	0	0	0	4625600	1552900
0	0	0	0	0	5681400	1842500	1886800	1471500
0	0	0	0	0	0	0	0	0
0	0	0	0	0	0	0	0	0
0	0	0	0	0	0	0	0	0
0	0	0	0	0	0	0	0	0
0	0	0	0	0	0	0	0	963060
0	0	369070	170120	521970	0	0	0	0
0	0	0	0	0	0	0	0	0
0	0	0	0	0	8076100	7277100	9272300	1690000
0	0	0	0	0	0	0	0	0
0	0	0	0	0	0	9858700	0	0
0	0	0	0	0	0	0	0	784790

0	0	0	0	0	0	0	0	0
0	0	0	0	0	0	0	0	0
0	0	0	0	757650	0	0	0	0
0	0	0	0	0	0	0	0	0
0	0	0	0	0	2161100	2127100	0	0
0	0	0	0	0	6905500	5335500	8376100	1822100
0	0	0	0	0	0	0	0	0
0	4708500	0	0	0	23800000	1.60E+07	19900000	1819500
0	0	0	0	0	0	0	0	0
0	0	0	0	0	0	0	0	0
0	0	0	0	0	0	3165500	0	0
0	0	0	0	0	6540900	5090700	5304800	0
0	0	0	0	0	0	0	0	0
0	0	0	0	0	6518000	0	5863600	1545400
0	0	0	0	0	6573100	0	7445000	1220200
0	0	0	0	0	0	0	0	0
0	0	0	0	0	0	2412800	3346500	973970
0	0	0	0	0	0	0	0	0
0	0	0	0	0	0	0	0	588430
3.70E+07	3.10E+07	1.03E+08	65700000	7.70E+07	22100000	18900000	6469300	12800000
0	0	0	0	0	0	0	0	0
0	0	0	0	0	0	0	0	0
0	0	332090	198130	887840	0	0	0	0
0	0	0	0	0	0	0	0	0
0	0	0	0	0	0	0	2874300	0
0	0	0	0	0	9392600	4020200	0	0
0	0	0	0	0	0	0	0	0
0	0	0	0	0	0	0	0	0
0	0	0	0	0	0	0	0	0
0	0	0	0	0	4591500	0	0	1217400
0	0	0	0	0	0	0	0	0
0	0	0	0	0	14600000	0	0	2807400
0	0	0	0	0	0	0	0	0
0	0	0	0	0	0	0	1906400	572910
0	0	0	0	0	0	0	0	0
0	0	0	0	0	0	0	0	0
0	0	0	0	0	0	0	0	0
0	0	0	0	0	0	0	0	0
0	0	0	0	0	0	0	0	0
0	0	0	0	0	0	0	0	0
0	0	0	0	0	0	0	0	0
0	0	0	0	0	8415600	0	2583000	1284100
0	0	0	0	0	0	0	0	0
0	0	0	0	0	2924000	0	0	0
0	0	0	0	0	0	0	0	0
0	0	0	0	1007100	0	0	0	0
0	0	0	0	0	0	0	0	0

0	0	0	0	0	0	0	0	0
0	0	0	0	0	0	0	0	0
12600000	0	0	0	0	67300000	59100000	49700000	15600000
0	0	0	0	0	2870800	0	0	843130
0	0	0	0	0	0	0	0	0
0	0	0	0	0	0	0	0	0
0	0	0	0	0	0	0	0	0
0	0	0	0	0	0	0	0	0
9242300	16200000	0	0	0	1.90E+07	13700000	52200000	7991000
0	0	0	0	0	0	0	0	0
0	0	0	0	0	0	0	0	0
0	0	0	0	359100	0	0	0	0
0	0	0	0	0	0	0	0	0
0	0	275730	0	952840	0	1121300	0	0
0	0	0	0	0	0	0	0	0
0	0	0	0	0	0	0	0	0
0	0	0	0	0	0	0	0	0
0	0	0	0	0	0	0	0	0
10600000	1.60E+07	5589100	2699600	10100000	1.12E+08	61600000	63500000	29800000
0	0	0	0	0	0	0	0	0
0	0	0	0	0	0	1978100	0	0
0	0	0	0	1016100	21600000	16400000	18100000	954720
0	0	0	0	0	0	0	0	0
0	0	0	0	0	0	0	0	0
3.47E+08	4.61E+08	NA	NA	NA	1.18E+09	8.24E+08	8.79E+08	NA
1.40E+09	1.17E+09	NA	NA	NA	2.60E+08	1.01E+09	2.60E+08	NA
2.90E+09	3.80E+09	NA	NA	NA	1.87E+10	1.36E+10	2.10E+10	NA
5.36E+08	5.99E+08	NA	NA	NA	9.17E+08	6.59E+08	5.91E+08	NA
30600000	9.00E+07	NA	NA	NA	5.17E+08	3.84E+08	5.31E+08	NA
3.33E+08	4.69E+08	NA	NA	NA	2.02E+09	1.39E+09	1.69E+09	NA
47100000	57700000	NA	NA	NA	4.97E+08	3.36E+08	3.84E+08	NA
2.25E+09	1.19E+09	NA	NA	NA	1.02E+09	5.67E+08	7.14E+08	NA
0	6792600	NA	NA	NA	1.29E+08	97800000	1.47E+08	NA
47300000	1.18E+08	NA	NA	NA	2.94E+08	1.81E+08	1.58E+08	NA
0	0	NA	NA	NA	0	5055400	106240	NA
7249200	24900000	NA	NA	NA	3.10E+08	2.26E+08	2.65E+08	NA
1.01E+08	50400000	NA	NA	NA	2.98E+08	2.01E+08	2.19E+08	NA
93200000	1.02E+08	NA	NA	NA	1.41E+08	2.40E+08	3.17E+08	NA
23500000	7441500	NA	NA	NA	94700000	61400000	78200000	NA
18600000	27100000	NA	NA	NA	62300000	34100000	6.00E+07	NA
19800000	54800000	NA	NA	NA	2.73E+08	2.02E+08	2.34E+08	NA
12300000	14800000	NA	NA	NA	90700000	51100000	94500000	NA
31900000	49300000	NA	NA	NA	4.74E+08	4.76E+08	3.11E+08	NA
6343000	19200000	NA	NA	NA	60100000	45100000	66600000	NA
353930	10500000	NA	NA	NA	7.00E+07	37500000	48100000	NA
36100000	50800000	NA	NA	NA	99600000	1.05E+08	98600000	NA
9189100	4.00E+07	NA	NA	NA	87600000	1.08E+08	1.25E+08	NA

1208700	1893900	NA	NA	NA	92800000	18400000	36200000	NA
1.70E+07	24300000	NA	NA	NA	1.66E+08	1.26E+08	1.48E+08	NA
30100000	55300000	NA	NA	NA	98100000	78200000	92300000	NA
2151800	3306200	NA	NA	NA	18400000	7454100	14500000	NA
2.30E+07	0	NA	NA	NA	0	7321500	0	NA
1162100	2558300	NA	NA	NA	31800000	39900000	43700000	NA
0	4006800	NA	NA	NA	21700000	24800000	23300000	NA
0	5571400	NA	NA	NA	45500000	3.80E+07	53700000	NA
19500000	2.00E+07	NA	NA	NA	92200000	41700000	57400000	NA
2652600	0	NA	NA	NA	1.92E+08	1.66E+08	2.13E+08	NA
882440	20700000	NA	NA	NA	38500000	0	0	NA
0	4139900	NA	NA	NA	83700000	75900000	68700000	NA
0	0	NA	NA	NA	1.07E+08	63100000	1.02E+08	NA
4462400	14600000	NA	NA	NA	16800000	35600000	32400000	NA
0	0	NA	NA	NA	0	2133900	0	NA
3604300	15200000	NA	NA	NA	2.50E+07	15900000	22400000	NA
0	0	NA	NA	NA	22600000	1.40E+07	1.40E+07	NA
7546500	9876000	NA	NA	NA	37200000	18400000	27700000	NA
0	0	NA	NA	NA	17900000	12300000	27100000	NA
0	0	NA	NA	NA	0	0	0	NA
0	0	NA	NA	NA	3.70E+07	29500000	25700000	NA
0	2432000	NA	NA	NA	3.30E+07	2.70E+07	78300000	NA
2448400	0	NA	NA	NA	52900000	38900000	49700000	NA
0	2758600	NA	NA	NA	18100000	27200000	18900000	NA
1.32E+08	1.64E+08	NA	NA	NA	43600000	25900000	35500000	NA
0	0	NA	NA	NA	22800000	2.90E+07	36400000	NA
0	0	NA	NA	NA	20900000	10800000	6090500	NA
0	0	NA	NA	NA	36300000	17100000	28900000	NA
737760	1124100	NA	NA	NA	20400000	23300000	24700000	NA
2020700	4573600	NA	NA	NA	14200000	22300000	4446600	NA
28700000	1736500	NA	NA	NA	6929300	6081500	2381300	NA
3900800	4103900	NA	NA	NA	48200000	3.10E+07	39700000	NA
0	0	NA	NA	NA	0	0	0	NA
0	0	NA	NA	NA	3067000	3448200	0	NA
0	0	NA	NA	NA	0	0	0	NA
0	2445500	NA	NA	NA	0	0	1965000	NA
0	0	NA	NA	NA	41300000	11300000	11100000	NA
0	0	NA	NA	NA	0	0	0	NA
1.11E+09	0	NA	NA	NA	0	0	0	NA
0	0	NA	NA	NA	32100000	6626400	9489300	NA
0	0	NA	NA	NA	0	0	0	NA
0	0	NA	NA	NA	20700000	5964200	9879900	NA
896450	608630	NA	NA	NA	660590	5018300	4784900	NA
5.33E+08	6.15E+08	NA	NA	NA	1.49E+08	1.23E+08	2.83E+08	NA
0	0	NA	NA	NA	0	0	0	NA
40700000	763340	NA	NA	NA	0	1.40E+07	22600000	NA
0	0	NA	NA	NA	0	0	3491200	NA

8464300	286300	NA	NA	NA	0	0	0	NA
0	0	NA	NA	NA	0	0	0	NA
0	0	NA	NA	NA	2149600	1302700	1540900	NA
0	0	NA	NA	NA	7076500	7107100	7473400	NA
3183500	5411700	NA	NA	NA	1.20E+07	7621200	9051700	NA
0	0	NA	NA	NA	0	0	0	NA
0	0	NA	NA	NA	40400000	0	35600000	NA
1784300	0	NA	NA	NA	0	2584800	2206800	NA
0	0	NA	NA	NA	21900000	9163000	14100000	NA
0	1026800	NA	NA	NA	12500000	7918100	15700000	NA
0	0	NA	NA	NA	11300000	3010500	2797100	NA
1634600	4369500	NA	NA	NA	0	0	0	NA
0	0	NA	NA	NA	0	0	0	NA
0	0	NA	NA	NA	16900000	6327600	11900000	NA
0	0	NA	NA	NA	17600000	9970300	8778300	NA
0	0	NA	NA	NA	0	0	0	NA
0	646250	NA	NA	NA	10100000	6642000	7202100	NA
0	0	NA	NA	NA	2703600	6060200	8536800	NA
0	1333900	NA	NA	NA	6195100	5814200	6239800	NA
0	612710	NA	NA	NA	5103800	3569800	4150300	NA
0	0	NA	NA	NA	24700000	3458200	16900000	NA
0	0	NA	NA	NA	0	202330	486120	NA
0	2.48E+08	NA	NA	NA	0	11400000	0	NA
0	0	NA	NA	NA	0	0	0	NA
0	0	NA	NA	NA	0	0	0	NA
0	0	NA	NA	NA	0	0	4015000	NA
0	0	NA	NA	NA	4571600	0	8148700	NA
0	0	NA	NA	NA	16400000	12600000	24300000	NA
5.33E+08	3.77E+08	NA	NA	NA	0	0	0	NA
0	0	NA	NA	NA	811330	0	0	NA
0	0	NA	NA	NA	9899500	10800000	17800000	NA
0	0	NA	NA	NA	4922400	5716100	4198800	NA
0	0	NA	NA	NA	5869800	3082400	4999500	NA
0	0	NA	NA	NA	3.30E+07	8974300	24800000	NA
0	0	NA	NA	NA	8952700	0	9511200	NA
0	0	NA	NA	NA	0	0	5191200	NA
0	0	NA	NA	NA	0	2843900	9909400	NA
0	0	NA	NA	NA	0	321340	0	NA
0	0	NA	NA	NA	0	11400000	18400000	NA
0	0	NA	NA	NA	361560	0	327940	NA
0	0	NA	NA	NA	0	0	0	NA
0	0	NA	NA	NA	0	0	0	NA
0	0	NA	NA	NA	21200000	0	10400000	NA
0	0	NA	NA	NA	15400000	4038500	13300000	NA
0	0	NA	NA	NA	0	3661200	4343900	NA
0	0	NA	NA	NA	0	0	0	NA
0	0	NA	NA	NA	0	0	0	NA

0	0	NA	NA	NA	0	0	0	NA
0	1398700	NA	NA	NA	0	0	0	NA
0	3355800	NA	NA	NA	2331600	1961900	0	NA
0	0	NA	NA	NA	8894000	1.70E+07	6983800	NA
0	0	NA	NA	NA	4949800	0	4022200	NA
0	0	NA	NA	NA	0	0	0	NA
4116600	4663400	NA	NA	NA	0	0	0	NA
0	0	NA	NA	NA	10600000	6776800	4493300	NA
0	0	NA	NA	NA	5771500	0	0	NA
0	0	NA	NA	NA	0	0	0	NA
0	0	NA	NA	NA	37100000	23600000	29900000	NA
25200000	21100000	NA	NA	NA	2.50E+07	14800000	19200000	NA
0	330940	NA	NA	NA	523110	353500	537610	NA
0	0	NA	NA	NA	8336900	633740	4657100	NA
0	147260	NA	NA	NA	11600000	4634400	8106300	NA
749050	753700	NA	NA	NA	0	0	0	NA
0	1459100	NA	NA	NA	4103500	3423400	4552500	NA
0	0	NA	NA	NA	3865100	639790	0	NA
0	0	NA	NA	NA	3633700	10100000	3827100	NA
0	1.40E+07	NA	NA	NA	0	0	0	NA
0	0	NA	NA	NA	0	0	0	NA
0	0	NA	NA	NA	0	0	0	NA
0	428140	NA	NA	NA	3963200	514400	0	NA
50500000	85800000	NA	NA	NA	1.47E+08	97500000	1.07E+08	NA
0	0	NA	NA	NA	6833300	7053100	6636000	NA
0	0	NA	NA	NA	0	0	0	NA
0	0	NA	NA	NA	0	0	0	NA
0	0	NA	NA	NA	0	0	4604100	NA
0	0	NA	NA	NA	0	0	0	NA
0	0	NA	NA	NA	0	0	0	NA
0	0	NA	NA	NA	5768800	3735400	4338700	NA
0	0	NA	NA	NA	5398500	0	4300000	NA
0	0	NA	NA	NA	0	0	0	NA
3115700	0	NA	NA	NA	0	0	0	NA
2262500	594620	NA	NA	NA	899760	491200	0	NA
0	0	NA	NA	NA	0	0	0	NA
0	0	NA	NA	NA	10800000	6549700	15500000	NA
403840	0	NA	NA	NA	0	0	3004800	NA
0	0	NA	NA	NA	0	0	0	NA
0	0	NA	NA	NA	10500000	6423500	4657100	NA
0	0	NA	NA	NA	33300000	27500000	33200000	NA
497230	892510	NA	NA	NA	1757300	462800	1213400	NA
0	0	NA	NA	NA	5217900	0	0	NA
0	0	NA	NA	NA	0	0	0	NA
0	0	NA	NA	NA	0	2039300	4241900	NA
0	0	NA	NA	NA	0	0	0	NA
0	514210	NA	NA	NA	0	0	0	NA

0	1465000	NA	NA	NA	0	0	0	NA
0	0	NA	NA	NA	0	291520	516860	NA
0	0	NA	NA	NA	0	0	0	NA
0	0	NA	NA	NA	0	0	0	NA
0	0	NA	NA	NA	0	0	0	NA
0	0	NA	NA	NA	0	0	2432500	NA
0	0	NA	NA	NA	0	0	312160	NA
0	506380	NA	NA	NA	1448800	1396500	1307500	NA
0	0	NA	NA	NA	723450	534910	806910	NA
0	0	NA	NA	NA	0	4680600	0	NA
0	0	NA	NA	NA	0	0	0	NA
0	0	NA	NA	NA	1841300	960720	0	NA
0	0	NA	NA	NA	7143700	0	0	NA
0	0	NA	NA	NA	3233800	0	0	NA
0	3734800	NA	NA	NA	0	0	0	NA
0	0	NA	NA	NA	2986700	7528400	2988300	NA
0	0	NA	NA	NA	0	1186900	0	NA
0	352940	NA	NA	NA	0	0	0	NA
0	0	NA	NA	NA	8611500	1808800	0	NA
0	0	NA	NA	NA	3384400	2394400	3707100	NA
0	0	NA	NA	NA	1595800	696530	323130	NA
0	0	NA	NA	NA	0	0	0	NA
0	0	NA	NA	NA	0	0	0	NA
0	0	NA	NA	NA	6151900	6789200	9033500	NA
0	0	NA	NA	NA	0	0	0	NA
0	0	NA	NA	NA	0	0	0	NA
0	0	NA	NA	NA	2596800	0	0	NA
0	0	NA	NA	NA	0	0	0	NA
0	0	NA	NA	NA	3206000	0	2640300	NA
0	0	NA	NA	NA	3795600	0	2745800	NA
0	0	NA	NA	NA	0	0	0	NA
0	0	NA	NA	NA	0	4670000	4307100	NA
0	0	NA	NA	NA	0	0	0	NA
0	0	NA	NA	NA	0	0	0	NA
0	0	NA	NA	NA	1233700	449220	570910	NA
0	0	NA	NA	NA	8856600	4198000	5084300	NA
0	0	NA	NA	NA	0	0	0	NA
0	0	NA	NA	NA	0	0	0	NA
2304000	0	NA	NA	NA	0	0	0	NA
0	0	NA	NA	NA	0	0	6062000	NA
0	0	NA	NA	NA	0	0	0	NA
0	0	NA	NA	NA	0	0	0	NA
0	0	NA	NA	NA	0	0	5667600	NA
0	0	NA	NA	NA	496430	220730	471290	NA
0	1541400	NA	NA	NA	1017900	0	0	NA
0	0	NA	NA	NA	0	0	0	NA
0	0	NA	NA	NA	0	2337300	0	NA

0	0	NA	NA	NA	6844900	4625500	4346600	NA
0	0	NA	NA	NA	0	0	0	NA
0	0	NA	NA	NA	0	0	0	NA
0	0	NA	NA	NA	0	0	0	NA
0	0	NA	NA	NA	6005600	641330	7313900	NA
0	0	NA	NA	NA	0	0	0	NA
0	0	NA	NA	NA	0	0	0	NA
0	0	NA	NA	NA	0	0	0	NA
0	0	NA	NA	NA	0	0	2369900	NA
0	0	NA	NA	NA	0	0	3778500	NA
8156000	0	NA	NA	NA	0	0	0	NA
0	0	NA	NA	NA	2591200	2263500	2770400	NA
0	0	NA	NA	NA	0	0	0	NA
0	0	NA	NA	NA	0	0	2.30E+07	NA
0	0	NA	NA	NA	0	0	3865900	NA
0	0	NA	NA	NA	0	0	0	NA
0	0	NA	NA	NA	960420	157950	415450	NA
0	0	NA	NA	NA	0	0	0	NA
0	0	NA	NA	NA	7391100	4009500	6490600	NA
0	0	NA	NA	NA	7235200	0	6731400	NA
0	0	NA	NA	NA	0	0	0	NA
0	0	NA	NA	NA	0	0	0	NA
0	0	NA	NA	NA	0	0	0	NA
0	0	NA	NA	NA	0	0	0	NA
0	0	NA	NA	NA	0	0	0	NA
0	0	NA	NA	NA	0	0	0	NA
0	0	NA	NA	NA	0	671760	0	NA
0	0	NA	NA	NA	0	1427700	0	NA
0	0	NA	NA	NA	0	307080	0	NA
0	0	NA	NA	NA	0	0	0	NA
0	0	NA	NA	NA	0	0	0	NA
0	0	NA	NA	NA	0	0	0	NA
0	0	NA	NA	NA	0	0	0	NA
0	0	NA	NA	NA	0	0	4850200	NA
0	645950	NA	NA	NA	0	0	0	NA
0	0	NA	NA	NA	0	0	0	NA
0	0	NA	NA	NA	0	0	0	NA
0	0	NA	NA	NA	0	0	0	NA
0	0	NA	NA	NA	0	0	0	NA
0	0	NA	NA	NA	0	2359500	2150100	NA
0	0	NA	NA	NA	0	0	0	NA
0	0	NA	NA	NA	0	0	0	NA
0	0	NA	NA	NA	1474400	1021700	1196100	NA
0	0	NA	NA	NA	0	0	0	NA
0	0	NA	NA	NA	0	0	206990	NA
0	0	NA	NA	NA	0	0	0	NA
0	0	NA	NA	NA	0	0	0	NA

0	0	NA	NA	NA	0	0	0	NA
0	0	NA	NA	NA	0	0	0	NA
0	0	NA	NA	NA	0	0	0	NA
0	4192200	NA	NA	NA	0	0	0	NA
0	0	NA	NA	NA	0	0	0	NA
0	0	NA	NA	NA	13500000	7040800	8761200	NA
0	0	NA	NA	NA	0	0	0	NA
0	0	NA	NA	NA	0	0	0	NA
0	0	NA	NA	NA	0	0	0	NA
0	403360	NA	NA	NA	6959100	10400000	4751900	NA
0	0	NA	NA	NA	0	0	0	NA
0	0	NA	NA	NA	0	0	0	NA
0	0	NA	NA	NA	0	0	0	NA
0	0	NA	NA	NA	10800000	6549700	8175400	NA
0	0	NA	NA	NA	0	0	0	NA
0	0	NA	NA	NA	0	0	0	NA
0	0	NA	NA	NA	0	0	0	NA
0	0	NA	NA	NA	0	0	0	NA
0	0	NA	NA	NA	0	0	0	NA
0	0	NA	NA	NA	0	2337300	0	NA
0	0	NA	NA	NA	0	0	0	NA
0	0	NA	NA	NA	0	0	0	NA
0	0	NA	NA	NA	0	0	0	NA
0	0	NA	NA	NA	4718300	2102900	2762500	NA
0	0	NA	NA	NA	0	0	0	NA
0	0	NA	NA	NA	0	0	0	NA
0	0	NA	NA	NA	0	0	0	NA
0	6460100	NA	NA	NA	53700000	1.60E+07	41600000	NA
0	0	NA	NA	NA	5363700	3328000	0	NA
0	0	NA	NA	NA	0	0	0	NA
0	0	NA	NA	NA	0	0	0	NA
0	0	NA	NA	NA	0	0	0	NA
0	0	NA	NA	NA	0	0	0	NA
0	0	NA	NA	NA	0	0	0	NA
0	0	NA	NA	NA	0	0	0	NA
0	0	NA	NA	NA	0	0	0	NA
0	0	NA	NA	NA	0	0	0	NA
0	0	NA	NA	NA	0	0	0	NA
0	0	NA	NA	NA	2.20E+07	14900000	12700000	NA
0	0	NA	NA	NA	9974300	0	0	NA
0	0	NA	NA	NA	4883900	4310000	0	NA
0	0	NA	NA	NA	0	3532600	0	NA
0	0	NA	NA	NA	0	0	0	NA
0	0	NA	NA	NA	0	0	0	NA
2837800	0	NA	NA	NA	0	0	0	NA
0	0	NA	NA	NA	0	0	0	NA
0	0	NA	NA	NA	0	0	0	NA

0	0	NA	NA	NA	0	0	0	NA
0	0	NA	NA	NA	0	0	0	NA
0	0	NA	NA	NA	0	0	0	NA
0	0	NA	NA	NA	0	0	0	NA
0	0	NA	NA	NA	0	0	0	NA
0	0	NA	NA	NA	0	0	0	NA
0	0	NA	NA	NA	0	0	0	NA
0	0	NA	NA	NA	0	0	0	NA
0	0	NA	NA	NA	0	0	0	NA
0	0	NA	NA	NA	0	0	0	NA
0	0	NA	NA	NA	0	0	0	NA
21900000	0	NA	NA	NA	3661400	30300000	32200000	NA
0	2566500	NA	NA	NA	19300000	12400000	16300000	NA
0	0	NA	NA	NA	7326900	5429700	5798100	NA
0	0	NA	NA	NA	17200000	17700000	15400000	NA
0	0	NA	NA	NA	6597200	3108000	4476800	NA
0	0	NA	NA	NA	0	0	0	NA
0	0	NA	NA	NA	0	0	0	NA
0	0	NA	NA	NA	0	12200000	0	NA
0	0	NA	NA	NA	0	0	0	NA
0	0	NA	NA	NA	0	0	0	NA
0	0	NA	NA	NA	3518600	2798600	2731700	NA
0	0	NA	NA	NA	0	0	0	NA
0	0	NA	NA	NA	0	0	0	NA
0	0	NA	NA	NA	0	0	0	NA
0	0	NA	NA	NA	2866400	0	0	NA
0	0	NA	NA	NA	0	0	5782000	NA
1414600	0	NA	NA	NA	0	492380	0	NA
0	0	NA	NA	NA	0	0	0	NA
0	0	NA	NA	NA	0	0	0	NA
0	0	NA	NA	NA	0	0	0	NA
0	726640	NA	NA	NA	0	0	0	NA
0	0	NA	NA	NA	0	0	0	NA
0	0	NA	NA	NA	0	0	0	NA
0	0	NA	NA	NA	0	0	0	NA
0	0	NA	NA	NA	0	0	0	NA
0	12300000	NA	NA	NA	98200000	60800000	98600000	NA
0	0	NA	NA	NA	91354	280080	410600	NA
0	0	NA	NA	NA	0	0	0	NA
0	0	NA	NA	NA	0	5383500	5858700	NA
NA	NA	1.21E+08	86300000	1.80E+08	NA	NA	NA	2.90E+08
NA	NA	1.20E+09	7.81E+08	2.10E+09	NA	NA	NA	5.05E+09
NA	NA	89400000	94400000	2.40E+08	NA	NA	NA	5.21E+08
NA	NA	1.32E+08	1.08E+08	2.23E+08	NA	NA	NA	1.97E+08
NA	NA	14600000	8984800	23900000	NA	NA	NA	1.08E+08
NA	NA	10600000	9907700	43100000	NA	NA	NA	1.25E+08
NA	NA	14900000	12600000	60200000	NA	NA	NA	75600000

NA	NA	3.86E+08	2.89E+08	6.22E+08	NA	NA	NA	2.10E+08
NA	NA	2171500	0	0	NA	NA	NA	0
NA	NA	0	392960	3730700	NA	NA	NA	28300000
NA	NA	1169000	1121400	13900000	NA	NA	NA	79200000
NA	NA	38200000	24900000	36400000	NA	NA	NA	81200000
NA	NA	4265100	4347200	9623600	NA	NA	NA	14100000
NA	NA	32500000	21600000	61600000	NA	NA	NA	1.04E+08
NA	NA	9175700	6357700	13900000	NA	NA	NA	22500000
NA	NA	13600000	8010100	24400000	NA	NA	NA	1.34E+08
NA	NA	3113000	5165000	21500000	NA	NA	NA	23800000
NA	NA	11900000	3131700	19600000	NA	NA	NA	85600000
NA	NA	0	1443800	5666600	NA	NA	NA	13400000
NA	NA	921580	1029200	3589200	NA	NA	NA	18700000
NA	NA	21900000	17800000	37100000	NA	NA	NA	69900000
NA	NA	1130500	1114500	2310400	NA	NA	NA	5325700
NA	NA	17900000	11100000	14900000	NA	NA	NA	42900000
NA	NA	0	0	0	NA	NA	NA	5339700
NA	NA	11300000	7995800	28200000	NA	NA	NA	20700000
NA	NA	0	0	0	NA	NA	NA	20700000
NA	NA	0	0	2713600	NA	NA	NA	27800000
NA	NA	6713600	4179600	5175900	NA	NA	NA	2924200
NA	NA	0	0	663710	NA	NA	NA	4414100
NA	NA	267370	254980	1282300	NA	NA	NA	23600000
NA	NA	1.20E+07	9311400	16500000	NA	NA	NA	11100000
NA	NA	232780	0	3493300	NA	NA	NA	5229900
NA	NA	505860	0	0	NA	NA	NA	6396700
NA	NA	0	5175300	0	NA	NA	NA	0
NA	NA	620100	606320	2329900	NA	NA	NA	44400000
NA	NA	0	0	0	NA	NA	NA	12600000
NA	NA	0	0	0	NA	NA	NA	6528200
NA	NA	3021300	4009400	6880200	NA	NA	NA	14300000
NA	NA	974240	0	2371900	NA	NA	NA	5120500
NA	NA	0	0	0	NA	NA	NA	5305300
NA	NA	3839200	3557400	6218300	NA	NA	NA	8223100
NA	NA	0	3238800	2112900	NA	NA	NA	12100000
NA	NA	0	0	1087300	NA	NA	NA	8521600
NA	NA	1730000	594520	515030	NA	NA	NA	1369300
NA	NA	0	0	0	NA	NA	NA	2699300
NA	NA	1106300	563700	3447900	NA	NA	NA	1042200
NA	NA	0	0	0	NA	NA	NA	7580700
NA	NA	0	569180	807270	NA	NA	NA	3838900
NA	NA	0	0	0	NA	NA	NA	0
NA	NA	0	0	0	NA	NA	NA	9908500
NA	NA	0	0	404440	NA	NA	NA	0
NA	NA	0	0	0	NA	NA	NA	6004900
NA	NA	0	0	0	NA	NA	NA	4002300
NA	NA	754830	246190	0	NA	NA	NA	6957300

NA	NA	1935300	750070	1603000	NA	NA	NA	1600500
NA	NA	0	0	0	NA	NA	NA	0
NA	NA	9783400	0	0	NA	NA	NA	0
NA	NA	0	0	0	NA	NA	NA	3465800
NA	NA	0	0	0	NA	NA	NA	0
NA	NA	0	0	10700000	NA	NA	NA	0
NA	NA	9865000	2441400	5173300	NA	NA	NA	0
NA	NA	0	0	0	NA	NA	NA	5205300
NA	NA	0	0	0	NA	NA	NA	3481300
NA	NA	0	0	0	NA	NA	NA	0
NA	NA	1976200	1148700	7085100	NA	NA	NA	15100000
NA	NA	0	0	0	NA	NA	NA	1698800
NA	NA	0	0	0	NA	NA	NA	3860700
NA	NA	0	0	0	NA	NA	NA	8063300
NA	NA	0	0	0	NA	NA	NA	5660600
NA	NA	373520	0	318530	NA	NA	NA	304520
NA	NA	0	0	0	NA	NA	NA	3640600
NA	NA	0	0	0	NA	NA	NA	2464000
NA	NA	2791600	2603400	2496600	NA	NA	NA	583690
NA	NA	0	0	0	NA	NA	NA	4925800
NA	NA	0	0	0	NA	NA	NA	2396100
NA	NA	0	0	1799300	NA	NA	NA	1922800
NA	NA	0	0	0	NA	NA	NA	1317900
NA	NA	0	0	0	NA	NA	NA	350700
NA	NA	0	0	978660	NA	NA	NA	0
NA	NA	6734100	2.14E+08	0	NA	NA	NA	0
NA	NA	748020	556360	2233200	NA	NA	NA	14100000
NA	NA	1080700	966600	608430	NA	NA	NA	3638400
NA	NA	0	0	0	NA	NA	NA	0
NA	NA	0	0	0	NA	NA	NA	11900000
NA	NA	0	0	0	NA	NA	NA	0
NA	NA	0	0	3412400	NA	NA	NA	0
NA	NA	0	0	0	NA	NA	NA	4083500
NA	NA	1535800	0	761460	NA	NA	NA	453210
NA	NA	0	0	0	NA	NA	NA	3923100
NA	NA	0	0	0	NA	NA	NA	825020
NA	NA	0	0	0	NA	NA	NA	13300000
NA	NA	435880	341050	865280	NA	NA	NA	0
NA	NA	0	0	0	NA	NA	NA	593450
NA	NA	0	0	0	NA	NA	NA	1097800
NA	NA	410720	319110	0	NA	NA	NA	1856400
NA	NA	0	0	0	NA	NA	NA	1652800
NA	NA	0	0	0	NA	NA	NA	4227500
NA	NA	0	0	0	NA	NA	NA	0
NA	NA	0	0	2364800	NA	NA	NA	1388400
NA	NA	791230	0	0	NA	NA	NA	3172300
NA	NA	0	0	635160	NA	NA	NA	4976000

NA	NA	0	28800000	0	NA	NA	NA	1515100
NA	NA	423380	0	0	NA	NA	NA	0
NA	NA	0	0	0	NA	NA	NA	1871200
NA	NA	0	0	0	NA	NA	NA	300640
NA	NA	0	0	0	NA	NA	NA	0
NA	NA	0	215330	0	NA	NA	NA	6597000
NA	NA	0	0	0	NA	NA	NA	0
NA	NA	0	0	0	NA	NA	NA	0
NA	NA	0	0	0	NA	NA	NA	0
NA	NA	557240	0	0	NA	NA	NA	0
NA	NA	0	0	0	NA	NA	NA	0
NA	NA	379130	380910	0	NA	NA	NA	1534200
NA	NA	0	0	0	NA	NA	NA	1801700
NA	NA	0	0	0	NA	NA	NA	7965800
NA	NA	0	0	0	NA	NA	NA	7884800
NA	NA	0	0	0	NA	NA	NA	8442900
NA	NA	0	0	0	NA	NA	NA	559980
NA	NA	0	0	0	NA	NA	NA	1563700
NA	NA	0	0	0	NA	NA	NA	0
NA	NA	0	0	0	NA	NA	NA	0
NA	NA	1016600	0	1650900	NA	NA	NA	5461400
NA	NA	0	0	0	NA	NA	NA	2004700
NA	NA	0	0	0	NA	NA	NA	0
NA	NA	0	0	0	NA	NA	NA	3928700
NA	NA	0	0	0	NA	NA	NA	3208000
NA	NA	0	0	0	NA	NA	NA	0
NA	NA	0	0	0	NA	NA	NA	3755400
NA	NA	0	0	0	NA	NA	NA	0
NA	NA	755720	454710	1013200	NA	NA	NA	707720
NA	NA	0	0	0	NA	NA	NA	0
NA	NA	0	0	0	NA	NA	NA	0
NA	NA	0	3976500	0	NA	NA	NA	275050
NA	NA	0	0	0	NA	NA	NA	1714900
NA	NA	0	852950	0	NA	NA	NA	2994800
NA	NA	486260	0	0	NA	NA	NA	1721800
NA	NA	0	0	0	NA	NA	NA	565450
NA	NA	0	0	0	NA	NA	NA	0
NA	NA	0	0	0	NA	NA	NA	0
NA	NA	0	0	0	NA	NA	NA	379640
NA	NA	0	0	0	NA	NA	NA	2060400
NA	NA	0	0	2.80E+07	NA	NA	NA	0
NA	NA	0	0	0	NA	NA	NA	0
NA	NA	552660	0	0	NA	NA	NA	0
NA	NA	0	0	0	NA	NA	NA	1756600
NA	NA	0	0	0	NA	NA	NA	0
NA	NA	0	0	0	NA	NA	NA	1645800
NA	NA	0	0	0	NA	NA	NA	830060

NA	NA	0	0	0	NA	NA	NA	275440
NA	NA	0	0	0	NA	NA	NA	820170
NA	NA	0	0	0	NA	NA	NA	0
NA	NA	0	0	93852	NA	NA	NA	0
NA	NA	0	0	0	NA	NA	NA	0
NA	NA	0	0	0	NA	NA	NA	0
NA	NA	0	0	3278200	NA	NA	NA	0
NA	NA	0	0	0	NA	NA	NA	1107300
NA	NA	748020	556360	2233200	NA	NA	NA	14100000
NA	NA	0	0	0	NA	NA	NA	0
NA	NA	0	0	0	NA	NA	NA	2053000
NA	NA	0	0	0	NA	NA	NA	0
NA	NA	0	0	0	NA	NA	NA	0
NA	NA	0	0	0	NA	NA	NA	0
NA	NA	0	0	0	NA	NA	NA	0
NA	NA	0	0	1183800	NA	NA	NA	3117000
NA	NA	0	0	0	NA	NA	NA	0
NA	NA	0	0	0	NA	NA	NA	1150000
NA	NA	0	0	0	NA	NA	NA	1605000
NA	NA	0	0	0	NA	NA	NA	0
NA	NA	0	0	0	NA	NA	NA	1038200
NA	NA	0	0	0	NA	NA	NA	0
NA	NA	0	0	0	NA	NA	NA	3883500
NA	NA	0	0	751310	NA	NA	NA	1117400
NA	NA	0	0	0	NA	NA	NA	1163900
NA	NA	0	0	0	NA	NA	NA	0
NA	NA	0	0	0	NA	NA	NA	0
NA	NA	0	0	0	NA	NA	NA	0
NA	NA	0	0	0	NA	NA	NA	0
NA	NA	0	0	0	NA	NA	NA	0
NA	NA	0	0	0	NA	NA	NA	1327700
NA	NA	0	0	0	NA	NA	NA	0
NA	NA	0	0	359200	NA	NA	NA	0
NA	NA	0	0	0	NA	NA	NA	0
NA	NA	0	0	0	NA	NA	NA	0
NA	NA	0	0	449040	NA	NA	NA	0
NA	NA	0	934260	1644500	NA	NA	NA	0
NA	NA	0	0	0	NA	NA	NA	2392800
NA	NA	0	0	0	NA	NA	NA	0
NA	NA	4384200	0	0	NA	NA	NA	0
NA	NA	0	0	0	NA	NA	NA	0
NA	NA	0	0	0	NA	NA	NA	0
NA	NA	3276300	1091000	2395500	NA	NA	NA	0
NA	NA	0	0	0	NA	NA	NA	257570
NA	NA	0	0	0	NA	NA	NA	856280
NA	NA	0	0	0	NA	NA	NA	1877300
NA	NA	0	0	874330	NA	NA	NA	0

NA	NA	0	0	0	NA	NA	NA	0
NA	NA	0	0	0	NA	NA	NA	0
NA	NA	7066900	0	4351900	NA	NA	NA	0
NA	NA	0	0	0	NA	NA	NA	0
NA	NA	0	0	0	NA	NA	NA	1326100
NA	NA	0	0	0	NA	NA	NA	2322800
NA	NA	9882200	8852900	23100000	NA	NA	NA	1.54E+08
NA	NA	0	0	0	NA	NA	NA	0
NA	NA	0	0	0	NA	NA	NA	0
NA	NA	0	0	0	NA	NA	NA	0
NA	NA	0	0	0	NA	NA	NA	0
NA	NA	2.23E+08	45600000	4.47E+08	NA	NA	NA	4.55E+08
NA	NA	0	0	0	NA	NA	NA	1739700
NA	NA	0	0	0	NA	NA	NA	0
NA	NA	0	0	0	NA	NA	NA	0
NA	NA	0	0	0	NA	NA	NA	0
NA	NA	0	0	0	NA	NA	NA	0
NA	NA	0	0	0	NA	NA	NA	0
NA	NA	0	0	0	NA	NA	NA	1025700
NA	NA	0	0	0	NA	NA	NA	983110
NA	NA	0	0	0	NA	NA	NA	0
NA	NA	0	0	0	NA	NA	NA	659820
NA	NA	0	0	0	NA	NA	NA	1167800
NA	NA	0	0	0	NA	NA	NA	0
NA	NA	0	0	0	NA	NA	NA	0
NA	NA	0	0	0	NA	NA	NA	0
NA	NA	0	0	0	NA	NA	NA	0
NA	NA	0	0	0	NA	NA	NA	0
NA	NA	0	0	259870	NA	NA	NA	0
NA	NA	0	0	376740	NA	NA	NA	824300
NA	NA	0	0	0	NA	NA	NA	0
NA	NA	0	0	0	NA	NA	NA	0
NA	NA	0	0	0	NA	NA	NA	0
NA	NA	0	0	0	NA	NA	NA	0
NA	NA	0	0	0	NA	NA	NA	0
NA	NA	0	0	0	NA	NA	NA	0
NA	NA	0	0	0	NA	NA	NA	0
NA	NA	0	0	0	NA	NA	NA	0
NA	NA	0	0	0	NA	NA	NA	0
NA	NA	0	0	0	NA	NA	NA	0
NA	NA	0	0	0	NA	NA	NA	0
NA	NA	0	0	0	NA	NA	NA	0
NA	NA	762110	0	0	NA	NA	NA	0
NA	NA	0	0	0	NA	NA	NA	0
NA	NA	0	0	0	NA	NA	NA	0
NA	NA	0	0	0	NA	NA	NA	690310
NA	NA	0	0	0	NA	NA	NA	0

NA	NA	0	0	0	NA	NA	NA	0
NA	NA	0	0	0	NA	NA	NA	0
NA	NA	0	0	0	NA	NA	NA	0
NA	NA	0	0	0	NA	NA	NA	0
NA	NA	0	0	0	NA	NA	NA	0
NA	NA	0	0	0	NA	NA	NA	0
NA	NA	0	0	0	NA	NA	NA	0
NA	NA	0	1139400	0	NA	NA	NA	0
NA	NA	0	0	0	NA	NA	NA	3706200
NA	NA	0	1235300	3136900	NA	NA	NA	0
NA	NA	0	0	0	NA	NA	NA	0
NA	NA	0	0	0	NA	NA	NA	2983500
NA	NA	0	0	0	NA	NA	NA	0
NA	NA	0	0	0	NA	NA	NA	0
NA	NA	0	0	0	NA	NA	NA	0
NA	NA	0	0	0	NA	NA	NA	2673700
NA	NA	0	0	0	NA	NA	NA	0
NA	NA	0	0	0	NA	NA	NA	0
NA	NA	0	0	0	NA	NA	NA	0
NA	NA	0	0	838560	NA	NA	NA	0
NA	NA	0	0	0	NA	NA	NA	0
NA	NA	0	0	0	NA	NA	NA	0
NA	NA	0	0	0	NA	NA	NA	0
NA	NA	0	0	0	NA	NA	NA	0
NA	NA	0	0	0	NA	NA	NA	232920
NA	NA	0	0	0	NA	NA	NA	0
NA	NA	0	0	0	NA	NA	NA	0
NA	NA	0	0	0	NA	NA	NA	0
NA	NA	0	0	0	NA	NA	NA	0
NA	NA	0	0	0	NA	NA	NA	0
NA	NA	0	0	0	NA	NA	NA	0
NA	NA	1400100	863620	0	NA	NA	NA	10300000
NA	NA	0	0	0	NA	NA	NA	0
NA	NA	0	0	0	NA	NA	NA	0
NA	NA	0	0	0	NA	NA	NA	0
NA	NA	7203100	5635400	11100000	NA	NA	NA	2.50E+07
NA	NA	0	0	1073100	NA	NA	NA	0
NA	NA	0	0	0	NA	NA	NA	0
NA	NA	0	0	0	NA	NA	NA	0
NA	NA	0	0	0	NA	NA	NA	0
NA	NA	0	0	0	NA	NA	NA	0
NA	NA	33600000	20100000	19300000	NA	NA	NA	2613200
NA	NA	0	0	0	NA	NA	NA	0
NA	NA	0	0	0	NA	NA	NA	0
NA	NA	755720	454710	1013200	NA	NA	NA	707720
NA	NA	0	0	0	NA	NA	NA	4632800
NA	NA	0	0	0	NA	NA	NA	1537500

NA	NA	0	0	0	NA	NA	NA	0
NA	NA	0	0	0	NA	NA	NA	0
NA	NA	0	0	0	NA	NA	NA	0
NA	NA	0	0	0	NA	NA	NA	0
NA	NA	0	0	0	NA	NA	NA	0
NA	NA	0	0	0	NA	NA	NA	0
NA	NA	0	0	0	NA	NA	NA	0
NA	NA	0	0	0	NA	NA	NA	0
NA	NA	0	0	0	NA	NA	NA	2808800
NA	NA	0	0	0	NA	NA	NA	0
NA	NA	0	0	0	NA	NA	NA	0
NA	NA	0	0	0	NA	NA	NA	1200900
NA	NA	0	0	0	NA	NA	NA	0
NA	NA	0	0	0	NA	NA	NA	0
NA	NA	0	0	0	NA	NA	NA	0
NA	NA	289930	228380	354210	NA	NA	NA	422910
NA	NA	0	0	0	NA	NA	NA	1458300
0	0	NA	NA	NA	54100000	30600000	60700000	NA
NA	NA	0	0	0	NA	NA	NA	0
NA	NA	2.10E+07	4813500	10400000	NA	NA	NA	3044300
193410	0	NA	NA	NA	0	1181900	1243400	NA
NA	NA	0	0	0	NA	NA	NA	0
0	0	NA	NA	NA	0	0	0	NA
0	2260700	NA	NA	NA	0	1320500	1707500	NA
NA	NA	763350	0	1344000	NA	NA	NA	0
NA	NA	0	0	0	NA	NA	NA	427390
4186400	5084300	NA	NA	NA	1.01E+08	68100000	1.02E+08	NA
0	0	NA	NA	NA	0	0	3609800	NA
0	0	NA	NA	NA	1.10E+07	0	0	NA
NA	NA	2961600	0	2345800	NA	NA	NA	1572900
1.64E+09	1.12E+09	NA	NA	NA	1.34E+09	7.44E+08	9.17E+08	NA
NA	NA	0	0	0	NA	NA	NA	0
0	0	NA	NA	NA	0	0	0	NA
1.90E+07	40700000	NA	NA	NA	2.10E+08	1.57E+08	2.77E+08	NA
NA	NA	0	1019800	2211500	NA	NA	NA	6252900
0	0	0	0	0	42200000	22200000	23700000	8621700
NA	NA	0	0	1210900	NA	NA	NA	8180800
1.60E+08	1.33E+08	NA	NA	NA	1.12E+08	76900000	78900000	NA
0	0	NA	NA	NA	5237900	2501600	11800000	NA
5236200	0	NA	NA	NA	4223200	2313300	3629900	NA
NA	NA	1157800	740550	1326700	NA	NA	NA	0
0	375980	NA	NA	NA	7583300	6482200	6322100	NA
0	3187400	NA	NA	NA	2094500	0	4717900	NA
34800000	1.02E+08	NA	NA	NA	7.41E+08	5.33E+08	6.83E+08	NA
NA	NA	1940000	2373300	1950900	NA	NA	NA	13200000
NA	NA	0	0	305170	NA	NA	NA	362790
NA	NA	0	0	0	NA	NA	NA	1630400

0	1200000	NA	NA	NA	1401400	1199800	7218700	NA
1630000	0	NA	NA	NA	7700700	7141400	6253100	NA
22300000	23200000	NA	NA	NA	38700000	28200000	4.60E+07	NA
0	799420	NA	NA	NA	9518500	2585700	16100000	NA
NA	NA	7913800	1395100	16200000	NA	NA	NA	0

FFPE_MAX F4.2	FFPE_MAX F8.2	FFPE_TX.M F3.1.M	FFPE_TX.M F5.1.M	FFPE_TX.M F7.1.M	FFPE_TX.M F3.2.M	FFPE_TX.M F5.2.M	FFPE_TX.M F7.2.M	FFPE_SDS.I F2.1.M
7.26E+08	1.47E+09	4.27E+09	3.04E+09	1.59E+09	1.91E+09	9.98E+08	1.37E+09	8.58E+09
1.78E+09	1.78E+09	4.27E+09	4.03E+09	5.18E+09	1.90E+09	1.32E+09	3.12E+09	6.16E+09
3.02E+09	3.50E+09	7.85E+09	6.87E+09	9.57E+09	3.64E+09	2.52E+09	5.89E+09	1.07E+10
1.03E+09	1.02E+09	2.12E+09	1.99E+09	3.00E+09	9.06E+08	6.48E+08	1.73E+09	3.07E+09
1.59E+09	1.50E+09	3.43E+09	3.10E+09	4.16E+09	1.55E+09	1.12E+09	2.59E+09	4.12E+09
3.85E+08	1.07E+09	1.47E+09	3.92E+09	2.65E+09	6.61E+08	1.28E+09	1.35E+09	1.10E+09
8.76E+08	1.82E+09	6.23E+09	5.87E+09	3.20E+09	2.40E+09	1.73E+09	2.32E+09	5.68E+09
4.24E+09	4.48E+09	1.06E+10	8.96E+09	1.23E+10	4.78E+09	3.00E+09	8.24E+09	1.25E+10
1.32E+09	1.33E+09	2.48E+09	2.41E+09	3.33E+09	1.30E+09	8.96E+08	2.13E+09	3.51E+09
5.61E+08	7.11E+08	1.18E+09	1.25E+09	1.80E+09	6.85E+08	4.65E+08	1.19E+09	1.75E+09
1.53E+09	1.55E+09	2.93E+09	2.87E+09	3.72E+09	1.58E+09	1.13E+09	2.62E+09	4.09E+09
9.90E+08	1.84E+09	5.36E+09	2.92E+09	1.80E+09	3.18E+09	1.24E+09	1.24E+09	7.14E+09
1.05E+09	1.09E+09	2.22E+09	2.21E+09	3.17E+09	9.38E+08	8.34E+08	1.89E+09	3.00E+09
6.50E+08	7.64E+08	1.82E+09	1.69E+09	2.33E+09	7.29E+08	5.09E+08	1.16E+09	2.72E+09
4.89E+08	4.22E+08	1.21E+09	1.22E+09	1.86E+09	3.93E+08	3.40E+08	8.76E+08	1.67E+09
83200000	1.28E+08	2.84E+08	2.29E+08	3.64E+08	1.44E+08	82600000	2.00E+08	3.66E+08
1.61E+08	1.94E+08	4.50E+08	4.12E+08	6.49E+08	1.67E+08	1.56E+08	3.75E+08	5.99E+08
1.15E+09	1.20E+09	2.65E+09	2.50E+09	3.13E+09	1.08E+09	8.75E+08	2.01E+09	3.67E+09
2.23E+08	2.43E+08	4.97E+08	4.54E+08	6.47E+08	2.47E+08	1.77E+08	4.70E+08	5.83E+08
4.27E+08	4.17E+08	8.01E+08	8.76E+08	1.07E+09	3.57E+08	3.09E+08	6.97E+08	1.35E+09
3.79E+08	4.15E+08	1.14E+09	8.70E+08	1.60E+09	5.27E+08	3.54E+08	8.99E+08	1.58E+09
8.58E+08	8.93E+08	1.97E+09	1.44E+09	2.46E+09	8.04E+08	5.70E+08	1.41E+09	2.68E+09
2.53E+08	2.96E+08	5.59E+08	5.49E+08	8.62E+08	2.44E+08	2.07E+08	4.28E+08	8.62E+08
9.62E+08	8.55E+08	1.69E+09	1.83E+09	1.27E+09	6.93E+08	5.58E+08	7.31E+08	1.82E+09
4.08E+08	5.48E+08	1.50E+09	1.37E+09	2.14E+09	5.75E+08	5.37E+08	7.73E+08	1.18E+09
3.60E+08	4.15E+08	1.06E+09	1.01E+09	1.35E+09	5.00E+08	3.21E+08	7.81E+08	1.34E+09
1.61E+08	1.99E+08	3.70E+08	2.80E+08	4.96E+08	1.72E+08	1.11E+08	3.13E+08	5.77E+08
7.68E+08	1.04E+09	2.18E+09	1.73E+09	2.23E+09	8.68E+08	6.08E+08	1.49E+09	3.57E+09
4.01E+08	5.91E+08	1.23E+09	1.20E+09	1.47E+09	5.90E+08	3.92E+08	9.75E+08	1.63E+09
6.03E+08	6.10E+08	1.12E+09	9.67E+08	1.34E+09	3.91E+08	3.32E+08	8.71E+08	1.78E+09
1.15E+08	1.84E+08	3.48E+08	3.84E+08	3.51E+08	1.50E+08	1.25E+08	1.76E+08	4.69E+08
7.38E+08	6.32E+08	1.70E+09	1.74E+09	1.85E+09	7.51E+08	4.99E+08	1.07E+09	1.64E+09
4.34E+08	4.51E+08	1.01E+09	9.38E+08	1.22E+09	3.86E+08	2.94E+08	8.26E+08	1.36E+09
3.11E+08	3.43E+08	7.90E+08	4.09E+08	8.93E+08	3.32E+08	1.76E+08	5.22E+08	6.35E+08
4.35E+08	3.53E+08	7.69E+08	8.34E+08	8.87E+08	3.23E+08	3.07E+08	5.17E+08	9.61E+08
2.00E+08	1.64E+08	3.35E+08	3.91E+08	5.68E+08	1.54E+08	1.05E+08	2.90E+08	5.10E+08
90800000	1.37E+08	3.27E+08	3.00E+08	4.35E+08	1.10E+08	95800000	2.17E+08	5.17E+08
5.89E+08	5.02E+08	9.24E+08	9.40E+08	9.82E+08	4.05E+08	3.40E+08	6.01E+08	1.29E+09
2.65E+08	2.50E+08	3.52E+08	3.50E+08	6.10E+08	1.99E+08	1.46E+08	4.45E+08	7.71E+08
1.20E+08	1.01E+08	4.26E+08	3.62E+08	5.87E+08	1.27E+08	85700000	2.10E+08	6.63E+08
66800000	56600000	1.28E+08	1.69E+08	1.47E+08	51300000	35700000	1.35E+08	1.90E+08
92800000	79500000	1.58E+08	1.62E+08	1.95E+08	81200000	5.80E+07	1.24E+08	1.89E+08
1.90E+08	2.32E+08	4.74E+08	3.74E+08	5.32E+08	2.26E+08	1.86E+08	4.01E+08	5.58E+08
23300000	5218600	24800000	1.05E+08	50300000	25600000	61100000	32300000	8.10E+07
2.42E+08	2.46E+08	5.97E+08	5.48E+08	7.31E+08	3.01E+08	2.08E+08	4.85E+08	9.19E+08

0	0	43100000	1018100	1205000	873080	0	0	7551800
1.22E+08	1.45E+08	3.45E+08	3.23E+08	3.59E+08	1.56E+08	1.36E+08	2.67E+08	4.36E+08
1.81E+08	2.40E+08	4.74E+08	4.68E+08	6.85E+08	2.61E+08	1.76E+08	4.32E+08	7.87E+08
4.85E+08	5.11E+08	8.72E+08	9.71E+08	1.26E+09	4.72E+08	3.98E+08	8.57E+08	1.52E+09
96100000	74200000	1.46E+08	1.28E+08	1.35E+08	54700000	49600000	1.85E+08	2.46E+08
59700000	55500000	1.03E+08	1.18E+08	1.68E+08	70200000	49500000	82900000	1.79E+08
1.49E+08	1.29E+08	2.76E+08	2.73E+08	2.84E+08	1.42E+08	89900000	2.36E+08	4.59E+08
80300000	6.10E+07	1.64E+08	1.80E+08	2.14E+08	72900000	62300000	1.09E+08	1.97E+08
1.15E+08	1.04E+08	1.98E+08	1.32E+08	3.28E+08	99600000	77900000	1.93E+08	2.29E+08
1.34E+08	1.72E+08	2.19E+08	1.68E+08	3.80E+08	81100000	71100000	1.99E+08	3.89E+08
3737300	0	10700000	0	6468000	0	0	0	4.70E+07
1.11E+08	1.25E+08	3.16E+08	2.52E+08	4.00E+08	1.25E+08	73700000	2.58E+08	4.02E+08
1.90E+08	2.11E+08	4.77E+08	4.19E+08	5.80E+08	1.77E+08	1.49E+08	3.54E+08	5.93E+08
2.28E+08	1.53E+08	2.75E+08	2.40E+08	4.16E+08	1.84E+08	1.22E+08	3.52E+08	3.88E+08
6.69E+08	7.63E+08	1.62E+09	1.31E+09	1.91E+09	9.40E+08	4.58E+08	1.56E+09	1.54E+09
95400000	96400000	1.98E+08	1.90E+08	2.56E+08	93800000	7.00E+07	1.48E+08	2.75E+08
34600000	37700000	82200000	88200000	1.18E+08	53500000	21800000	71400000	1.22E+08
61500000	64100000	1.51E+08	1.35E+08	1.66E+08	67100000	46100000	1.29E+08	2.00E+08
34900000	26700000	69800000	52800000	89700000	28800000	2.00E+07	6.60E+07	68500000
73900000	47500000	1.12E+08	93400000	1.63E+08	67800000	4.30E+07	1.09E+08	1.57E+08
1.05E+08	1.41E+08	1.94E+08	2.45E+08	4.08E+08	94400000	53600000	2.82E+08	2.44E+08
67300000	6.40E+07	1.55E+08	1.45E+08	1.93E+08	64200000	50100000	1.21E+08	2.25E+08
1.30E+08	1.49E+08	2.82E+08	2.49E+08	4.29E+08	1.31E+08	85700000	2.32E+08	3.64E+08
1.28E+08	1.63E+08	2.08E+08	1.94E+08	2.55E+08	91300000	67400000	1.85E+08	3.88E+08
57500000	45500000	1.24E+08	1.24E+08	2.06E+08	49600000	50800000	1.67E+08	2.92E+08
0	7085400	2190500	17900000	0	1118500	4106300	0	61300000
9.00E+08	9.80E+08	1.76E+09	1.66E+09	2.35E+09	9.21E+08	6.17E+08	1.80E+09	2.12E+09
97700000	70100000	1.36E+08	1.61E+08	2.87E+08	81500000	80500000	1.74E+08	2.88E+08
1.25E+08	1.31E+08	3.16E+08	2.86E+08	4.45E+08	1.52E+08	1.17E+08	2.92E+08	4.90E+08
1.11E+08	9.10E+07	2.64E+08	2.29E+08	3.32E+08	1.20E+08	81800000	1.99E+08	3.73E+08
0	0	0	0	203830	6529200	0	0	0
23900000	2.30E+07	30900000	43700000	7.80E+07	15900000	12400000	50600000	67700000
2.72E+08	4.10E+08	6.71E+08	8.58E+08	6.86E+08	6.87E+08	1.91E+08	1.20E+09	5.81E+08
74400000	1.39E+08	3.12E+08	2.56E+08	4.51E+08	1.18E+08	81900000	2.46E+08	3.74E+08
24300000	29200000	38200000	54900000	80600000	20200000	29500000	4.30E+07	9.90E+07
1.04E+08	1.18E+08	2.05E+08	1.98E+08	2.78E+08	89100000	82600000	2.02E+08	3.03E+08
7.40E+07	51500000	1.05E+08	1.11E+08	1.35E+08	43400000	42300000	98200000	1.26E+08
6098400	9877000	367710	8552100	33200000	275290	882730	14400000	10100000
1.16E+08	84600000	1.61E+08	1.75E+08	2.52E+08	87100000	45700000	1.59E+08	3.50E+08
30400000	26100000	46300000	57100000	75800000	3.10E+07	16800000	3.30E+07	6.70E+07
24600000	2.40E+07	5.60E+07	52100000	98300000	23400000	15600000	44300000	78500000
20700000	27100000	44800000	38700000	5.10E+07	17100000	19500000	62700000	55200000
1.02E+08	63300000	2.06E+08	1.59E+08	2.83E+08	92800000	42300000	2.16E+08	2.99E+08
31500000	45900000	85600000	40100000	7.10E+07	38300000	22600000	39900000	9.70E+07
1.08E+08	1.23E+08	2.51E+08	1.10E+08	2.67E+08	1.16E+08	84800000	1.89E+08	2.60E+08
9788600	18700000	29400000	27200000	35200000	14700000	21100000	14900000	56900000
4980900	5123700	26300000	14800000	49400000	4665500	4945600	16200000	48500000

24900000	25800000	83700000	1.17E+08	1.56E+08	28100000	33100000	90500000	1.54E+08
14200000	17500000	2.60E+07	20600000	45300000	14900000	14700000	36700000	46100000
59200000	49200000	1.37E+08	1.09E+08	1.73E+08	6.30E+07	30100000	89800000	1.97E+08
14800000	18500000	43500000	39700000	45500000	22100000	15400000	27700000	59500000
16500000	11800000	36600000	31400000	40400000	11600000	13600000	9148000	42300000
58800000	62900000	92100000	93900000	1.92E+08	5.00E+07	36100000	89700000	1.51E+08
16700000	20500000	45500000	16100000	60500000	30200000	14400000	50100000	5.80E+07
1.17E+08	2.04E+08	3.12E+08	2.22E+08	5.58E+08	1.76E+08	1.77E+08	4.17E+08	5.11E+08
18900000	19400000	74800000	23100000	64600000	29500000	27600000	27700000	44300000
0	0	0	3222300	14600000	0	7961500	6092400	11100000
2.82E+08	2.56E+08	4.40E+08	5.31E+08	2.40E+08	1.16E+08	2.17E+08	4.80E+08	4.97E+08
50600000	46400000	87300000	57200000	1.24E+08	35700000	30500000	4.00E+07	1.14E+08
1.12E+08	91200000	2.29E+08	2.16E+08	2.37E+08	90100000	78700000	1.89E+08	2.94E+08
22100000	1.30E+07	36400000	23900000	58100000	10500000	12300000	36300000	3.30E+07
1.16E+08	1.24E+08	1.84E+08	1.93E+08	2.62E+08	1.15E+08	76300000	1.67E+08	3.56E+08
1.14E+08	1.19E+08	2.96E+08	2.40E+08	3.23E+08	80200000	78400000	1.56E+08	4.13E+08
65900000	74700000	1.23E+08	95300000	1.00E+08	40100000	35200000	62300000	1.81E+08
1.14E+08	83900000	1.42E+08	1.06E+08	2.13E+08	70900000	42100000	1.36E+08	1.92E+08
6.30E+07	63500000	1.34E+08	1.05E+08	1.97E+08	55800000	37200000	1.21E+08	1.41E+08
88300000	86900000	2.39E+08	2.48E+08	3.09E+08	98400000	68400000	1.66E+08	3.39E+08
0	2911100	2.20E+07	5355200	0	9723400	1201900	2485700	22500000
0	1355500	0	0	0	0	0	0	2.54E+08
12800000	2.40E+07	5.20E+07	52900000	89900000	18700000	16500000	31100000	69300000
47300000	58500000	65300000	75400000	1.43E+08	41400000	22100000	69300000	1.44E+08
41400000	38500000	45200000	48500000	67100000	21700000	26300000	4.50E+07	95600000
18300000	22500000	33200000	25500000	39300000	9580800	2349900	39400000	40500000
91200000	96400000	2.11E+08	1.86E+08	2.81E+08	86900000	6.10E+07	1.99E+08	3.05E+08
53900000	69500000	1.76E+08	1.78E+08	2.29E+08	69600000	42800000	1.63E+08	3.17E+08
35300000	25700000	61900000	79200000	1.11E+08	45400000	24100000	67300000	1.11E+08
12200000	1.10E+07	55800000	31500000	60900000	10500000	11700000	57800000	76500000
65300000	59500000	1.35E+08	9.00E+07	1.60E+08	62200000	45400000	1.13E+08	1.68E+08
4.20E+07	7.90E+07	58400000	1.23E+08	2.02E+08	47600000	5.70E+07	60500000	1.72E+08
1.40E+07	13400000	15600000	9742600	4.30E+07	11800000	7843300	6881000	4.70E+07
16500000	29500000	44300000	21600000	36300000	14600000	13900000	36100000	54800000
3.50E+07	24400000	54100000	72300000	56600000	19700000	12400000	47400000	72100000
11300000	6005000	13500000	14100000	13800000	3915700	3881600	16200000	11800000
27100000	20700000	51500000	46200000	68600000	32800000	19700000	31300000	92100000
27600000	31800000	50600000	45900000	52800000	27400000	18400000	51900000	68900000
9686400	11900000	76200000	52200000	75400000	14400000	11400000	30700000	81600000
1.72E+08	1.11E+08	1.74E+08	1.45E+08	2.48E+08	1.47E+08	42100000	1.72E+08	2.75E+08
26700000	19400000	1.05E+08	63800000	1.06E+08	9872000	29800000	2.50E+07	1.31E+08
7395200	14600000	13500000	17600000	16800000	5245400	9549200	1478300	40600000
0	519020	2489800	2561900	0	49300000	0	0	0
4.30E+07	56400000	58500000	94800000	1.15E+08	54400000	31500000	8.90E+07	1.53E+08
7044600	7398300	27700000	23800000	51700000	10700000	11600000	17200000	54700000
15100000	11200000	27200000	23700000	53900000	9771000	8433000	19800000	40800000
2848900	5898800	10700000	0	28500000	0	1572400	0	25200000

781790	0	0	2622400	8907000	0	0	4457400	9842800
8445400	8573200	9936500	18600000	7272900	4628700	8378100	16100000	35100000
14200000	12700000	23300000	23600000	35500000	3487400	6642200	19100000	34700000
33400000	17800000	29300000	36400000	67400000	33800000	15300000	29700000	52100000
1.31E+08	72300000	1.36E+08	1.50E+08	1.98E+08	76900000	56200000	1.30E+08	1.95E+08
7027400	13900000	19800000	29200000	28400000	7384200	9035100	19700000	2.40E+07
11200000	15100000	14700000	16500000	29500000	9714200	9222500	20800000	30800000
26900000	23300000	82200000	7.40E+07	57700000	2.40E+07	19600000	45700000	1.32E+08
0	0	0	0	0	0	0	0	0
17900000	9225700	15700000	0	70900000	19900000	17800000	17700000	17700000
1.06E+08	1.39E+08	2.49E+08	2.37E+08	3.50E+08	1.04E+08	63400000	2.70E+08	4.67E+08
21800000	18200000	27600000	54700000	73700000	21100000	2.30E+07	38900000	43200000
5612600	2832000	6795100	7425000	20600000	6866600	5335100	8062900	20400000
70800000	88300000	1.07E+08	1.07E+08	3.25E+08	63600000	57300000	1.50E+08	1.80E+08
2.00E+07	2.30E+07	32400000	40500000	45900000	26900000	17700000	42800000	51500000
19800000	15800000	38500000	41500000	52400000	12700000	11500000	40900000	40200000
15500000	10800000	18700000	6484100	33800000	3695400	9023900	13700000	28200000
0	0	3667800	4096200	8144600	0	1533400	4157700	4582500
23500000	2.10E+07	69300000	6.50E+07	1.03E+08	25800000	18200000	39800000	83400000
13400000	19400000	33900000	21100000	44600000	8446900	4794600	2.00E+07	44100000
13400000	11800000	25100000	33900000	2.70E+07	15100000	10600000	30600000	51500000
77300000	81300000	1.63E+08	1.13E+08	2.39E+08	78300000	52500000	1.60E+08	1.68E+08
0	0	0	0	95800000	0	39400000	0	0
1.10E+07	7077800	4445900	6283000	9437500	5984600	2261700	13800000	9344800
8129500	0	0	0	0	0	0	0	6361700
0	0	2491300	0	0	1106200	0	0	2512500
20600000	16500000	50500000	56300000	99400000	23900000	12800000	29800000	75800000
5973700	8168200	30300000	26100000	28100000	11300000	6852900	17700000	52200000
12800000	18600000	35500000	41300000	49500000	19200000	14700000	3.50E+07	23600000
0	0	0	0	0	0	0	0	0
32400000	25800000	65900000	65200000	68500000	25300000	18200000	3.80E+07	9.30E+07
28200000	29900000	71800000	40100000	35600000	26800000	24100000	63300000	1.01E+08
15600000	21600000	45200000	39600000	40400000	14100000	11200000	23200000	40900000
43200000	26800000	53800000	53500000	69500000	19400000	21400000	40200000	77800000
34100000	36200000	38600000	17900000	35800000	14700000	8490300	3.60E+07	45900000
16200000	18900000	22100000	22400000	22500000	11700000	7127600	21800000	36700000
3561700	11100000	34900000	27400000	32500000	10700000	11800000	7901600	29300000
4115000	6704200	12200000	16100000	22100000	4672400	5799000	1.30E+07	14500000
7415900	17800000	26600000	23300000	25200000	11900000	7779300	24900000	24200000
12400000	14100000	41600000	24700000	36200000	16500000	8929000	16400000	42700000
16100000	15600000	2.50E+07	63400000	81100000	16600000	32100000	29200000	75700000
12500000	11800000	21900000	1.60E+07	30200000	1.20E+07	7451800	34300000	29300000
4458200	5123400	22600000	11300000	24300000	0	5268000	1.30E+07	32100000
1.79E+08	2.17E+08	1.62E+08	2.50E+08	1.38E+08	7.40E+07	91300000	1.85E+08	1.23E+08
11800000	19200000	32400000	27700000	8562500	11300000	7609100	1.30E+07	55700000
6142900	6662900	26200000	23800000	55600000	6705300	7310300	14800000	4.10E+07
0	2144200	0	0	0	0	0	5846200	0

18900000	1.70E+07	21900000	17800000	45800000	6749500	7201600	2.70E+07	28500000
2.60E+07	35600000	55300000	48900000	76400000	23500000	21600000	49900000	1.01E+08
5814100	22600000	50800000	44700000	81300000	23300000	11200000	31600000	78600000
0	5674800	16100000	21300000	0	3721800	6103800	0	25500000
24500000	15200000	54100000	47500000	86900000	17800000	18800000	27600000	1.05E+08
12800000	7254300	23700000	26100000	27400000	9324600	6920000	26300000	31300000
866340	0	8012900	7216400	13700000	879370	518560	5459900	10800000
55900000	49900000	96300000	1.22E+08	2.20E+08	54800000	26600000	1.24E+08	1.98E+08
19900000	2.60E+07	55700000	25700000	2.60E+07	20700000	14500000	30400000	86200000
1384400	7686800	24400000	8200800	20500000	4020300	3306300	19300000	26900000
8923600	7258000	12900000	10900000	23400000	6536600	5502700	12900000	17900000
9357500	15300000	30200000	35100000	44400000	9286100	4022900	22500000	6.20E+07
0	0	0	0	0	0	0	0	0
54300000	45100000	94600000	1.23E+08	1.76E+08	6.70E+07	43700000	1.03E+08	1.64E+08
8976000	9839100	9476800	0	18700000	9957300	4498100	18300000	26500000
14300000	6284600	24600000	5246900	29900000	13600000	1.00E+07	8085400	31700000
13700000	5028800	34500000	2.40E+07	40500000	7522500	7903700	19100000	43900000
7806500	14900000	14200000	12400000	29600000	7167900	3255600	12100000	30900000
7138800	27200000	62500000	49700000	67300000	13600000	8715000	36600000	85800000
51600000	50900000	1.45E+08	1.03E+08	1.72E+08	49600000	33200000	97600000	1.75E+08
44800000	59400000	1.11E+08	1.47E+08	1.45E+08	70400000	35600000	1.25E+08	1.46E+08
60700000	33700000	1.27E+08	1.17E+08	1.77E+08	65600000	24200000	1.22E+08	1.93E+08
1.20E+08	2.68E+08	9.35E+08	7.20E+08	4.95E+08	4.40E+08	2.19E+08	3.81E+08	1.32E+09
16700000	20500000	68100000	93200000	99500000	20900000	16300000	52700000	1.23E+08
25500000	27500000	45700000	51800000	65200000	11600000	11800000	45200000	72800000
1205800	15500000	50700000	38200000	37100000	21200000	19700000	23200000	33300000
0	2095500	0	2379500	0	1540400	0	2142000	0
18600000	24300000	3880400	18800000	32200000	13200000	1.40E+07	37800000	21900000
13900000	8940500	25100000	23800000	47300000	6154100	4349900	13300000	35300000
0	13500000	1147600	0	0	0	0	46900000	213850
6825100	10800000	14500000	1.30E+07	24700000	4624700	5568400	14400000	26200000
26200000	22600000	30100000	24900000	57300000	2.60E+07	1.20E+07	44200000	47100000
0	0	0	0	0	18800000	0	0	0
0	0	11700000	2780200	0	0	1094500	2461000	0
6091200	8195300	28200000	29100000	32400000	2720900	6150600	14500000	96900000
16100000	26100000	42600000	52300000	57800000	20400000	10500000	48100000	53200000
15700000	17600000	33300000	28600000	4.00E+07	14500000	14100000	23800000	51800000
14500000	11800000	21200000	20800000	52700000	8977200	8646300	29300000	40300000
3337000	2251900	2123100	6926600	16200000	2436700	3251900	6006100	5962100
15900000	10600000	15700000	27600000	3.00E+07	12200000	12100000	16200000	19100000
4.50E+07	14400000	80900000	78900000	1.31E+08	39200000	1859400	83200000	1.24E+08
14400000	8874000	24800000	17600000	22700000	5305700	8333700	14300000	51800000
33500000	36800000	56100000	41100000	76700000	29900000	16100000	37100000	7.50E+07
1.30E+07	14900000	23500000	26400000	2.90E+07	13100000	10900000	19100000	27900000
0	0	4885000	0	0	8981500	0	0	23700000
51700000	3.40E+07	9.80E+07	83100000	1.12E+08	41300000	38800000	74700000	70400000
7164100	11900000	27300000	39900000	3.30E+07	1.20E+07	8681500	25100000	4.80E+07

5845100	1957300	3017800	3110200	8352500	1975700	804400	6683700	4934300
15200000	9207200	2.20E+07	21200000	30300000	7888900	8157400	29500000	34300000
0	0	0	0	0	0	1437400	8871900	0
3829000	4609200	9534000	7074600	11700000	2738900	1501700	5299800	11700000
41200000	42400000	1.20E+07	15300000	2.10E+08	8116300	26900000	12900000	32800000
1579800	4962900	15200000	13900000	1.60E+07	7828800	4550900	5507600	20800000
1415300	0	0	0	0	0	0	0	0
0	0	0	0	0	0	0	0	0
2840700	1707600	0	0	0	1677800	1498100	0	4573000
1.94E+09	3.43E+09	6.42E+09	6.09E+09	8.11E+09	2.83E+09	2.31E+09	2.84E+09	8.33E+09
22800000	22300000	37700000	39200000	50500000	18800000	11400000	34700000	43700000
8500400	6447200	7555100	12600000	8885700	1088500	3958500	16100000	1.50E+07
2235800	1733800	4114000	1646000	4739500	1791500	1190600	1781500	48400000
13600000	17200000	10300000	21800000	10800000	11700000	7449700	18500000	2.90E+07
7337800	7931700	24100000	12300000	33900000	9672500	5019200	22900000	35600000
4483900	15400000	4640200	12700000	17600000	8431100	11700000	22200000	1.90E+07
625720	0	7825800	7653300	10200000	0	0	0	12700000
1825700	5784100	14300000	6335000	8991600	5878400	3805400	7746100	27100000
7868300	8485400	1.50E+07	21600000	18800000	9339500	7438400	14700000	20800000
6481200	8641500	11600000	11600000	16300000	5285800	2366000	12500000	20300000
7455800	3472600	1.30E+07	1.40E+07	2793700	10400000	4194500	13600000	15800000
3051000	4014000	6905400	7269300	10900000	2403200	1891700	6028200	9292400
5021200	11100000	0	15700000	19800000	6670000	851620	6131000	26900000
0	7112900	0	28800000	0	3251300	9221600	1089000	3.30E+07
7333600	2246200	4477700	5060300	16200000	1582500	1872100	12600000	17800000
38400000	32100000	1.42E+08	82200000	1.83E+08	56300000	44300000	81600000	2.19E+08
8467200	8195300	11600000	14700000	24500000	6857900	6735000	8459900	21800000
8693500	3369900	14900000	2.20E+07	27100000	6320900	0	1090800	23800000
6674800	9447600	24600000	16600000	1254900	9734400	6056500	15400000	3.10E+07
2733900	2128100	3721000	8084200	13400000	1631700	320450	4253700	4428700
0	0	0	0	0	0	0	2917300	0
17200000	3113200	0	4864500	0	0	858610	0	0
10900000	7601900	33100000	29500000	48500000	12400000	4376300	32900000	50600000
7618500	2486000	15900000	12700000	11100000	6249300	2374900	12500000	10800000
6346500	5557700	9382200	6964700	11900000	5501900	2253000	9546300	2.00E+07
583940	5568600	2599000	2336200	6371000	4360200	1036400	8130800	20200000
4566400	6068200	8469700	4303900	3731100	3033100	1480700	3633700	13600000
0	0	0	0	0	0	0	0	0
0	1826900	0	0	930860	2616100	0	5965200	0
9322600	7890700	10800000	1.10E+07	12900000	4619800	4346300	12400000	11700000
2201200	2983000	15900000	4506800	15600000	2528000	2413200	4920400	19200000
2803400	6831500	6506100	6166300	8350600	3104300	4126200	12500000	11100000
7776400	3859900	4574500	14500000	15200000	0	4481900	11600000	18600000
0	0	0	0	0	0	0	0	0
5366300	2771900	4536000	4442600	11200000	4972800	2096300	6966200	14100000
10900000	10600000	8531000	13400000	41900000	0	12900000	10100000	34400000
5521400	6095100	9589300	1.00E+07	12500000	5145200	2372300	10600000	16900000

8506000	18200000	14800000	1.30E+07	40500000	7553300	2727000	21500000	52100000
31400000	29300000	49200000	57500000	95900000	29100000	22200000	44100000	86500000
20300000	22900000	32900000	4.00E+07	5.40E+07	13500000	15200000	33800000	40500000
2113600	2212200	0	0	0	2586600	2224900	6960400	1595800
2088000	5584400	8625800	16500000	19700000	16600000	4801500	13300000	2.00E+07
0	0	0	0	0	0	0	0	0
9001300	6447600	20200000	14200000	18100000	8779800	3751200	17900000	2.70E+07
0	1166900	3024400	3568600	2.00E+07	0	1349400	0	0
8308500	3039200	10600000	16700000	17600000	3774400	4782900	5908300	11400000
6039800	8860800	11300000	12200000	20800000	3889900	0	7272800	1.20E+07
0	1575600	0	3580100	13900000	0	0	0	10500000
3615800	5579300	11200000	8447200	17300000	0	3953800	8476400	22800000
0	0	0	0	0	0	0	0	0
11100000	4694500	0	1247000	11100000	4816500	0	13600000	13700000
6458400	6127600	12800000	8516000	10600000	5418400	4643200	6021000	19400000
0	0	0	0	0	0	0	0	0
477070	587130	798110	1093600	811310	807000	722920	895530	710620
7498400	10200000	4025500	4393400	11700000	3575300	1819800	3935800	13200000
1555500	0	8950600	7035900	16100000	0	1124100	2681200	19300000
25300000	8455400	28300000	11800000	3.90E+07	16200000	11600000	13700000	3.00E+07
2695600	2016900	5557400	5573900	6733800	0	705190	4617300	18500000
4533900	9870800	17500000	18500000	37800000	9897500	5999100	13800000	19800000
19600000	16500000	29800000	28900000	3.30E+07	15200000	9120300	30100000	47400000
5206300	4141600	4266600	6105400	11700000	2556000	1578400	5423700	7910600
3311100	2879200	2671800	4161900	16100000	1747200	2016200	5439800	13400000
0	6440000	3661300	9836300	19900000	2063600	4088000	11800000	21400000
1698800	3222400	5915800	5064800	6914200	2764500	2921800	11100000	11400000
16100000	15100000	2043300	10300000	16900000	7990100	1.00E+07	27100000	0
0	0	26300000	12100000	34400000	6686600	2429600	4566900	24400000
9527300	6403500	3760100	3394100	3270200	1933100	874000	1434300	3441500
23300000	17200000	29700000	26500000	46100000	19700000	12100000	34900000	6.20E+07
12300000	13100000	32100000	39800000	50400000	1.80E+07	16100000	28100000	5.50E+07
4342700	3052500	9223900	2370500	25700000	1424800	7482900	3517700	12800000
5128600	6635800	0	0	5137100	3243700	2179100	8843500	1.60E+07
268970	1742300	0	0	599700	1348300	2440000	5410100	0
20800000	23400000	46300000	30600000	41500000	26200000	11300000	50500000	42200000
0	0	0	0	0	0	0	0	0
4174100	3679400	4862100	13100000	9260200	2912100	1741900	5757800	9410000
21800000	20900000	37500000	3.40E+07	51300000	18200000	1.20E+07	30500000	53500000
0	0	27100000	28200000	42300000	15300000	9864900	29700000	49700000
2874100	9171500	6798800	5897800	11300000	5695000	1258200	12200000	13200000
7497000	4708100	16700000	12400000	8521100	6429300	6440900	9913300	28700000
35600000	38400000	1.09E+08	92500000	1.66E+08	50200000	24700000	93900000	2.55E+08
6107700	20500000	16500000	19800000	35900000	10400000	6571600	1.70E+07	18600000
57400000	2197700	8471600	5474800	0	4838700	1941400	0	3879700
0	0	0	0	1979500	0	0	0	1852800
3564400	2802300	7807600	10900000	17100000	3291400	2065700	7041800	1.90E+07

11400000	663280	9082600	16700000	1637400	1427200	3697600	1010200	12400000
2653300	3714600	2208800	7794400	2983200	2251200	5888000	7269700	14200000
6930800	6519000	3560300	17200000	5553200	6337700	1776400	0	26100000
745720	0	1565900	0	7919300	2432200	0	2691100	5371800
3616700	3233600	8372700	7952500	11500000	537520	2432800	6888700	10300000
3433800	2497600	9699600	16200000	1.70E+07	5503300	3452500	4696500	20900000
2109000	13100000	1702200	22900000	1912400	13400000	622870	25800000	37700000
2982500	4518800	3131200	3199700	5094300	3410000	5074700	9011000	4730800
0	0	0	2398800	0	0	0	0	0
2988900	0	0	2604800	4081900	0	700010	0	3122000
3876800	1686300	0	0	0	838970	983060	1421300	5069100
0	0	0	0	0	0	0	0	0
0	5849800	9443000	0	14500000	4779500	3996600	0	14500000
4649700	8200600	2.10E+07	5738500	13900000	1.00E+07	2385800	8073600	30300000
7003100	1471800	3306000	3314300	13100000	1670000	3655200	5303000	7044300
8014800	11700000	23100000	22400000	36600000	1.10E+07	8479200	22100000	16800000
2914200	6529100	7528600	0	33700000	5691100	2650700	11300000	2.20E+07
4775800	2952700	6234500	13400000	11100000	4343600	3657100	5288800	10600000
2341300	4443000	7714200	17100000	6651000	9607200	6560200	15500000	19100000
0	0	0	0	0	0	0	0	7853700
0	2774400	0	0	0	0	0	0	0
10600000	6574600	8796300	0	6049900	3852900	3616900	9126000	0
1596900	2282300	3144700	0	8970600	1710400	2022900	2759800	2228600
0	1923300	1839700	0	2078900	2435800	444620	0	7733900
15700000	17400000	38600000	23900000	50400000	11300000	10700000	30500000	40100000
0	468500	0	155380	0	652400	0	0	0
1399300	860480	0	1393700	1111500	1505600	876140	1553700	1761200
0	0	0	0	0	0	0	0	0
2836600	2434300	4159100	4566300	5116500	1894100	1809300	4385600	6020600
0	0	0	0	0	0	0	0	0
0	0	0	0	0	0	0	5837000	0
2116600	1720300	2934800	2944300	51400000	1592700	884230	4974200	13500000
5207200	1193200	18700000	1.80E+07	30400000	6004700	3271800	10500000	21900000
6729700	5323800	0	0	12500000	0	869550	9367700	0
0	0	7054500	0	10300000	0	2519100	2046300	0
0	0	0	2326500	0	0	3437000	6424500	2846000
5330500	6993200	9609100	11300000	8919300	4533000	2663900	1.20E+07	13300000
17400000	8356600	21100000	2.80E+07	28800000	5379500	4400600	18100000	3.10E+07
3084600	3059300	8883400	1.50E+07	1.60E+07	3733900	2354300	7727400	13400000
1811400	1551100	5954500	6965200	9033000	0	0	2955900	8500400
1625600	2965500	12500000	21900000	14800000	5458600	3282700	7285200	24500000
3411300	5272000	0	1548700	2425400	0	866870	8054100	14600000
1116600	2827800	4028400	2156800	76405	1567700	0	4258700	7767400
0	0	0	0	0	0	1203300	0	0
0	2394700	5917000	16100000	9392600	2117900	1473900	0	1.80E+07
2590000	4016900	7263600	11300000	6511200	2281200	3500500	3730600	9660200
1356400	905700	0	0	1437000	1361300	954590	4390400	2914700

13200000	2588900	0	19700000	11100000	9790500	2194400	13200000	10700000
5366100	6055800	8010600	6994000	19700000	4264500	2789300	8105800	6282200
8636100	8773400	7954600	1.10E+07	12200000	8962700	3437400	17900000	20200000
2562400	2665700	3616800	3156800	4108600	1747200	1239000	3687900	4945600
4936700	11900000	29600000	30400000	39800000	3590800	6588700	2.50E+07	53200000
4628400	0	0	10300000	5448400	1534100	3531100	5425300	0
3228100	5323500	4910700	12200000	12200000	4521500	4209800	6222400	12400000
610580	1683300	9199900	0	10600000	0	3124100	9926400	8539800
0	0	4380600	0	0	0	0	0	0
392010	441920	1052900	975190	1917100	512650	1059000	1152600	599280
0	0	0	0	0	0	0	0	0
10100000	8273900	1.10E+07	12100000	9281000	2930800	3031300	4813600	12300000
10800000	9289400	20300000	18200000	23800000	7412600	7245600	16400000	2.10E+07
1481300	601380	3966000	0	0	1414300	0	0	0
0	3574400	0	4731600	8543400	693300	0	7156200	15300000
1889300	0	0	0	0	0	0	0	0
6086000	14100000	9509000	4696900	12300000	7694500	3780000	18100000	2.10E+07
0	1455100	0	3813600	0	0	3262700	0	3579400
5561800	5163500	27300000	21600000	36600000	1946100	0	11500000	33100000
0	0	0	0	0	0	0	0	0
8979300	0	13100000	9933600	13600000	2227500	5105300	7710000	26400000
0	0	0	0	3097200	0	0	0	0
3143000	708420	238750	2827000	414990	917320	1956100	2335600	3964500
0	0	0	0	0	0	0	0	0
5598800	4404700	8477100	8411500	0	1847200	1906700	0	12100000
3622000	2715900	3875600	4644100	6548200	2451300	2056000	6357100	7468000
1393600	2623300	6955200	5694700	8014400	3351900	4437400	4962400	13800000
2331400	1838900	3712900	7672400	4603200	6608000	3171100	4833500	7009100
1420200	1450000	2900800	2938300	2876000	1273200	1082100	2109200	4284200
0	5598300	8900400	4561900	5604200	0	1884200	0	8403900
4515400	9257900	6124500	5856400	13600000	3770600	2763000	6949000	21700000
1209600	349940	1149100	0	6073200	605830	0	0	1019000
0	767310	2492000	2324000	0	0	0	1710500	0
0	0	0	0	0	0	0	0	0
0	0	0	7749800	6123200	0	0	0	9649300
0	0	0	0	0	0	0	0	0
561590	2455900	2649400	0	4704600	1115000	1082400	3694800	4511000
0	0	0	0	0	0	0	0	0
0	0	0	4960200	0	758700	739860	0	4288600
0	0	0	0	0	0	1069400	0	0
0	0	0	0	0	0	0	0	0
4335500	6146800	7174800	7570300	9270500	6120500	4192000	11400000	13300000
2409100	2036800	3180900	3199300	5925200	3319100	0	3771500	14500000
5266600	4437600	8289100	8748200	11500000	2815900	1833100	8441800	12600000
0	0	0	0	0	0	0	0	0
1341200	1869800	2976000	2501200	4244900	3293200	1494800	3336000	5317600
1155900	0	10900000	10600000	1.80E+07	5171200	3162100	10600000	10600000

5875600	4949500	2939000	9027500	14300000	2087900	3055200	16800000	14600000
827540	854790	1275800	1408800	1610000	881240	596730	1116700	1602700
998360	1085600	1464600	1879300	1729800	669860	572080	1637800	1804100
0	3222600	9012600	0	9984300	0	1967400	0	10700000
2078200	1304500	1.00E+07	7115600	2902000	0	977700	2043500	8195000
1768100	4318700	9163300	4393000	4489200	3755800	3082100	6741300	13900000
8398200	8893700	13200000	0	22700000	4374500	3169600	17300000	9270800
3727700	4700100	0	6695700	11800000	2414600	3049900	5814700	12300000
0	0	0	0	0	0	0	0	0
718590	3776400	1079500	719900	1181600	3263400	1476500	6518400	1462600
1309200	1621500	5616800	3088000	8524600	900830	888600	4317300	3233000
0	0	5680900	0	4202900	0	0	0	10900000
0	523960	4649500	0	0	836550	0	0	0
0	0	0	0	0	0	0	0	0
6365500	6018000	19100000	21200000	0	6818300	0	10900000	0
0	6814800	0	0	0	0	0	0	0
1531800	0	2424000	3301100	3795000	997280	1087000	9419300	9373500
0	5658400	18200000	4473800	1.90E+07	0	0	5817500	15100000
0	1722900	5037500	0	0	0	401410	0	0
0	0	0	0	0	0	0	0	0
10100000	7004600	19100000	15300000	14400000	10200000	5640800	18800000	23400000
0	0	0	0	0	0	0	0	0
1244500	0	0	0	0	1945500	0	0	3305800
1271000	5022700	0	0	0	1772200	3231500	1607500	0
0	0	0	0	0	0	0	0	0
1722600	2387400	4877900	3506600	6573600	3331200	1157500	4859100	7523100
10900000	8775900	0	4821300	5862900	9172100	1664700	7238300	1.20E+07
0	3.00E+07	10100000	58800000	11200000	21200000	3473200	43100000	10400000
2352800	0	0	5352300	0	0	0	717920	0
0	4245000	8904100	6731700	19100000	1406100	2381600	0	18700000
301840	1158600	0	746380	516620	2643800	213360	554360	561250
743190	4479200	4341200	6477600	7174400	2336600	0	5778900	9351500
11700000	1.10E+07	6467500	1608300	6273800	0	1462900	8008200	5734400
0	0	0	0	0	0	0	0	0
0	0	3927100	5764800	4849200	1657600	1578700	2650300	10200000
12600000	1488600	0	548390	0	0	4172500	0	760100
34900000	2544500	64300000	65600000	96700000	32200000	5582100	68700000	1.00E+08
0	0	0	0	0	0	0	0	0
0	0	0	0	0	0	0	0	0
20400000	12400000	26200000	17100000	28900000	10100000	4459600	21300000	32400000
0	0	0	0	0	0	0	0	0
2547500	0	0	5291100	8479900	0	2806300	4337700	8685400
11900000	22200000	15800000	1.90E+07	23200000	0	3489900	0	29200000
1615300	5238400	5189500	4881300	8167500	8574900	1891000	0	7119000
4186100	5369600	3118300	8240900	2597900	3800700	5053600	1.00E+07	16600000
2055200	2908700	4163400	0	3921000	2313900	1634900	2872400	5722800
1098400	1078500	5347300	0	2748500	0	1707200	1793300	3224900

0	0	0	0	0	0	0	0	0
0	0	0	0	0	0	0	0	0
1459700	3486700	4085800	8648400	16100000	0	0	7187300	14900000
0	0	0	648280	373570	0	301080	804730	464650
1968700	2926800	0	0	5774600	1219200	2156100	9359200	0
1458100	0	5297700	12500000	6025800	1779500	11100000	3794600	6878900
982330	2628000	7607600	11200000	1018400	5099300	2957200	6319600	852200
734990	7302500	0	0	2106700	0	0	0	2905000
0	0	0	0	17600000	0	0	0	0
3490500	1152000	3406400	0	0	3425600	0	0	3660900
1400700	12200000	7975200	0	9386400	1838200	0	9314400	8361900
0	0	3916000	0	0	0	0	0	0
0	0	0	0	0	0	0	0	0
0	0	0	0	0	0	0	0	0
0	0	0	0	0	0	0	0	0
1227200	0	0	2131600	0	0	0	0	0
0	0	3282900	2791000	0	0	0	0	4674800
4467300	6687300	24800000	6464200	14900000	1818100	4522100	4044900	1.40E+07
0	2375700	0	0	0	0	0	0	0
6297300	4244800	0	16800000	0	1589700	2442200	0	0
0	0	0	0	0	0	0	0	0
0	2914900	0	7944000	1958800	4427800	4473500	6782800	3383500
0	0	0	4918800	4434700	0	0	0	9339700
0	4174300	12300000	15600000	12600000	3857600	3204700	6982100	18500000
5521700	3850200	16100000	18300000	16600000	3564200	3864000	12800000	13800000
0	0	0	0	0	0	0	0	0
11800000	15100000	5642200	24600000	4009900	13300000	9630900	20100000	2337200
478840	1224000	2464700	2186000	4360500	0	493050	795730	5582000
20100000	7532400	18800000	14700000	46300000	0	8727900	10400000	12200000
13200000	12100000	29500000	25800000	34300000	15900000	6263600	29600000	15700000
0	0	0	0	0	0	0	0	0
0	0	6674100	0	8517600	502700	0	3250600	0
4784400	4872900	0	0	1.80E+07	0	0	2489200	19400000
5708000	6053200	6000400	5304200	1.30E+07	0	2007500	7738000	12100000
0	0	3716200	2313000	2398300	6402100	0	0	0
0	0	0	8067000	0	0	2220000	0	11200000
5807200	6452500	590750	871420	674700	7507800	280970	8044800	5525400
0	0	0	927740	0	0	0	1530300	0
0	0	0	0	0	0	0	0	0
1694800	1683300	14700000	0	0	0	0	1649800	2568900
3227300	0	4797900	4591700	647810	0	1227200	4060600	5242900
4630500	3368300	3413300	0	10700000	0	0	13800000	13400000
0	0	0	1326600	0	0	0	0	13200000
10400000	1.20E+07	22400000	21100000	36700000	12500000	8742200	18900000	14500000
2159500	0	3912800	4822400	6833800	2573600	0	4713000	8299400
0	0	0	0	0	0	0	0	0
2998900	1421300	0	973120	4427800	1526500	1148600	3293000	5040200

1610600	0	0	0	0	1322200	0	0	4503600
5268800	2447400	0	4968100	0	1830000	1558100	3945700	0
6143900	2517500	0	0	0	0	1451500	0	0
0	0	0	0	0	0	0	0	0
0	4370200	8705600	8169700	1.50E+07	4135700	2852000	10900000	12800000
2581200	3154500	1268300	1898300	1724100	1432600	691730	2066400	2543000
0	8681000	21200000	19300000	26700000	1681100	6373700	1.40E+07	28200000
1383100	8585900	6069800	3167200	3647600	0	1622700	0	10100000
0	0	0	0	0	0	0	0	0
0	0	0	0	0	0	0	0	0
2045400	0	6334900	0	0	1656200	1009100	0	0
0	0	0	0	0	0	0	0	0
3624500	4745900	0	3987700	7138600	0	1040400	4441900	7234200
0	0	0	0	0	0	0	0	0
1643600	721100	0	0	0	0	0	2947300	0
1040200	1142300	0	1859100	7803600	0	782400	1591100	0
0	0	0	0	0	0	0	0	0
0	0	0	0	3975700	0	0	0	2302100
1782700	3014300	0	3000100	0	1996300	2393200	7147900	4354400
0	0	2288500	1663900	2343700	0	0	1118000	1892600
4960800	0	7308500	5558300	0	0	3658700	7621900	2.50E+07
0	0	0	0	0	0	0	0	0
2325900	6084200	1194800	0	6766100	0	1819800	2117100	11600000
527420	803670	0	0	2242100	0	0	0	0
0	0	0	0	0	0	0	0	0
2064200	0	0	0	4225400	0	1173700	2897800	0
258090	0	0	0	568280	285380	0	0	281940
1018200	0	0	0	2368000	0	496190	0	2621100
4007000	4502100	4394800	5241100	0	4308500	2136800	5488400	2627500
3920000	2835100	4066100	0	0	2711000	0	8742400	0
1667500	1454500	1716000	1685800	1849300	3869700	2584500	6126100	2371700
0	0	0	0	0	0	0	0	3847900
0	0	0	0	5150500	0	4405500	0	0
5210700	4174200	22200000	21100000	27800000	9865800	7612700	17900000	3170900
0	0	0	0	0	0	0	3773900	0
0	0	0	0	0	0	0	0	0
1778600	1138600	0	2771900	0	1890700	1086000	3252700	0
393030	0	0	0	625150	0	244210	0	502090
2647500	3962400	0	0	14700000	3808000	2908400	5923500	1.20E+07
2475800	1394600	0	0	2316500	1798400	0	2170300	2540200
0	0	0	0	0	0	0	0	0
0	5050200	12400000	12600000	20100000	7314500	6146900	0	2.10E+07
0	3030000	0	6855500	5401300	0	0	4574600	5379200
0	0	0	0	0	0	0	0	0
6295400	6885100	10700000	9979600	12300000	5365800	4982700	7417300	16600000
1005000	786000	2229200	2095700	2380900	1489900	559030	1812800	2193800
0	0	0	0	0	0	0	0	0

1390800	0	0	0	0	2017900	0	0	0
0	0	0	0	0	0	0	0	0
0	0	0	0	0	0	0	0	0
0	288630	0	0	2148100	1013300	0	1730100	0
0	0	0	0	0	0	0	0	3964400
6442500	0	3930400	0	4509800	0	0	0	6976600
1.30E+07	6550600	4624200	10900000	24100000	6799500	4122100	9377800	16300000
0	0	0	1378100	1613800	0	0	0	0
403000	0	0	0	0	0	0	0	367650
5095700	3447000	0	274140	3263700	2062400	0	2850400	5482700
2023100	1659200	0	0	0	1036700	0	2128400	0
2087700	737650	786160	1499200	0	4844100	2238500	4764500	0
0	5710900	8500800	0	633180	3737800	3491400	10200000	0
0	0	0	0	34500000	0	0	0	0
0	0	0	0	3558200	0	0	4019800	972750
737330	1247800	4442600	6303300	6905400	0	2122100	913670	9222100
326700	1101400	1140400	3330200	0	0	468550	0	0
0	0	0	0	0	0	0	0	0
0	0	0	0	0	0	539100	916370	0
0	0	0	0	0	2708300	2020700	0	897540
0	0	0	0	0	0	0	0	0
0	0	3536300	0	0	0	0	0	0
0	374210	1461400	800670	761670	1105400	276050	1148200	1008300
4268400	2058200	0	0	0	2619300	1160200	3572000	0
7293800	9369000	17600000	15100000	19700000	9973700	6815100	16300000	26700000
9557600	6259200	19800000	21200000	27900000	7759400	0	32200000	0
3028300	2014900	0	0	9130100	1397400	0	4134700	11900000
0	0	0	393980	5220300	0	0	175580	0
0	0	0	0	0	0	0	0	1149800
4421500	1921000	2970400	0	6868600	0	0	3579700	4829900
1599400	6394100	8345600	0	0	4154700	2702500	10900000	12200000
0	0	0	0	0	0	0	0	0
0	0	0	1892700	2530800	0	0	0	0
0	0	0	0	0	0	0	0	0
3329900	1437000	0	4318600	0	0	0	0	20100000
5661700	2388300	6064200	5858100	9857600	2902800	2312100	1.60E+07	14100000
1348700	0	0	7558100	7669900	1451400	1144300	206250	2449200
3222500	3575600	7839900	8899300	11700000	3658000	2997600	5837300	19600000
0	0	53800000	0	0	0	0	0	81700000
2550800	1747600	3164900	1611700	3113500	1091700	878060	2063000	4004700
0	3326500	0	0	0	1090600	0	4373100	0
1951100	2167300	0	2789100	11400000	0	6149000	4911100	4199300
0	0	0	0	0	0	0	0	0
0	0	0	0	0	0	0	0	0
0	0	0	0	0	0	0	0	0
0	0	3730400	0	0	0	0	0	14200000
0	0	5019700	0	0	7184600	0	0	0

0	0	0	0	0	0	0	0	0
0	0	0	0	0	0	0	0	0
0	0	0	0	0	0	0	0	0
0	0	0	0	0	0	0	0	0
0	2577500	8807400	17500000	32900000	3835500	1904100	23100000	13900000
0	0	0	0	0	0	0	0	0
2726200	4711500	0	0	3671700	890820	0	4345900	5561900
876310	1973400	1342400	1471000	0	1734800	1252700	1546800	1754800
0	0	0	0	0	0	0	0	0
398920	0	0	3043500	0	0	0	0	7225300
0	0	0	0	0	0	0	0	0
0	0	0	2815200	0	0	0	0	5319400
0	0	0	0	0	0	0	3506900	0
0	0	0	0	0	2959200	0	0	0
2294800	1891800	8150300	0	7316500	0	0	0	0
0	0	0	0	0	0	18900000	0	0
0	0	0	0	0	0	0	0	0
0	0	0	0	0	0	0	0	0
5134800	2633500	6218700	0	0	0	0	5500300	2059200
0	0	0	0	0	0	0	0	0
4292200	3142100	0	4889900	0	4757200	1806100	7647800	6845600
2934800	1331400	0	0	0	1485100	1253200	2336000	0
0	0	0	0	0	0	0	0	0
0	0	0	0	0	0	0	0	0
0	0	212560	366920	0	0	0	0	0
0	0	0	0	0	0	0	0	0
0	0	0	0	0	0	0	0	0
6504800	2255200	0	3105500	3058800	1421600	0	2163900	4506300
0	0	0	0	0	6613100	0	0	5154500
0	0	0	0	0	0	0	0	0
0	0	0	0	0	0	0	0	0
2219900	0	0	0	0	1770400	1085300	0	0
578070	644800	2686800	0	0	2916900	1528900	797420	2331000
0	0	0	0	0	0	0	0	0
1952700	1665500	0	0	0	0	1533600	2820800	7344100
3461500	4747100	1912700	1941900	2667200	1189900	676080	2861000	3097500
0	0	0	0	0	0	0	0	0
0	0	0	0	0	0	0	0	0
0	0	0	0	0	0	0	0	0
0	0	0	0	0	0	0	0	0
5649500	0	0	0	0	0	0	0	0
0	0	0	0	0	0	0	0	0
0	3669400	0	0	0	0	0	5692700	10200000
4147800	3466700	4825600	0	11100000	0	2932200	4910100	9249500
0	0	0	0	0	0	0	0	0
0	0	3995200	0	5480200	0	0	0	15400000
2225300	0	0	0	5876300	4651400	3064300	3338900	5808300

2314000	0	0	0	0	0	0	0	0
0	0	0	0	0	0	0	0	0
0	0	0	0	3385600	0	0	0	0
1178900	0	0	0	0	0	0	2424900	0
870040	0	3777000	2078800	2666100	0	1926000	3372400	0
3323200	4350500	5046600	4195700	8577300	2653100	1637400	6562400	9209000
331260	0	0	0	0	0	0	0	0
18600000	11600000	16900000	22400000	30300000	8313400	2253000	23600000	9094100
0	0	0	0	0	0	0	0	0
0	0	0	0	0	0	0	0	0
1866000	0	0	0	0	0	0	0	0
0	0	6045700	6964700	8240500	0	0	7343900	9094100
0	0	0	0	0	0	0	0	0
3037900	3614900	4222800	0	5766300	4141200	2611600	6043900	1.60E+07
0	4963300	11900000	0	10600000	3210700	2396400	6180800	0
0	0	0	457280	0	0	0	0	0
3301400	2698000	0	5469000	9516400	0	0	4080600	2689200
0	0	0	0	0	0	1069400	0	0
0	1582300	0	0	3569400	0	0	1453300	0
34700000	24900000	24400000	0	24700000	19500000	23200000	43800000	8469200
0	0	0	0	0	0	0	0	0
0	0	0	0	0	0	0	0	0
0	0	0	0	0	0	0	0	0
0	0	0	0	0	0	0	0	0
0	0	4086000	0	4837900	1385000	0	0	11100000
0	2010000	5238500	0	0	1819900	1424100	0	0
0	0	0	0	0	0	0	13300000	0
0	0	0	0	0	0	0	0	0
0	0	0	0	0	0	0	0	0
1814500	1988500	0	0	0	2770200	1528500	2423200	0
0	0	0	0	0	0	0	0	0
5647800	4795300	9101600	0	0	3882500	2928500	8628000	0
0	0	0	0	0	0	0	0	0
1908000	1175300	0	0	0	0	0	0	0
0	0	0	0	0	0	0	0	0
0	0	0	0	0	0	0	0	0
0	0	0	0	0	0	0	0	0
0	0	0	0	0	0	0	0	0
0	0	0	0	0	0	0	0	0
5050200	2757800	0	0	0	0	1392400	3859000	0
0	0	0	0	0	0	0	0	0
3331900	3398900	0	1943200	3100700	2349200	1684100	5344900	9313300
0	0	0	0	0	0	0	0	0
4403500	0	0	0	0	0	0	0	0
0	0	0	0	0	0	0	0	0
0	0	0	0	0	0	0	0	0
0	0	0	0	0	0	0	0	0

0	0	0	0	0	0	0	0	0
0	0	0	0	0	0	0	0	0
31800000	0	61100000	57100000	96700000	29900000	0	63200000	90700000
0	2691200	0	0	3426100	0	1145100	0	0
0	0	0	0	0	0	0	0	0
0	0	3945600	0	0	0	0	0	0
0	0	0	0	0	0	0	0	0
0	0	0	0	0	0	0	0	0
24800000	27500000	39600000	1.70E+07	33400000	14600000	9463900	34600000	33800000
0	0	0	0	0	0	0	0	0
0	0	0	0	0	0	0	0	0
0	0	0	0	0	0	0	0	0
0	0	0	0	0	0	0	0	0
0	0	0	791960	0	49400000	0	0	0
0	0	0	0	0	0	0	0	0
0	0	0	0	0	0	0	0	0
0	0	0	0	0	0	0	0	0
0	0	0	0	0	0	0	0	0
64400000	57200000	96100000	1.15E+08	1.60E+08	95300000	33100000	1.11E+08	1.08E+08
0	0	0	0	0	0	0	0	0
0	0	0	0	0	0	0	0	0
2177800	1737900	2.10E+07	1.60E+07	5779200	8400200	1387500	4828700	36100000
0	0	0	0	0	0	0	0	0
0	0	0	0	0	0	0	0	0
NA	NA	1.02E+09	9.91E+08	1.30E+09	NA	NA	NA	1.57E+09
NA	NA	6.32E+08	5.28E+08	1.44E+08	NA	NA	NA	8.21E+08
NA	NA	1.78E+10	1.62E+10	2.12E+10	NA	NA	NA	2.80E+10
NA	NA	6.93E+08	6.39E+08	3.48E+08	NA	NA	NA	5.36E+08
NA	NA	5.51E+08	5.08E+08	8.53E+08	NA	NA	NA	7.60E+08
NA	NA	2.17E+09	2.00E+09	2.54E+09	NA	NA	NA	2.56E+09
NA	NA	3.41E+08	3.41E+08	5.44E+08	NA	NA	NA	6.27E+08
NA	NA	8.30E+08	1.24E+09	8.43E+08	NA	NA	NA	9.90E+08
NA	NA	1.01E+08	1.13E+08	2.05E+08	NA	NA	NA	2.08E+08
NA	NA	2.45E+08	2.74E+08	2.86E+08	NA	NA	NA	3.83E+08
NA	NA	8778200	901030	37600000	NA	NA	NA	0
NA	NA	2.87E+08	2.97E+08	4.63E+08	NA	NA	NA	3.43E+08
NA	NA	2.49E+08	2.99E+08	3.28E+08	NA	NA	NA	3.21E+08
NA	NA	1.79E+08	95500000	2.71E+08	NA	NA	NA	2.94E+08
NA	NA	6.80E+07	55800000	1.14E+08	NA	NA	NA	1.34E+08
NA	NA	68800000	45200000	92700000	NA	NA	NA	1.08E+08
NA	NA	2.71E+08	2.28E+08	3.97E+08	NA	NA	NA	4.68E+08
NA	NA	1.16E+08	87400000	1.55E+08	NA	NA	NA	1.52E+08
NA	NA	5.03E+08	3.21E+08	4.08E+08	NA	NA	NA	5.61E+08
NA	NA	85200000	76700000	55100000	NA	NA	NA	86200000
NA	NA	35400000	45800000	1.09E+08	NA	NA	NA	93300000
NA	NA	1.04E+08	1.04E+08	1.09E+08	NA	NA	NA	2.21E+08
NA	NA	1.49E+08	75500000	1.67E+08	NA	NA	NA	1.04E+08

NA	NA	27400000	37300000	46800000	NA	NA	NA	57900000
NA	NA	1.55E+08	1.51E+08	1.92E+08	NA	NA	NA	2.24E+08
NA	NA	1.12E+08	63800000	1.30E+08	NA	NA	NA	1.19E+08
NA	NA	26200000	32900000	32800000	NA	NA	NA	26200000
NA	NA	3745500	1456600	0	NA	NA	NA	0
NA	NA	16300000	5.40E+07	60300000	NA	NA	NA	44500000
NA	NA	19400000	19200000	20500000	NA	NA	NA	34900000
NA	NA	43500000	65100000	72400000	NA	NA	NA	83200000
NA	NA	33400000	60200000	48900000	NA	NA	NA	67800000
NA	NA	1.56E+08	1.58E+08	2.57E+08	NA	NA	NA	3.49E+08
NA	NA	55800000	0	68900000	NA	NA	NA	43400000
NA	NA	6.30E+07	79100000	1.18E+08	NA	NA	NA	77500000
NA	NA	76900000	73100000	1.18E+08	NA	NA	NA	1.36E+08
NA	NA	25600000	21400000	13300000	NA	NA	NA	38700000
NA	NA	2605300	9164400	0	NA	NA	NA	4327300
NA	NA	16700000	22200000	17400000	NA	NA	NA	34200000
NA	NA	24700000	12600000	32800000	NA	NA	NA	19200000
NA	NA	49500000	1.80E+07	47700000	NA	NA	NA	56100000
NA	NA	3.60E+07	45400000	23600000	NA	NA	NA	66900000
NA	NA	1499600	32500000	0	NA	NA	NA	0
NA	NA	36500000	22400000	56400000	NA	NA	NA	4.60E+07
NA	NA	46900000	39700000	42700000	NA	NA	NA	52800000
NA	NA	53800000	3.60E+07	74100000	NA	NA	NA	86200000
NA	NA	24100000	1.60E+07	31300000	NA	NA	NA	51700000
NA	NA	2.70E+07	3.00E+07	72100000	NA	NA	NA	80200000
NA	NA	44600000	28400000	68800000	NA	NA	NA	54100000
NA	NA	5009000	29700000	7165400	NA	NA	NA	12600000
NA	NA	49700000	23800000	5.10E+07	NA	NA	NA	4.60E+07
NA	NA	10100000	26300000	33200000	NA	NA	NA	3.10E+07
NA	NA	5217100	12800000	24400000	NA	NA	NA	16700000
NA	NA	11400000	6954200	8556200	NA	NA	NA	2904500
NA	NA	36800000	20800000	54200000	NA	NA	NA	65700000
NA	NA	0	0	1373200	NA	NA	NA	0
NA	NA	7512600	16300000	6245900	NA	NA	NA	0
NA	NA	7406600	0	0	NA	NA	NA	0
NA	NA	0	2956100	0	NA	NA	NA	8171800
NA	NA	2860900	35400000	1.30E+07	NA	NA	NA	13500000
NA	NA	0	9670700	0	NA	NA	NA	733860
NA	NA	41700000	681800	5.50E+08	NA	NA	NA	37500000
NA	NA	11500000	13700000	1.90E+07	NA	NA	NA	33900000
NA	NA	0	0	0	NA	NA	NA	1087300
NA	NA	5191800	11700000	16800000	NA	NA	NA	1.60E+07
NA	NA	0	0	4679100	NA	NA	NA	1695500
NA	NA	3.55E+08	3.15E+08	1.34E+08	NA	NA	NA	5.76E+08
NA	NA	13300000	0	0	NA	NA	NA	0
NA	NA	16400000	1025900	19400000	NA	NA	NA	22700000
NA	NA	9827300	0	13100000	NA	NA	NA	0

NA	NA	0	0	0	NA	NA	NA	0
NA	NA	0	0	714230	NA	NA	NA	0
NA	NA	1545200	1566800	2767800	NA	NA	NA	2479000
NA	NA	1.40E+07	33900000	19600000	NA	NA	NA	22400000
NA	NA	6189800	11100000	15200000	NA	NA	NA	13800000
NA	NA	0	0	2975800	NA	NA	NA	0
NA	NA	0	19400000	0	NA	NA	NA	42900000
NA	NA	0	5352500	0	NA	NA	NA	8020200
NA	NA	21100000	5302900	1.90E+07	NA	NA	NA	21900000
NA	NA	35700000	12700000	13600000	NA	NA	NA	20300000
NA	NA	1551800	9854400	7299400	NA	NA	NA	4146300
NA	NA	0	1885500	0	NA	NA	NA	0
NA	NA	0	0	0	NA	NA	NA	0
NA	NA	13700000	6919500	14500000	NA	NA	NA	18400000
NA	NA	10900000	1.50E+07	18700000	NA	NA	NA	19200000
NA	NA	0	0	21400000	NA	NA	NA	0
NA	NA	8157100	15900000	8465400	NA	NA	NA	9266700
NA	NA	0	0	0	NA	NA	NA	5092200
NA	NA	24100000	22600000	25200000	NA	NA	NA	30600000
NA	NA	4714600	2244300	12300000	NA	NA	NA	4676000
NA	NA	17600000	1.10E+07	8280900	NA	NA	NA	18800000
NA	NA	0	0	0	NA	NA	NA	0
NA	NA	0	465450	0	NA	NA	NA	51900000
NA	NA	0	0	0	NA	NA	NA	0
NA	NA	2310500	0	12900000	NA	NA	NA	703860
NA	NA	10400000	9193200	7143900	NA	NA	NA	0
NA	NA	7009400	0	0	NA	NA	NA	5168800
NA	NA	37700000	5291700	22300000	NA	NA	NA	8516500
NA	NA	0	0	0	NA	NA	NA	0
NA	NA	0	0	1096400	NA	NA	NA	0
NA	NA	6523000	6324400	12300000	NA	NA	NA	23800000
NA	NA	7605600	5817800	8742300	NA	NA	NA	8455700
NA	NA	6763000	0	3770400	NA	NA	NA	27600000
NA	NA	9525700	7077400	27700000	NA	NA	NA	31600000
NA	NA	23900000	10600000	0	NA	NA	NA	6672100
NA	NA	0	0	0	NA	NA	NA	0
NA	NA	0	2263500	15800000	NA	NA	NA	13800000
NA	NA	0	0	0	NA	NA	NA	759490
NA	NA	5407800	2988300	16800000	NA	NA	NA	0
NA	NA	1148500	2054600	1293300	NA	NA	NA	228070
NA	NA	0	0	0	NA	NA	NA	0
NA	NA	0	0	0	NA	NA	NA	0
NA	NA	0	0	0	NA	NA	NA	14400000
NA	NA	14700000	5174300	11300000	NA	NA	NA	19300000
NA	NA	3424300	1331100	0	NA	NA	NA	0
NA	NA	0	0	0	NA	NA	NA	0
NA	NA	29200000	0	0	NA	NA	NA	2670700

NA	NA	0	25200000	0	NA	NA	NA	0
NA	NA	0	0	0	NA	NA	NA	0
NA	NA	1883400	4456900	6492900	NA	NA	NA	7604100
NA	NA	7939300	28200000	17400000	NA	NA	NA	1.70E+07
NA	NA	225930	8195000	0	NA	NA	NA	6684900
NA	NA	0	5125300	4251200	NA	NA	NA	7468800
NA	NA	843490	1608500	642140	NA	NA	NA	0
NA	NA	6699500	8907100	10600000	NA	NA	NA	11200000
NA	NA	12400000	0	0	NA	NA	NA	6177500
NA	NA	0	0	0	NA	NA	NA	0
NA	NA	28800000	24400000	48800000	NA	NA	NA	4.20E+07
NA	NA	19700000	18800000	23500000	NA	NA	NA	2.60E+07
NA	NA	501700	593040	894570	NA	NA	NA	2715900
NA	NA	2626600	1735000	2509200	NA	NA	NA	2439000
NA	NA	0	0	0	NA	NA	NA	0
NA	NA	0	0	0	NA	NA	NA	0
NA	NA	4476500	3936500	11900000	NA	NA	NA	9504000
NA	NA	1418900	2627900	5435400	NA	NA	NA	1725900
NA	NA	5904400	4951100	13500000	NA	NA	NA	19800000
NA	NA	0	2566700	0	NA	NA	NA	0
NA	NA	0	0	0	NA	NA	NA	0
NA	NA	2538200	0	0	NA	NA	NA	0
NA	NA	980480	0	3491500	NA	NA	NA	0
NA	NA	1.57E+08	1.19E+08	2.95E+08	NA	NA	NA	3.24E+08
NA	NA	9405000	0	0	NA	NA	NA	0
NA	NA	5036300	6537500	0	NA	NA	NA	0
NA	NA	5250900	3730000	0	NA	NA	NA	0
NA	NA	2737900	3043500	6592000	NA	NA	NA	17900000
NA	NA	0	0	0	NA	NA	NA	0
NA	NA	0	0	0	NA	NA	NA	0
NA	NA	0	0	4691300	NA	NA	NA	9413100
NA	NA	0	2753500	0	NA	NA	NA	6784800
NA	NA	560530	4585300	3697000	NA	NA	NA	4774700
NA	NA	0	0	0	NA	NA	NA	0
NA	NA	0	0	1374200	NA	NA	NA	0
NA	NA	0	0	0	NA	NA	NA	0
NA	NA	16200000	1.50E+07	27600000	NA	NA	NA	13300000
NA	NA	0	0	4241600	NA	NA	NA	0
NA	NA	0	0	0	NA	NA	NA	0
NA	NA	4231700	3964700	6443100	NA	NA	NA	4039200
NA	NA	3.20E+07	49200000	72600000	NA	NA	NA	7.20E+07
NA	NA	821250	889560	2418300	NA	NA	NA	646960
NA	NA	0	0	5119300	NA	NA	NA	0
NA	NA	0	0	0	NA	NA	NA	0
NA	NA	0	0	9934200	NA	NA	NA	0
NA	NA	15100000	0	0	NA	NA	NA	0
NA	NA	0	0	0	NA	NA	NA	0

NA	NA	0	0	0	NA	NA	NA	0
NA	NA	0	0	0	NA	NA	NA	0
NA	NA	2360800	2068600	0	NA	NA	NA	3806200
NA	NA	0	0	0	NA	NA	NA	0
NA	NA	0	0	0	NA	NA	NA	0
NA	NA	3436500	0	0	NA	NA	NA	3118800
NA	NA	0	377020	0	NA	NA	NA	501340
NA	NA	1128700	1128200	6950500	NA	NA	NA	1458200
NA	NA	1659200	2003200	1628000	NA	NA	NA	934860
NA	NA	0	0	0	NA	NA	NA	0
NA	NA	2966100	0	0	NA	NA	NA	0
NA	NA	0	0	2094400	NA	NA	NA	1675900
NA	NA	4261300	3693800	5579600	NA	NA	NA	9130600
NA	NA	8344100	7856400	10100000	NA	NA	NA	9880400
NA	NA	0	0	0	NA	NA	NA	0
NA	NA	3209400	5359400	3791800	NA	NA	NA	4066900
NA	NA	0	0	0	NA	NA	NA	0
NA	NA	0	8872300	0	NA	NA	NA	0
NA	NA	0	0	9106600	NA	NA	NA	0
NA	NA	4205500	4693400	4770800	NA	NA	NA	3993600
NA	NA	0	0	1798100	NA	NA	NA	507460
NA	NA	0	0	0	NA	NA	NA	0
NA	NA	13600000	0	24400000	NA	NA	NA	30700000
NA	NA	7513800	4650500	6210100	NA	NA	NA	11700000
NA	NA	0	3.50E+07	0	NA	NA	NA	0
NA	NA	6432000	5018000	0	NA	NA	NA	8080300
NA	NA	0	0	3910900	NA	NA	NA	1595300
NA	NA	0	0	0	NA	NA	NA	0
NA	NA	0	0	0	NA	NA	NA	0
NA	NA	0	0	0	NA	NA	NA	0
NA	NA	0	0	0	NA	NA	NA	0
NA	NA	1757300	0	2897500	NA	NA	NA	3808200
NA	NA	0	0	0	NA	NA	NA	0
NA	NA	9507300	0	3364500	NA	NA	NA	4134800
NA	NA	0	0	689500	NA	NA	NA	705460
NA	NA	0	4883700	7124700	NA	NA	NA	6629800
NA	NA	0	0	0	NA	NA	NA	0
NA	NA	0	0	0	NA	NA	NA	0
NA	NA	0	0	0	NA	NA	NA	0
NA	NA	0	0	0	NA	NA	NA	0
NA	NA	0	0	0	NA	NA	NA	0
NA	NA	0	0	490470	NA	NA	NA	9924000
NA	NA	670540	0	0	NA	NA	NA	0
NA	NA	0	0	7683300	NA	NA	NA	0
NA	NA	0	0	0	NA	NA	NA	0
NA	NA	0	0	0	NA	NA	NA	0
NA	NA	0	2989500	4826800	NA	NA	NA	0

NA	NA	5336600	8207200	18300000	NA	NA	NA	7291200
NA	NA	0	0	5147500	NA	NA	NA	0
NA	NA	0	0	0	NA	NA	NA	0
NA	NA	0	0	0	NA	NA	NA	57300000
NA	NA	1873500	2163200	2188400	NA	NA	NA	1138100
NA	NA	0	0	374030	NA	NA	NA	9472600
NA	NA	0	0	8385800	NA	NA	NA	8046700
NA	NA	3594100	0	0	NA	NA	NA	0
NA	NA	0	0	4079300	NA	NA	NA	0
NA	NA	0	7812900	0	NA	NA	NA	6875900
NA	NA	0	0	0	NA	NA	NA	0
NA	NA	2151000	0	3259900	NA	NA	NA	4617500
NA	NA	2298800	0	0	NA	NA	NA	0
NA	NA	0	9651700	24400000	NA	NA	NA	30700000
NA	NA	0	3173600	0	NA	NA	NA	5011100
NA	NA	0	0	2515300	NA	NA	NA	2985800
NA	NA	0	949390	839110	NA	NA	NA	0
NA	NA	0	0	0	NA	NA	NA	0
NA	NA	0	0	8498700	NA	NA	NA	0
NA	NA	0	6475600	0	NA	NA	NA	0
NA	NA	0	0	0	NA	NA	NA	0
NA	NA	379400	1055200	622500	NA	NA	NA	0
NA	NA	0	0	0	NA	NA	NA	341500
NA	NA	0	0	0	NA	NA	NA	0
NA	NA	0	0	0	NA	NA	NA	0
NA	NA	0	0	0	NA	NA	NA	0
NA	NA	0	0	0	NA	NA	NA	1566300
NA	NA	0	0	0	NA	NA	NA	0
NA	NA	0	0	769600	NA	NA	NA	0
NA	NA	0	0	0	NA	NA	NA	0
NA	NA	0	0	0	NA	NA	NA	0
NA	NA	0	0	0	NA	NA	NA	0
NA	NA	0	0	0	NA	NA	NA	0
NA	NA	0	0	6526500	NA	NA	NA	0
NA	NA	0	0	0	NA	NA	NA	3538500
NA	NA	0	3103500	0	NA	NA	NA	0
NA	NA	0	0	0	NA	NA	NA	0
NA	NA	0	0	0	NA	NA	NA	0
NA	NA	0	2632800	0	NA	NA	NA	0
NA	NA	2082600	1972800	2208100	NA	NA	NA	0
NA	NA	0	0	6157500	NA	NA	NA	0
NA	NA	0	0	0	NA	NA	NA	0
NA	NA	1451800	1497700	1316900	NA	NA	NA	1533700
NA	NA	0	0	2894700	NA	NA	NA	0
NA	NA	304520	404980	0	NA	NA	NA	0
NA	NA	0	0	0	NA	NA	NA	0
NA	NA	0	0	0	NA	NA	NA	2384400

NA	NA	0	0	0	NA	NA	NA	0
NA	NA	0	0	0	NA	NA	NA	0
NA	NA	0	0	3199200	NA	NA	NA	0
NA	NA	0	0	0	NA	NA	NA	1873200
NA	NA	0	0	17600000	NA	NA	NA	0
NA	NA	6384900	6631400	10300000	NA	NA	NA	14200000
NA	NA	0	0	0	NA	NA	NA	0
NA	NA	0	0	0	NA	NA	NA	0
NA	NA	0	0	0	NA	NA	NA	0
NA	NA	4064100	7687300	5446600	NA	NA	NA	5612700
NA	NA	0	0	0	NA	NA	NA	0
NA	NA	0	0	0	NA	NA	NA	0
NA	NA	0	0	0	NA	NA	NA	0
NA	NA	4709300	4395000	12900000	NA	NA	NA	9510500
NA	NA	0	0	0	NA	NA	NA	0
NA	NA	0	0	0	NA	NA	NA	0
NA	NA	0	0	0	NA	NA	NA	0
NA	NA	0	0	0	NA	NA	NA	0
NA	NA	0	0	0	NA	NA	NA	0
NA	NA	0	2989500	0	NA	NA	NA	0
NA	NA	0	0	2092100	NA	NA	NA	0
NA	NA	0	0	0	NA	NA	NA	4332400
NA	NA	0	0	0	NA	NA	NA	0
NA	NA	0	0	0	NA	NA	NA	0
NA	NA	0	0	0	NA	NA	NA	0
NA	NA	0	0	0	NA	NA	NA	0
NA	NA	0	0	0	NA	NA	NA	0
NA	NA	35800000	20200000	34100000	NA	NA	NA	1.08E+08
NA	NA	0	0	0	NA	NA	NA	7084900
NA	NA	0	0	0	NA	NA	NA	0
NA	NA	0	0	0	NA	NA	NA	0
NA	NA	0	0	0	NA	NA	NA	0
NA	NA	0	0	0	NA	NA	NA	0
NA	NA	0	0	0	NA	NA	NA	0
NA	NA	0	0	0	NA	NA	NA	0
NA	NA	0	0	0	NA	NA	NA	0
NA	NA	0	0	0	NA	NA	NA	0
NA	NA	0	0	0	NA	NA	NA	0
NA	NA	17500000	16700000	2.50E+07	NA	NA	NA	25100000
NA	NA	0	0	0	NA	NA	NA	0
NA	NA	0	0	17800000	NA	NA	NA	3585400
NA	NA	0	0	0	NA	NA	NA	0
NA	NA	0	0	0	NA	NA	NA	0
NA	NA	0	0	0	NA	NA	NA	0
NA	NA	0	0	0	NA	NA	NA	0
NA	NA	0	0	0	NA	NA	NA	0
NA	NA	0	0	0	NA	NA	NA	0

NA	NA	0	0	0	NA	NA	NA	0
NA	NA	0	0	0	NA	NA	NA	0
NA	NA	0	0	0	NA	NA	NA	0
NA	NA	0	0	0	NA	NA	NA	0
NA	NA	0	0	0	NA	NA	NA	0
NA	NA	0	0	0	NA	NA	NA	0
NA	NA	0	0	0	NA	NA	NA	0
NA	NA	0	0	0	NA	NA	NA	0
NA	NA	0	0	0	NA	NA	NA	0
NA	NA	0	0	0	NA	NA	NA	0
NA	NA	0	0	0	NA	NA	NA	0
NA	NA	40900000	2584600	47800000	NA	NA	NA	75500000
NA	NA	0	0	9907700	NA	NA	NA	21100000
NA	NA	7143600	5844000	7497500	NA	NA	NA	7717000
NA	NA	0	0	24400000	NA	NA	NA	41800000
NA	NA	4202100	3787900	4754600	NA	NA	NA	7299200
NA	NA	0	0	0	NA	NA	NA	0
NA	NA	0	0	0	NA	NA	NA	0
NA	NA	0	0	0	NA	NA	NA	0
NA	NA	0	0	0	NA	NA	NA	0
NA	NA	0	0	0	NA	NA	NA	0
NA	NA	3538200	3861400	3229400	NA	NA	NA	6151600
NA	NA	0	0	0	NA	NA	NA	0
NA	NA	0	0	0	NA	NA	NA	0
NA	NA	0	0	0	NA	NA	NA	0
NA	NA	0	0	0	NA	NA	NA	0
NA	NA	0	0	0	NA	NA	NA	0
NA	NA	0	0	0	NA	NA	NA	0
NA	NA	0	0	0	NA	NA	NA	0
NA	NA	0	0	0	NA	NA	NA	0
NA	NA	0	0	0	NA	NA	NA	0
NA	NA	0	0	0	NA	NA	NA	0
NA	NA	0	0	0	NA	NA	NA	0
NA	NA	0	0	0	NA	NA	NA	0
NA	NA	0	0	0	NA	NA	NA	0
NA	NA	0	0	0	NA	NA	NA	0
NA	NA	8.10E+07	3.80E+07	63700000	NA	NA	NA	1.30E+08
NA	NA	531960	592240	2682200	NA	NA	NA	407100
NA	NA	0	0	0	NA	NA	NA	0
NA	NA	8962000	9044300	8902200	NA	NA	NA	0
4.53E+08	4.92E+08	NA	NA	NA	4.23E+08	3.44E+08	9.04E+08	NA
9.74E+09	1.00E+10	NA	NA	NA	1.04E+10	7.04E+09	1.58E+10	NA
9.57E+08	8.65E+08	NA	NA	NA	1.00E+09	7.07E+08	1.75E+09	NA
3.33E+08	2.04E+08	NA	NA	NA	2.79E+08	2.39E+08	1.38E+08	NA
1.77E+08	2.01E+08	NA	NA	NA	1.51E+08	1.30E+08	3.11E+08	NA
2.42E+08	2.09E+08	NA	NA	NA	2.54E+08	1.78E+08	4.88E+08	NA
1.13E+08	1.38E+08	NA	NA	NA	1.29E+08	1.01E+08	2.65E+08	NA

3.35E+08	3.65E+08	NA	NA	NA	3.27E+08	2.45E+08	4.66E+08	NA	
0	2431600	NA	NA	NA	7779300	4059400	3843600	NA	
60400000	56800000	NA	NA	NA	53400000	4.60E+07	1.26E+08	NA	
1.17E+08	1.13E+08	NA	NA	NA	1.13E+08	9.30E+07	2.44E+08	NA	
1.42E+08	1.33E+08	NA	NA	NA	1.27E+08	1.01E+08	2.34E+08	NA	
27800000	35200000	NA	NA	NA	4.50E+07	29200000	50400000	NA	
65700000	1.38E+08	NA	NA	NA	1.04E+08	73100000	3.28E+08	NA	
4.80E+07	40900000	NA	NA	NA	35900000	31400000	84600000	NA	
2.79E+08	2.30E+08	NA	NA	NA	2.51E+08	1.05E+08	2.96E+08	NA	
12400000	63500000	NA	NA	NA	53900000	33400000	61800000	NA	
1.70E+08	1.37E+08	NA	NA	NA	1.35E+08	1.04E+08	1.77E+08	NA	
21500000	41100000	NA	NA	NA	47800000	25700000	80100000	NA	
32400000	40700000	NA	NA	NA	41100000	19500000	77200000	NA	
1.23E+08	1.04E+08	NA	NA	NA	1.12E+08	90400000	2.29E+08	NA	
14600000	1.10E+07	NA	NA	NA	8533700	5093200	16300000	NA	
57500000	37100000	NA	NA	NA	48800000	44100000	66300000	NA	
17800000	10600000	NA	NA	NA	14100000	10800000	21600000	NA	
51600000	44700000	NA	NA	NA	49200000	34300000	95200000	NA	
32900000	44700000	NA	NA	NA	54400000	36800000	96100000	NA	
42400000	2.10E+07	NA	NA	NA	41500000	2.10E+07	84700000	NA	
2364800	7918600	NA	NA	NA	8286100	3576700	2358500	NA	
3687500	12500000	NA	NA	NA	11600000	4066900	12600000	NA	
38500000	33700000	NA	NA	NA	31100000	30800000	58100000	NA	
31200000	16200000	NA	NA	NA	21700000	17400000	28800000	NA	
14700000	20100000	NA	NA	NA	2.10E+07	11900000	31900000	NA	
9332300	1.10E+07	NA	NA	NA	6700400	7331100	20900000	NA	
0	0	NA	NA	NA	0	0	0	NA	
91500000	80200000	NA	NA	NA	69700000	59600000	1.88E+08	NA	
16200000	19500000	NA	NA	NA	25100000	14400000	43900000	NA	
1.10E+07	12400000	NA	NA	NA	14900000	1.10E+07	20500000	NA	
20900000	28500000	NA	NA	NA	31300000	22300000	51200000	NA	
10100000	6629400	NA	NA	NA	6087700	1563900	8757000	NA	
7849700	8535800	NA	NA	NA	8638100	9736200	13100000	NA	
13500000	13800000	NA	NA	NA	11400000	7350000	24300000	NA	
1.70E+07	21600000	NA	NA	NA	14700000	16700000	34600000	NA	
6777700	14100000	NA	NA	NA	13300000	10700000	28900000	NA	
2765000	4415800	NA	NA	NA	1937600	2001900	2934400	NA	
10300000	3841700	NA	NA	NA	1833500	3314800	8935000	NA	
595690	0	NA	NA	NA	493040	0	553430	NA	
10100000	14800000	NA	NA	NA	23600000	10700000	23400000	NA	
6589900	7357000	NA	NA	NA	9699900	8239200	13200000	NA	
0	0	NA	NA	NA	18800000	0	22900000	NA	
11200000	16800000	NA	NA	NA	16900000	11900000	40200000	NA	
970300	4598400	NA	NA	NA	0	0	3269100	NA	
6562300	8703400	NA	NA	NA	10900000	3138400	11400000	NA	
12900000	18200000	NA	NA	NA	11900000	10300000	21500000	NA	
14300000	11600000	NA	NA	NA	2562500	13800000	1.60E+07	NA	

8082800	2020600	NA	NA	NA	1630200	5330800	4659200	NA
0	0	NA	NA	NA	4.70E+07	0	0	NA
0	0	NA	NA	NA	0	0	2720300	NA
9329400	8564100	NA	NA	NA	5061900	6933900	19500000	NA
0	0	NA	NA	NA	2869000	792740	0	NA
0	3501000	NA	NA	NA	0	7214000	0	NA
7388000	7239900	NA	NA	NA	7711600	458310	19400000	NA
8760500	8611300	NA	NA	NA	9715900	3804400	28300000	NA
3697800	4639300	NA	NA	NA	11200000	3645800	10800000	NA
3281200	1239500	NA	NA	NA	1993900	1341800	3224200	NA
14700000	33700000	NA	NA	NA	22600000	20700000	40300000	NA
2441900	2436600	NA	NA	NA	1543900	987800	4187000	NA
6046400	6349900	NA	NA	NA	8404000	4727700	17500000	NA
7615000	6015600	NA	NA	NA	7898400	4502100	13100000	NA
14900000	13400000	NA	NA	NA	7944000	5368700	25200000	NA
1521300	992340	NA	NA	NA	1186500	280930	2403300	NA
5034800	8314000	NA	NA	NA	8216000	2032500	1.40E+07	NA
8619500	0	NA	NA	NA	4290600	43700000	17400000	NA
1023100	1577700	NA	NA	NA	657490	1260100	1832400	NA
2259100	7831900	NA	NA	NA	8211700	8775200	19100000	NA
5274400	8845200	NA	NA	NA	5335800	2704200	1.20E+07	NA
14300000	3069100	NA	NA	NA	11300000	2059600	21700000	NA
1366900	0	NA	NA	NA	0	588020	7355200	NA
1202900	863770	NA	NA	NA	981360	919900	2046200	NA
0	0	NA	NA	NA	0	0	0	NA
0	3125900	NA	NA	NA	0	0	2.66E+08	NA
1.70E+07	25600000	NA	NA	NA	20200000	16100000	37100000	NA
2887400	2577800	NA	NA	NA	3643700	1218100	8449000	NA
4619300	3921300	NA	NA	NA	5198800	2713200	8935100	NA
0	17500000	NA	NA	NA	13900000	0	0	NA
2125500	0	NA	NA	NA	480760	0	683280	NA
0	0	NA	NA	NA	0	0	0	NA
6172700	5722600	NA	NA	NA	5732900	3859700	12700000	NA
5969200	0	NA	NA	NA	2129900	745230	6146700	NA
0	1088200	NA	NA	NA	0	790130	8353300	NA
2205800	1668200	NA	NA	NA	0	1154400	5047600	NA
22500000	17700000	NA	NA	NA	19900000	16700000	27400000	NA
0	0	NA	NA	NA	0	0	0	NA
1074100	2310400	NA	NA	NA	0	2367800	11700000	NA
1852100	1968500	NA	NA	NA	438210	1576400	2561200	NA
1910300	0	NA	NA	NA	2270700	3886900	5170300	NA
4349900	2417800	NA	NA	NA	3335100	3183600	4195100	NA
15400000	4066100	NA	NA	NA	5929400	3289500	18300000	NA
0	0	NA	NA	NA	0	0	0	NA
19100000	3994200	NA	NA	NA	3487700	11100000	4285400	NA
3768400	5604800	NA	NA	NA	0	4773800	11100000	NA
9504200	1884000	NA	NA	NA	7907300	5722100	1.70E+07	NA

0	0	NA	NA	NA	0	0	2339200	NA
0	6224600	NA	NA	NA	188600	0	259540	NA
0	3267500	NA	NA	NA	2524200	3810800	5428000	NA
509070	540120	NA	NA	NA	0	0	746080	NA
363460	0	NA	NA	NA	430140	428590	0	NA
12900000	5506000	NA	NA	NA	12100000	8449600	22700000	NA
0	0	NA	NA	NA	4876300	1574200	0	NA
0	6133700	NA	NA	NA	2932100	0	4721800	NA
367000	0	NA	NA	NA	0	0	0	NA
0	0	NA	NA	NA	0	0	1.89E+08	NA
0	5276200	NA	NA	NA	0	5968200	0	NA
4682200	851170	NA	NA	NA	2487600	699600	5163200	NA
2924000	4139500	NA	NA	NA	0	0	1965500	NA
13300000	15700000	NA	NA	NA	9699300	9323400	26900000	NA
5122200	14700000	NA	NA	NA	3901300	2378700	6386600	NA
13100000	1.40E+07	NA	NA	NA	13300000	7387600	25400000	NA
2249000	1614100	NA	NA	NA	1743500	690630	6094300	NA
0	3335800	NA	NA	NA	1516100	2095000	3332100	NA
0	0	NA	NA	NA	0	0	1554300	NA
0	0	NA	NA	NA	0	0	0	NA
20300000	22700000	NA	NA	NA	17100000	13300000	21200000	NA
4781300	5088000	NA	NA	NA	4498600	1710100	4959600	NA
659940	0	NA	NA	NA	508460	0	1280600	NA
3782100	3920400	NA	NA	NA	3754900	1805400	12100000	NA
8531900	1521800	NA	NA	NA	3764800	3871900	8259400	NA
0	0	NA	NA	NA	0	0	0	NA
4781600	5654300	NA	NA	NA	6498000	2414500	9920900	NA
0	0	NA	NA	NA	0	0	0	NA
4876100	2743700	NA	NA	NA	1938900	1689800	7005000	NA
460090	2799900	NA	NA	NA	3514200	2849400	6897600	NA
0	0	NA	NA	NA	0	0	0	NA
1838700	8892000	NA	NA	NA	1599700	1314100	842140	NA
1799500	3669200	NA	NA	NA	4248100	2062700	7068900	NA
5646900	5599400	NA	NA	NA	3118200	2003000	8140700	NA
0	8455500	NA	NA	NA	437490	207130	0	NA
0	1856700	NA	NA	NA	1164000	663300	761420	NA
0	0	NA	NA	NA	0	0	0	NA
0	0	NA	NA	NA	0	2275600	5846800	NA
2517300	1016800	NA	NA	NA	1110000	899520	1688100	NA
17100000	4371500	NA	NA	NA	3654800	2119100	12900000	NA
0	0	NA	NA	NA	0	0	0	NA
9700600	3428400	NA	NA	NA	0	0	14700000	NA
0	366740	NA	NA	NA	712960	621070	678110	NA
3419000	3117000	NA	NA	NA	802420	627500	6697400	NA
4376100	2483700	NA	NA	NA	4667300	0	3254100	NA
4136000	2580100	NA	NA	NA	1280900	1556600	3059000	NA
1744700	1445200	NA	NA	NA	1422900	991630	2287900	NA

0	716150	NA	NA	NA	710720	274300	1218600	NA
3267600	1396100	NA	NA	NA	0	0	3942400	NA
0	0	NA	NA	NA	0	3501900	0	NA
0	0	NA	NA	NA	0	0	0	NA
0	0	NA	NA	NA	0	0	0	NA
0	483710	NA	NA	NA	0	0	0	NA
0	0	NA	NA	NA	0	0	0	NA
1834000	1723900	NA	NA	NA	1273600	3206100	6773100	NA
1.60E+07	2.00E+07	NA	NA	NA	19700000	13800000	36600000	NA
1226500	3549400	NA	NA	NA	1621800	1314600	2523700	NA
4018200	3906400	NA	NA	NA	2272900	1543600	4257700	NA
0	0	NA	NA	NA	0	9152500	0	NA
933620	2492900	NA	NA	NA	3028200	3023100	6457700	NA
0	0	NA	NA	NA	0	0	0	NA
0	0	NA	NA	NA	0	0	0	NA
9606400	8626100	NA	NA	NA	7799800	8062500	16400000	NA
1653300	1982600	NA	NA	NA	0	0	0	NA
1861000	1279000	NA	NA	NA	1604600	1916100	0	NA
6247400	0	NA	NA	NA	0	0	8121200	NA
1046800	1070000	NA	NA	NA	654330	567130	280030	NA
0	0	NA	NA	NA	1425700	0	0	NA
0	0	NA	NA	NA	0	0	0	NA
0	466250	NA	NA	NA	6259400	0	647910	NA
0	1935900	NA	NA	NA	2511100	1367500	3730100	NA
3107200	2198800	NA	NA	NA	1780000	819420	3046700	NA
0	0	NA	NA	NA	0	0	6290800	NA
0	0	NA	NA	NA	0	0	0	NA
1072800	0	NA	NA	NA	0	4028500	0	NA
722570	0	NA	NA	NA	0	48073	580670	NA
0	28300000	NA	NA	NA	0	0	0	NA
0	0	NA	NA	NA	313980	1863800	584930	NA
1255000	0	NA	NA	NA	0	0	0	NA
0	0	NA	NA	NA	0	0	0	NA
0	0	NA	NA	NA	0	0	0	NA
0	0	NA	NA	NA	0	0	0	NA
0	0	NA	NA	NA	0	0	0	NA
3281900	0	NA	NA	NA	0	0	6875800	NA
4307300	8082600	NA	NA	NA	12600000	7896100	21300000	NA
0	0	NA	NA	NA	0	0	0	NA
0	849940	NA	NA	NA	0	0	0	NA
0	0	NA	NA	NA	0	0	0	NA
0	0	NA	NA	NA	0	666960	2065300	NA
0	0	NA	NA	NA	0	0	0	NA
0	592000	NA	NA	NA	0	0	0	NA
2697400	0	NA	NA	NA	1382600	1142200	2277000	NA
1653900	2590500	NA	NA	NA	0	0	0	NA
0	7198000	NA	NA	NA	0	3522600	0	NA

955040	0	NA	NA	NA	0	0	0	NA
0	0	NA	NA	NA	0	0	1561700	NA
0	0	NA	NA	NA	4860600	4133200	18800000	NA
0	0	NA	NA	NA	0	0	0	NA
0	2319500	NA	NA	NA	2762500	1446700	3914200	NA
1885600	1520000	NA	NA	NA	1979200	1269800	7027800	NA
3.31E+08	3.11E+08	NA	NA	NA	3.83E+08	2.18E+08	7.61E+08	NA
0	0	NA	NA	NA	0	0	0	NA
1602600	0	NA	NA	NA	0	1009700	2271000	NA
0	0	NA	NA	NA	0	0	0	NA
6596900	0	NA	NA	NA	6979500	0	0	NA
9.80E+08	2.34E+09	NA	NA	NA	2.01E+09	1.52E+09	1.28E+09	NA
3994600	946810	NA	NA	NA	0	1465000	5762300	NA
0	0	NA	NA	NA	0	0	1508400	NA
0	0	NA	NA	NA	0	0	0	NA
0	0	NA	NA	NA	0	0	0	NA
0	0	NA	NA	NA	0	0	0	NA
2603600	0	NA	NA	NA	0	0	0	NA
0	0	NA	NA	NA	0	0	0	NA
2396300	4239300	NA	NA	NA	3732400	1476800	3382000	NA
683780	418770	NA	NA	NA	1172900	435970	942170	NA
1148500	728340	NA	NA	NA	1637300	928700	2545800	NA
2189300	2325100	NA	NA	NA	2226000	0	4906300	NA
3011900	955690	NA	NA	NA	945670	1089100	4856500	NA
0	0	NA	NA	NA	0	0	0	NA
0	0	NA	NA	NA	0	0	0	NA
0	0	NA	NA	NA	0	0	0	NA
1045100	0	NA	NA	NA	0	0	2077800	NA
0	0	NA	NA	NA	0	0	0	NA
1268400	0	NA	NA	NA	0	0	0	NA
0	0	NA	NA	NA	7926100	0	0	NA
0	0	NA	NA	NA	0	0	0	NA
0	0	NA	NA	NA	0	0	0	NA
3687300	0	NA	NA	NA	0	0	0	NA
0	0	NA	NA	NA	0	0	0	NA
0	0	NA	NA	NA	0	0	0	NA
0	1205200	NA	NA	NA	0	822040	3157300	NA
1618600	456070	NA	NA	NA	483340	0	862820	NA
0	0	NA	NA	NA	0	0	0	NA
0	0	NA	NA	NA	6763200	0	0	NA
0	0	NA	NA	NA	0	0	0	NA
2202900	0	NA	NA	NA	0	1089100	3032000	NA
1106200	0	NA	NA	NA	0	0	273300	NA
0	0	NA	NA	NA	0	0	0	NA
0	2449400	NA	NA	NA	436810	0	0	NA
16300000	2101000	NA	NA	NA	2620200	2580900	4285400	NA
0	0	NA	NA	NA	27400000	0	0	NA

0	0	NA	NA	NA	1319700	0	2785500	NA
0	0	NA	NA	NA	0	0	4729200	NA
0	0	NA	NA	NA	0	0	0	NA
0	0	NA	NA	NA	0	0	0	NA
0	0	NA	NA	NA	0	0	0	NA
0	0	NA	NA	NA	0	0	0	NA
0	0	NA	NA	NA	0	0	0	NA
0	0	NA	NA	NA	0	0	0	NA
0	0	NA	NA	NA	4333200	0	9132100	NA
3700400	3983000	NA	NA	NA	4499500	3823400	7271800	NA
0	0	NA	NA	NA	0	0	0	NA
0	0	NA	NA	NA	0	0	0	NA
0	0	NA	NA	NA	0	0	4206700	NA
0	0	NA	NA	NA	0	0	0	NA
0	0	NA	NA	NA	0	0	0	NA
0	4841300	NA	NA	NA	6014000	4054100	13700000	NA
0	0	NA	NA	NA	0	0	0	NA
0	0	NA	NA	NA	0	0	347660	NA
0	396240	NA	NA	NA	0	0	1632800	NA
0	0	NA	NA	NA	0	0	0	NA
0	0	NA	NA	NA	0	0	0	NA
0	0	NA	NA	NA	0	0	0	NA
0	0	NA	NA	NA	0	0	0	NA
0	0	NA	NA	NA	1166900	0	0	NA
0	0	NA	NA	NA	647380	412850	6662900	NA
0	0	NA	NA	NA	6.03E+08	8497500	0	NA
0	5276200	NA	NA	NA	0	3940400	0	NA
0	0	NA	NA	NA	0	0	0	NA
0	0	NA	NA	NA	0	0	0	NA
0	0	NA	NA	NA	0	0	0	NA
0	0	NA	NA	NA	0	0	0	NA
15200000	16600000	NA	NA	NA	16300000	7424000	3.00E+07	NA
0	0	NA	NA	NA	0	0	0	NA
0	0	NA	NA	NA	7.67E+08	0	0	NA
824510	0	NA	NA	NA	0	0	0	NA
41400000	36500000	NA	NA	NA	43500000	33800000	7.20E+07	NA
0	0	NA	NA	NA	0	0	0	NA
0	0	NA	NA	NA	0	0	0	NA
0	0	NA	NA	NA	0	0	0	NA
0	0	NA	NA	NA	0	0	0	NA
0	0	NA	NA	NA	0	0	0	NA
14500000	6242100	NA	NA	NA	6464300	4343300	10800000	NA
0	0	NA	NA	NA	0	0	0	NA
0	0	NA	NA	NA	0	0	0	NA
4876100	2743700	NA	NA	NA	1938900	1689800	7005000	NA
2066000	7240700	NA	NA	NA	8339700	5023400	15300000	NA
3106200	2630500	NA	NA	NA	3463700	1769600	6289300	NA

0	0	NA	NA	NA	0	0	2877100	NA
0	0	NA	NA	NA	0	0	0	NA
0	0	NA	NA	NA	0	0	0	NA
0	0	NA	NA	NA	0	0	0	NA
0	0	NA	NA	NA	0	0	0	NA
0	0	NA	NA	NA	0	0	0	NA
0	0	NA	NA	NA	0	0	0	NA
2204300	0	NA	NA	NA	0	0	3634000	NA
4883300	5327600	NA	NA	NA	4374500	5352500	10600000	NA
0	0	NA	NA	NA	0	0	0	NA
0	0	NA	NA	NA	0	0	0	NA
4140900	0	NA	NA	NA	1360800	1671200	8231900	NA
7467700	6527800	NA	NA	NA	0	5251400	16600000	NA
0	0	NA	NA	NA	0	0	0	NA
0	0	NA	NA	NA	0	0	2382300	NA
1361100	1047300	NA	NA	NA	1015200	612520	4776400	NA
5076700	27700000	NA	NA	NA	0	8260600	18300000	NA
NA	NA	55900000	40400000	49300000	NA	NA	NA	44800000
0	0	NA	NA	NA	0	0	0	NA
9022700	8198800	NA	NA	NA	2573400	3261000	8845800	NA
NA	NA	1465200	1603900	1506800	NA	NA	NA	1373300
793180	0	NA	NA	NA	0	0	0	NA
NA	NA	0	1605100	310320	NA	NA	NA	0
NA	NA	0	0	0	NA	NA	NA	0
5864300	0	NA	NA	NA	0	2683400	0	NA
526880	1782200	NA	NA	NA	1010000	1447500	1355100	NA
NA	NA	1.01E+08	1.26E+08	1.21E+08	NA	NA	NA	1.30E+08
NA	NA	12500000	4934400	0	NA	NA	NA	6307800
NA	NA	6715800	0	0	NA	NA	NA	2513100
5336000	8132300	NA	NA	NA	6547900	0	0	NA
NA	NA	1.10E+09	1.09E+09	1.31E+09	NA	NA	NA	1.26E+09
3412900	4465600	NA	NA	NA	7571200	2666900	8064300	NA
NA	NA	0	0	0	NA	NA	NA	0
NA	NA	2.56E+08	2.14E+08	3.81E+08	NA	NA	NA	3.93E+08
7147300	7093300	NA	NA	NA	8545800	8005400	17200000	NA
14700000	1.30E+07	24600000	19400000	34400000	11700000	11700000	26600000	41100000
12800000	13600000	NA	NA	NA	12500000	8742200	29800000	NA
NA	NA	91900000	79800000	94500000	NA	NA	NA	1.39E+08
NA	NA	0	6239300	15400000	NA	NA	NA	6933400
NA	NA	0	2576200	4465300	NA	NA	NA	2770100
0	0	NA	NA	NA	1294500	1697600	320370	NA
NA	NA	8086200	6872800	14200000	NA	NA	NA	6720300
NA	NA	0	0	19200000	NA	NA	NA	12200000
NA	NA	6.43E+08	5.94E+08	8.05E+08	NA	NA	NA	8.48E+08
20900000	20100000	NA	NA	NA	17700000	12700000	3.50E+07	NA
813090	712720	NA	NA	NA	722960	473920	1186000	NA
3568700	4881900	NA	NA	NA	3087900	2874900	6857400	NA

NA	NA	10700000	7742600	11200000	NA	NA	NA	2255900
NA	NA	7090600	10200000	11600000	NA	NA	NA	15300000
NA	NA	21300000	28700000	71400000	NA	NA	NA	67500000
NA	NA	0	6830300	21300000	NA	NA	NA	23200000
0	0	NA	NA	NA	0	0	0	NA

FFPE_SDS.I	FFPE_SDS.I	FFPE_SDS.I	FFPE_SDS.I	FFPE_SDS.MAX
**F6.1.M**	**F9.1.M**	**F2.2.M**	**F6.2.M**	**F9.2.M**
1.87E+09	1.87E+09	4.18E+09	1.04E+09	9.36E+08
3.78E+09	4.76E+09	2.60E+09	2.50E+09	1.88E+09
7.92E+09	9.47E+09	4.48E+09	4.56E+09	3.55E+09
2.58E+09	3.01E+09	1.20E+09	1.43E+09	9.85E+08
2.97E+09	3.72E+09	1.99E+09	2.22E+09	1.42E+09
2.98E+09	9.79E+08	4.47E+08	2.02E+09	4.08E+08
4.51E+09	2.20E+09	2.67E+09	2.44E+09	8.70E+08
1.05E+10	1.18E+10	6.37E+09	6.10E+09	4.16E+09
2.27E+09	3.24E+09	1.79E+09	1.53E+09	1.09E+09
1.32E+09	1.69E+09	9.36E+08	1.00E+09	6.85E+08
2.90E+09	3.83E+09	1.94E+09	1.93E+09	1.55E+09
1.27E+09	2.53E+09	3.78E+09	8.01E+08	1.08E+09
2.66E+09	3.15E+09	1.44E+09	1.48E+09	1.14E+09
1.81E+09	2.41E+09	9.32E+08	8.86E+08	8.30E+08
1.56E+09	1.78E+09	6.11E+08	6.75E+08	5.26E+08
2.09E+08	3.33E+08	1.82E+08	1.43E+08	1.53E+08
4.60E+08	6.27E+08	2.87E+08	2.74E+08	2.30E+08
2.75E+09	3.04E+09	1.65E+09	1.67E+09	1.21E+09
4.33E+08	4.71E+08	3.38E+08	3.33E+08	2.47E+08
9.47E+08	9.36E+08	5.94E+08	4.54E+08	3.99E+08
1.22E+09	1.25E+09	6.45E+08	6.54E+08	5.39E+08
2.00E+09	2.32E+09	9.70E+08	1.01E+09	7.82E+08
6.98E+08	7.48E+08	3.80E+08	3.88E+08	2.86E+08
1.69E+09	1.76E+09	6.86E+08	8.38E+08	6.51E+08
1.40E+09	2.52E+09	6.66E+08	7.63E+08	5.94E+08
1.02E+09	1.19E+09	6.40E+08	5.74E+08	3.93E+08
3.64E+08	5.17E+08	2.30E+08	2.38E+08	1.82E+08
1.73E+09	2.40E+09	1.19E+09	1.39E+09	1.11E+09
1.43E+09	1.75E+09	8.06E+08	7.94E+08	6.25E+08
1.81E+09	1.57E+09	1.02E+09	1.11E+09	7.22E+08
3.26E+08	3.55E+08	2.09E+08	1.88E+08	1.31E+08
1.85E+09	2.28E+09	8.70E+08	9.71E+08	6.87E+08
9.22E+08	1.09E+09	5.93E+08	5.64E+08	4.51E+08
6.43E+08	6.96E+08	3.75E+08	4.11E+08	2.74E+08
9.46E+08	8.80E+08	6.06E+08	6.63E+08	4.28E+08
3.59E+08	4.62E+08	1.87E+08	2.16E+08	1.82E+08
3.30E+08	3.99E+08	1.88E+08	1.63E+08	1.34E+08
9.89E+08	1.16E+09	4.51E+08	5.02E+08	4.64E+08
6.31E+08	6.71E+08	3.80E+08	3.95E+08	2.75E+08
4.80E+08	5.46E+08	1.59E+08	1.50E+08	1.24E+08
1.87E+08	1.93E+08	1.25E+08	98900000	78800000
1.25E+08	1.39E+08	1.16E+08	1.24E+08	71500000
4.42E+08	5.27E+08	3.16E+08	2.79E+08	2.16E+08
83300000	1.34E+08	2.30E+07	38900000	3.70E+07
7.03E+08	8.19E+08	3.75E+08	3.42E+08	3.37E+08

0	0	2251400	0	0
3.82E+08	3.90E+08	2.21E+08	2.20E+08	1.70E+08
5.89E+08	6.62E+08	3.68E+08	3.26E+08	2.41E+08
1.01E+09	1.54E+09	7.23E+08	6.99E+08	5.16E+08
1.84E+08	1.95E+08	1.43E+08	1.28E+08	50500000
1.34E+08	1.61E+08	85800000	95100000	60200000
3.00E+08	3.22E+08	2.37E+08	1.90E+08	1.50E+08
1.37E+08	1.52E+08	88400000	84700000	43200000
2.46E+08	2.98E+08	1.25E+08	1.28E+08	88600000
3.21E+08	3.82E+08	1.45E+08	1.82E+08	1.39E+08
5302400	5.00E+07	4303700	0	12800000
2.73E+08	3.24E+08	2.30E+08	1.88E+08	1.24E+08
3.27E+08	6.43E+08	2.33E+08	2.21E+08	1.90E+08
3.04E+08	3.70E+08	2.79E+08	2.48E+08	1.88E+08
1.17E+09	1.44E+09	1.14E+09	9.62E+08	5.59E+08
1.96E+08	2.21E+08	1.01E+08	1.37E+08	76600000
7.00E+07	1.16E+08	58200000	5.80E+07	46300000
1.53E+08	1.90E+08	1.02E+08	93400000	61600000
82900000	98500000	42400000	52500000	41500000
95400000	1.37E+08	92300000	85900000	47500000
3.52E+08	3.43E+08	1.68E+08	2.26E+08	1.61E+08
1.34E+08	1.84E+08	1.04E+08	75400000	70200000
2.64E+08	3.28E+08	1.60E+08	1.67E+08	1.33E+08
2.65E+08	3.75E+08	1.63E+08	1.28E+08	1.11E+08
1.90E+08	2.55E+08	74500000	6.70E+07	70300000
6132300	2536500	16100000	0	1659000
1.67E+09	1.94E+09	1.34E+09	1.39E+09	8.37E+08
2.08E+08	2.15E+08	1.57E+08	1.34E+08	85500000
3.01E+08	3.70E+08	2.01E+08	1.87E+08	1.61E+08
2.73E+08	2.85E+08	1.77E+08	1.73E+08	1.13E+08
0	0	0	0	0
48800000	62400000	32800000	42400000	24400000
1.01E+09	6.96E+08	8.26E+08	4.41E+08	3.08E+08
1.95E+08	4.00E+08	1.65E+08	1.88E+08	1.30E+08
5.80E+07	85900000	45500000	20800000	25800000
2.03E+08	2.83E+08	1.33E+08	1.09E+08	9.40E+07
93500000	1.29E+08	67400000	79300000	40300000
14800000	15300000	473810	8241800	7782600
1.77E+08	2.62E+08	1.29E+08	82600000	85600000
44500000	46500000	31100000	30900000	20800000
53600000	73600000	40600000	39400000	32900000
54700000	58100000	3.20E+07	47600000	24700000
2.09E+08	2.11E+08	1.69E+08	7.50E+07	79900000
51800000	89300000	45300000	53300000	3.20E+07
2.30E+08	2.93E+08	1.51E+08	1.33E+08	99900000
20200000	39300000	39100000	20700000	20800000
32500000	47200000	1.10E+07	5465000	0

74800000	1.13E+08	69500000	59800000	37300000
33100000	27300000	12700000	23900000	20800000
1.30E+08	1.48E+08	62200000	56200000	42200000
4.00E+07	33700000	27500000	27100000	10300000
22200000	32500000	16800000	6029100	14700000
1.08E+08	1.51E+08	81600000	89600000	68900000
42900000	57300000	32300000	27400000	19500000
4.13E+08	3.04E+08	2.44E+08	2.30E+08	1.66E+08
72700000	55300000	43200000	41500000	28900000
15300000	0	0	0	2248300
3.72E+08	5.51E+08	1.85E+08	3.26E+08	2.06E+08
70300000	1.04E+08	61300000	46800000	47700000
1.71E+08	2.75E+08	1.48E+08	1.58E+08	1.16E+08
30300000	31900000	29200000	33100000	23500000
2.29E+08	2.49E+08	1.72E+08	1.53E+08	1.09E+08
2.76E+08	2.97E+08	1.52E+08	1.18E+08	88900000
1.27E+08	76300000	2.80E+07	83100000	28600000
1.26E+08	1.29E+08	99900000	97500000	59600000
1.32E+08	1.31E+08	81600000	77800000	41100000
2.45E+08	3.14E+08	1.34E+08	1.14E+08	1.18E+08
0	2339500	6999700	1487400	1168100
0	0	22500000	0	9151300
66900000	50800000	29300000	40900000	31300000
8.50E+07	58800000	74800000	77400000	59600000
62400000	7.40E+07	4.70E+07	44800000	36600000
50600000	9.20E+07	25900000	27900000	16800000
3.13E+08	2.30E+08	1.53E+08	1.26E+08	1.01E+08
2.36E+08	2.88E+08	1.28E+08	1.35E+08	1.07E+08
1.21E+08	95600000	4.70E+07	58900000	41300000
59300000	61200000	18700000	25800000	18400000
1.05E+08	1.53E+08	54100000	55100000	46800000
1.58E+08	1.34E+08	62300000	58400000	54300000
30300000	43700000	2.40E+07	25200000	12200000
25300000	5.20E+07	18800000	29600000	18100000
47300000	73500000	45500000	52100000	45100000
12200000	15700000	10500000	6947500	6423800
7.00E+07	79800000	37200000	29700000	19100000
50700000	54100000	31700000	42300000	29300000
30300000	75800000	26100000	22400000	12400000
1.64E+08	1.55E+08	1.45E+08	1.19E+08	62500000
71300000	1.02E+08	19900000	35700000	35800000
16200000	39600000	12800000	13100000	9574900
4020300	1848300	68300000	59300000	0
86200000	1.13E+08	76500000	56500000	36600000
3.70E+07	2.80E+07	11700000	19400000	8153800
23400000	52300000	20700000	2.00E+07	16200000
37200000	17800000	3929600	7722300	7631700

6965900	18800000	0	2732200	1691000
21200000	27300000	6283600	10100000	2554600
13700000	23600000	13900000	9299100	13500000
34900000	39700000	22400000	28900000	14900000
1.51E+08	1.76E+08	90500000	6.50E+07	67600000
25400000	28500000	3516200	21600000	11800000
10500000	47300000	1.80E+07	8371800	1.10E+07
84300000	92700000	54900000	5.20E+07	36700000
0	0	0	0	0
69100000	3.50E+07	26500000	22600000	2235300
3.52E+08	4.08E+08	2.27E+08	2.19E+08	1.56E+08
50100000	48100000	44600000	28100000	23900000
16800000	17500000	9695700	10700000	2259700
2.12E+08	2.46E+08	1.18E+08	99800000	68100000
31500000	4.20E+07	3.80E+07	40700000	29500000
44400000	76900000	23200000	2.60E+07	14400000
36900000	21400000	17700000	18100000	12800000
7746200	7174400	0	0	2815700
5.20E+07	81500000	26500000	33300000	26200000
31600000	32500000	11900000	14700000	10300000
34200000	56600000	18200000	19300000	15400000
1.61E+08	1.31E+08	1.41E+08	1.33E+08	87400000
0	0	1959200	0	4238500
11700000	21100000	11100000	11500000	4362500
132210	438190	0	0	0
904760	0	0	0	0
40800000	59700000	13600000	23900000	29100000
33900000	31400000	16300000	20200000	9567300
28400000	39700000	32800000	23100000	21100000
0	0	0	0	0
83900000	6.60E+07	34400000	33800000	28800000
80200000	60400000	51400000	26600000	1.20E+07
32100000	6990300	20300000	16200000	7169200
93200000	6.20E+07	38900000	1.70E+07	1.80E+07
43700000	43400000	50100000	55700000	32900000
28300000	27800000	13800000	19900000	11600000
23500000	2.40E+07	21600000	10300000	12700000
15300000	19400000	8043400	10100000	8165200
1.70E+07	21300000	23600000	15900000	11900000
22400000	35100000	12900000	10700000	20600000
41600000	67300000	27800000	21600000	18700000
18500000	26100000	16700000	21500000	5026100
0	20800000	1152400	8494600	2282200
2.21E+08	72700000	3.13E+08	2.09E+08	1.33E+08
21300000	39900000	20300000	23400000	16400000
24500000	33600000	11300000	5861800	13900000
0	0	0	0	974980

2.00E+07	42600000	2.10E+07	19700000	15300000
61200000	78500000	52600000	45100000	36500000
52300000	74200000	4.20E+07	43900000	3.10E+07
0	0	1.20E+07	2702800	0
6.50E+07	64200000	38500000	46800000	22100000
22500000	3.00E+07	16300000	15100000	1.40E+07
1513200	27200000	4590600	1080300	1910300
1.07E+08	1.62E+08	55900000	55900000	51600000
49800000	76600000	4.30E+07	25800000	19600000
1.50E+07	3269500	9661300	8819900	9926300
8838100	1.40E+07	1.20E+07	12500000	7155400
41500000	44700000	18100000	22400000	8723500
0	0	0	0	0
1.15E+08	1.14E+08	80300000	73900000	42100000
14500000	16200000	14600000	9293600	2410000
14800000	24500000	1.60E+07	12600000	6266200
38700000	53200000	1.70E+07	22100000	10900000
24400000	25400000	14100000	12200000	13300000
46800000	63600000	30300000	32300000	24900000
1.06E+08	1.32E+08	72800000	59800000	44100000
1.46E+08	1.24E+08	93700000	69700000	57900000
1.33E+08	1.60E+08	1.07E+08	46900000	5.50E+07
5.61E+08	18600000	7.57E+08	2.92E+08	1.71E+08
1.08E+08	91100000	50300000	32900000	23300000
74700000	73500000	29700000	26300000	22700000
48500000	26800000	11900000	19200000	26200000
0	0	1571500	1808800	1208500
28900000	10100000	41400000	40600000	16700000
39500000	37400000	15400000	16100000	6383100
0	0	0	0	0
20200000	32800000	7680300	10500000	8179400
27800000	34500000	37100000	23300000	21300000
4666800	5418100	0	0	5672800
0	0	194940	0	0
45600000	43600000	13300000	0	1759900
42300000	54500000	17100000	2.50E+07	25300000
27800000	32200000	26700000	22500000	13300000
20600000	23600000	1.80E+07	14100000	8222700
3865500	9913100	8548100	17300000	4090800
33400000	2.20E+07	5265300	19400000	9311800
88300000	1.07E+08	65500000	18300000	36200000
25400000	24400000	11300000	1.40E+07	13200000
80400000	92600000	63700000	54100000	36800000
23800000	30100000	17100000	20600000	17900000
0	0	0	0	0
1.23E+08	64300000	54600000	66700000	3.80E+07
2.10E+07	24500000	8473200	18900000	21400000

10100000	8835200	4339600	10900000	3220500
22700000	3.00E+07	16500000	1.30E+07	11500000
0	0	0	4957700	523320
7985300	15100000	4668400	3852000	5091300
1.13E+08	1.30E+08	62800000	46800000	35400000
15400000	17400000	5744000	11600000	8260100
0	0	0	0	0
0	0	0	0	0
2367900	3643500	2828200	2637700	1346100
5.79E+09	8.74E+09	4.56E+09	2.09E+09	3.21E+09
46500000	44700000	26400000	30400000	22900000
13900000	12800000	10400000	6623700	8464400
33700000	3026600	17500000	0	1648900
27500000	45900000	22200000	21400000	17200000
29700000	17500000	26700000	12800000	8282200
16300000	27500000	15800000	21200000	18500000
0	10900000	0	7052500	0
10100000	7592100	9041400	9855300	4287800
16900000	34200000	10500000	15200000	10700000
12500000	10600000	10900000	6609800	7537000
12900000	21500000	10300000	10700000	1703800
5574400	8090000	6047000	7355700	3353300
15200000	14400000	8390700	3151800	7655300
0	3819300	1474200	9807000	3108700
9833600	15300000	2536300	14400000	5139300
1.65E+08	1.32E+08	88600000	97200000	70700000
2.80E+07	12500000	6738400	12200000	11700000
4161300	19700000	10500000	1104700	13300000
15800000	28500000	10100000	9864200	10600000
9412900	2604600	2721100	2333100	1061100
0	3778500	0	0	0
6270300	0	0	1877200	2004600
25100000	41300000	37900000	23300000	12300000
8368000	9314200	10100000	3902500	2927300
11100000	9413300	7382600	5943000	4096500
17700000	3.10E+07	13100000	4321600	5825900
9999100	4472200	4573000	0	4260100
0	0	0	0	0
0	1007500	0	3294100	2412800
8429800	9806800	9838800	10600000	4383200
4766800	14200000	5149500	4467100	2962100
6447400	12200000	6905400	6827200	4105100
12400000	12200000	8731300	6631100	4220400
4876900	0	0	0	0
10400000	13500000	4608100	9392100	4732100
41900000	4.40E+07	12300000	2.00E+07	17900000
1.10E+07	10400000	1.00E+07	8816600	5847100

40500000	38700000	18700000	2.00E+07	15600000
63300000	88500000	39300000	32400000	25100000
31100000	41900000	23700000	25600000	19800000
465390	5787200	4316800	439120	2280500
12500000	6999200	8607000	7801700	5133700
0	0	1839000	0	0
23300000	20400000	13800000	20400000	10700000
3565700	3323900	0	0	1960500
17200000	12900000	7947300	2890600	5442500
21400000	9440200	4937100	7324300	11100000
0	0	4181000	982100	869920
0	13600000	2189100	8354600	5153900
0	0	0	0	0
2567100	18300000	4300400	9942300	1692200
5995400	1.20E+07	7367300	1094600	5467400
0	0	0	0	0
263800	881390	1319000	0	540380
12200000	11200000	7598900	8159400	5687400
11900000	11100000	4879800	2644700	0
77600000	59500000	19700000	28900000	14900000
1.00E+07	8399100	5613000	0	2317600
21800000	25200000	15800000	14200000	6497200
28500000	30200000	17200000	15100000	13600000
20200000	16200000	4159500	5267200	2679500
7564300	1.10E+07	6203600	3122800	2836300
10900000	16100000	19500000	10300000	1.10E+07
9785500	6282400	8294700	8356700	5163300
11500000	11700000	13600000	9133600	6206500
13800000	29600000	6852400	0	3004400
0	0	2143600	1820400	0
3.00E+07	38200000	27400000	27700000	14100000
38800000	56100000	23800000	0	16200000
7247000	19900000	10700000	9711600	4493200
4932900	9058600	7371300	6947500	7473800
3548000	0	0	445440	3091400
35600000	35400000	46600000	34700000	24200000
0	0	0	0	0
20300000	11500000	0	12200000	4395700
39200000	38900000	30400000	22800000	16900000
40200000	43600000	18300000	20800000	12200000
5884700	10300000	6133900	9737900	5857600
24600000	12500000	12200000	11700000	7608300
1.19E+08	1.91E+08	48500000	59700000	46800000
15300000	26700000	17300000	17800000	9539500
77500000	4531100	0	77700000	2542800
0	0	918460	0	0
1.10E+07	14400000	4422100	4216300	2399400

8506400	13300000	826630	11200000	7903600
6352400	6517000	5766800	4236500	3439600
12700000	12700000	2745000	10800000	6293600
4873800	0	1747300	5833900	936150
7409100	9454900	4341600	4390000	3256300
17100000	12100000	5704800	4330600	2208600
1031800	27100000	1272000	19300000	12500000
2218100	5532200	2224200	7033700	2197900
0	0	0	0	0
2081300	2837000	3577000	0	0
0	0	2518000	0	0
779360	7367600	0	805940	695140
0	10600000	8221300	0	6743000
17200000	2.30E+07	2533000	10400000	4473700
0	9637900	2082900	2044500	1409300
22700000	30600000	14700000	13400000	11600000
31100000	23600000	4006800	14200000	9912100
11400000	1.20E+07	7543000	3810200	3234600
4836000	6879300	12200000	0	6602900
0	0	0	0	0
0	0	0	0	0
3962000	8826900	4708000	15100000	4827500
1456900	10300000	1385800	1386400	2252900
0	7230900	0	0	748250
48800000	54900000	22100000	22100000	1.10E+07
0	0	0	0	0
1178900	1360300	3278900	2385900	1414200
0	0	0	0	0
0	0	3134200	3137500	2467500
0	5.10E+07	0	0	0
0	0	0	0	0
1419100	1660200	1435500	2080000	1439300
24600000	14100000	8929300	15500000	6496700
0	0	0	3324500	2114500
8190200	1.30E+07	0	1333400	1292200
0	8135900	6234600	5716400	4367700
8122800	12200000	6877900	7165900	4996100
19800000	46200000	4514000	6583400	10700000
8177200	14900000	2402300	3695700	5544500
6067900	4415000	2209600	3189900	1670800
12700000	19300000	3088800	5178600	3266600
10300000	10800000	758050	0	3608500
5556200	5538100	2837200	3935200	1718800
0	0	1966200	0	0
4099900	13900000	2251900	2280200	1982400
4670800	17800000	2973200	1618400	5513000
1310800	1594200	8223300	3020500	1025800

5066800	24100000	16400000	16200000	8748400
9306200	9102700	9674600	9208500	4043400
7722900	19400000	15900000	14100000	7004200
3139600	3879600	2306700	2945200	1838500
36700000	41200000	22300000	17500000	12200000
29800000	1.10E+07	6061400	6072600	3562100
9497100	12300000	6983900	7896300	4839400
0	4462000	1898200	0	5500000
0	0	2804400	0	0
5006700	3865200	617230	580090	550970
2083900	0	0	8206800	0
10200000	10900000	6546400	7636800	2384100
7391300	19500000	11700000	1.30E+07	9239700
0	0	0	0	0
8333900	1607700	0	8459600	3342500
0	0	0	6601800	1737200
5761800	0	12900000	12900000	4237800
578010	3894000	0	1517600	4902000
26900000	28700000	8647600	10300000	7157600
0	0	0	0	0
10400000	15300000	11400000	8569100	5123400
0	0	0	2197500	558490
906290	6265100	4699700	767580	726330
0	0	0	0	0
8657600	8847600	6672000	3133300	1636800
3574600	6272300	3910600	5338200	2344100
4243600	6003800	2123300	3595800	2248900
1946300	4090400	5996700	3186700	235940
3073600	2911800	1853200	0	1712000
5481100	11400000	3954000	2749400	4765100
17400000	12300000	7868300	7764000	6838000
782600	1436200	0	0	509270
0	0	0	0	0
0	0	0	0	0
7569500	7629100	1918800	0	0
0	0	0	0	0
2203200	0	3552200	593720	4196700
0	0	0	0	0
0	0	0	0	0
1768300	0	0	0	1910200
0	0	0	0	0
8749700	13100000	9876100	5158800	3594200
9088100	11500000	5307600	1665300	1193300
7524300	1.80E+07	7452100	6322800	3816800
0	0	0	0	0
3397400	7373900	2788900	5265200	2004700
0	5335200	0	0	0

9173600	15800000	76200000	8133800	4364800
1476800	1293200	1079600	1364300	705820
2234100	1690500	874330	1416400	850440
4700200	9588700	4795200	0	1616600
2927200	6130100	4145700	2945200	1495900
3202000	4121900	5522900	5573900	2412200
31100000	0	13100000	17700000	8851500
0	9999300	6083700	8093200	4276500
0	0	0	0	0
1208900	961570	902810	1038600	631310
2399000	8722700	0	1748300	1612700
7278500	13500000	0	0	0
2481400	0	1849300	2906800	0
0	0	0	0	0
18600000	27700000	9743300	7603700	6995400
0	0	0	8485700	5982600
9550300	12500000	6544800	4145500	2275700
10800000	27300000	2243000	9030100	4678900
3901600	0	3168200	4636900	0
0	0	0	0	0
1.80E+07	10700000	15300000	10400000	4393900
0	0	0	0	0
1894400	2438800	2798600	0	0
5084300	7280400	7601000	8127500	5578200
0	0	0	0	0
4722000	6463500	7871400	7543000	3239600
4881300	10500000	2861200	6339300	6892700
9853000	58900000	28800000	7024600	3691000
0	0	0	0	1481900
13700000	16400000	0	0	4748200
234460	766060	415220	364930	636270
3463300	9788900	8047900	3052400	3339500
7151100	5664800	13300000	9990800	7688900
0	0	0	0	0
8308200	0	2158900	0	2162900
0	1059900	5475600	3939900	6298900
81800000	95900000	53900000	7921700	29100000
0	8587000	0	0	0
0	0	0	0	0
21700000	28100000	23700000	19800000	11400000
0	0	0	0	0
17400000	24600000	0	3096500	2749500
18500000	4.10E+07	0	15200000	21100000
4524500	8574000	10900000	4604300	7144800
0	11800000	12500000	8857300	5534700
3511200	5070900	3761300	3539800	3058800
1588600	6417100	1504000	1823300	0

0	0	0	0	0
0	0	0	0	0
12500000	12300000	0	2345300	1442500
465410	2249000	0	0	319870
10400000	12700000	4071400	6393800	6058100
4844500	17400000	3854800	2390400	5356800
11300000	3330400	572830	4407200	4968100
0	0	32600000	931230	0
0	0	0	8313900	0
0	3363600	0	735140	2827600
18400000	8939000	21400000	7259900	5159000
2787300	0	0	0	0
0	0	0	0	0
0	0	0	0	0
0	0	0	0	0
2670900	0	7811500	11900000	0
0	3195500	0	0	0
7832500	8536900	15700000	4819600	5924400
3989400	0	0	2379800	0
1.70E+07	22700000	6405300	9890100	2274000
0	0	0	0	0
0	6919000	5849400	8072100	3451700
0	6652600	0	0	1519400
10900000	13600000	4397800	5895300	6747700
9645300	9803300	2727300	4070900	1375600
0	0	0	0	0
22500000	24600000	17700000	12800000	13100000
3347000	4026500	2781500	2084700	2495000
27700000	22300000	18500000	8343700	7138000
8024600	2.70E+07	25800000	14700000	1.20E+07
0	0	0	0	0
0	0	2912600	1210800	3209700
13800000	1.70E+07	2330300	0	0
10600000	39900000	6110800	7069500	4895300
10200000	0	9744800	926950	815050
0	2888400	4612900	0	2415200
0	5541600	2861200	2946400	6892700
0	1650600	0	1616000	0
0	0	0	0	0
2366700	0	700490	2045400	0
494610	4723300	1334900	1432100	541630
6831300	11700000	7357700	6714800	2712900
6982100	1756400	0	0	178360
18500000	2.60E+07	18200000	24300000	16300000
8146200	4773400	3816200	0	2808100
0	0	0	0	0
2876800	0	3101500	2162300	0

0	0	1794000	0	0
0	0	2898300	3033700	2042600
0	0	0	0	0
0	1220700	0	0	0
13900000	9319600	7169400	6137100	3246000
2016100	2489600	2048800	1987300	1658300
17700000	13300000	7886500	7651500	1430600
1627700	8842400	0	2627300	1846200
0	0	0	0	0
0	0	0	0	0
6564900	0	0	0	0
0	0	0	0	0
5991900	7910500	4412000	3226400	1718700
0	0	0	0	0
0	0	1228000	0	1165000
4889000	5211300	990810	2300300	0
0	0	0	0	0
0	339960	0	0	0
189000	6194900	7120500	5557500	1872000
1666400	2310600	1617800	0	838750
16200000	7662400	0	5532400	5307500
0	0	0	0	0
56400000	7683200	1726200	6048700	2523000
0	4784600	0	1725200	2978900
0	0	0	0	0
3936200	3703000	2653300	2644700	0
0	0	154490	0	0
0	0	4870300	0	993700
0	0	1649200	0	0
5656500	3974000	3360700	4548500	0
2134400	0	4882500	6053300	3288900
0	0	0	4933200	0
0	0	0	9722500	7838700
16700000	33700000	6549100	12300000	4279300
0	0	0	0	0
0	3605300	0	0	0
0	0	2389800	2144700	0
381920	0	0	414420	0
0	7493200	4339100	5855600	2993900
0	0	1691500	1182800	0
0	0	0	0	0
12300000	18200000	13100000	1.20E+07	7494500
1.10E+07	19200000	0	0	0
0	0	0	0	0
10900000	11200000	10100000	9209100	4622400
1395000	525250	1155300	982790	1293500
0	0	0	0	0

0	0	0	0	0
0	0	0	0	0
0	0	0	0	0
498740	0	0	0	0
0	4263000	0	2777000	1942200
5397500	5060600	3367700	4630400	0
12900000	12400000	9040100	7265000	5980100
1127200	1531600	0	0	0
3541600	4044800	0	0	0
0	0	4775900	4722600	0
3506400	5830500	1612600	2281100	4243200
0	961160	4119800	2807200	719850
0	12700000	7861600	7184000	5731400
0	0	20100000	2717600	0
0	0	950550	2574600	1421700
5302400	11200000	1633800	1254400	1591100
0	0	1323000	2189900	0
0	0	0	0	0
0	0	0	0	0
577790	326850	1811600	1597500	607340
0	0	0	0	0
0	0	0	0	0
1055900	1339200	0	424690	0
0	0	4328100	2126700	0
21600000	18100000	0	12200000	8018900
0	0	2760100	3713900	5286100
8639100	10100000	0	5314600	0
0	4837300	1703900	0	0
2393700	0	0	0	0
1.30E+07	12200000	2815400	5785900	4673500
0	10200000	9170100	8020600	0
0	0	0	0	0
0	1489200	0	0	0
0	0	0	0	0
3575900	0	5543400	4091600	4016700
8827700	8313400	3649100	3290700	4916600
8674800	7425900	2290800	231900	5365500
2929000	0	5286400	4762800	4037200
0	0	0	0	0
3377700	2855400	1873700	2581600	1508800
0	0	0	0	2338800
1483100	9547000	4072600	2772600	1836800
0	0	0	0	0
0	0	0	0	0
3641600	3885000	0	0	585330
0	0	4766200	0	0
0	0	0	0	0

0	0	0	0	0
0	0	0	0	0
0	0	492440	0	0
0	0	0	0	0
3.80E+07	11900000	4839100	11500000	7292400
0	0	0	0	0
0	3655100	2695300	0	3377300
2880100	0	1281000	1747100	3169300
0	0	0	0	0
5213100	6688900	3072100	0	1636300
0	0	0	0	0
0	0	0	0	0
0	0	0	0	0
0	0	4468900	0	0
0	7504000	3553600	3563100	2544900
0	4299500	0	2111200	2313800
1444900	0	0	0	0
0	4217100	0	0	0
0	2069800	7895300	5791100	0
0	0	0	0	0
0	7838200	8620500	3153400	0
0	0	1638200	1964400	1894100
0	0	0	0	0
0	2337300	0	888300	0
0	0	0	0	0
0	0	0	0	0
0	0	0	0	0
0	0	1315000	0	0
0	0	0	9601400	0
0	0	0	0	0
0	0	0	0	0
0	0	0	0	1033700
2071500	2073600	0	0	426560
0	0	0	0	0
5426400	0	2411000	0	0
1995400	3368700	1936000	2536200	2301000
0	0	0	0	0
0	0	0	0	0
0	0	0	593350	0
0	0	0	0	0
0	0	0	2570000	1736400
0	0	0	0	0
0	10600000	5685400	4661900	3298900
4569600	0	4608600	4184700	2575200
3407400	6463700	0	1931700	0
5284600	2320700	5822400	4709300	0
0	1.63E+08	3392700	2375100	3820100

0	0	0	0	0
0	0	0	0	0
0	0	0	0	0
0	0	0	0	0
0	0	0	0	637880
9506600	9359500	5906900	5652500	4314100
0	0	0	0	0
18900000	25400000	19900000	19300000	15600000
0	0	0	0	0
0	0	0	0	0
0	0	0	0	0
5198700	6649600	0	0	0
0	0	0	0	0
11200000	10300000	5007700	4529400	3647700
7713200	8831300	10300000	7604300	2663800
0	0	0	0	0
5175800	3372500	6719000	5701100	1901200
0	0	0	0	0
0	9684200	2551200	0	1567600
23900000	27900000	4.80E+07	38600000	24600000
0	0	0	0	0
0	0	0	0	0
0	0	0	0	0
0	0	0	0	0
0	0	0	0	799660
0	3549200	3205400	2302000	4421000
0	0	0	11900000	0
0	0	0	0	0
0	0	0	0	0
0	0	2747200	2447900	1667300
0	0	0	0	0
0	0	7729800	8191500	0
0	0	0	0	0
0	0	0	0	0
0	0	0	0	0
0	0	0	0	0
0	0	0	0	0
0	0	0	0	0
0	0	0	0	0
0	0	3952100	2113300	0
0	0	0	0	0
8281300	7313500	5659700	872940	2778600
0	0	0	0	0
0	0	0	0	0
0	0	0	0	0
0	0	0	0	0
0	0	0	0	0

0	0	0	0	0
0	0	0	0	0
69100000	80600000	52100000	0	25700000
2623100	5032800	1676100	0	1204200
0	0	0	0	0
0	3584600	0	0	0
0	0	0	0	0
0	0	0	0	0
29600000	36800000	29600000	20300000	16800000
0	0	0	0	0
0	0	0	0	0
0	0	0	0	0
0	0	0	0	0
0	0	68300000	59300000	0
0	0	0	0	0
0	0	0	0	0
0	0	0	0	0
0	0	0	0	0
85100000	89100000	71300000	80700000	46300000
0	0	0	0	0
0	0	0	0	0
22500000	2.60E+07	2644400	2199400	5178400
0	0	0	0	0
0	0	0	0	0
1.22E+09	1.33E+09	NA	NA	NA
1.78E+08	3.55E+08	NA	NA	NA
2.09E+10	2.82E+10	NA	NA	NA
5.77E+08	5.82E+08	NA	NA	NA
6.62E+08	7.85E+08	NA	NA	NA
1.75E+09	2.21E+09	NA	NA	NA
4.21E+08	4.74E+08	NA	NA	NA
8.52E+08	1.11E+09	NA	NA	NA
1.92E+08	2.34E+08	NA	NA	NA
2.98E+08	3.44E+08	NA	NA	NA
3117000	0	NA	NA	NA
2.92E+08	3.99E+08	NA	NA	NA
2.65E+08	2.81E+08	NA	NA	NA
4.23E+08	2.91E+08	NA	NA	NA
82600000	1.31E+08	NA	NA	NA
8.00E+07	1.12E+08	NA	NA	NA
2.39E+08	4.03E+08	NA	NA	NA
1.02E+08	1.36E+08	NA	NA	NA
3.03E+08	3.20E+08	NA	NA	NA
34800000	9.20E+07	NA	NA	NA
72700000	62800000	NA	NA	NA
1.41E+08	1.89E+08	NA	NA	NA
1.45E+08	1.43E+08	NA	NA	NA

84600000	22700000	NA	NA	NA
1.65E+08	2.24E+08	NA	NA	NA
84700000	1.17E+08	NA	NA	NA
21800000	11900000	NA	NA	NA
0	1.16E+08	NA	NA	NA
36100000	54100000	NA	NA	NA
13800000	28100000	NA	NA	NA
5.10E+07	83600000	NA	NA	NA
72800000	62500000	NA	NA	NA
2.30E+08	2.93E+08	NA	NA	NA
0	37300000	NA	NA	NA
41200000	71200000	NA	NA	NA
1.01E+08	93800000	NA	NA	NA
3.80E+07	40300000	NA	NA	NA
0	0	NA	NA	NA
24700000	21600000	NA	NA	NA
2.80E+07	23400000	NA	NA	NA
42100000	40800000	NA	NA	NA
24400000	55600000	NA	NA	NA
0	17700000	NA	NA	NA
29600000	38200000	NA	NA	NA
43300000	1.13E+08	NA	NA	NA
56100000	93300000	NA	NA	NA
20800000	33300000	NA	NA	NA
42200000	31900000	NA	NA	NA
48800000	39200000	NA	NA	NA
13800000	39200000	NA	NA	NA
34200000	28300000	NA	NA	NA
24400000	13600000	NA	NA	NA
4637100	20400000	NA	NA	NA
1364400	2980200	NA	NA	NA
48200000	47100000	NA	NA	NA
0	0	NA	NA	NA
0	0	NA	NA	NA
0	1.70E+07	NA	NA	NA
6014600	7538800	NA	NA	NA
13700000	18700000	NA	NA	NA
0	0	NA	NA	NA
2693900	5493500	NA	NA	NA
12300000	14600000	NA	NA	NA
0	0	NA	NA	NA
0	8662500	NA	NA	NA
18300000	4722400	NA	NA	NA
1.77E+08	2.25E+08	NA	NA	NA
3602000	0	NA	NA	NA
31300000	264940	NA	NA	NA
0	2506200	NA	NA	NA

0	0	NA	NA	NA
0	0	NA	NA	NA
1955100	1668100	NA	NA	NA
16400000	10200000	NA	NA	NA
9361300	13700000	NA	NA	NA
2982200	86200000	NA	NA	NA
14600000	0	NA	NA	NA
1780800	5380200	NA	NA	NA
7189300	14300000	NA	NA	NA
3952300	27100000	NA	NA	NA
13200000	4782500	NA	NA	NA
0	0	NA	NA	NA
4672500	0	NA	NA	NA
9767400	15300000	NA	NA	NA
24300000	21800000	NA	NA	NA
3757000	0	NA	NA	NA
5298700	4454300	NA	NA	NA
11400000	6991800	NA	NA	NA
6564100	10100000	NA	NA	NA
3965200	5150800	NA	NA	NA
5426700	15300000	NA	NA	NA
0	0	NA	NA	NA
0	0	NA	NA	NA
0	0	NA	NA	NA
0	0	NA	NA	NA
10100000	15600000	NA	NA	NA
5739300	4524400	NA	NA	NA
14100000	57100000	NA	NA	NA
1.28E+08	0	NA	NA	NA
6575600	0	NA	NA	NA
12100000	46800000	NA	NA	NA
4687500	6991200	NA	NA	NA
6565200	35200000	NA	NA	NA
23600000	2.10E+07	NA	NA	NA
0	11900000	NA	NA	NA
0	8973200	NA	NA	NA
15700000	8229700	NA	NA	NA
0	0	NA	NA	NA
17600000	3492600	NA	NA	NA
0	1352300	NA	NA	NA
16400000	0	NA	NA	NA
0	0	NA	NA	NA
3011900	0	NA	NA	NA
18400000	17200000	NA	NA	NA
4965000	0	NA	NA	NA
0	0	NA	NA	NA
0	0	NA	NA	NA

44600000	2.09E+08	NA	NA	NA
0	0	NA	NA	NA
4750600	1640300	NA	NA	NA
33400000	1.90E+07	NA	NA	NA
5221000	8280800	NA	NA	NA
750550	818330	NA	NA	NA
0	920430	NA	NA	NA
6236000	12800000	NA	NA	NA
0	4109100	NA	NA	NA
0	13800000	NA	NA	NA
38100000	31800000	NA	NA	NA
19800000	31700000	NA	NA	NA
1127100	761430	NA	NA	NA
1987300	2272600	NA	NA	NA
0	0	NA	NA	NA
0	0	NA	NA	NA
8980600	11900000	NA	NA	NA
1214000	5764100	NA	NA	NA
3717800	5727700	NA	NA	NA
0	0	NA	NA	NA
0	0	NA	NA	NA
0	0	NA	NA	NA
0	0	NA	NA	NA
1.52E+08	2.64E+08	NA	NA	NA
0	0	NA	NA	NA
0	8299100	NA	NA	NA
0	0	NA	NA	NA
14600000	13200000	NA	NA	NA
3679600	0	NA	NA	NA
0	0	NA	NA	NA
4111400	4493900	NA	NA	NA
3941200	5392100	NA	NA	NA
3342400	0	NA	NA	NA
0	0	NA	NA	NA
635440	793560	NA	NA	NA
3116600	0	NA	NA	NA
18700000	21100000	NA	NA	NA
0	4102200	NA	NA	NA
0	0	NA	NA	NA
3026700	8696300	NA	NA	NA
35400000	65500000	NA	NA	NA
2924100	1793000	NA	NA	NA
0	0	NA	NA	NA
0	0	NA	NA	NA
0	0	NA	NA	NA
0	8713900	NA	NA	NA
419940	5306000	NA	NA	NA

8958900	0	NA	NA	NA
2543100	0	NA	NA	NA
3362600	0	NA	NA	NA
0	0	NA	NA	NA
0	0	NA	NA	NA
0	4875400	NA	NA	NA
265980	381020	NA	NA	NA
1217500	0	NA	NA	NA
613410	1143300	NA	NA	NA
3755900	0	NA	NA	NA
0	0	NA	NA	NA
0	0	NA	NA	NA
0	4809000	NA	NA	NA
7673100	6766700	NA	NA	NA
0	0	NA	NA	NA
2621000	3445100	NA	NA	NA
0	0	NA	NA	NA
0	0	NA	NA	NA
0	3935500	NA	NA	NA
3717100	3692200	NA	NA	NA
629970	715020	NA	NA	NA
0	0	NA	NA	NA
32300000	23200000	NA	NA	NA
8824500	5049200	NA	NA	NA
0	0	NA	NA	NA
14600000	5481800	NA	NA	NA
0	2308900	NA	NA	NA
0	0	NA	NA	NA
0	0	NA	NA	NA
3051600	0	NA	NA	NA
0	0	NA	NA	NA
4837800	7724200	NA	NA	NA
0	0	NA	NA	NA
2422700	0	NA	NA	NA
0	1585500	NA	NA	NA
4752200	3611300	NA	NA	NA
0	64200000	NA	NA	NA
0	0	NA	NA	NA
0	0	NA	NA	NA
0	0	NA	NA	NA
0	0	NA	NA	NA
0	0	NA	NA	NA
0	0	NA	NA	NA
0	8633700	NA	NA	NA
0	0	NA	NA	NA
0	390480	NA	NA	NA
0	3546600	NA	NA	NA

5556800	8611400	NA	NA	NA
0	3210600	NA	NA	NA
0	3479200	NA	NA	NA
0	0	NA	NA	NA
751580	1835500	NA	NA	NA
0	380250	NA	NA	NA
0	0	NA	NA	NA
0	0	NA	NA	NA
0	3198500	NA	NA	NA
4611400	0	NA	NA	NA
0	0	NA	NA	NA
2699000	3785300	NA	NA	NA
1145600	1811900	NA	NA	NA
1.90E+07	23200000	NA	NA	NA
0	5577500	NA	NA	NA
0	3387000	NA	NA	NA
332650	0	NA	NA	NA
2810200	3991100	NA	NA	NA
0	4970200	NA	NA	NA
8358600	8208100	NA	NA	NA
2822500	0	NA	NA	NA
1651700	621830	NA	NA	NA
0	0	NA	NA	NA
0	0	NA	NA	NA
0	0	NA	NA	NA
0	0	NA	NA	NA
0	1115600	NA	NA	NA
0	0	NA	NA	NA
3282500	0	NA	NA	NA
0	0	NA	NA	NA
0	0	NA	NA	NA
0	0	NA	NA	NA
0	0	NA	NA	NA
6514300	0	NA	NA	NA
0	0	NA	NA	NA
0	3310400	NA	NA	NA
0	0	NA	NA	NA
0	0	NA	NA	NA
0	0	NA	NA	NA
2361900	2587600	NA	NA	NA
0	0	NA	NA	NA
1.29E+08	0	NA	NA	NA
2331600	1152500	NA	NA	NA
2338500	0	NA	NA	NA
0	297880	NA	NA	NA
0	0	NA	NA	NA
0	0	NA	NA	NA

0	0	NA	NA	NA
0	0	NA	NA	NA
0	0	NA	NA	NA
0	0	NA	NA	NA
14500000	0	NA	NA	NA
8067000	16300000	NA	NA	NA
0	0	NA	NA	NA
0	0	NA	NA	NA
0	0	NA	NA	NA
4666700	14600000	NA	NA	NA
0	0	NA	NA	NA
0	0	NA	NA	NA
0	0	NA	NA	NA
5390200	8538000	NA	NA	NA
0	0	NA	NA	NA
0	0	NA	NA	NA
0	0	NA	NA	NA
0	0	NA	NA	NA
0	0	NA	NA	NA
0	3546600	NA	NA	NA
0	0	NA	NA	NA
3389400	7883700	NA	NA	NA
0	0	NA	NA	NA
0	0	NA	NA	NA
0	0	NA	NA	NA
0	0	NA	NA	NA
0	0	NA	NA	NA
39900000	59700000	NA	NA	NA
0	4009900	NA	NA	NA
0	0	NA	NA	NA
0	0	NA	NA	NA
0	0	NA	NA	NA
0	0	NA	NA	NA
0	0	NA	NA	NA
0	5805100	NA	NA	NA
0	0	NA	NA	NA
479940	0	NA	NA	NA
0	0	NA	NA	NA
15400000	16800000	NA	NA	NA
0	4259600	NA	NA	NA
0	0	NA	NA	NA
0	0	NA	NA	NA
0	0	NA	NA	NA
0	0	NA	NA	NA
0	0	NA	NA	NA
0	0	NA	NA	NA
0	0	NA	NA	NA

0	0	NA	NA	NA
0	0	NA	NA	NA
0	0	NA	NA	NA
0	0	NA	NA	NA
0	0	NA	NA	NA
0	0	NA	NA	NA
0	0	NA	NA	NA
0	0	NA	NA	NA
0	0	NA	NA	NA
4335800	0	NA	NA	NA
0	0	NA	NA	NA
58100000	4955000	NA	NA	NA
17700000	20300000	NA	NA	NA
6506100	24800000	NA	NA	NA
0	0	NA	NA	NA
3853300	5069900	NA	NA	NA
0	0	NA	NA	NA
0	0	NA	NA	NA
1.20E+07	0	NA	NA	NA
0	0	NA	NA	NA
0	0	NA	NA	NA
3687600	4033500	NA	NA	NA
0	0	NA	NA	NA
0	4612500	NA	NA	NA
0	0	NA	NA	NA
0	0	NA	NA	NA
0	0	NA	NA	NA
0	0	NA	NA	NA
0	0	NA	NA	NA
0	0	NA	NA	NA
0	0	NA	NA	NA
0	0	NA	NA	NA
0	0	NA	NA	NA
0	0	NA	NA	NA
0	0	NA	NA	NA
0	0	NA	NA	NA
90300000	1.40E+08	NA	NA	NA
263950	421860	NA	NA	NA
0	0	NA	NA	NA
6718100	0	NA	NA	NA
NA	NA	6.35E+08	6.68E+08	5.47E+08
NA	NA	1.50E+10	1.22E+10	1.05E+10
NA	NA	1.19E+09	1.30E+09	9.66E+08
NA	NA	2.72E+08	3.05E+08	2.50E+08
NA	NA	2.45E+08	2.87E+08	1.82E+08
NA	NA	3.88E+08	3.87E+08	2.82E+08
NA	NA	1.96E+08	2.11E+08	1.37E+08

NA	NA	5.33E+08	4.60E+08	4.93E+08
NA	NA	4176600	5789800	1739800
NA	NA	77300000	94100000	77100000
NA	NA	1.89E+08	1.77E+08	1.39E+08
NA	NA	1.92E+08	1.51E+08	1.43E+08
NA	NA	5.50E+07	37700000	37600000
NA	NA	1.59E+08	96100000	58400000
NA	NA	78100000	55900000	46100000
NA	NA	2.53E+08	2.23E+08	1.51E+08
NA	NA	5.40E+07	55100000	6.60E+07
NA	NA	1.78E+08	1.46E+08	87500000
NA	NA	76600000	60400000	50400000
NA	NA	70500000	47500000	36700000
NA	NA	1.78E+08	1.53E+08	1.09E+08
NA	NA	9576900	12800000	3418700
NA	NA	75900000	51100000	38700000
NA	NA	28500000	14300000	16400000
NA	NA	68800000	53800000	51900000
NA	NA	58600000	5.30E+07	34500000
NA	NA	83500000	27500000	5.20E+07
NA	NA	8641500	4186500	2749300
NA	NA	7095500	19800000	12200000
NA	NA	59400000	42400000	34700000
NA	NA	18300000	3.50E+07	9410700
NA	NA	21500000	22700000	15600000
NA	NA	12900000	22800000	10900000
NA	NA	0	0	0
NA	NA	1.14E+08	1.25E+08	1.03E+08
NA	NA	30900000	25300000	17900000
NA	NA	10100000	14100000	12200000
NA	NA	51400000	45700000	31400000
NA	NA	15100000	0	2318700
NA	NA	11600000	11400000	8799400
NA	NA	19500000	26100000	20100000
NA	NA	29700000	30600000	2.50E+07
NA	NA	43100000	14200000	15900000
NA	NA	6231800	7917300	3054500
NA	NA	11400000	9810700	10300000
NA	NA	7871100	5179000	5297800
NA	NA	18800000	25900000	18500000
NA	NA	12700000	13800000	12400000
NA	NA	0	0	2.20E+07
NA	NA	20700000	22600000	26200000
NA	NA	0	0	0
NA	NA	11700000	10700000	4944700
NA	NA	18100000	31400000	15100000
NA	NA	16700000	16700000	1470500

NA	NA	7723000	3477600	1750100
NA	NA	2673800	5575500	0
NA	NA	0	0	0
NA	NA	12900000	13600000	8006700
NA	NA	0	6228000	5260200
NA	NA	0	0	0
NA	NA	11400000	9768400	0
NA	NA	16200000	12700000	10200000
NA	NA	21300000	6379600	4067400
NA	NA	4569200	2043600	0
NA	NA	26500000	40600000	30400000
NA	NA	3338900	1527900	935190
NA	NA	9445500	7950800	5936500
NA	NA	8443000	11100000	8902600
NA	NA	16200000	1.00E+07	7112800
NA	NA	2358900	1406200	821510
NA	NA	5975600	14400000	10500000
NA	NA	17500000	11300000	13400000
NA	NA	1026000	990040	1446700
NA	NA	14700000	7550700	11100000
NA	NA	10800000	12500000	9957900
NA	NA	4675400	21100000	13300000
NA	NA	1519400	5397300	1250400
NA	NA	1372600	1895000	573170
NA	NA	0	0	0
NA	NA	1.96E+08	0	3384700
NA	NA	36700000	43600000	1.90E+07
NA	NA	6867600	10600000	0
NA	NA	3322400	3264000	2860500
NA	NA	0	0	0
NA	NA	3297600	685070	1896800
NA	NA	0	0	0
NA	NA	7596800	10200000	3402400
NA	NA	2862800	0	890200
NA	NA	2174800	6473800	0
NA	NA	3878000	0	1251800
NA	NA	29600000	19700000	22800000
NA	NA	0	0	0
NA	NA	1523300	3974900	2611100
NA	NA	605680	2119800	1623300
NA	NA	2074200	6237300	2792200
NA	NA	4604600	5851900	2400400
NA	NA	13300000	6897500	4078900
NA	NA	0	2589600	0
NA	NA	19900000	17700000	10100000
NA	NA	9597100	11800000	8030200
NA	NA	11500000	9902300	9371500

NA	NA	0	0	0
NA	NA	5357000	5939100	6269600
NA	NA	4923900	3679400	2662900
NA	NA	673860	603130	599990
NA	NA	0	0	371110
NA	NA	15300000	20200000	11100000
NA	NA	3875100	0	1438500
NA	NA	4507800	5585100	4004900
NA	NA	0	0	0
NA	NA	0	0	0
NA	NA	6605700	2498200	7297200
NA	NA	4788000	6927200	1590400
NA	NA	5850900	286460	1065400
NA	NA	20400000	22100000	13700000
NA	NA	16300000	21100000	4605100
NA	NA	21400000	1.80E+07	13100000
NA	NA	2446900	4213100	0
NA	NA	4258800	2844900	2862100
NA	NA	0	326880	312680
NA	NA	0	0	25800000
NA	NA	19200000	27100000	21200000
NA	NA	3189800	8173100	2014500
NA	NA	0	25300000	0
NA	NA	5545000	9652300	9450800
NA	NA	7585100	4484500	2874800
NA	NA	0	0	0
NA	NA	5052200	0	4150100
NA	NA	0	0	0
NA	NA	5208200	9979000	0
NA	NA	4016100	3265800	2804600
NA	NA	3981600	0	0
NA	NA	1854400	2929000	1936700
NA	NA	4511800	6815300	2813900
NA	NA	6352800	4481100	3395800
NA	NA	0	2447800	307750
NA	NA	5224200	4102000	4391000
NA	NA	0	0	0
NA	NA	7405900	6173300	0
NA	NA	2968900	1349000	3563400
NA	NA	4661300	6246900	1977300
NA	NA	752280	0	0
NA	NA	1.30E+07	12800000	0
NA	NA	296560	486720	124590
NA	NA	3814600	3761400	2652300
NA	NA	5779500	2512000	1963300
NA	NA	2927800	5567800	3193100
NA	NA	1960100	2304800	1212000

NA	NA	595120	0	768840
NA	NA	3336300	0	2383700
NA	NA	0	0	0
NA	NA	0	0	0
NA	NA	0	0	0
NA	NA	0	0	0
NA	NA	0	0	0
NA	NA	2447600	0	0
NA	NA	32800000	34100000	1.90E+07
NA	NA	531880	1690100	1698000
NA	NA	3982300	3658000	2182000
NA	NA	0	0	0
NA	NA	5318100	5164900	0
NA	NA	1245400	0	0
NA	NA	0	0	0
NA	NA	5179700	3081200	2754600
NA	NA	1614600	0	0
NA	NA	2366200	0	1502300
NA	NA	5481600	7001400	4899700
NA	NA	655290	1194800	734110
NA	NA	0	2496100	0
NA	NA	0	0	0
NA	NA	0	0	739010
NA	NA	3201800	3297300	1883300
NA	NA	6698200	2830600	1411500
NA	NA	3106900	0	3730300
NA	NA	0	0	0
NA	NA	0	2202700	0
NA	NA	499140	506260	0
NA	NA	0	0	0
NA	NA	0	2505900	0
NA	NA	0	0	0
NA	NA	0	0	0
NA	NA	2155700	0	1732900
NA	NA	0	0	0
NA	NA	6910800	0	0
NA	NA	0	0	0
NA	NA	6718900	9969400	14400000
NA	NA	0	0	0
NA	NA	0	0	0
NA	NA	0	4501600	0
NA	NA	0	1766500	2403600
NA	NA	0	0	0
NA	NA	0	0	0
NA	NA	1787300	2066300	0
NA	NA	3965300	2286200	0
NA	NA	9301100	8403300	0

NA	NA	0	1011600	0
NA	NA	0	0	0
NA	NA	0	0	2969400
NA	NA	0	0	0
NA	NA	2884400	2944500	0
NA	NA	2756600	2594000	0
NA	NA	4.87E+08	4.07E+08	2.77E+08
NA	NA	0	0	0
NA	NA	0	2225300	0
NA	NA	0	0	0
NA	NA	0	0	0
NA	NA	3.35E+09	9.13E+08	2.01E+09
NA	NA	1809600	1715900	2189100
NA	NA	0	0	0
NA	NA	0	0	0
NA	NA	0	0	0
NA	NA	0	0	0
NA	NA	0	0	1906900
NA	NA	0	2516700	0
NA	NA	2410300	2639100	4333200
NA	NA	0	738470	388560
NA	NA	0	0	1224200
NA	NA	3131400	3793900	1725900
NA	NA	2728100	0	0
NA	NA	238620	0	0
NA	NA	0	0	0
NA	NA	0	0	0
NA	NA	1478300	0	0
NA	NA	0	0	0
NA	NA	1175000	0	0
NA	NA	0	0	0
NA	NA	2814200	0	0
NA	NA	0	0	0
NA	NA	2205900	0	0
NA	NA	0	0	0
NA	NA	0	0	0
NA	NA	0	0	0
NA	NA	721640	713800	387730
NA	NA	0	0	0
NA	NA	0	0	0
NA	NA	0	1488800	0
NA	NA	2728100	0	0
NA	NA	0	0	0
NA	NA	0	0	0
NA	NA	0	0	0
NA	NA	3309400	2846600	1421300
NA	NA	0	0	0

NA	NA	1635400	0	0
NA	NA	8349200	0	0
NA	NA	0	0	0
NA	NA	0	0	0
NA	NA	0	0	0
NA	NA	0	0	0
NA	NA	0	0	0
NA	NA	0	0	0
NA	NA	13100000	0	0
NA	NA	6392800	4183900	4012600
NA	NA	0	0	0
NA	NA	0	0	0
NA	NA	2401400	0	0
NA	NA	0	0	0
NA	NA	0	0	0
NA	NA	9953200	0	4837500
NA	NA	1206600	0	0
NA	NA	0	0	0
NA	NA	542660	612890	564120
NA	NA	0	0	0
NA	NA	0	0	0
NA	NA	0	0	0
NA	NA	0	0	0
NA	NA	0	0	0
NA	NA	684880	4817100	0
NA	NA	0	0	0
NA	NA	0	0	5009300
NA	NA	0	0	0
NA	NA	0	0	0
NA	NA	0	0	0
NA	NA	21700000	0	0
NA	NA	31600000	15700000	13900000
NA	NA	0	0	0
NA	NA	0	0	0
NA	NA	992930	0	0
NA	NA	72400000	64600000	41200000
NA	NA	0	0	3920400
NA	NA	0	0	0
NA	NA	0	0	0
NA	NA	0	0	0
NA	NA	0	0	0
NA	NA	9699500	9633300	7787200
NA	NA	0	0	0
NA	NA	0	1632800	0
NA	NA	5760000	6982200	0
NA	NA	15400000	13800000	7750400
NA	NA	5179700	6945500	5924400

NA	NA	0	1874600	0
NA	NA	0	0	0
NA	NA	0	0	0
NA	NA	0	0	0
NA	NA	0	0	0
NA	NA	0	0	0
NA	NA	0	0	0
NA	NA	2026300	0	0
NA	NA	8744700	7826000	5355500
NA	NA	0	0	0
NA	NA	0	0	0
NA	NA	2536300	7249300	2349600
NA	NA	0	12500000	6168200
NA	NA	0	0	0
NA	NA	0	0	0
NA	NA	1098900	1291400	4499000
NA	NA	16600000	7762400	6883700
49900000	61400000	NA	NA	NA
NA	NA	1824000	0	0
NA	NA	1.50E+07	4408200	7479200
1112200	0	NA	NA	NA
NA	NA	0	0	737300
0	0	NA	NA	NA
0	0	NA	NA	NA
NA	NA	7128600	0	0
NA	NA	1306500	1574900	2397100
87400000	1.34E+08	NA	NA	NA
0	15600000	NA	NA	NA
3248000	0	NA	NA	NA
NA	NA	0	4019900	1.60E+07
1.17E+09	1.29E+09	NA	NA	NA
NA	NA	4499000	6627200	4009100
0	0	NA	NA	NA
3.35E+08	2.72E+08	NA	NA	NA
NA	NA	9533500	8759300	9033600
20700000	28800000	1.80E+07	7662400	10400000
NA	NA	18200000	27200000	16800000
90200000	98700000	NA	NA	NA
11500000	14100000	NA	NA	NA
4867200	86100000	NA	NA	NA
NA	NA	0	0	0
8873900	8109400	NA	NA	NA
0	5301400	NA	NA	NA
7.59E+08	8.87E+08	NA	NA	NA
NA	NA	2.80E+07	23900000	14500000
NA	NA	2170600	1377300	780970
NA	NA	3603100	15800000	0

673820	11100000	NA	NA	NA
6090700	9680700	NA	NA	NA
55800000	71500000	NA	NA	NA
9907900	9136800	NA	NA	NA
NA	NA	0	0	0
