# Supplementary material for: Standardizing Proteomics Workflow for Liquid Chromatography-Mass Spectrometry: Technical and Statistical Considerations
Source: J Proteomics Bioinform. Author manuscript; Available in PMC 2020 Mar 6. (PMC7059694; doi:10.35248/0974-276x.19.12.496)
Supplement: suppl file 2 — More details about experimental procedures. [file NIHMS1032169-supplement-suppl_file_2.pdf]

Experimental Procedures

Liquid Chromatography

Peptide separation was achieve using a Dionex Acclaim PepMap 100 75µm x 2cm, nanoViper (C18, 3µm, 100Å) trap, and a Dionex Acclaim PepMap RSLC 50µm x 15cm, nanoViper (C18, 2µm, 100Å) separating column. An EASY n-LC (Thermo) UHPLC system was used to resolve peptide separation using a 140min linear gradient from 2% v/v acetonitrile / 0.1% v/v formic acid to 40% v/v acetonitrile / 0.1% v/v formic acid. Peptides were introduced into the Orbitrap ELITE MS using a 40mm stainless steel emitter (Thermo P/N ES542) and a Nanospray Flex source (Thermo) was used to position the end of the emitter near the ion transfer capillary of the mass spectrometer.

Mass Spectrometry Data Acquisition

MS data was collected using an Nth Order Double Play with ETD Decision Tree method created in Xcalibur v2.2. Scan event one of the method obtained a Fourier transform MS MS1 scan (normal mass range; 60,000 resolution, full scan type, positive polarity, profile data type) for the range 300-2000m/z. Scan event two obtained ion trap MS MS2 scans (normal mass range, rapid scan rate, centroid data type) on up to twenty peaks that had a minimum signal threshold of 5,000 counts from scan event one. A decision tree was used to determine whether collision-induced dissociation (CID) or Electron-transfer dissociation (ETD) activation was used. An ETD scan was triggered if any of the following held: an ion had charge state 3 and m/z less than 650, an ion had charge state 4 and m/z less than 900, an ion had charge state 5 and m/z less than 950, or an ion had charge state greater than 5; a CID scan was triggered in all other cases. The lock mass option was enabled (0% lock mass abundance) using the 371.101236m/z polysiloxane peak as an internal calibrant.

Data Analysis with Proteome Discoverer v1.4.1.14 and Scaffold Q+S v4.4.3

Proteome Discoverer v1.4.1.114 was used to analyze the data collected by the mass spectrometer. The database used in Mascot v2.5.1 and SequestHT searches was a 4/7/2015 version of the UniprotKB Homo sapiens reference proteome canonical and isoform sequences with the 1/1/2012 version of the common Repository of Adventitious Proteins (cRAP) database (thegpm.org) appended to it (the cRAP database contains common contaminant proteins observed in mass spectrometry experiments). To estimate the FDR, a Target Decoy Peptide-Spectrum Match Validator node was included in the Proteome Discoverer workflow.

The Proteome Discover was used for extraction of MS2 scan data from the Xcalibur RAW file, separate searches of CID and ETD MS2 scans in Mascot and Sequest, and collection of the results into a single file (.msf extension) prior to loading into Scaffold Q+S v4.4.3. The FDR for peptides was calculated using the Scaffold Local FDR algorithm. Protein probabilities were calculated using the Protein Prophet algorithm. Proteins were grouped by the Scaffold protein cluster analysis to satisfy the parsimony principle. Label-free quantification of identified proteins were exported as total precursor ion area values to an excel sheet for analysis of proteomics data (Supplementary file 1).
